# *Pseudomonas aeruginosa* DesB Promotes *Staphylococcus aureus* Growth Inhibition in Coculture by Controlling the Synthesis of HAQs

**DOI:** 10.1371/journal.pone.0134624

**Published:** 2015-07-31

**Authors:** Sejeong Kim, Yohan Yoon, Kyoung-Hee Choi

**Affiliations:** 1 Department of Food and Nutrition, Sookmyung Women’s University, Seoul, Korea; 2 Department of Oral Microbiology, College of Dentistry, Wonkwang University, Iksan, Korea; Indian Institute of Science, INDIA

## Abstract

*Pseudomonas aeruginosa* is a pathogen that can cause serious infections and usually coexists with other pathogens, such as *Staphylococcus aureus*. Virulence factors are important for maintaining a presence of the organisms in these multispecies environments, and DesB plays an important role in *P*. *aeruginosa* virulence. Therefore, we investigated the effect of DesB on *S*. *aureus* reduction under competitive situation. Liquid cultures of *P*. *aeruginosa* wild type (WT) and its *desB* mutant were spotted on agar plates containing *S*. *aureus*, and the size of the clear zones was compared. In addition, interbacterial competition between *P*. *aeruginosa* and *S*. *aureus* was observed over time during planktonic coculture. The transcriptional profiles of the WT and *desB* mutant were compared by qRT-PCR and microarray to determine the role of DesB in *S*. *aureus* reduction at the molecular level. As a result, the clear zone was smaller for the *desB* mutant than for *P*. *aeruginosa* PAO1 (WT), and in planktonic coculture, the number of *S*. *aureus* cells was reduced in the *desB* mutant. qRT-PCR and microarray revealed that the expression of MvfR-controlled *pqsA-E* and *phnAB* operons was significantly decreased, but the *mexEF-oprN* operon was highly expressed. The results indicate that intracellular levels of 4-hydroxy-2-heptylquinoline (HHQ), a ligand of MvfR, are reduced due to MexEF-OprN-mediated efflux in *desB* mutant, resulting in the decrease of MvfR binding to *pqsA-E* promoter and the reduction of 4-hydroxy-2-alkylquinolines (HAQs) synthesis. Overexpression of *mexEF-oprN* operon in *desB* mutant was phenotypically confirmed by observing significantly increased resistance to chloramphenicol. In conclusion, these results suggest that DesB plays a role in the inhibition of *S*. *aureus* growth by controlling HAQ synthesis.

## Introduction

The gram-negative opportunistic pathogen *Pseudomonas aeruginosa* is a causative agent of nosocomial and life-threatening infections in injured, burned, and immunocompromised patients [1]. This human pathogen produces multiple extracellular factors, such as elastases, proteases, and rhamnolipids that break down host proteins, such as elastin and collagen, as well as phospholipids in the lungs, which consequently impair host tissue function [2]. In addition, the pathogen releases a variety of virulence factors, such as exotoxins, pyocyanin, proteases, hemolysins, and quorum sensing (QS) molecules, such as pseudomonas quinolone signal (PQS) to infect host cells or outcompete other microorganisms in mixed microbial communities [3,4,5].

In clinical settings, most microbes exist primarily in polymicrobial communities, which affect interspecies interaction and alter clinical outcomes. Bacterial pathogens such as *Staphylococcus aureus* and *Candida albicans* are commonly isolated from clinical samples along with *P*. *aeruginosa* [6,7,8]. In polymicrobial infections involving *P*. *aeruginosa*, synergistic, mutual interactions that contribute to disease pathogenesis are frequently observed. Microbes in mixed communities are capable of enhancing their own growth, virulence, and persistence [6]. Therefore, studies with single-species-based analysis are not relevant to clinical conditions. Mixed infections with *P*. *aeruginosa* and *S*. *aureus* are more virulent than single-species infections, cause more severe disease, and are frequently associated with chronic wound and lung infections [6,9,10]. Nevertheless, in this ecological niche, the relationship between *P*. *aeruginosa* and *S*. *aureus* is competitive rather than cooperative. *P*. *aeruginosa* secretes toxic substances, such as alkyl-hydroxyquinoline N-oxides, hydrogen cyanide, and pyocyanin, that impede the proliferation of *S*. *aureus* [6,11]. In addition, *P*. *aeruginosa* strains that produce LasA endopeptidase induce the lysis of *S*. *aureus* by cleaving specific bonds in its peptidoglycan, further promoting *P*. *aeruginosa* growth [12]. During *in vivo* coculture, lysed *S*. *aureus* cells provide useable iron for *P*. *aeruginosa* growth under low-iron conditions [13]. In addition, peptidoglycan released from *S*. *aureus* can stimulate the production of several virulence factors, including pyocyanin and elastase, by *P*. *aeruginosa* and enhances its virulence in a *Drosophila* infection model [14]. Therefore, in a polymicrobial community, *P*. *aeruginosa* exhibits increased virulence in the presence of *S*. *aureus*.

However, the growth of *S*. *aureus* is not completely inhibited by *P*. *aeruginosa*. *S*. *aureus* has defense mechanisms that help the organism outcompete *P*. *aeruginosa* in the same infection; thus, it coexists as a persister [15]. For example, 4-hydroxy-2-heptylquinoline-N-oxide (HQNO) produced by *P*. *aeruginosa* inhibits the growth of *S*. *aureus* strains, and leads to the development of small-colony variants (SCVs) that are resistant to antibiotics and contribute to bacterial persistence [16,17].

Virulence factor production by *P*. *aeruginosa* is extremely important for growth and pathogenesis in multispecies environments. Our previous studies demonstrated that *P*. *aeruginosa* DesB, an aerobic desaturase, plays an important role in virulence [18]. A mutant harboring a transposon insertion in the *desB* gene exhibited significantly reduced production of various exoproducts, including pyocyanin, protease, elastase, and rhamnolipids, as well as decreased motility [18]. In addition, a *Caenorhabditis elegans* infection study demonstrated that DesB is involved in virulence [18]. Similarly, a study using transposon site hybridization (TraSH) method in a mouse infection model showed that *Mycobacterium bovis* DesA3, a membrane-bound aerobic desaturase, is also necessary for survival and pathogenesis [19]. However, the role of DesB in interspecies interactions during coculture with other pathogens has not yet been studied.

Therefore, in this study, we aimed to determine if DesB plays a role in the relationship between *P*. *aeruginosa* and *S*. *aureus* during coculture, and if so, what role does it play.

## Materials and Methods

### Bacterial strains and culture conditions

The bacterial strains used in this study are listed in Table 1. All bacterial strains were kind gift. Among these strains, *desA*, *desT*, and *fabA* mutants are *P*. *aeruginosa* PAOl harboring each truncated gene, and *desB* mutant harbors an insertion of an IS*lacZ*/*hah* transposon in *desB* gene. The strains were routinely maintained on Luria-Bertani medium (LB; 10 g/L tryptone, 5 g/L yeast extract, 10 g/L NaCl; Difco) and grown at 37°C.

**Table 1:** Bacterial strains used in this study.

Strain		Relevant characteristics^1^	Reference
*P*. *aeruginosa*	PAO1	Prototroph	[20]
	PAO482	PAO1 Δ*desT*::*FRT*	[21]
	PAO651	PAO1 Δ*desA*::*FRT*	[21]
	PAO652	PAO1 Δ*fabA*::*FRT*	[21]
	13272	Tc^r^; PAO1 *desB*::IS*lacZ*/*hah*	[21]
	PAO739	Tc^r^; 13272 Δ*fabA*::*FRT*	[21]
*S*. *aureus*	ATCC25923	Wild-type strain, clinical isolate	FDA strain, Seattle 1945

^1^Abbreviations: r, resistant; Tc, tetracycline; *FRT*, FLP recognition target.

### Lysis of *S*. *aureus*

*S*. *aureus* was incubated in LB broth at 37°C for 18h. This overnight culture was then diluted to 1:25 with fresh LB broth and incubated to mid-log growth phase. An aliquot of this subculture then was mixed with 2 mL of LB medium containing 0.8% agar to an optical density at 600 nm (OD_600_) of 0.2. After allowing the plate to solidify, 3 μL of an overnight culture of *P*. *aeruginosa* was spotted onto the plate, and it was incubated at 37°C for 24 h. After incubation, the plate was imaged using a universal digital camera.

### Interspecies growth competition assay using planktonic cultures

The interbacterial competition assay was performed as described previously with minor modifications [22]. *S*. *aureus* and *P*. *aeruginosa* strains were streaked on LB agar plates and incubated at 37°C for 24 h. The next day, colonies of approximately the same size were selected from the plates, inoculated in 5 mL of LB broth, and incubated for 18 h. The overnight cultures were washed with 1 mL of PBS and resuspended to an OD_600_ of 1.0 and 2.5 for *P*. *aeruginosa* and *S*. *aureus*, respectively. *P*. *aeruginosa* and *S*. *aureus* were mixed at 1:1 (vol/vol). A 10-μL aliquot of the mixture was spotted on a cellulose acetate filter disc and placed on the LB agar plate, which was incubated at 37°C. The growth of individual bacterial species was analyzed by resuspending the filter disc in 0.5 mL of PBS and plating the suspension on Cetrimide agar (CA; Sigma, St. Louis, MO, USA) plates for *P*. *aeruginosa* and on Mannitol salt agar (MSA; Difco) plates for *S*. *aureus*.

### Minimum inhibitory concentration (MIC) determination

*P*. *aeruginosa* strains were grown overnight (18 h) and subcultured in 5 mL of sterile LB broth to log phase (OD_600_ 0.7–1.0). The culture was diluted with LB broth to OD_600_ 0.1. Then, 100 μL aliquots of serial two-fold dilutions of chloramphenicol in LB broth were prepared in a 96-well plate at final concentrations of 0–512 μg/mL, and an equal volume of bacterial culture (at OD_600_ 0.1) was added. After a 24-h incubation, inhibition of *P*. *aeruginosa* growth was assessed by measuring the OD_600_ using a microplate reader (Bio Tek Instruments, Inc., Winooski, VT, USA). The MIC was defined as the concentration at which was no growth was observed.

### Serial dilution spotting assay

*P*. *aeruginosa* strains were cultured in LB broth at 37°C for 18 h, and then the overnight culture (approximately density: 10^8^ CFU/mL) was serially diluted (10-fold, 10^0^−10^6^ CFU/mL) in PBS. 2 μL of the diluents was vertically spotted on LB agar containing 0, 8, 16, 32, 64, or 128 μg/mL chloramphenicol. After 24 h of incubation at 37°C, spot formation was assessed, and the chloramphenicol resistance of the WT and *desB* mutant strain was compared.

### Quantitative RT-PCR (qRT-PCR)

*P*. *aeruginosa* colonies freshly grown on LB agar plates were inoculated into 5 mL of LB broth and incubated at 37°C for 18 h. These overnight cultures were diluted 1:25 in fresh LB broth, and grown to an OD_600_ of 0.4–0.5. Total RNA was extracted from 1 mL cultures of *P*. *aeruginosa* strains using the Qiagen RNase mini kit (Qiagen, Hilden, Germany) according to the manufacturer’s instructions. Then, one U of RNase-free DNase I (Invitrogen, CA, USA) was added to one μg of extracted total RNA, and the reaction mixture was incubated for 15 min at room temperature. Next, the enzyme was inactivated by adding 1 μL of 25 mM EDTA, and heating the mixture at 65°C for 10 min. cDNA was synthesized from the RNA using the Superscript III first-strand synthesis system (Invitrogen). The PCR reaction mixture (20 μL) contained 10 μL of VeriQuest SYBR Green qPCR master mix (USB, Affymetrix), 1 μL of 10 μM forward primer, 1 μL of 10 μM reverse primer, 2 μL of cDNA, and 6 μL of sterile distilled water. The reaction mixtures were denatured by incubation at 95°C for 10 min, which was followed by 35 cycles of 95°C for 15 s and at 60°C for 1 min. The target genes *mvfR*, *pqsA*, *mexE*, *mexF*, *mesT*, and *oprN* were amplified using the designed primers listed in Table 2. The constitutively expressed housekeeping gene *rpoD* was used to normalize gene expression. The relative expression of the genes was obtained from the calculated Ct values.

**Table 2:** Primers used for qRT PCR.

Gene (Product size)	Primer name	Sequence (5ʹ-3ʹ)
*pqsA* (71 bp)	pqsA-F	CTGGACGACAACCAGATCCT
	pqsA-R	ATGTGCGAGGGAATCTGTTC
*mvfR* (96 bp)	mvfR-F	CGTACTGCTCGACGATTTCA
	mvfR-R	ATATCGATTTCCGCGTTGTC
*mexE* (88 bp)	mexE-F	CACCCTGATCAAGGACGAAG
	mexE-R	GCGGTAGACGGTCTTGTTGT
*mexF* (100 bp)	mexF-F	TCTACGACCCGACCATCTTC
	mexF-R	AGGAACAGGATCACCACCAG
*mexT* (80 bp)	mexT-F	GCCGCGCCAACTATCTATT
	mexT-R	CAGTTCGTCGGTGTAGCTGA
*oprN* (87 bp)	oprN-F	GCAACCTGGAGAACCAGAAG
	oprN-R	CGCGCAGTACGTCGAGTT
*rpoD* (75 bp)	rpoD-F	CTGCAATTCCTCGACCTGAT
	rpoD-R	GCGACGGTATTCGAACTTGT

### Microarray analysis

Total RNA was prepared from the *P*. *aeruginosa* strains using the same as procedure as described for qRT-PCR, except that a greater volume of culture was used. The quality of the purified RNA was confirmed using an Agilent 2100 Bioanalyzer System. cDNA was generated and labeled using the Bioprime labeling kit (Invitrogen), and the microarray hybridization was performed using Hybridization solution (MYcroarray.com). The microarray data were normalized and analyzed using Genowiz 4.0 (Ocimum Biosolutions, India). Total 5544 genes were analyzed, and among these, the comparative transcriptional profile of HAQ-related genes between WT and *desB* mutant was used for data interpretation.

### Statistical analysis

Interspecies growth competition assay was repeated with two samples in each repeat, and MIC assay was repeated with six samples in each repeat. The bacterial cell counts (log CFU mL^-1^) and OD values at several timepoints or chloramphenicol concentrations were analysed by the mixed model procedure (SAS Institute, Cary, NC, USA). Pairwise t-tests were performed for comparisons of least squares means among the interactions at alpha = 0.05. All experiments except for microarray analysis were performed in replicate, but microarray data were confirmed by qRT-PCR.

## Results and Discussion

### DesB is involved in *S*. *aureus* reduction

**Fig 1:**
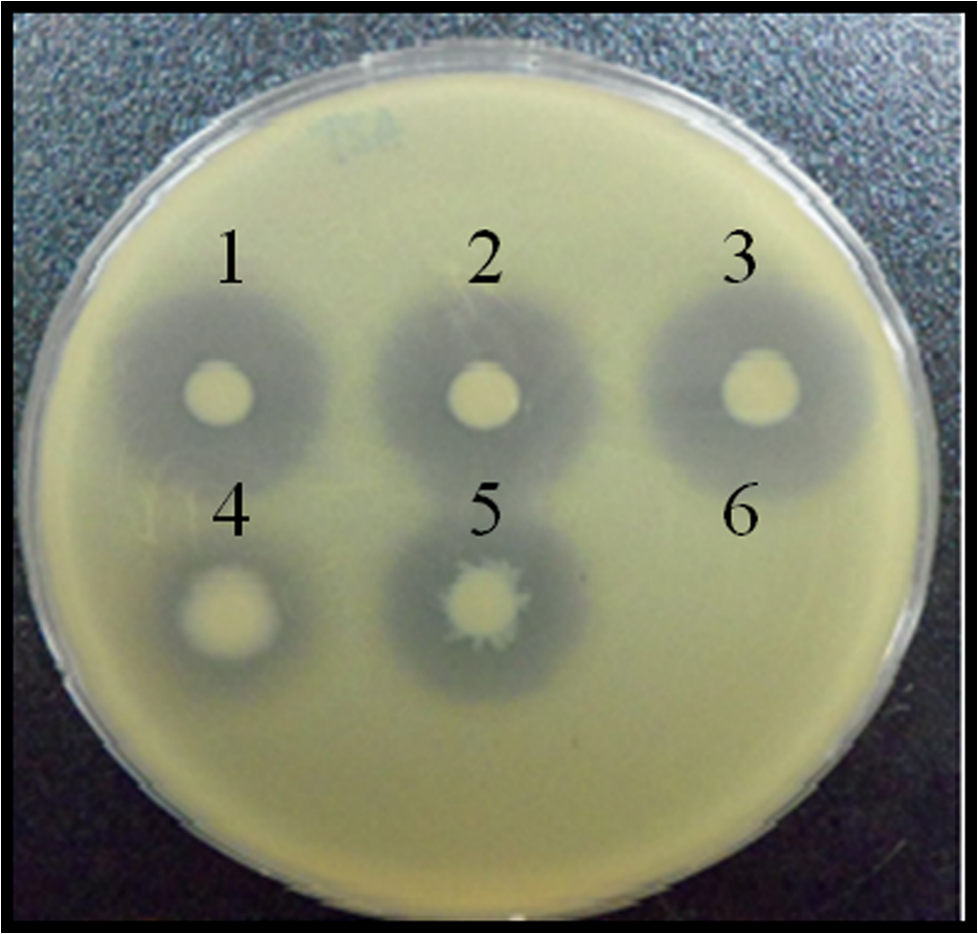
Spot assay for *Staphylococcus aureus* reduction. Overnight cultures of *Pseudomonas aeruginosa* PAO1 strains (wild type [1] and various mutants Δ*desT* [2], Δ*desA* [3], *desB* [4], Δ*fabA* [5], and Δ*fabA desB* [6]) were spotted onto an LB agar plate containing *Staphylococcus aureus* ATCC25923. After incubation at 37°C for 24 h, the diameter of the clear zone was measured.

Other microbes are usually detected in *P*. *aeruginosa* infections. It was previously shown that *P*. *aeruginosa* lyses *S*. *aureus* during coculture [23]. *P*. *aeruginosa* and *S*. *aureus* affect each other, and interspecies communication is required for the expression of various virulence factors [24]. Certain virulence factors obstruct the proliferation of *S*. *aureus* by lysis or growth inhibition. Schweizer and Choi [18] found that DesB, an aerobic desaturase expressed by *P*. *aeruginosa*, is closely associated with the production of virulence factors, particularly elastase, rhamnolipids, and pyocyanin. Therefore, we hypothesized that DesB also plays a role in *S*. *aureus* reduction under mixed culture conditions. Accordingly, we conducted comparative analyses of the virulence of WT and *desB* mutant strains during coculture with *S*. *aureus*. In addition to *desB*, genes involved in unsaturated fatty acids (UFAs) synthesis, such as *desA*, *desT*, and *fabA* was also evaluated in order to investigate if UFA synthesis is involved in *S*. *aureus* reduction. In the spot assay, the *desB* mutant showed clear zones with smaller diameters than those surrounding the WT strain (Fig 1), whereas the *desA* (phospholipid acyl desaturase) and *desT* (TetR family transcriptional regulator) mutants showed clear zones of similar diameter to those surrounding the WT strain. In addition, the *fabA* mutant showed slightly lower activity of cell number decrease than the WT strain, and the *fabA desB* double mutant showed no *S*. *aureus* reduction (Fig 1). The *P*. *aeruginosa fabA* gene encodes *β*-hydroxydecanoyl-ACP dehydrase, which is involved in fatty acid synthesis under aerobic and anaerobic conditions [25]. In our previous study, we found that the *fabA* mutant used in the present study has a very low growth rate compared to other mutants, including *desA*, *desB*, and *desT* mutants [21]. Therefore, the decreased *S*. *aureus* reduction ability of the *fabA* mutant, and the zero-activity of the *fabAdesB* double mutant are not due to a deficiency of FabA activity but result from the slow-growth phenotype of the *fabA* mutant. This result indicates that DesB is an important factor for *S*. *aureus* reduction.

**Fig 2:**
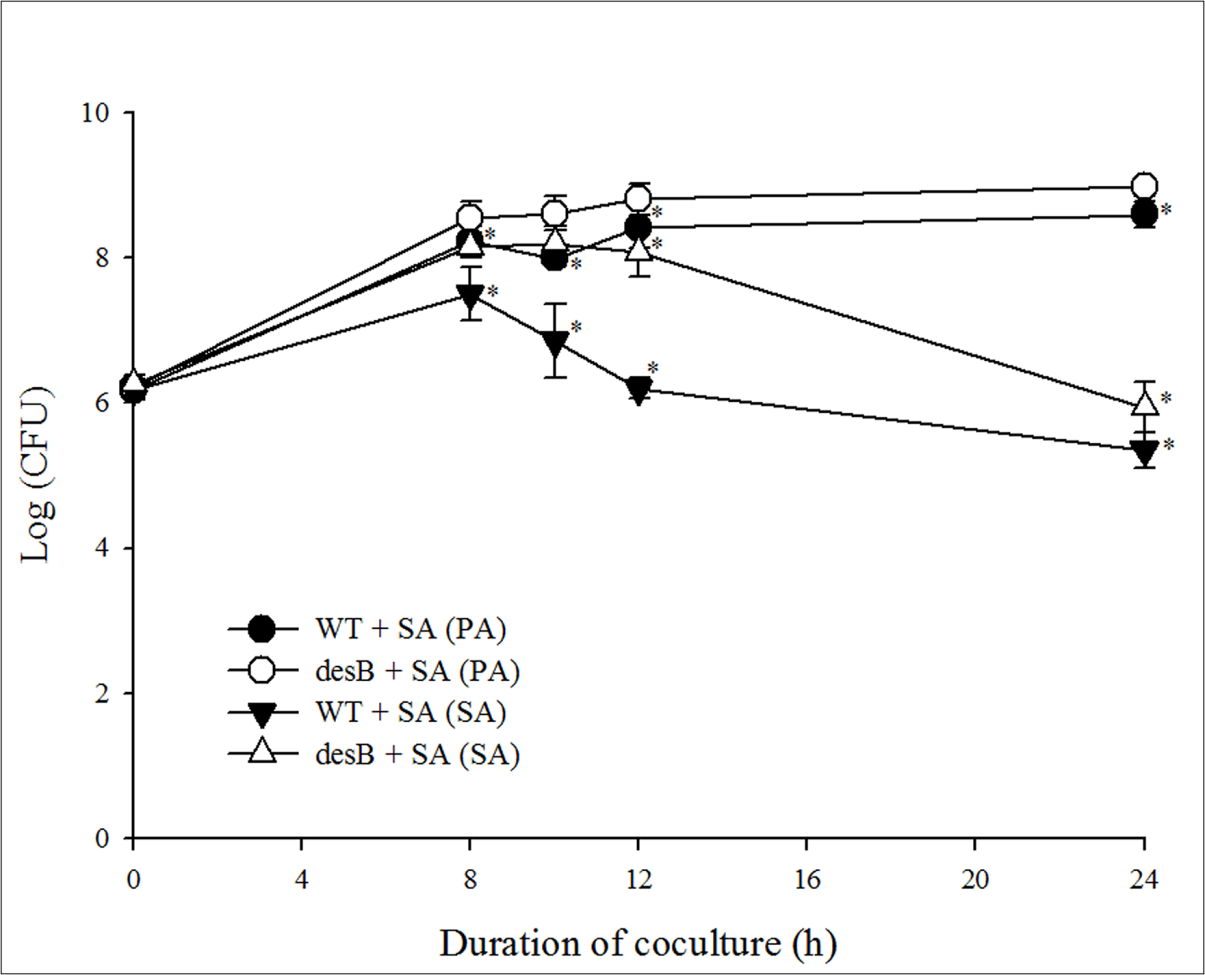
Interspecies growth competition assay. Mixtures of *P*. *aeruginosa* (PA) wild type (WT) or *desB* mutant (*desB*) strains and *S*. *aureus* (SA) were spotted on a cellulose acetate filter disc and placed on an LB agar plate. During incubation, the growth of the individual bacterial species was analyzed by resuspending the filter disc in 0.5 mL of PBS and plating the suspension on selective agar. Means with (*) are significantly different (*P* < 0.05) in same timepoint.

Furthermore, a time-course growth competition assay between *P*. *aeruginosa* and *S*. *aureus* was conducted to confirm the deficiency phenotype of *S*. *aureus* reduction in the *desB* mutant in planktonic culture. Mutation of the *desB* gene did not influence the growth of *P*. *aeruginosa*; however, it did affect the extent of *S*. *aureus* reduction in coculture (Fig 2). The total *S*. *aureus* cell count decreased after 10 h of coculture with the WT strain, whereas a decrease in the number of *S*. *aureus* cells was observed after 12 h of coculture with the *desB* mutant strain, suggesting that *S*. *aureus* reduction by the *desB* mutant was retarded due to the lack of functional DesB. In addition, a greater number of *S*. *aureus* cells were maintained in a coculture with the *desB* mutant than in a coculture with WT over the same incubation period. However, comparatively smaller difference between two strains was observed at the 24 h timepoint than at the 10 h and 12 h timepoints. It can be inferred that the phenotype of *desB* mutant at this timepoint was attributed to delayed production of lysis- or growth inhibition-associated virulence factors.

Also, it may be possible that *S*. *aureus* responds to WT or *desB* mutant in terms of SCV formation in *S*. *aureus* under coculture condition. Pyocyanin stimulates SCV selection in *S*. *aureus* and the SCVs appeared after 24h of cocultivation [11]. Since *desB* mutant displayed reduced pyocyanin production [18], SCVs may be formed less in *desB* mutant than in WT. However, SCVs were not found in this study because coculture experiment was conducted until 24 h of coculture.

This indicated that *P*. *aeruginosa* DesB is involved in *S*. *aureus* reduction. *P*. *aeruginosa* produces various extracellular antimicrobial substances associated with a decrease in *S*. *aureus* cell number that are mostly regulated by the *pqsA-E* operon [26–28]. In addition, *P*. *aeruginosa* secretes LasA protease, which lyses *S*. *aureus*, and transcription of the *lasA* gene is controlled by LasR [23,29]. Thus, we assumed that the reduction of *S*. *aureus* cells may result from correlation between DesB and production of these factors. However, it should be pointed out that the *desB* mutant retains some reduction ability. This finding could be explained by the presence of DesB-independent factors that participate in *S*. *aureus* reduction even in the absence of DesB activity [14,27,30]. It was discussed more detailedly later in this paper.

### DesB positively regulates the transcription of the MvfR-regulated genes *pqs* and *phn*

**Fig 3:**
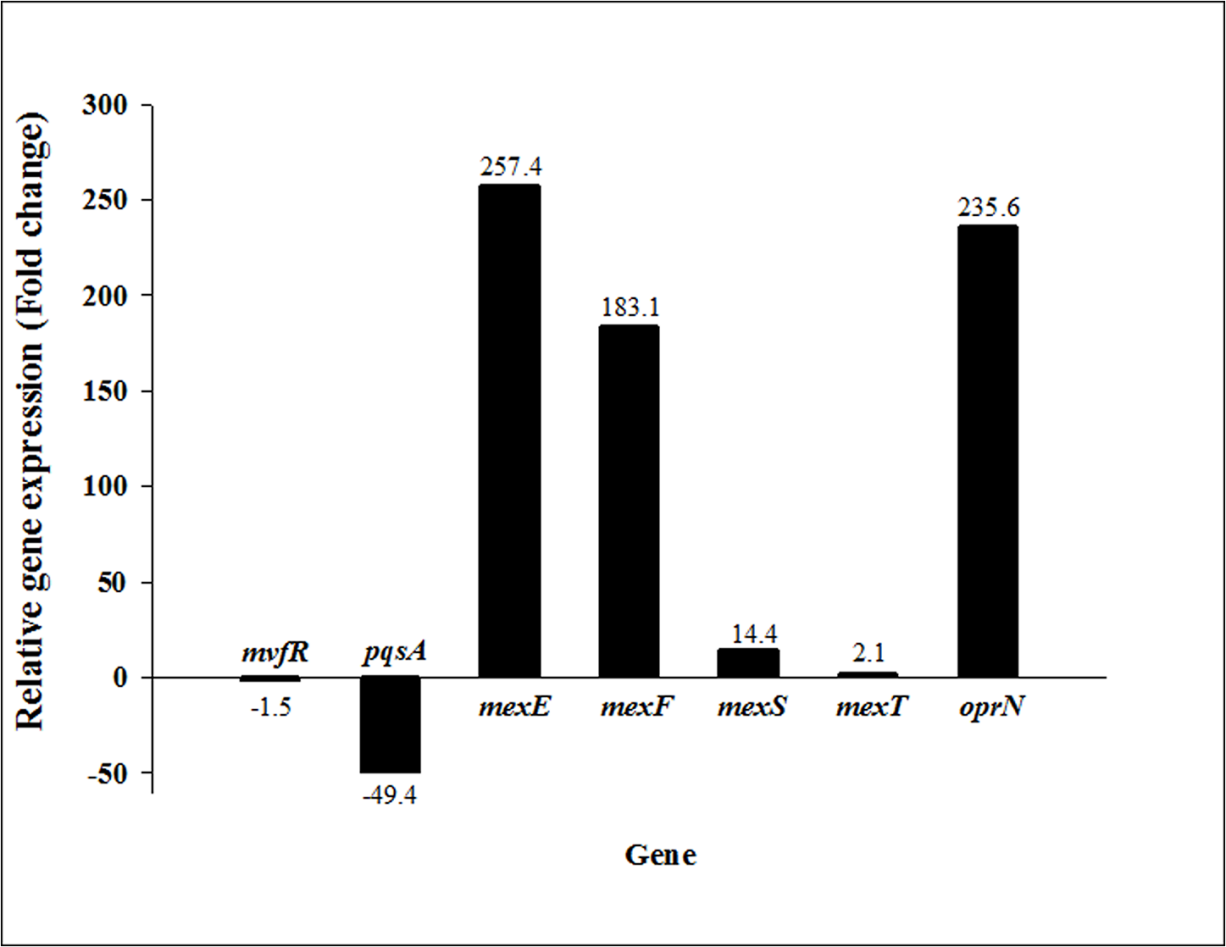
qRT-PCR analysis. Total RNA was extracted from 1 mL of the wild type (WT) and *desB* mutant (*desB*) *P*. *aeruginosa* PAO1 strains grown until an OD_600_ of 0.4–0.5, and then cDNA was synthesized. The relative gene expression of *mvfR*, *pqsA*, *mexE*, *mexF*, *mexT*, and *oprN* in WT and *desB* was compared by qRT-PCR. The results are expressed as the fold-change of the relative gene expression in *desB* compared to that in WT.

qRT-PCR and microarray analyses were conducted to determine the possible molecular mechanism underlying DesB-involved *S*. *aureus* reduction. Mashburn et al. [13] reported that a *P*. *aeruginosa pqsA* mutant exhibited reduced *S*. *aureus* lysis during coculture, indicating that PqsA or anthranilate-coenzyme A ligase [31] is essential for complete *S*. *aureus* lytic activity. Thus, we investigated the possibility of a correlation between *desB* and two other genes, *pqsA* and its regulator *mvfR*. The results showed that in the *desB* mutant, the transcription of *mvfR* was slightly reduced, whereas *pqsA* expression was reduced approximately 50-fold compared to the levels in WT (Fig 3). This result suggested that DesB plays a significant role in *P*. *aeruginosa-*catalyzed *S*. *aureus* reduction in mixed culture by controlling *pqsA* gene expression. PqsA is required for HAQ synthesis, which regulates the production of the cell-to-cell communication factors and virulence factors of *P*. *aeruginosa*, including elastase and pyocyanin [32,33]. However, from this result, we could not predict how DesB regulates *pqsA* expression at the molecular level. Consequently, a microarray analysis was performed to elucidate the molecular mechanism underlying DesB-related *S*. *aureus* reduction. Based on the fact that DesB controls *pqsA*, transcriptional levels of HAQ-related genes including QS genes in WT and *desB* mutant were compared.

The expression patterns of *pqsA* and *mvfR* in the qRT-PCR analysis were also consistent with microarray data (Table 3). The levels of all the *pqs* genes, including *pqsA*, were significantly reduced in the *desB* mutant, whereas the expression levels of two other *S*. *aureus* lysis-related genes, *lasA* (encoding LasA protease) and *lasR* (required for *lasA* transcription) in the *desB* mutant and WT strains were similar.

**Table 3:** Selected microarray analytical data to compare the expression of HAQ*-*related genes in WT and *desB* mutant.

*desB* /WT 1	Name	Product	Locus_tag
***S*. *aureus* lysis**			
1.1	*lasR*	Transcriptional regulator LasR	PA1430
1.0	*lasA*	LasA protease precursor	PA1871
**Anthranilate synthesis**			
-1.4	*trpE*	Anthranilate synthetase component I	PA0609
1.0	*trpG*	Anthranilate synthase component II	PA0649
**-17.5**	***phnA***	**Anthranilate synthase component I**	**PA1001**
**-6.5**	***phnB***	**Anthranilate synthase component II**	**PA1002**
1.0	*kynU*	Hypothetical protein	PA2080
-1.2	*kynB*	Kynurenine formamidase, KynB	PA2081
1.6	*kynA*	Hypothetical protein	PA2579
**QS-regulatory systems**			
-1.1	*vfr*	Transcriptional regulator Vfr	PA0652
1.1	*lasR*	Transcriptional regulator LasR	PA1430
-1.6	*rsaL*	Regulatory protein RsaL	PA1431
1.2	*lasI*	Autoinducer synthesis protein LasI	PA1432
1.5	*qscR*	Quorum-sensing control repressor	PA1898
-1.1	*gacA*	Response regulator GacA	PA2586
**-3.0**	***rhlI***	**Autoinducer synthesis protein RhlI**	**PA3476**
-1.8	*rhlR*	Transcriptional regulator RhlR	PA3477
**-8.8** ^**2**^	***pqsA***	**Probable coenzyme A ligase**	**PA0996**
**-6.9**	***pqsB***	**Homologous to beta-keto-acyl-acyl-carrier**	**PA0997**
**-10.5**	***pqsC***	**Homologous to beta-keto-acyl-acyl-carrier**	**PA0998**
**-6.2**	***pqsD***	**3-oxoacyl-[acyl-carrier-protein] synthase III**	**PA0999**
**-5.8**	***pqsE***	**Quinolone signal response protein**	**PA1000**
**-17.5**	***phnA***	**Anthranilate synthase component I**	**PA1001**
**-6.5**	***phnB***	**Anthranilate synthase component II**	**PA1002**
-1.2	*mvfR*	Transcriptional regulator	PA1003
-1.8	*pqsH*	Probable FAD-dependent monooxygenase	PA2587
**Efflux pump**			
**10.8**	***mexS***	**Probable oxidoreductase**	**PA2491**
1.2	*mexT*	Transcriptional regulator MexT	PA2492
**61.4**	***mexE***	**Resistance-nodulation-cell division (RND)**	**PA2493**
1.6	*mexF*	Resistance-nodulation-cell division (RND)	PA2494
**9.0**	***oprN***	**Multidrug efflux outer membrane protein OprN**	**PA2495**

^1^ Fold change is reported as relative gene expression of *desB* mutant compared to WT (= 1).

^2^ The gene for more than 2-fold change in expression is bold.

Recently, Beaume et al [34] reported that *lasA* is involved in *S*. *aureus* lysis, whereas *pqs* is responsible for *S*. *aureus* growth inhibition rather than lysis. Therefore, this indicates that *desB* mutant have reduced ability in *S*. *aureus* growth inhibition by a considerable decrease in *pqs* expression. Also, microarray analysis revealed that LasA plays a role in *S*. *aureus* lysis as a DesB-independent factor in the absence of functional DesB. In addition, Beaume et al. [34] demonstrated that a *carB* gene, involved in pyrimidine biosynthesis, is required for *S*. *aureus* growth inhibition without any influence on PQS synthesis. Thus, CarB also contributes to *S*. *aureus* growth inhibition in a DesB-independent manner [34].

*P*. *aeruginosa* produces *S*. *aureus* growth inhibition-associated materials, such as 4-hydroxy-2-alkylquinolines (HAQs), which have antimicrobial activity. These HAQs include 4-hydroxy-2-heptylquinoline (HHQ), 4-hydroxy-2-nonylquinoline (HNQ), pseudomonas quinolone signal (PQS), and 4-hydroxy-2-heptylquinoline *N*-oxide (HQNO) [35]. Synthesis of these HAQs is mainly controlled by the *P*. *aeruginosa pqs* system, which comprises *pqsA-E* and *pqsH*. PqsA-D catalyzes the synthesis of HHQ molecules, which are converted to PQS by PqsH. The *pqsA-E* genes are expressed under the control of the transcriptional regulator MvfR, whereas *pqsH* is regulated by LasR, but not by MvfR. Thus, the transcriptional pattern of *pqsA-E* is distinct from that of *pqsH* in the *desB* mutant. The PQS system is interlinked with two quorum-sensing systems, *las* and *rhl* [36]. MvfR, a regulator of the *pqs* system, is positively controlled by LasR and negatively regulated by RhlR [37]. Comparative transcriptional analysis showed that in the *desB* mutant, expression of the *pqs* operon was reduced, whereas the *rhl* and *las* QS genes were normally expressed, indicating that *rhlR* and *lasR* are not involved in the reduced *pqs* expression observed in the *desB* mutant. However, *rhlI* expression was reduced by *desB* mutation compared to the levels in WT. Since, according to McKnight et al. [38], *rhlI* expression is positively regulated by PQS, we could assume that the decreased *rhlI* transcription observed in the *desB* mutant may be attributed to reduced levels of PQS.

In addition, expression of *phnA* and *phnB* in the *desB* mutant was significantly lower than that in WT. In addition to the *pqs* operon, transcription of the *phnAB* genes is also under the control of MvfR. The *phnAB*-encoded proteins are responsible for conversion of shikimic acid to anthranilate, which is continually used in HAQs synthesis catalyzed by PqsA-D. TrpE and TrpG also catalyze anthranilate synthesis from chorismic acid, and the anthranilate produced by this pathway is known to be utilized for either tryptophan or HAQ synthesis [39]. In addition to the PhnAB pathway, there is an alternative anthranilate synthesis pathway, called the kynurenine pathway, which consists of KynA, KynB, and KynU, and catalyzes the conversion of tryptophan to anthranilate. However, dissimilar to *pqs* expression, transcriptional expression of *kynA*, *kynB*, *kynU*, and *trp*EG was not reduced in the *desB* mutant. Consequently, the above results indicate that anthranilate synthesis is controlled by DesB, and that only MvfR-regulated *phnAB* expression is associated with this regulation (Table 3). Although *pqsA-E* and *phnAB* are co-regulated by MvfR [40], only a small difference in *mvfR* expression between the *desB* mutant and WT was observed in the microarray analysis. This phenomenon could be explained by MvfR activation. HHQ functions as a ligand for the LasR-type transcriptional regulator MvfR and enhances MvfR binding to the *pqsA-E* promoter [41], thereby activating the *pqsABCDE* and *phnAB* operons, which is followed by increased production of HAQs. Therefore, in the *desB* mutant, HHQ was not sufficient to stimulate MvfR binding to the promoter, even if *mvfR* was expressed at a similar level to that in the WT strain; thus, resulting in decreased MvfR-governed activation of the downstream *pqs* and *phn* genes in the *desB* mutant and reduced production of HAQs.

In addition, the amounts of crude PQS produced by WT and *desB* mutant were indirectly measured by spot assay for *S*. *aureus* growth inhibition in order to compare PQS levels between two strains. As a result, extract of *desB* mutant exhibited the considerable decrease of *S*. *aureus* growth inhibition compared to the one of WT, meaning that *desB* mutant produced much less PQS than WT (data not shown).

### The *mexEF-oprN* operon is overexpressed in the *desB* mutant

We hypothesized that the low levels of HHQ and concomitant decrease of PQS might lead to the reduced *S*. *aureus* growth inhibition via a yet unknown regulatory mechanism. Interestingly, we showed, both by qRT-PCR and by microarray analyses, that the *mexEF-oprN* operon was highly overexpressed in the *desB* mutant compared to the levels in WT (Table 3, Fig 3). Although the *mexF* expression levels in the microarray analysis appear to differ from the levels in the qRT-PCR analysis due to the relatively lower accuracy of microarray data compared to qRT-PCR, the same tendency for transcriptional expression of *mexEF-oprN* was shown in the results of both methods. The *mexEF*-*oprN* operon in *P*. *aeruginosa* encodes a resistance-nodulation-cell division (RND)-type efflux pump, MexEF-OprN. The MexEF-OprN system is not induced in most *P*. *aeruginosa* strains, thereby allowing the expression of QS-regulated virulence determinants [42]. The *mexEF-oprN* multidrug efflux operon is highly expressed in the presence of antibiotics, nitrosative stress, or disulfide stress [43,44]. Expression of the *mexEF-oprN* operon is known to be positively regulated by MexT, which is encoded by a gene located immediately upstream of the *mexEF-oprN* operon, and the *mexT* gene is negatively regulated by MexS, an oxidoreductase [45,46]. This pump is an important factor for antibiotic resistance, and it transports various molecules, such as chloramphenicol, fluoroquinolones, triclosan, and trimethoprim [47]. Fukuda et al. [48] reported a norfloxacin-resistant mutant of *P*. *aeruginosa* PAO1, called an *nfxC*-type mutant, and showed that an *nfxC*-type mutant overexpresses the MexEF-OprN efflux pump. Kohler et al. [49] reported that the *nfxC-*type mutant shows decreased *rhlI* expression, and the resulting overexpression of the efflux system negatively affects cell-to-cell signaling in *P*. *aeruginosa*. The transcriptional profile of the *desB* mutant is similar to that of the *nfxC*-type mutant in terms of the levels of *pqsA*, *phnAB*, and type III secretion system-related gene expression [50], but different in terms of *lasB* and *rhlAB* expression. The wild-type PAO1 strain used in this study contains a 8-bp insertion in *mexT*, which encodes an inactive and uninducible protein, whereas its isogenic *nfxC* mutant harbors a deletion of the 8-bp insert and produces functional MexT [42]. However, our transcriptional analyses revealed that the *mexEF-oprN* operon was greatly overexpressed in *desB* mutant, even if *mexT* expression was not altered due to suppression via increased *mexS* expression (Table 3, Fig 3). According to Kohler et al. [45], *nfxC*-type mutant produces the effector of MexT, thus causing MexT activation at the posttranslational level and consequently constant overexpression of the *mexEF*-*oprN* operon. In addition, MexT also positively regulates several other genes such as *PA1744*, *PA1970*, *PA2759*, *PA3229*, *PA4623*, and *PA4881* [51], and our transcriptional analysis revealed that these genes were highly expressed in *desB* mutant (data not shown). Hence, we could assume that posttranslationally activated MexT contributes to the overexpression of *mexEF*-*oprN* and other additional genes. Alternatively, the results suggest that *mexEF-oprN* overexpression in the *desB* mutant is MexT independent. Kumar and Schweizer [52] observed that large colony variants lacking several efflux pumps exhibited *mexEX-oprN* overexpression, even though they harbor nonfunctional MexT [52]. This finding suggested that metabolic stress due to the fitness impairment of the variants caused overexpression of the MexEF-OprN efflux pump via a yet uncharacterized regulatory mechanism in the absence of MexT activation [52]. Likewise, our findings could be explained by a disruption of normal metabolism due to the *desB* mutation, which affects cell fitness and facilitates MexEF-OprN overexpression in the absence of MexT-controlled regulation.

**Fig 4:**
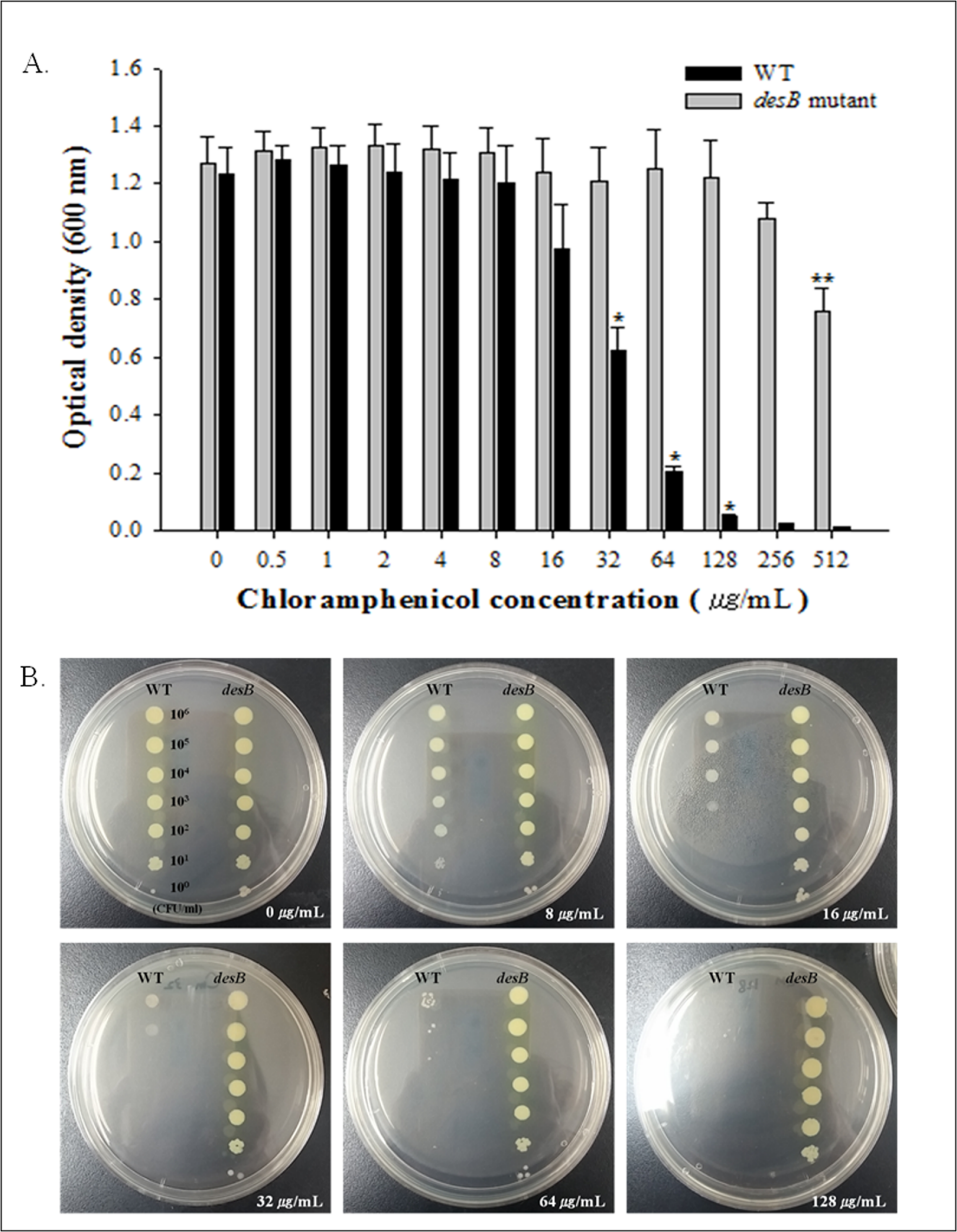
Chloramphenicol resistance of *Pseudomonas aeruginosa* PAO1 wild type (WT) and *desB* mutant (*desB*) strains: MIC (A) and serial dilution-spotting assay (B). (A) WT and *desB* were exposed to chloramphenicol (0–512 μg/mL). After 24-h incubation, the optical density at 600 nm (OD_600_) was measured to determine bacterial growth in the presence of chloramphenicol. (B) Overnight cultures of WT and *desB* were 10-fold serially diluted and then spotted on chloramphenicol-containing agar plates (0, 8, 16, 32, 64, and 128 μg/mL). After incubation, growth was observed. Means with (*, **) are significantly different (*P* < 0.05) in WT or *desB* mutant.

In order to confirm the phenotype of *mexEF*-*oprN* efflux pump overexpression in the *desB* mutant, we determined the MIC of chloramphenicol, a representative substrate for MexEF-OprN and performed serial dilution-spotting assays. The results showed that the *desB* mutant was considerably more resistant to chloramphenicol that the WT, and the MIC of the *desB* mutant was greater than 512 μg/mL, compared to 128 μg/mL for WT (Fig 4A). In the serial dilution-spotting assay, all ten-fold dilutions of the *desB* mutant grew normally up to 128 μg/mL of chloramphenicol, whereas growth of WT was reduced starting at 16 μg/mL (Fig 4B). This result demonstrated that the *desB* mutant is highly resistant to antibiotics due to MexEF-OprN overexpression. Lamarche and Deziel [53] demonstrated that besides antibiotics, the MexEF-OprN efflux pump also exports HHQ, which results in low levels of HHQ and PQS inside the bacterial cells. Likewise, overexpressed MexEF-OprN in the *desB* mutant also led to reduced production of HAQs. In addition, Olivares et al. [50] reported that PQS in *P*. *aeruginosa* overexpressing *mexEF-oprN* was not detected in early stationary phase but PQS was produced in late stationary phase. Thus, it can be explained that reduced *S*. *aureus* growth inhibition in *desB* mutant comes from delayed PQS production (Fig 2).

## Conclusion

**Fig 5:**
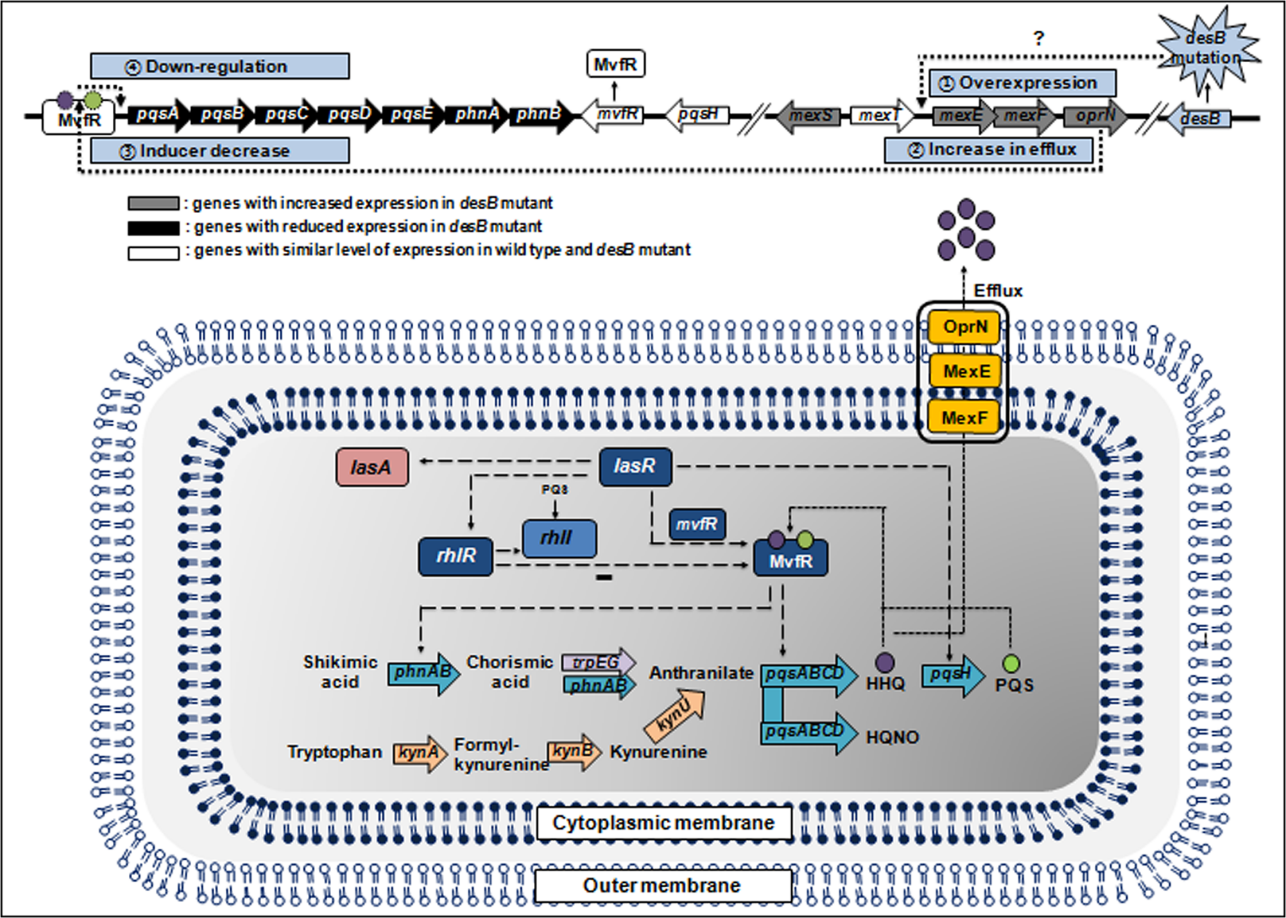
Diagram of the metabolic pathway and possible mechanism related to the effect of *Pseudomonas aeruginosa* DesB on *Staphylococcus aureus* growth inhibition.

This study demonstrated that the *desB* mutation results in overexpression of MexEF-OprN, which subsequently contributes to 1) decreased HAQs levels inside the cells, 2) reduced MvfR binding to the *pqsA-E* promoter, and 3) suppression of HAQ synthesis. Ultimately, these events lead to impaired production of the virulence factors involved in *S*. *aureus* growth inhibition (Fig 5). In other words, *P*. *aeruginosa* DesB is very involved in *S*. *aureus* growth inhibition in mixed microbial communities. However, further studies are needed to determine how *desB* mutation is linked to MexT-independent *mexEF-OprN* expression at the molecular level.

## Supporting Information

S1 Appendix Microarray data comparing gene expression of *P*. *aeruginosa* PAO1 (WT) and its *desB* mutant (*desB* mutant). (PDF)
