# Supplementary material for: Pseudomonas aeruginosa DesB Promotes Staphylococcus aureus Growth Inhibition in Coculture by Controlling the Synthesis of HAQs
Source: PLoS One. 2015 Jul 31;10(7):e0134624. doi: 10.1371/journal.pone.0134624 (PMC4521719; doi:10.1371/journal.pone.0134624)
Supplement: S1 Appendix — (PDF) [file pone.0134624.s001.pdf]

S1 Appendix. Microarray data comparing gene expression of *desB* mutant and Wild Type (WT) *P. aeruginosa* PAO1.

	**Normalized data**	**Raw data**	**Annotation**				
**oligo_id**	***desB* Mutant/WT**	***desB* Mutant/WT**	**name**	**product**	**locus_tag**	**start**	**tm**
1674662	0.932	0.7307351	null	hypothetical protein	PA0012	19	92.05
1674665	1.025	-2.044429	null	hypothetical protein	PA0014	77	93.06
1674667	0.945	0.64382017	null	conserved hypothetical protein	PA0006	63	92.04
1674670	0.898	0.728108	lptA	lysophosphatidic acid acyltransferase, LptA	PA0005	111	94.55
1674673	0.707	0.39659554	null	hypothetical protein	PA0015	108	93.08
1674675	1.083	0.8846668	glyQ	glycyl-tRNA synthetase alpha chain	PA0009	1	93.68
1674678	0.862	0.70868206	gyrB	DNA gyrase subunit B	PA0004	32	95.54
1674681	1.402	1.0493845	tag	DNA-3-methyladenine glycosidase I	PA0010	1	92.8
1674684	0.953	0.69345546	null	probable 2-OH-lauroyltransferase	PA0011	626	93.5
1674687	0.955	0.8240508	null	hypothetical protein	PA0007	27	92.09
1674690	1.039	0.9914293	dnaN	DNA polymerase III, beta chain	PA0002	61	93.01
1674693	0.664	0.48996735	null	conserved hypothetical protein	PA0017	818	94.08
1674696	0.986	0.85232306	null	hypothetical protein	PA0020	2	92.22
1674699	0.774	0.62634856	trkA	potassium uptake protein TrkA	PA0016	663	92.34
1674702	1.144	1.0051725	def	polypeptide deformylase	PA0019	165	92.28
1674705	0.751	0.2640857	qor	quinone oxidoreductase	PA0023	31	95.32
1674708	0.948	0.8500069	aroE	shikimate dehydrogenase	PA0025	3	92.12
1674711	0.830	0.52204186	fmt	methionyl-tRNA formyltransferase	PA0018	62	95.06
1674714	0.933	0.740577	dnaA	chromosomal replication initiator protein DnaA	PA0001	39	92.7
1674717	1.009	-1.371515	null	conserved hypothetical protein	PA0021	223	95.87
1674720	0.903	-3.095532	null	conserved hypothetical protein	PA0013	120	93.86
1674723	1.009	1.8507935	null	hypothetical protein	PA0033	68	93.04
1674726	0.906	0.26181298	null	hypothetical protein	PA0027	532	92.04
1674729	1.438	1.2588356	null	probable two-component response regulator	PA0034	106	95.94
1674732	0.990	0.5383705	null	probable transcriptional regulator	PA0032	144	94.22
1674735	1.009	1.3399541	betC	choline sulfatase	PA0031	112	95.08
1674738	1.597	1.1109747	null	hypothetical protein	PA0038	25	92.01
1674740	1.376	0.6618182	null	probable sulfate transporter	PA0029	72	94.4
1674743	0.727	0.56670934	plcB	phospholipase C, PlcB	PA0026	214	94.86
1674746	1.316	1.1716852	null	hypothetical protein	PA0039	58	92.72
1674748	0.789	0.60503936	null	hypothetical protein	PA0042	37	94.01
1674751	1.040	0.70034903	trpI	transcriptional regulator TrpI	PA0037	151	93.96
1674754	1.009	0.9486928	null	hypothetical protein	PA0030	401	94.24
1674757	0.895	0.89257556	hemF	coproporphyrinogen III oxidase, aerobic	PA0024	307	93.2
1674760	0.821	0.6593613	recF	RecF protein	PA0003	148	92.09
1674763	0.818	0.6658424	null	hypothetical protein	PA0045	1	92.52
1674766	1.587	0.65258896	null	hypothetical protein	PA0047	1	93.22
1674769	0.741	0.5139088	null	conserved hypothetical protein	PA0022	172	92.2
1674772	0.918	0.7857598	null	hypothetical protein	PA0046	285	92.09
1674775	1.188	1.0375464	null	conserved hypothetical protein	PA0040	1	92.89
1674778	0.871	1.3402451	exoT	exoenzyme T	PA0044	16	92.47
1674781	0.699	1.9754188	null	hypothetical protein	PA0052	1	96.53
1674784	1.012	0.8503232	null	hypothetical protein	PA0053	44	92.33
1674786	0.545	0.6041758	trpB	tryptophan synthase beta chain	PA0036	156	93.94
1674789	0.582	0.3109576	null	conserved hypothetical protein	PA0054	18	92.25
1674792	0.440	0.15454826	null	hypothetical protein	PA0055	92	92.74
1674795	0.940	0.77745074	null	hypothetical protein	PA0028	72	96.2
1674798	0.807	0.69925106	null	conserved hypothetical protein	PA0060	3	92.84
1674800	1.009	1.2894788	null	hypothetical protein	PA0057	84	95.29
1674803	0.817	0.6758561	null	hypothetical protein	PA0061	196	92.58
1674805	1.009	1.2626263	phzH	potential phenazine-modifying enzyme	PA0051	177	93.63
1674808	1.122	0.7540703	null	probable transcriptional regulator	PA0056	3	93.02
1674811	1.090	0.88060015	null	hypothetical protein	PA0063	24	97.18
1674814	1.241	0.8668002	osmC	osmotically inducible protein OsmC	PA0059	103	92.51
1674817	0.827	0.71325314	null	hypothetical protein	PA0068	50	92.19
1674819	0.835	3.4859624	null	hypothetical protein	PA0064	44	97.72
1674822	0.849	0.5645336	null	hypothetical protein	PA0065	159	93.43
1674825	1.110	0.7285837	glyS	glycyl-tRNA synthetase beta chain	PA0008	73	93.36
1674828	1.103	0.9615396	null	hypothetical protein	PA0070	115	95.24
1674831	1.052	0.13989	null	conserved hypothetical protein	PA0066	245	94.98
1674834	0.955	-0.9630952	null	probable transcriptional regulator	PA0048	65	93.91
1674837	0.752	0.291655	null	hypothetical protein	PA0072	119	96.27
1674840	1.007	0.74874675	null	probable phosphoprotein phosphatase	PA0075	2	92.05
1674843	0.764	0.655513	null	probable ATP-binding component of ABC	PA0073	301	93.95
1674846	1.274	1.0444212	ppkA	serine/threonine protein kinase PpkA	PA0074	8	92.15
1674849	1.145	0.9435423	null	hypothetical protein	PA0071	881	94.35
1674852	0.817	0.61965907	null	hypothetical protein	PA0077	129	92.42
1674855	1.100	1.0751078	prlC	oligopeptidase A	PA0067	25	93.7
1674858	0.728	0.4951245	null	conserved hypothetical protein	PA0069	204	92.56
1674863	0.524	0.27559906	null	hypothetical protein	PA0080	378	95.72
1674864	0.780	0.4812037	null	hypothetical protein	PA0043	48	97.13
1674867	0.783	0.6751469	null	hypothetical protein	PA0082	1	93.6
1674870	1.126	0.9105385	trpA	tryptophan synthase alpha chain	PA0035	75	93.27
1674873	0.878	0.82585204	null	conserved hypothetical protein	PA0085	182	94.31
1674876	0.861	0.71489793	null	conserved hypothetical protein	PA0083	47	94.54
1674879	0.722	-0.3531955	null	hypothetical protein	PA0062	120	96.34
1674882	0.649	0.5221622	null	hypothetical protein	PA0087	138	92.95
1674884	0.719	0.50579256	null	hypothetical protein	PA0089	341	93.67
1674887	0.988	0.737563	null	hypothetical protein	PA0081	42	92
1674890	0.547	0.3807984	null	hypothetical protein	PA0078	489	93.13
1674893	1.011	0.6881051	null	hypothetical protein	PA0076	613	95.04
1674895	0.518	0.2991473	null	hypothetical protein	PA0086	26	95.86
1674898	0.987	0.7673646	null	hypothetical protein	PA0092	128	96.83
1674900	1.256	0.724972	null	hypothetical protein	PA0058	156	95.93
1674903	1.307	0.88239366	null	hypothetical protein	PA0094	9	92.4
1674906	0.537	0.4019557	null	hypothetical protein	PA0093	172	92.69
1674909	0.846	0.054371815	null	hypothetical protein	PA0100	32	94.92
1674912	1.369	0.64077723	null	hypothetical protein	PA0101	2	92.37
1674915	1.136	0.7948085	null	hypothetical protein	PA0049	1	93.3
1674918	1.175	0.9552248	null	hypothetical protein	PA0098	341	94.14
1674921	0.435	1.7432768	null	hypothetical protein	PA0097	285	93.83
1674924	0.980	0.6965378	null	probable carbonic anhydrase	PA0102	2	92.16
1674927	0.650	0.3176514	null	hypothetical protein	PA0096	314	94.11
1674928	1.158	0.9646203	null	probable hemagglutinin	PA0041	1603	95.46
1674931	1.009	0.65792274	coxB	cytochrome c oxidase, subunit II	PA0105	68	97.69
1674934	1.635	1.0986711	null	hypothetical protein	PA0109	72	93.15
1674935	0.915	0.53752005	null	hypothetical protein	PA0099	46	95.2
1674938	1.509	1.2144442	null	hypothetical protein	PA0110	5	95.62
1674941	0.757	-0.3778405	null	hypothetical protein	PA0104	58	93.92
1674944	1.221	4.033913	null	conserved hypothetical protein	PA0107	428	93.52
1674947	1.028	1.2737023	null	probable cytochrome c oxidase assembly factor	PA0113	39	92.63
1674950	1.009	0.3938261	null	probable sulfate transporter	PA0103	3	92.44

1674953	0.645	0.26047713	null	hypothetical protein	PA0079	30	94.39
1674956	1.134	0.82485944	null	probable ClpA/B-type chaperone	PA0090	69	92.83
1674959	0.739	4.6107144	null	conserved hypothetical protein	PA0115	72	92.47
1674962	1.218	0.94013005	null	hypothetical protein	PA0116	186	94.75
1674965	2.077	1.793966	null	hypothetical protein	PA0111	244	94.71
1674968	1.077	0.6362974	null	probable short chain dehydrogenase	PA0117	265	93.83
1674971	0.976	0.70605993	null	hypothetical protein	PA0118	20	93.32
1674974	0.966	0.78794545	null	probable transcriptional regulator	PA0120	436	93.38
1674977	0.585	0.4345355	null	conserved hypothetical protein	PA0122	216	93.02
1674980	1.065	0.9573746	null	hypothetical protein	PA0125	94	96.4
1674983	1.215	0.8582128	null	hypothetical protein	PA0121	39	92.06
1674986	0.588	0.39592183	null	probable transcriptional regulator	PA0123	300	95.15
1674989	1.388	1.2039547	null	hypothetical protein	PA0126	131	94.99
1674992	1.144	0.9862615	null	hypothetical protein	PA0124	102	92.51
1674994	0.961	0.88094485	null	conserved hypothetical protein	PA0128	6	92.37
1674997	0.955	0.568551	null	hypothetical protein	PA0131	29	94.74
1674999	1.272	1.4256566	coxA	cytochrome c oxidase, subunit I	PA0106	201	92.57
1675002	0.938	0.54610085	null	hypothetical protein	PA0127	124	94.7
1675005	0.789	0.68027276	null	conserved hypothetical protein	PA0095	113	92.02
1675008	0.669	-0.094526984	gabP	gamma-aminobutyrate permease	PA0129	57	95.35
1675011	1.009	1.2820148	null	hypothetical protein	PA0135	10	93.93
1675012	1.173	0.6753551	null	probable aldehyde dehydrogenase	PA0130	228	92.82
1675015	0.989	0.72381675	null	probable dicarboxylate transporter	PA0119	404	93.72
1675018	1.266	0.71959496	null	probable permease of ABC transporter	PA0137	503	94.77
1675021	1.364	1.3278224	ahpC	alkyl hydroperoxide reductase subunit C	PA0139	81	92.76
1675024	1.075	2.3404295	null	beta-alanine--pyruvate transaminase	PA0132	948	95.3
1675027	1.354	1.0256879	null	conserved hypothetical protein	PA0141	264	94.35
1675030	0.785	0.59162176	null	hypothetical protein	PA0142	177	92.67
1675033	1.186	0.85306823	null	hypothetical protein	PA0144	111	94.44
1675036	1.086	0.7978322	ahpF	alkyl hydroperoxide reductase subunit F	PA0140	51	93.02
1675039	1.163	1.3204104	null	probable permease of ABC transporter	PA0138	817	96.37
1675042	1.215	1.0282848	null	hypothetical protein	PA0145	388	93.52
1675045	1.121	0.83674544	null	conserved hypothetical protein	PA0146	1	95.99
1675048	2.223	0.6993464	null	probable guanine deaminase	PA0134	35	95.2
1675051	0.677	0.57552874	null	conserved hypothetical protein	PA0084	238	92.28
1675054	0.894	0.72543824	nuh	nonspecific ribonucleoside hydrolase	PA0143	1	94.98
1675057	0.965	0.4764164	null	probable transcriptional regulator	PA0133	483	94.43
1675060	0.969	0.8543557	null	probable sigma-70 factor, ECF subfamily	PA0149	361	94.17
1675062	0.587	0.3015074	null	probable transmembrane sensor	PA0150	1	96.21
1675065	0.441	-0.29577923	pcaH	protocatechuate 3,4-dioxygenase, beta subunit	PA0153	9	92.98
1675068	0.923	0.5166778	pcaR	transcriptional regulator PcaR	PA0155	65	92.78
1675071	1.040	1.0359805	pcaG	protocatechuate 3,4-dioxygenase, alpha subunit	PA0154	15	94.43
1675074	1.914	0.045847964	null	probable ATP-binding component of ABC	PA0136	30	95.06
1675077	1.541	0.27523813	null	probable adenosine deaminase	PA0148	2	93.37
1675080	2.068	1.4207598	null	probable oxidoreductase	PA0147	509	96.52
1675083	0.899	0.41971284	null	hypothetical protein	PA0160	98	94.77
1675084	0.742	1.6611391	null	probable Resistance-Nodulation-Cell Division	PA0156	181	94.45
1675087	1.074	1.9132512	null	hypothetical protein	PA0112	105	97.52
1675090	1.147	0.9412325	null	conserved hypothetical protein	PA0114	192	93.87
1675093	1.042	1.125993	null	probable TonB-dependent receptor	PA0151	6	97.22
1675096	0.202	0.11854134	null	hypothetical protein	PA0165	55	94.02
1675099	1.247	1.2482351	null	probable Resistance-Nodulation-Cell Division	PA0158	65	92.89
1675102	0.694	0.3086841	pcaQ	transcriptional regulator PcaQ	PA0152	387	92.01
1675105	0.483	0.24898496	null	probable porin	PA0162	312	93.85
1675108	0.980	0.5282991	null	probable transcriptional regulator	PA0163	1	93.83
1675111	0.893	0.7306911	null	hypothetical protein	PA0170	1	94.82
1675114	1.133	0.44184095	null	hypothetical protein	PA0169	14	95.71
1675117	1.047	2.034936	null	probable Resistance-Nodulation-Cell Division	PA0157	437	97.67
1675120	1.009	0.0719246	null	conserved hypothetical protein	PA0174	246	93.52
1675123	0.966	0.7503234	null	probable gamma-glutamyltranspeptidase	PA0164	9	96.68
1675126	0.510	1.1297619	null	probable chemotaxis protein methyltransferase	PA0175	23	92.41
1675129	0.923	0.4654762	null	probable purine-binding chemotaxis protein	PA0177	220	92.25
1675132	0.947	0.68313503	null	probable two-component response regulator	PA0179	21	92.57
1675135	0.564	0.23809776	null	hypothetical protein	PA0171	2	92.93
1675138	0.808	0.6406058	null	probable chemotaxis transducer	PA0180	1	94.71
1675141	2.185	1.2469579	null	probable transcriptional regulator	PA0159	181	96.51
1675145	0.858	0.7153772	null	conserved hypothetical protein	PA0168	223	96
1675147	1.394	0.7365349	null	probable methylesterase	PA0173	166	92.02
1675150	1.043	0.9613274	null	probable transcriptional regulator	PA0181	478	97.68
1675153	2.301	0.36190477	atsA	arylsulfatase	PA0183	238	92.96
1675156	1.000	1.4875677	null	hypothetical protein	PA0187	311	94.35
1675159	1.009	1.9733334	null	hypothetical protein	PA0188	274	93.84
1675162	0.377	-1.7100093	null	hypothetical protein	PA0172	85	95.8
1675165	1.164	0.8983291	null	probable two-component sensor	PA0178	132	93.4
1675168	1.258	3.548718	null	probable porin	PA0189	723	94.8
1675171	1.389	0.9497526	null	probable short-chain dehydrogenase	PA0182	1	92.26
1675174	1.335	0.4997076	null	probable binding protein component of ABC	PA0186	158	92.96
1675177	0.899	0.510872	null	probable acid phosphatase	PA0190	27	92.84
1675180	1.042	1.6885716	null	hypothetical protein	PA0193	133	93.64
1675183	0.106	-0.12938848	null	probable transcriptional regulator	PA0191	121	93.06
1675186	0.893	0.2646447	aer2	aerotaxis transducer Aer2	PA0176	683	93.36
1675189	1.907	1.2315758	pntAB	putative NDA(P) transhydrogenase subunit alpha	PA0195.1	29	92.05
1675191	1.734	1.3373997	null	hypothetical protein	PA0194	183	93.55
1675194	1.009	0.2773077	exbD1	transport protein ExbD	PA0199	159	95.94
1675197	1.009	0.06625152	null	hypothetical protein	PA0197	175	93.52
1675200	1.143	1.089814	null	hypothetical protein	PA0200	137	94.18
1675202	1.280	0.98180276	exbB1	transport protein ExbB	PA0198	374	94.56
1675205	1.124	0.8803868	pntB	pyridine nucleotide transhydrogenase, beta	PA0196	12	92.26
1675208	0.767	2.068653	pntA	putative NDA(P) transhydrogenase subunit alpha	PA0195	48	94.98
1675211	0.447	0.25263184	null	hypothetical protein	PA0201	2	92.96
1675214	1.009	0.17055306	null	probable permease of ABC transporter	PA0204	1	94.01
1675217	1.482	1.0060937	null	probable permease of ABC transporter	PA0205	168	94.28
1675220	1.009	1.14	null	probable TonB-dependent receptor	PA0192	202	94.09
1675223	0.726	0.5242479	null	probable transcriptional regulator	PA0167	378	95.05
1675226	0.932	0.6773692	null	probable transcriptional regulator	PA0207	153	93.93
1675229	1.129	0.21358533	mdcC	malonate decarboxylase delta subunit	PA0210	208	97.62
1675230	1.090	0.8308169	null	probable permease of ABC transporter	PA0185	151	96.55
1675233	1.076	2.4488888	null	conserved hypothetical protein	PA0209	69	95.93
1675236	1.030	0.8042649	mdcE	malonate decarboxylase gamma subunit	PA0212	114	96.69
1675239	1.009	0.56095237	null	hypothetical protein	PA0213	40	97.5
1675242	1.157	0.6464035	null	probable transporter	PA0215	185	93.11
1675245	0.681	-0.78347766	null	probable transporter	PA0216	178	95.95
1675248	1.009	3.2075038	null	probable binding protein component of ABC	PA0203	456	92.36
1675251	0.777	0.48619762	null	probable ATP-binding component of ABC	PA0184	792	93.52
1675254	0.847	-0.78377306	null	probable transcriptional regulator	PA0218	62	96.43
1675257	1.111	0.53618115	null	probable transcriptional regulator	PA0217	142	96.5
1675260	1.100	1.2089286	null	probable amidase	PA0202	234	92.97
1675263	1.057	0.3262319	null	probable dihydrodipicolinate synthetase	PA0223	170	93.16
1675266	1.009	-6.550476	null	probable aldolase	PA0224	64	92.8

1675269	1.188	1.1564847	mdcA	malonate decarboxylase alpha subunit	PA0208	168	93.07
1675272	1.067	0.8495358	null	probable transcriptional regulator	PA0225	134	93.72
1675275	0.793	0.62523335	null	probable transporter	PA0166	60	97.47
1675278	0.948	-0.45090908	null	probable amino acid permease	PA0220	204	95.46
1675281	1.397	1.1114105	null	hypothetical protein	PA0222	14	93.06
1675284	0.947	0.70754594	null	probable CoA transferase, subunit A	PA0226	133	92.55
1675287	0.660	0.17257482	pcaD	beta-ketoadipate enol-lactone hydrolase	PA0231	95	97.17
1675290	0.978	-0.5342309	null	probable CoA transferase, subunit B	PA0227	110	94.76
1675293	1.076	4.3008337	null	probable transcriptional regulator	PA0233	217	94.68
1675296	1.228	0.81480294	pcaT	dicarboxylic acid transporter PcaT	PA0229	60	93.06
1675299	1.075	1.3438385	null	probable aminotransferase	PA0221	328	93.52
1675302	0.820	0.25186208	null	hypothetical protein	PA0234	65	92.16
1675305	0.930	0.6063889	pcaC	gamma-carboxymuconolactone decarboxylase	PA0232	90	92.28
1675308	2.073	0.6810299	null	probable ATP-binding component of ABC	PA0206	1	93.11
1675311	1.265	0.9421792	null	probable transcriptional regulator	PA0236	24	97.86
1675314	1.275	0.55169404	null	hypothetical protein	PA0239	75	92.02
1675317	0.610	0.27681008	null	probable oxidoreductase	PA0237	130	96.08
1675320	0.743	0.041911203	null	hypothetical protein	PA0088	255	97.78
1675323	0.887	0.31948715	pcaF	beta-ketoadipyl CoA thiolase PcaF	PA0228	581	96.52
1675326	1.493	1.2019614	null	probable transcriptional regulator	PA0243	1	95.66
1675329	1.009	0.93337107	mdcD	malonate decarboxylase beta subunit	PA0211	535	96.51
1675332	0.928	-1.2609525	null	hypothetical protein	PA0238	387	96.34
1675335	0.739	1.0688492	null	hypothetical protein	PA0242	550	92.88
1675338	0.810	0.5246633	null	hypothetical protein	PA0244	92	92.97
1675341	1.446	0.5456569	pobA	p-hydroxybenzoate hydroxylase	PA0247	467	96.12
1675344	1.074	2.897841	null	hypothetical protein	PA0251	19	95.52
1675347	0.930	0.70877963	null	conserved hypothetical protein	PA0250	260	92.4
1675350	0.294	0.052413415	null	hypothetical protein	PA0252	4	95.71
1675353	1.356	-2.7063773	pcaK	4-hydroxybenzoate transporter PcaK	PA0235	318	93.79
1675356	0.728	1.097255	null	probable aldehyde dehydrogenase	PA0219	365	93.5
1675359	1.009	-0.77550256	null	probable major facilitator superfamily (MFS)	PA0241	163	93.72
1675362	0.986	0.79989713	null	probable acetyltransferase	PA0249	1	95.87
1675365	1.176	1.0149462	null	probable transcriptional regulator	PA0253	115	92.99
1675368	0.610	2.3481426	null	hypothetical protein	PA0256	47	94.41
1675371	2.385	-0.15956709	null	probable porin	PA0240	73	96.4
1675374	0.836	0.67181253	null	conserved hypothetical protein	PA0255	119	94.4
1675377	1.120	0.8060322	null	conserved hypothetical protein	PA0254	389	95.75
1675380	0.767	0.72626877	null	hypothetical protein	PA0261	107	94.16
1675383	0.803	0.3868299	null	hypothetical protein	PA0264	197	92.14
1675386	0.960	0.7843109	null	probable major facilitator superfamily (MFS)	PA0246	1	96.57
1675389	0.616	0.11294623	null	hypothetical protein	PA0257	167	92.45
1675392	1.138	0.9822456	null	hypothetical protein	PA0259	74	93.43
1675395	0.910	0.75501025	gabD	succinate-semialdehyde dehydrogenase	PA0265	215	93.17
1675398	0.621	0.16205533	null	hypothetical protein	PA0267	21	95.27
1675401	1.082	0.78027326	null	hypothetical protein	PA0260	11	92.81
1675406	0.817	0.5837115	hcpC	secreted protein Hcp	PA0263	402	92.55
1675407	1.009	2.2858858	null	probable transcriptional regulator	PA0272	82	92.89
1675410	1.201	0.98185825	null	conserved hypothetical protein	PA0269	86	93.81
1675413	0.562	-1.7940013	null	hypothetical protein	PA0271	4	93.87
1675416	1.212	0.9447997	null	probable transcriptional regulator	PA0248	47	97.18
1675419	0.993	0.6880008	null	hypothetical protein	PA0270	111	92.08
1675422	0.692	0.49103934	null	conserved hypothetical protein	PA0277	509	96.09
1675425	1.091	0.9638525	gabT	4-aminobutyrate aminotransferase	PA0266	231	93.21
1675429	1.021	0.72666943	null	probable transcriptional regulator	PA0275	148	94.77
1675431	1.258	0.15000002	null	probable transcriptional regulator	PA0268	127	93.78
1675434	0.830	0.5658757	null	probable transcriptional regulator	PA0279	375	94.54
1675437	0.763	0.106862746	null	hypothetical protein	PA0276	5	92.54
1675440	0.351	0.30381584	null	hypothetical protein	PA0284	24	92
1675441	0.155	0.056756884	cysT	sulfate transport protein CysT	PA0282	261	92.04
1675444	0.117	1.1364679	cysA	sulfate transport protein CysA	PA0280	48	94.52
1675447	1.244	1.0871203	null	probable fatty acid desaturase	PA0286	3	92.28
1675450	0.914	0.23262644	null	probable major facilitator superfamily (MFS)	PA0273	392	96.84
1675453	1.148	1.0340381	gpuA	3-guanidinopropionase	PA0288	313	94.46
1675456	1.009	-1.7371428	gpuP	3-guanidinopropionate transport protein	PA0287	49	94.97
1675459	0.163	0.10280943	cysW	sulfate transport protein CysW	PA0281	387	94.54
1675462	0.660	0.23155367	aguB	N-carbamoylputrescine amidohydrolase	PA0293	95	93.82
1675465	1.327	2.2082558	null	hypothetical protein	PA0290	159	92.09
1675468	0.258	0.20303647	sbp	sulfate-binding protein precursor	PA0283	416	93.25
1675471	1.024	0.51775163	gpuR	transcriptional activator GpuR	PA0289	657	94.62
1675474	1.005	0.9187592	oprE	Anaerobically-induced outer membrane porin OprE	PA0291	511	93.46
1675477	0.674	0.08880615	aguR	transcriptional regulator AguR	PA0294	108	93.12
1675480	1.034	2.2706242	aroQ2	3-dehydroquinate dehydratase	PA0245	1	93.41
1675483	1.033	-2.4555733	null	hypothetical protein	PA0274	703	97.67
1675486	0.415	-0.15923421	spuA	probable glutamine amidotransferase	PA0297	200	94.19
1675489	0.955	0.70269907	null	probable periplasmic polyamine binding protein	PA0295	288	92.62
1675492	0.944	2.7445612	null	conserved hypothetical protein	PA0285	92	93.25
1675495	0.331	-0.21268281	pcaB	3-carboxy-cis,cis-muconate cycloisomerase	PA0230	378	93.94
1675498	0.910	0.50582784	aguA	agmatine deiminase	PA0292	296	93.13
1675501	1.282	0.8374907	null	conserved hypothetical protein	PA0262	2315	95.8
1675504	0.932	0.67175525	spuB	probable glutamine synthetase	PA0298	216	94.45
1675507	1.041	0.8351976	spuG	polyamine transport protein PotH	PA0303	109	92.99
1675510	0.973	0.79208577	null	hypothetical protein	PA0309	6	94.27
1675513	0.905	0.37139216	null	hypothetical protein	PA0308	1	94.31
1675516	1.093	0.5526515	null	hypothetical protein	PA0310	87	92.85
1675519	12.123	0.79379505	null	hypothetical protein	PA0307	3	92.7
1675522	1.438	1.467912	coIII	cytochrome c oxidase, subunit III	PA0108	2	92.87
1675525	0.787	0.64009905	null	conserved hypothetical protein	PA0312	227	95.89
1675528	1.009	-3.621212	null	hypothetical protein	PA0311	74	94.7
1675531	1.019	0.7822391	spuH	polyamine transport protein PotI	PA0304	389	93.11
1675534	0.671	-1.1442304	null	probable transcriptional regulator	PA0306	357	95.37
1675537	1.009	0.890175	null	probable glutamine synthetase	PA0296	10	92.6
1675540	1.009	0.83022624	null	probable permease of ABC transporter	PA0313	316	95.34
1675543	1.043	0.8625868	spuF	polyamine transport protein PotG	PA0302	399	93.12
1675546	1.570	1.4652739	spuC	putrescine aminotransferase	PA0299	64	92.84
1675550	0.919	0.78548336	null	hypothetical protein	PA0315	270	96.05
1675552	1.369	0.9241744	spuE	polyamine transport protein	PA0301	110	93
1675555	1.113	1.0252903	serA	D-3-phosphoglycerate dehydrogenase	PA0316	156	94.87
1675558	0.909	0.7395471	null	hypothetical protein	PA0319	10	92.3
1675561	0.944	0.58198655	null	conserved hypothetical protein	PA0320	162	92.43
1675564	7.422	4.293871	null	probable binding protein component of ABC	PA0323	74	94.27
1675567	0.872	0.535132	null	probable permease of ABC transporter	PA0325	99	92.83
1675570	0.804	0.46795836	null	conserved hypothetical protein	PA0318	73	92.16
1675573	1.003	0.8771698	null	conserved hypothetical protein	PA0329	177	93.72
1675575	0.129	1.2059004	null	conserved hypothetical protein	PA0091	1625	95.57
1675578	0.977	0.6298428	null	hypothetical protein	PA0327	9	92.22
1675581	0.759	0.38810605	null	probable acetylpolyamine aminohydrolase	PA0321	175	94.22
1675584	1.130	0.9238163	spuD	polyamine transport protein	PA0300	33	97.86
1675587	1.188	0.95325434	null	hypothetical protein	PA0328	10	92.58
1675590	0.997	0.8581545	null	hypothetical protein	PA0317	7	94.83

1675593	1.233	0.9889658	rpiA	ribose 5-phosphate isomerase	PA0330	36	93.76
1675598	0.865	0.747357	ygdP	Nudix hydrolase YgdP	PA0336	330	94.81
1675599	0.541	-0.25209746	null	hypothetical protein	PA0278	145	92.46
1675602	0.881	0.641137	ilvA1	threonine dehydratase, biosynthetic	PA0331	391	92.26
1675605	0.701	0.59571993	null	hypothetical protein	PA0338	13	92.34
1675608	0.410	-0.50927246	null	hypothetical protein	PA0339	196	95.6
1675611	1.215	0.86535054	lgt	prolipoprotein diacylglyceryl transferase	PA0341	110	95.64
1675614	1.009	1.5166566	null	probable ATP-binding component of ABC	PA0326	887	92.25
1675617	1.343	0.9901574	null	probable transporter	PA0322	97	92.5
1675620	0.751	-1.4777778	null	hypothetical protein	PA0343	3	92.73
1675623	0.711	0.4991444	thyA	thymidylate synthase	PA0342	67	93.91
1675626	1.009	-0.32926828	null	hypothetical protein	PA0346	185	94.12
1675629	0.726	0.5470909	null	hypothetical protein	PA0333	1	94.77
1675632	1.097	0.95193446	null	probable permease of ABC transporter	PA0324	48	92.21
1675635	1.239	1.0759208	null	hypothetical protein	PA0332	255	94.48
1675636	0.447	-0.38803437	null	hypothetical protein	PA0344	1130	97.79
1675639	1.009	1.2018065	glpQ	glycerophosphoryl diester phosphodiesterase,	PA0347	351	93.32
1675642	1.088	0.9358243	null	hypothetical protein	PA0348	1	95.42
1675645	1.259	0.96752864	null	probable major facilitator superfamily (MFS)	PA0334	199	94.87
1675648	0.869	0.6282861	folA	dihydrofolate reductase	PA0350	126	96.63
1675651	0.578	-3.7634132	null	hypothetical protein	PA0356	1	94.06
1675654	0.762	0.46898833	null	conserved hypothetical protein	PA0354	166	93.63
1675657	1.064	0.8584639	ilvD	dihydroxy-acid dehydratase	PA0353	1	93.18
1675660	0.925	0.20203122	null	hypothetical protein	PA0349	367	92.49
1675663	0.899	0.80229825	ptsP	phosphoenolpyruvate-protein phosphotransferase	PA0337	64	93.73
1675666	0.973	0.80580187	null	hypothetical protein	PA0360	13	93.39
1675669	1.114	1.0191368	null	hypothetical protein	PA0359	278	96.58
1675672	0.692	0.50550234	null	hypothetical protein	PA0358	45	93.87
1675675	0.379	0.20366065	mutM	formamidopyrimidine-DNA glycosylase	PA0357	243	97.62
1675678	1.015	0.82045877	fdx1	ferredoxin (4Fe-4S)	PA0362	126	96.1
1675681	0.981	0.7367496	null	hypothetical protein	PA0345	821	97.29
1675683	0.963	0.76218516	coaD	phosphopantetheine adenylyltransferase	PA0363	5	92.25
1675686	0.965	0.69847476	null	conserved hypothetical protein	PA0340	306	92.93
1675689	0.726	0.5140945	null	hypothetical protein	PA0369	182	97.3
1675691	1.194	0.878115	pfpI	protease PfpI	PA0355	375	92.13
1675694	1.240	1.0219809	null	probable oxidoreductase	PA0364	501	93.41
1675697	0.551	0.058895133	null	conserved hypothetical protein	PA0368	28	97.57
1675700	0.370	0.74325645	null	hypothetical protein	PA0365	209	96.13
1675703	0.470	-0.22624978	null	probable transporter	PA0352	33	96.73
1675706	1.024	0.7685014	ftsY	signal recognition particle receptor FtsY	PA0373	99	97.66
1675709	1.185	0.8644551	null	hypothetical protein	PA0371	119	93.63
1675712	1.121	0.9503425	ftsE	cell division protein FtsE	PA0374	1	93.44
1675715	0.907	0.56257576	null	probable aldehyde dehydrogenase	PA0366	24	92.84
1675718	0.898	0.7319313	null	conserved hypothetical protein	PA0370	376	93.4
1675721	0.752	0.53132766	ftsX	cell division protein FtsX	PA0375	34	96.57
1675724	0.743	0.60493284	null	hypothetical protein	PA0377	348	96.19
1675727	1.345	1.0143933	null	probable transcriptional regulator	PA0367	217	92.47
1675731	0.598	0.06502825	null	conserved hypothetical protein	PA0380	1	93.84
1675732	1.062	0.7635692	null	probable transglycosylase	PA0378	365	94.81
1675735	17.690	2.6165986	null	hypothetical protein	PA0384	156	92.54
1675738	1.829	0.66975486	null	hypothetical protein	PA0305	274	92.39
1675741	0.685	0.5580175	micA	DNA mismatch repair protein MicA	PA0382	141	95.02
1675744	0.903	0.0901343	null	probable gamma-glutamyltranspeptidase precursor	PA0361	344	94.98
1675747	0.744	-0.19087692	null	conserved hypothetical protein	PA0379	108	92.59
1675749	0.799	0.6665532	null	conserved hypothetical protein	PA0387	389	97.23
1675752	0.837	0.7325867	null	probable oxidase	PA0386	1	97.19
1675755	1.334	0.79624856	null	conserved hypothetical protein	PA0383	587	92.11
1675758	0.726	0.5423502	null	hypothetical protein	PA0389	124	92.63
1675761	0.996	1.1497437	null	conserved hypothetical protein	PA0351	313	92.98
1675764	0.879	0.7077803	null	hypothetical protein	PA0385	268	92.77
1675767	0.907	0.7176004	metX	homoserine O-acetyltransferase	PA0390	89	92.72
1675770	0.929	0.8049877	null	conserved hypothetical protein	PA0394	207	94.74
1675773	0.945	0.7000486	null	probable zinc protease	PA0372	317	92.98
1675776	0.842	0.69356275	null	conserved hypothetical protein	PA0392	345	92.22
1675779	0.909	0.748008	proC	pyrroline-5-carboxylate reductase	PA0393	714	93.22
1675782	0.927	0.7122425	pilT	twitching motility protein PilT	PA0395	192	93.08
1675785	0.923	0.7512921	null	hypothetical protein	PA0398	143	92
1675788	0.942	0.7843641	null	cystathionine beta-synthase	PA0399	60	94.39
1675791	0.883	1.0294396	null	hypothetical protein	PA0335	197	95.12
1675794	1.175	1.0680947	pyrB	aspartate carbamoyltransferase	PA0402	169	92.57
1675797	0.848	0.40692157	null	probable cation efflux system protein	PA0397	1	95.2
1675800	0.847	0.7358211	rpoH	sigma factor RpoH	PA0376	315	92.61
1675803	1.162	1.0138825	null	conserved hypothetical protein	PA0405	242	97.45
1675806	0.812	0.6890131	pilU	twitching motility protein PilU	PA0396	173	94.09
1675809	0.812	0.672562	null	conserved hypothetical protein	PA0404	130	93.13
1675811	0.814	0.6078847	gshB	glutathione synthetase	PA0407	27	93.65
1675814	0.943	0.85495394	pilG	twitching motility protein PilG	PA0408	204	92.16
1675816	1.057	0.94567716	pyrR	transcriptional regulator PyrR	PA0403	426	92.78
1675819	0.601	0.5198812	pilI	twitching motility protein PilI	PA0410	154	95.67
1675822	0.748	0.6591694	pilK	methyltransferase PilK	PA0412	152	93.19
1675825	0.753	0.5772327	chpD	probable transcriptional regulator	PA0416	108	92.51
1675828	0.730	0.49653107	null	noncatalytic dihydroorotase-like protein	PA0401	193	96.01
1675831	0.915	0.57737195	null	hypothetical protein	PA0391	179	92.25
1675834	1.255	0.9425599	null	probable binding protein component of ABC	PA0314	192	92.74
1675837	0.944	0.76798224	chpB	probable methylesterase	PA0414	593	94.2
1675840	0.883	0.7211382	chpA	still frameshift probable component of	PA0413	105	95.81
1675843	1.043	0.85858595	chpC	probable chemotaxis protein	PA0415	1	92.81
1675846	0.656	0.53169364	null	hypothetical protein	PA0418	12	96.26
1675849	0.827	0.92749375	null	conserved hypothetical protein	PA0419	1	95.18
1675852	1.425	1.2794526	null	conserved hypothetical protein	PA0423	57	92.86
1675855	0.432	0.80351627	mexR	multidrug resistance operon repressor MexR	PA0424	62	94.13
1675858	1.060	0.8816047	mexA	Resistance-Nodulation-Cell Division (RND)	PA0425	99	95.75
1675861	0.799	0.6271652	null	conserved hypothetical protein	PA0422	95	92.08
1675864	1.061	0.8487488	bioA	adenosylmethionine-8-amino-7-oxononanoate	PA0420	150	92.7
1675867	1.170	0.7685919	null	hypothetical protein	PA0429	109	94.88
1675870	0.946	0.77592975	metF	5,10-methylenetetrahydrofolate reductase	PA0430	1	92.23
1675873	0.985	0.8765375	oprM	Major intrinsic multiple antibiotic resistance	PA0427	31	94.29
1675876	0.977	0.70269984	chpE	probable chemotaxis protein	PA0417	18	93.02
1675879	0.997	0.8057102	null	probable cystathionine gamma-lyase	PA0400	101	92.14
1675882	0.909	0.8985237	pilJ	twitching motility protein PilJ	PA0411	46	92.34
1675885	1.085	0.9679836	sahH	S-adenosyl-L-homocysteine hydrolase	PA0432	76	93.2
1675889	1.093	0.9393317	null	hypothetical protein	PA0388	259	94.08
1675891	0.820	0.7109445	null	hypothetical protein	PA0431	307	95.35
1675894	0.853	0.85988027	null	hypothetical protein	PA0433	1	92.84
1675896	1.093	0.9270837	mexB	Resistance-Nodulation-Cell Division (RND)	PA0426	168	92.58
1675899	1.076	-1.84375	null	hypothetical protein	PA0434	199	97.86
1675902	0.850	0.73793226	pilH	twitching motility protein PilH	PA0409	212	93.49
1675907	0.810	0.54850495	null	probable transcriptional regulator	PA0436	534	94.25
1675908	0.829	0.43380752	dht	dihydropyrimidinase	PA0441	255	92.74

1675911	1.122	0.9272443	codB	cytosine permease	PA0438	474	94.86
1675914	1.024	0.7190626	null	hypothetical protein	PA0435	1	94.03
1675917	0.398	0.2824074	null	N-carbamoyl-beta-alanine amidohydrolase	PA0444	1	92.88
1675920	1.468	1.0530356	null	probable oxidoreductase	PA0440	159	95.37
1675923	0.586	0.13287835	null	probable transcriptional regulator	PA0448	71	95.76
1675926	0.890	0.66748685	null	probable ATP-dependent RNA helicase	PA0428	312	94.26
1675929	0.842	0.5938229	thiG	thiamine biosynthesis protein, thiazole moiety	PA0381	196	92.51
1675932	1.110	0.6251853	null	probable stomatin-like protein	PA0452	66	95.64
1675935	1.448	1.2332258	null	hypothetical protein	PA0453	31	96.07
1675938	0.870	0.912675	codA	cytosine deaminase	PA0437	159	96.91
1675941	1.128	1.1218704	null	hypothetical protein	PA0449	97	94.17
1675943	0.817	0.58601177	null	hypothetical protein	PA0421	115	95.55
1675946	0.651	0.63468254	null	probable cold-shock protein	PA0456	35	93.06
1675947	1.211	0.5871836	gcdH	glutaryl-CoA dehydrogenase	PA0447	122	93.27
1675950	1.004	0.90882033	null	probable phosphate transporter	PA0450	115	93.71
1675953	1.009	0.43888888	null	probable oxidoreductase	PA0439	5	92.64
1675956	1.057	0.7674955	null	hypothetical protein	PA0462	14	93.45
1675959	0.991	0.7632825	dbpA	RNA helicase DbpA	PA0455	31	93.09
1675962	1.032	0.7910553	null	hypothetical protein	PA0460	48	97.4
1675965	1.059	0.9335148	null	conserved hypothetical protein	PA0446	1	95.27
1675968	1.173	1.1743352	null	hypothetical protein	PA0457	38	94.72
1675971	1.095	0.94733685	null	conserved hypothetical protein	PA0451	54	97.9
1675975	1.009	4.9904757	null	hypothetical protein	PA0466	221	94.41
1675976	0.947	0.77465516	creC	two-component sensor CreC	PA0464	2	92.55
1675979	1.009	2.0126984	creD	inner membrane protein CreD	PA0465	341	93.71
1675982	0.597	0.44067854	null	conserved hypothetical protein	PA0461	109	94.5
1675985	0.915	-0.23825398	null	probable transporter	PA0443	149	93.69
1675988	1.022	0.81010073	null	hypothetical protein	PA0468	665	96.82
1675991	1.373	1.2025464	null	probable sigma-70 factor, ECF subfamily	PA0472	5	92.26
1675994	0.913	0.5875045	null	probable transmembrane sensor	PA0471	83	95.74
1675997	0.752	0.5447367	null	hypothetical protein	PA0469	470	92.76
1676000	1.401	0.8833933	null	probable glutathione S-transferase	PA0473	129	94.4
1676003	0.984	0.96414506	null	probable hydroxamate-type ferrisiderophore	PA0470	1	97.61
1676006	0.862	0.7235497	null	probable transposase	PA0445	45	92.38
1676009	1.303	0.96158373	null	conserved hypothetical protein	PA0467	49	96.77
1676012	1.178	0.95136625	null	probable transcriptional regulator	PA0477	10	92.4
1676015	0.979	0.42187572	null	probable N-acetyltransferase	PA0478	229	92.39
1676019	1.074	0.9753847	null	probable transcriptional regulator	PA0475	55	95.12
1676021	0.977	0.65545636	null	hypothetical protein	PA0481	10	92.27
1676024	1.246	1.0299913	null	probable acetyltransferase	PA0483	209	92.85
1676027	1.076	4.5848417	null	probable hydrolase	PA0480	104	95.64
1676030	0.595	0.9892038	null	probable permease	PA0476	324	97.85
1676033	1.367	0.66556644	null	probable transcriptional regulator	PA0479	1	92.53
1676036	0.766	0.32331735	null	conserved hypothetical protein	PA0484	233	92.28
1676039	1.009	1.9227188	null	probable phosphoribosyl transferase	PA0489	242	93.09
1676041	1.684	1.8787991	null	conserved hypothetical protein	PA0488	70	94.59
1676042	1.973	0.8805073	null	hypothetical protein	PA0474	48	94.69
1676045	0.982	0.7129076	null	hypothetical protein	PA0490	131	93.04
1676047	1.071	0.76740944	null	conserved hypothetical protein	PA0485	248	92.78
1676050	0.792	0.7435354	null	probable biotin-requiring enzyme	PA0493	100	92.01
1676051	0.935	0.6778941	null	probable molybdenum transport regulator	PA0487	129	94.77
1676054	0.977	0.78840315	null	conserved hypothetical protein	PA0492	507	96.22
1676057	1.009	-2.8428903	null	hypothetical protein	PA0497	219	92.53
1676060	0.966	0.7931667	null	hypothetical protein	PA0498	459	93.22
1676063	1.296	-1.2500987	null	probable major facilitator superfamily (MFS)	PA0458	81	94.15
1676066	0.609	0.14482169	null	conserved hypothetical protein	PA0496	582	94.67
1676069	1.338	1.0178039	null	probable pili assembly chaperone	PA0499	10	92.04
1676072	0.980	0.8367241	null	hypothetical protein	PA0495	288	92.22
1676075	0.712	-0.49672168	null	probable transcriptional regulator	PA0491	482	96.33
1676078	1.113	0.9904636	null	hypothetical protein	PA0505	162	97.5
1676080	0.641	-13.500651	bioB	biotin synthase	PA0500	86	96.77
1676083	1.074	2.7080808	bioD	dethiobiotin synthase	PA0504	1	93.23
1676086	0.921	0.7350391	creB	two-component response regulator CreB	PA0463	15	92.12
1676089	0.899	0.53110665	null	probable biotin synthesis protein BioC	PA0503	194	95.34
1676092	0.845	0.6966024	bioF	8-amino-7-oxononanoate synthase	PA0501	722	94.74
1676095	1.258	1.0138365	nirN	probable c-type cytochrome	PA0509	540	92.23
1676098	1.038	0.9094486	null	conserved hypothetical protein	PA0512	185	96.24
1676101	0.513	-0.76082814	null	probable acyl-CoA carboxylase subunit	PA0494	610	94.04
1676104	0.956	0.863402	null	probable transcriptional regulator	PA0513	143	94.27
1676107	1.100	0.9586994	nirL	heme d1 biosynthesis protein NirL	PA0514	322	92.3
1676110	1.059	0.963147	nirJ	heme d1 biosynthesis protein NirJ	PA0511	98	93.78
1676113	0.940	0.98985165	nirF	heme d1 biosynthesis protein NirF	PA0516	1	96.67
1676116	1.026	0.98210984	null	probable transcriptional regulator	PA0515	261	92.54
1676120	0.876	0.8580898	nirC	probable c-type cytochrome precursor	PA0517	237	97.64
1676122	1.196	1.09264	nirM	cytochrome c-551 precursor	PA0518	57	93.18
1676124	0.723	0.63926405	null	hypothetical protein	PA0522	118	95.45
1676125	1.113	1.1578388	nirS	nitrite reductase precursor	PA0519	56	96.87
1676128	0.888	0.7708918	null	probable cytochrome c oxidase subunit	PA0521	433	92.97
1676131	0.741	0.5649675	null	hypothetical protein	PA0406	47	95.24
1676134	1.207	0.6477502	null	probable biotin biosynthesis protein bioH	PA0502	513	96.21
1676137	1.274	1.2151916	nirQ	regulatory protein NirQ	PA0520	264	93.51
1676140	0.963	1.1107442	norC	nitric-oxide reductase subunit C	PA0523	69	93.97
1676143	1.229	1.0726999	null	probable acyl-CoA dehydrogenase	PA0506	223	95
1676146	1.323	1.3471986	norB	nitric-oxide reductase subunit B	PA0524	144	96.32
1676149	1.113	1.0913479	dnr	transcriptional regulator Dnr	PA0527	32	92.95
1676152	1.495	1.2675306	null	probable acyl-CoA dehydrogenase	PA0508	34	94.5
1676155	1.601	1.1476392	null	conserved hypothetical protein	PA0529	472	92.07
1676158	1.026	0.5181224	null	hypothetical protein	PA0532	64	92.14
1676161	0.831	0.87120116	null	hypothetical protein	PA0526	148	94.48
1676162	0.813	0.59208417	glcB	malate synthase G	PA0482	86	95.29
1676165	0.893	0.91188633	null	probable dinitrification protein NorD	PA0525	1277	94.35
1676168	1.861	1.5054574	null	probable class III pyridoxal phosphate-dependent	PA0530	129	96.24
1676171	1.181	0.87415683	null	hypothetical protein	PA0536	447	92.41
1676174	1.513	1.1961032	dsbB	disulfide bond formation protein	PA0538	1	92.64
1676177	1.205	1.07989	null	conserved hypothetical protein	PA0537	433	92.35
1676180	0.762	0.5392024	null	conserved hypothetical protein	PA0486	82	93.02
1676183	1.009	0.10209525	null	probable transcriptional regulator	PA0528	8	92.14
1676186	1.009	-0.09193106	null	hypothetical protein	PA0540	147	93.72
1676189	1.156	1.1344404	null	conserved hypothetical protein	PA0534	27	93.6
1676192	0.569	0.119091414	null	probable transcriptional regulator	PA0533	508	92.38
1676195	1.109	0.8071553	null	hypothetical protein	PA0544	3	92.13
1676198	1.253	0.69462585	null	probable acyl-CoA dehydrogenase	PA0507	236	92.85
1676201	3.964	-1.2704761	null	probable glutamine amidotransferase	PA0531	1	94.45
1676204	1.058	0.3499533	null	hypothetical protein	PA0541	44	94.62
1676207	0.935	0.5570808	null	probable transcriptional regulator	PA0547	7	93.42
1676210	1.013	0.6727128	null	conserved hypothetical protein	PA0550	92	93.09
1676213	1.026	2.9745584	null	hypothetical protein	PA0545	318	92.88
1676216	0.883	0.67000806	epd	D-erythrose 4-phosphate dehydrogenase	PA0551	130	94.43
1676219	1.271	0.92212117	metK	methionine adenosyltransferase	PA0546	37	93.91

1676222	0.963	0.68769234	null	hypothetical protein	PA0553	83	95.88
1676224	0.807	0.36025143	null	probable ClpA/B protease ATP binding subunit	PA0459	9	92.56
1676227	0.761	0.27221352	null	hypothetical protein	PA0539	187	92.84
1676230	1.009	0.2847619	null	hypothetical protein	PA0557	10	93.57
1676233	1.079	1.0224987	fda	fructose-1,6-bisphosphate aldolase	PA0555	108	95.61
1676236	0.880	0.7590959	null	hypothetical protein	PA0554	276	92.45
1676239	1.135	0.7796118	pgk	phosphoglycerate kinase	PA0552	81	97.42
1676242	0.727	0.5962813	null	hypothetical protein	PA0556	402	94.88
1676245	1.225	0.953129	null	conserved hypothetical protein	PA0560	7	93.98
1676248	0.595	0.4596932	null	probable transcriptional regulator	PA0535	466	93.31
1676251	0.688	1.1095238	null	hypothetical protein	PA0543	423	93.51
1676254	1.218	0.5186347	null	hypothetical protein	PA0561	169	92.23
1676257	1.316	0.9757721	null	hypothetical protein	PA0549	174	92.52
1676260	1.140	0.86276215	tktA	transketolase	PA0548	117	94.79
1676263	1.007	0.86911994	null	conserved hypothetical protein	PA0454	1	97.67
1676266	0.983	0.9069295	null	conserved hypothetical protein	PA0563	48	92.74
1676269	0.796	0.60496384	null	probable hydrolase	PA0562	1	94.76
1676272	0.600	-0.6410766	null	conserved hypothetical protein	PA0558	311	96.55
1676275	0.849	-0.23004456	null	conserved hypothetical protein	PA0559	255	93.85
1676278	0.832	0.35880226	null	hypothetical protein	PA0574	65	94.46
1676281	0.826	0.49292845	null	hypothetical protein	PA0568	12	93.14
1676285	0.980	0.8547329	null	conserved hypothetical protein	PA0567	1	94.61
1676286	1.009	1.0587698	null	hypothetical protein	PA0571	85	94.11
1676289	1.009	1.6536682	null	hypothetical protein	PA0569	269	92.86
1676292	0.519	-0.39595357	null	hypothetical protein	PA0572	29	92.08
1676295	1.603	0.6360761	null	probable transcriptional regulator	PA0564	391	95.97
1676298	0.541	0.48890716	rpsU	30S ribosomal protein S21	PA0579	138	95.46
1676299	0.719	0.64145905	null	conserved hypothetical protein	PA0578	119	93.01
1676302	0.767	-0.59324086	null	hypothetical protein	PA0573	67	94.1
1676305	0.773	0.52953225	gcp	O-sialoglycoprotein endopeptidase	PA0580	82	93.72
1676308	0.805	0.6945058	dnaG	DNA primase	PA0577	3	92.83
1676311	0.809	0.4412287	folB	dihydroneopterin aldolase	PA0582	173	93.41
1676314	1.040	0.04027779	null	hypothetical protein	PA0585	207	95.3
1676317	1.037	1.0428482	null	conserved hypothetical protein	PA0581	24	95.2
1676320	0.878	0.77242064	rpoD	sigma factor RpoD	PA0576	153	92.25
1676323	0.854	0.37473923	cca	tRNA nucleotidyl transferase	PA0584	27	94.04
1676326	0.783	0.196027	null	conserved hypothetical protein	PA0587	12	92.63
1676329	0.947	0.7597142	null	hypothetical protein	PA0583	148	95.16
1676332	0.999	0.8374691	null	conserved hypothetical protein	PA0542	185	92.59
1676335	1.325	0.9851859	null	conserved hypothetical protein	PA0586	334	92.28
1676338	1.007	0.85418844	null	conserved hypothetical protein	PA0589	271	95.02
1676341	1.286	1.0666068	null	conserved hypothetical protein	PA0588	155	92.79
1676344	1.036	0.84130466	ostA	organic solvent tolerance protein OstA	PA0595	62	94.83
1676347	0.888	0.7670332	null	hypothetical protein	PA0566	454	97.08
1676349	0.954	0.11403509	null	conserved hypothetical protein	PA0591	33	95.17
1676352	0.816	0.5676481	null	probable nucleotidyl transferase	PA0597	130	93.77
1676355	1.569	1.0184877	null	hypothetical protein	PA0596	125	94.99
1676358	0.958	0.6791979	ksgA	rRNA (adenine-N6,N6)-dimethyltransferase	PA0592	58	95.46
1676361	0.775	0.6461589	null	hypothetical protein	PA0598	140	95.26
1676364	0.791	0.68335927	pdxA	pyridoxal phosphate biosynthetic protein PdxA	PA0593	208	97.52
1676367	1.466	1.1173049	null	probable two-component response regulator	PA0601	276	93.92
1676370	0.379	-1.2014291	null	probable binding protein component of ABC	PA0604	123	93.29
1676373	1.228	0.36521098	null	probable binding protein component of ABC	PA0602	92	93.41
1676376	0.697	0.48689866	null	hypothetical protein	PA0570	122	92.35
1676379	0.705	0.4395259	null	probable ATP-binding component of ABC	PA0603	20	92.01
1676382	1.030	0.9846443	null	probable uroporphyrin-III c-methyltransferase	PA0510	7	92.57
1676385	1.205	0.8650206	null	hypothetical protein	PA0599	211	94.06
1676389	1.364	1.0042323	null	conserved hypothetical protein	PA0565	120	95
1676391	0.915	-2.9931188	null	probable permease of ABC transporter	PA0606	196	92.78
1676394	0.860	0.62987477	rpe	ribulose-phosphate 3-epimerase	PA0607	2	93.55
1676397	0.747	0.5863516	null	probable phosphoglycolate phosphatase	PA0608	30	96.06
1676400	0.997	0.83032495	prtN	transcriptional regulator PrtN	PA0610	1	93.58
1676403	1.289	0.89917326	null	hypothetical protein	PA0615	1	92.41
1676406	1.224	0.8625983	null	hypothetical protein	PA0612	1	93.95
1676407	1.279	0.88831675	null	probable bacteriophage protein	PA0617	136	94.23
1676410	1.504	1.232336	null	hypothetical protein	PA0614	35	94.71
1676413	1.559	1.25103	null	conserved hypothetical protein	PA0621	135	92.04
1676416	1.146	0.94901055	null	hypothetical protein	PA0616	210	92.37
1676419	1.064	0.74409753	null	conserved hypothetical protein	PA0575	107	97.46
1676422	0.606	0.41133648	null	probable permease of ABC transporter	PA0605	168	93.33
1676425	0.887	0.5513043	null	probable two-component sensor	PA0600	92	97.23
1676428	1.151	0.8013469	null	probable bacteriophage protein	PA0618	116	92.29
1676431	0.717	-2.9392207	null	hypothetical protein	PA0613	15	93.24
1676434	1.300	0.9730592	null	hypothetical protein	PA0624	52	96.22
1676437	1.250	0.5606409	null	conserved hypothetical protein	PA0627	22	93.17
1676439	1.009	1.7664655	null	probable bacteriophage protein	PA0620	71	92.41
1676442	2.418	1.0301411	null	probable bacteriophage protein	PA0622	15	92.7
1676445	0.894	0.7313104	apaH	bis(5'-nucleosyl)-tetraphosphatase	PA0590	134	94.6
1676448	1.223	1.0023037	null	probable bacteriophage protein	PA0619	37	92.64
1676451	1.705	0.7205121	null	hypothetical protein	PA0632	34	92.59
1676453	1.368	1.042754	null	hypothetical protein	PA0631	1	93.04
1676455	1.061	1.752381	null	hypothetical protein	PA0630	68	96.41
1676457	2.295	1.7352396	null	hypothetical protein	PA0626	57	93.29
1676460	1.040	0.89606106	null	probable bacteriophage protein	PA0623	45	95.9
1676463	1.097	0.8712374	null	hypothetical protein	PA0635	112	96.71
1676466	1.009	0.75616884	null	conserved hypothetical protein	PA0629	166	94.19
1676469	1.669	0.9693939	null	hypothetical protein	PA0633	47	93.76
1676472	1.277	0.8655571	null	probable bacteriophage protein	PA0640	178	96
1676476	1.527	1.2998209	null	conserved hypothetical protein	PA0637	208	94.25
1676478	1.628	0.91897976	null	conserved hypothetical protein	PA0639	234	93.91
1676481	1.437	0.6915611	prtR	transcriptional regulator PrtR	PA0611	43	93.82
1676484	1.426	0.6422107	null	hypothetical protein	PA0634	220	94.29
1676486	1.027	0.72630644	null	hypothetical protein	PA0645	114	92.56
1676487	1.690	2.1008523	null	hypothetical protein	PA0644	35	92.46
1676489	2.771	0.9555195	null	probable bacteriophage protein	PA0641	185	95.03
1676492	1.819	0.8721798	null	hypothetical protein	PA0643	289	96.03
1676495	1.127	0.94401103	null	hypothetical protein	PA0648	67	92.28
1676496	1.009	-0.6981859	null	hypothetical protein	PA0642	517	92.22
1676497	1.001	0.8298073	null	hypothetical protein	PA0647	81	92.26
1676498	1.413	0.31271133	null	hypothetical protein	PA0625	37	93.97
1676501	0.636	0.4968241	trpC	indole-3-glycerol-phosphate synthase	PA0651	1	93.88
1676504	1.527	1.1454464	null	hypothetical protein	PA0636	43	94.01
1676507	1.082	2.0007143	null	conserved hypothetical protein	PA0628	94	92.47
1676510	0.920	0.76986784	vfr	transcriptional regulator Vfr	PA0652	67	92.95
1676513	0.444	-0.08470907	trpD	anthranilate phosphoribosyltransferase	PA0650	28	92.39
1676516	0.709	0.5927974	trpE	anthranilate synthetase component I	PA0609	48	94.31
1676519	1.009	1.2947311	null	probable bacteriophage protein	PA0638	121	94.16
1676522	1.032	0.88868034	trpG	anthranilate synthase component II	PA0649	63	92.37
1676525	1.457	0.68773246	null	hypothetical protein	PA0646	149	92.75

1676528	1.595	1.2128214	null	probable HIT family protein	PA0656	99	93.52
1676530	0.853	0.7423114	null	conserved hypothetical protein	PA0665	2	92.64
1676533	1.283	0.6486834	null	hypothetical protein	PA0660	43	93.46
1676536	0.979	0.69401836	speD	S-adenosylmethionine decarboxylase proenzyme	PA0654	61	93.05
1676539	0.881	0.7252515	null	hypothetical protein	PA0655	318	93.2
1676542	0.648	0.3812965	null	hypothetical protein	PA0663	245	93.98
1676545	0.813	0.69318944	null	hypothetical protein	PA0659	45	94.69
1676548	1.084	0.9726149	null	conserved hypothetical protein	PA0667	58	92.93
1676551	0.689	0.62341225	null	conserved hypothetical protein	PA0666	72	92.83
1676554	0.797	0.6569146	tyrZ	tyrosyl-tRNA synthetase 2	PA0668	35	95.16
1676557	0.983	0.6409359	argC	N-acetyl-gamma-glutamyl-phosphate reductase	PA0662	2	92.32
1676560	0.788	0.14872618	null	hypothetical protein	PA0671	64	95.44
1676563	1.376	0.34640566	hemO	heme oxygenase	PA0672	2	92.98
1676566	0.865	0.64122224	null	hypothetical protein	PA0664	3	92.14
1676570	1.009	1.542122	null	hypothetical protein	PA0673	231	95.19
1676571	0.984	0.7020011	null	conserved hypothetical protein	PA0653	145	92.17
1676574	1.194	0.95573676	null	HxcW putative pseudopilin	PA0677	484	93.56
1676577	1.629	0.9710679	null	probable ATPase	PA0657	25	92.19
1676580	0.986	5.848254	null	hypothetical protein	PA0679	185	92.62
1676583	1.009	1.2259307	null	HxcT pseudopilin	PA0681	2	92.45
1676586	1.009	1.7323996	null	HxcX atypical pseudopilin	PA0682	240	94.73
1676589	1.053	0.7674089	null	probable sigma-70 factor, ECF subfamily	PA0675	20	92.14
1676592	1.071	0.73670185	null	probable type II secretion system protein	PA0684	536	93.33
1676594	0.999	0.80661136	null	hypothetical protein	PA0670	308	96.56
1676597	1.009	1.5374849	null	probable transmembrane sensor	PA0676	36	94.72
1676600	1.009	-4.5235786	null	probable DNA polymerase alpha chain	PA0669	28	93.04
1676603	8.533	-0.79735494	null	probable type II secretion system protein	PA0683	360	97.76
1676606	1.253	0.3852078	null	hypothetical protein	PA0674	156	95.79
1676609	1.034	1.0171839	null	conserved hypothetical protein	PA0661	11	92.68
1676611	1.152	0.79398644	null	probable binding protein component of ABC	PA0688	31	97.14
1676614	1.009	0.8966595	null	hypothetical protein	PA0689	32	92.86
1676617	1.009	0.8806349	null	hypothetical protein	PA0691	98	93.41
1676619	1.002	0.8000792	null	probable type II secretion system protein	PA0687	760	97.06
1676622	0.659	0.49676695	null	probable short-chain dehydrogenase	PA0658	297	92.63
1676625	1.052	0.3478846	exbD2	transport protein ExbD	PA0694	263	96.26
1676628	1.009	1.5971265	null	hypothetical protein	PA0692	1	92.99
1676631	1.163	0.6976079	null	probable type II secretion system protein	PA0685	2	93.14
1676634	1.009	1.848903	null	hypothetical protein	PA0695	281	93.2
1676637	1.303	0.8267419	null	hypothetical protein	PA0696	234	96.46
1676640	2.744	0.7305602	null	hypothetical protein	PA0697	346	92.63
1676643	2.107	4.667798	null	probable transcriptional regulator	PA0701	112	93.43
1676646	0.751	-1.2780886	null	HxcU putative pseudopilin	PA0678	6	92.99
1676649	0.580	0.2251776	null	probable amidase	PA0704	55	93.12
1676652	0.619	-0.1116334	null	hypothetical protein	PA0702	131	94.37
1676655	1.074	1.9047619	null	probable peptidyl-prolyl cis-trans isomerase,	PA0699	356	93.92
1676658	1.176	0.9297905	null	hypothetical protein	PA0700	253	96.01
1676659	1.166	0.9638262	null	probable major facilitator superfamily (MFS)	PA0703	112	92.04
1676662	1.009	1.3860508	null	HxcV putative pseudopilin	PA0680	276	96.15
1676665	2.109	-0.5917939	null	hypothetical protein	PA0709	62	93.43
1676668	0.626	0.426344	cat	chloramphenicol acetyltransferase	PA0706	22	92.95
1676671	0.803	1.6605129	toxR	transcriptional regulator ToxR	PA0707	23	93.56
1676674	0.810	0.64576256	null	probable glycosyl transferase	PA0705	15	94.43
1676677	0.870	0.32875767	null	probable transcriptional regulator	PA0708	59	93.29
1676680	1.009	-0.3288228	null	hypothetical protein	PA0711	81	92.58
1676684	4.312	0.7709447	gloA2	lactoylglutathione lyase	PA0710	1	94.97
1676685	1.883	1.3726346	null	hypothetical protein	PA0713	193	93.43
1676687	1.009	-0.48461533	null	hypothetical protein	PA0690	233	94.18
1676690	0.866	0.58147085	null	hypothetical protein of bacteriophage Pf1	PA0717	75	94.34
1676692	1.176	1.1174579	null	hypothetical protein of bacteriophage Pf1	PA0719	160	96.14
1676695	1.080	0.5970549	null	hypothetical protein of bacteriophage Pf1	PA0722	1	93.91
1676698	1.176	0.9358465	null	hypothetical protein of bacteriophage Pf1	PA0721	16	97.58
1676699	1.524	1.172699	null	hypothetical protein of bacteriophage Pf1	PA0718	34	97.95
1676702	0.962	0.8551127	surA	peptidyl-prolyl cis-trans isomerase SurA	PA0594	31	95.66
1676705	1.056	0.84987134	coaB	coat protein B of bacteriophage Pf1)	PA0723	7	92.55
1676707	1.009	0.862465	null	hypothetical protein from bacteriophage Pf1	PA0727	84	93.72
1676710	0.965	0.5972034	null	helix destabilizing protein of bacteriophage	PA0720	9	92.28
1676713	0.659	0.5757448	null	hypothetical protein	PA0716	640	93.24
1676714	0.114	1.0558207	null	hypothetical protein	PA0712	3	93.16
1676716	1.052	0.7588744	null	probable coat protein A of bacteriophage Pf1	PA0724	152	93.56
1676719	1.097	0.84670794	null	hypothetical protein of bacteriophage Pf1	PA0725	128	92.85
1676721	0.374	4.4512386	null	probable bacteriophage integrase	PA0728	15	92.26
1676724	1.376	0.9202835	null	hypothetical protein of bacteriophage Pf1	PA0726	5	92.09
1676727	1.240	0.9904808	null	hypothetical protein	PA0734	112	96.23
1676729	0.999	0.8008259	null	probable transferase	PA0730	30	92.42
1676732	1.009	1.472055	null	probable type II secretion system protein	PA0686	366	92.68
1676735	0.944	0.787145	null	hypothetical protein	PA0732	201	92.03
1676738	1.009	0.29775995	null	hypothetical protein	PA0731	103	94.49
1676741	0.142	0.41253287	null	hypothetical protein	PA0735	131	92.09
1676744	1.009	-0.5220289	null	probable transcriptional regulator	PA0739	245	93.87
1676747	1.249	0.55839574	null	conserved hypothetical protein	PA0738	133	96.85
1676749	1.176	0.46071807	null	hypothetical protein	PA0737	3	92.5
1676752	1.100	0.88456506	null	probable pseudouridylate synthase	PA0733	54	96.06
1676755	1.066	0.7966348	null	hypothetical protein	PA0742	51	92.1
1676758	0.711	0.2502076	null	probable beta-lactamase	PA0740	50	97.92
1676761	1.269	1.1300448	null	probable enoyl-CoA hydratase/isomerase	PA0745	10	92.13
1676764	1.284	0.7378502	null	conserved hypothetical protein	PA0741	180	96.51
1676767	1.002	0.83162403	null	hypothetical protein	PA0736	400	94.67
1676770	0.852	0.6344341	null	hypothetical protein	PA0749	129	92.49
1676773	1.481	1.1379726	null	probable enoyl-CoA hydratase/isomerase	PA0744	44	93.78
1676776	1.061	0.8695105	null	hypothetical protein	PA0714	88	95.89
1676779	1.058	0.8936124	ung	uracil-DNA glycosylase	PA0750	118	95.36
1676782	1.013	2.247949	null	conserved hypothetical protein	PA0751	217	93.05
1676785	1.835	-0.38918334	null	probable acyl-CoA dehydrogenase	PA0746	235	93.29
1676788	0.821	0.5649458	null	hypothetical protein	PA0758	109	92.74
1676791	1.009	0.5676099	null	probable two-component sensor	PA0757	405	95.92
1676795	1.285	-7.016365	null	hypothetical protein	PA0753	381	96.24
1676797	0.638	0.4484633	null	conserved hypothetical protein	PA0760	60	92.97
1676800	0.960	0.8473204	algU	sigma factor AlgU	PA0762	16	92.48
1676803	0.845	0.6392641	null	conserved hypothetical protein	PA0759	1	93.88
1676806	1.009	0.66568804	null	conserved hypothetical protein	PA0752	573	95.53
1676811	1.028	6.2966065	null	probable 3-hydroxyisobutyrate dehydrogenase	PA0743	803	93.99
1676812	1.155	0.89865446	nadB	L-aspartate oxidase	PA0761	47	96.98
1676815	5.482	0.5766428	null	hypothetical protein	PA0754	68	97.38
1676818	0.917	0.9141466	mucB	negative regulator for alginate biosynthesis	PA0764	1	93.94
1676821	0.973	0.9127129	mucA	anti-sigma factor MucA	PA0763	139	94.79
1676824	0.680	0.5917134	mucC	positive regulator for alginate biosynthesis	PA0765	286	94.55
1676827	0.939	0.64490426	mucD	serine protease MucD precursor	PA0766	39	96.58
1676830	0.844	0.69754326	null	hypothetical protein	PA0769	180	92.21
1676831	0.948	-0.16704544	null	hypothetical protein	PA0698	336	93.47

1676834	0.981	0.8090464	lepB	signal peptidase I	PA0768	90	95.22
1676837	0.966	0.48027262	recO	DNA repair protein RecO	PA0772	12	95.87
1676840	1.215	0.32819742	null	still frameshift probable transcriptional	PA0748	211	96.72
1676843	0.748	-1.6694828	era	GTP-binding protein Era	PA0771	107	97.41
1676846	1.094	0.7674099	rnc	ribonuclease III	PA0770	160	95.4
1676849	1.254	0.5133753	null	hypothetical protein	PA0776	21	92.44
1676852	0.865	0.63389605	null	probable ATP-dependent protease	PA0779	2	92.49
1676855	1.266	1.1383007	null	conserved hypothetical protein	PA0774	19	97.52
1676858	1.272	-0.35848486	null	probable porin	PA0755	546	94.29
1676861	0.694	0.4959467	null	hypothetical protein	PA0777	144	96.74
1676864	1.142	1.3093617	pruR	proline utilization regulator	PA0780	1	94.47
1676867	1.009	1.7893775	null	probable acyl carrier protein phosphodiesterase	PA0785	65	96.22
1676870	0.946	0.5456109	pdxJ	pyridoxal phosphate biosynthetic protein PdxJ	PA0773	139	95.88
1676873	1.164	1.0071492	null	hypothetical protein	PA0781	6	95.11
1676876	1.057	2.3817458	null	probable transporter	PA0786	47	92.88
1676879	0.901	0.5281678	null	probable transcriptional regulator	PA0784	101	93.77
1676882	0.784	0.5610878	putA	proline dehydrogenase PutA	PA0782	1	92.66
1676885	1.345	0.9016989	null	probable transcriptional regulator	PA0791	1	97.09
1676888	1.147	1.0074801	prpD	propionate catabolic protein PrpD	PA0792	3	92.39
1676891	0.834	0.5252098	null	probable amino acid permease	PA0789	3	92.18
1676894	0.802	0.65920365	icp	inhibitor of cysteine peptidase	PA0778	200	94.21
1676897	2.243	1.0629025	null	hypothetical protein	PA0790	3	92.61
1676900	1.801	1.2847062	null	hypothetical protein	PA0788	3	92.42
1676903	1.009	1.3868364	pmtA	phospholipid methyltransferase	PA0798	1	93.9
1676906	1.030	0.7736447	lepA	GTP-binding protein LepA	PA0767	40	92.46
1676909	1.176	0.9756969	putP	sodium/proline symporter PutP	PA0783	13	92.41
1676912	0.867	0.52818364	null	hypothetical protein	PA0787	289	92.84
1676915	0.477	0.8803717	null	probable transcriptional regulator	PA0797	53	92.29
1676918	1.679	0.8831371	null	hypothetical protein	PA0793	186	93.62
1676921	1.106	0.6652387	null	probable aconitate hydratase	PA0794	412	94.77
1676924	1.123	0.91640073	prpC	citrate synthase 2	PA0795	240	92.69
1676927	0.923	0.8027908	null	hypothetical protein	PA0805	24	92.67
1676928	0.858	0.75042045	null	hypothetical protein	PA0802	175	92.64
1676930	1.074	0.9123022	null	probable oxidoreductase	PA0804	200	94.3
1676933	1.009	1.3769119	null	hypothetical protein	PA0803	330	92.36
1676936	1.025	0.5944574	null	hypothetical protein	PA0800	172	95.59
1676939	0.947	0.7570284	null	probable haloacid dehalogenase	PA0810	1	93.27
1676942	0.839	0.65393317	null	probable helicase	PA0799	93	95.17
1676945	2.780	-0.91812867	null	hypothetical protein	PA0808	222	92.8
1676948	1.306	0.38571432	null	conserved hypothetical protein	PA0807	321	92.92
1676951	0.911	0.5795916	null	hypothetical protein	PA0806	54	92.9
1676953	1.009	0.14152074	exbB2	transport protein ExbB2	PA0693	71	92.95
1676956	1.175	0.78657645	null	probable transcriptional regulator	PA0815	23	92.63
1676959	4.070	2.4639244	null	conserved hypothetical protein	PA0814	49	97.81
1676960	1.273	0.70377606	null	hypothetical protein	PA0801	444	92.37
1676963	1.002	0.9205713	null	hypothetical protein	PA0818	1	96.79
1676966	1.438	2.2040532	null	hypothetical protein	PA0813	866	93.09
1676969	1.028	-0.6676283	null	hypothetical protein	PA0822	11	92
1676972	1.092	0.627354	null	hypothetical protein	PA0819	1	94.9
1676975	1.458	1.0892956	null	hypothetical protein	PA0821	80	92.29
1676978	0.749	0.66697437	null	hypothetical protein	PA0820	452	92.66
1676981	1.536	1.0965474	null	hypothetical protein	PA0823	90	93.48
1676983	1.100	-0.10388793	null	hypothetical protein	PA0825	222	92.5
1676985	1.329	0.45926848	null	hypothetical protein	PA0826	94	92.58
1676988	1.009	-0.2070138	null	hypothetical protein	PA0812	145	92.14
1676991	1.076	0.08614727	null	hypothetical protein	PA0824	127	94.82
1676994	0.570	-5.1337233	null	probable transcriptional regulator	PA0816	177	97.6
1676997	1.046	0.40816092	null	hypothetical protein	PA0827	98	94.87
1677000	1.070	-0.221651	null	probable transcriptional regulator	PA0828	94	95.73
1677003	1.251	0.7823431	null	hypothetical protein	PA0830	15	93.82
1677006	1.240	1.0031872	null	hypothetical protein	PA0833	78	92.38
1677009	0.817	0.26511264	null	probable two-component response regulator	PA0756	394	95.09
1677012	1.009	1.2747167	null	probable hydrolase	PA0829	173	93.55
1677015	1.192	1.031005	null	probable transporter	PA0809	186	92.33
1677018	1.075	0.7432353	null	conserved hypothetical protein	PA0832	18	92.55
1677021	0.996	0.4907996	null	conserved hypothetical protein	PA0834	171	94.06
1677024	0.725	0.22967324	prpB	carboxyphosphonoenolpyruvate phosphonomutase	PA0796	377	92.3
1677027	1.217	0.9591321	null	probable acetate kinase	PA0836	627	94.5
1677030	1.569	0.7571951	null	hypothetical protein	PA0841	234	92.27
1677033	1.111	0.9715643	oruR	transcriptional regulator OruR	PA0831	146	94.68
1677036	0.988	0.773598	null	probable glutathione peroxidase	PA0838	82	93.45
1677039	0.891	0.6530691	plcR	phospholipase accessory protein PlcR precursor	PA0843	162	92.42
1677042	0.929	0.5852777	null	conserved hypothetical protein	PA0775	76	95.95
1677045	1.326	0.5555985	null	probable sulfate uptake protein	PA0846	1	96.89
1677048	1.045	0.8059772	null	probable transcriptional regulator	PA0839	370	92.81
1677051	1.009	1.5709937	plcH	hemolytic phospholipase C precursor	PA0844	503	94.98
1677054	1.378	1.1874211	null	probable glycosyl transferase	PA0842	54	95.97
1677057	1.068	0.8941496	null	probable major facilitator superfamily (MFS)	PA0811	930	95.54
1677060	1.009	0.35758853	null	hypothetical protein	PA0847	101	93.73
1677063	0.822	0.64326084	null	hypothetical protein	PA0850	289	96.1
1677066	0.305	-0.14412603	trxB2	thioredoxin reductase 2	PA0849	1	94.2
1677069	0.764	-1.0953869	null	hypothetical protein	PA0851	82	92.2
1677072	1.211	0.919189	null	probable ring-cleaving dioxygenase	PA0817	1	93.46
1677075	0.965	0.88140213	null	conserved hypothetical protein	PA0845	251	93.44
1677078	0.618	0.5386169	bolA	morphogene protein BolA	PA0857	138	92.79
1677081	1.140	1.0005122	null	hypothetical protein	PA0856	144	94.66
1677084	1.155	0.7131832	null	probable oxidoreductase	PA0853	250	93.69
1677087	1.558	1.5190299	null	hypothetical protein	PA0859	118	95.57
1677090	0.895	0.7270379	null	hypothetical protein	PA0862	1	94.49
1677093	1.077	0.7849693	fumC2	fumarate hydratase	PA0854	389	94.86
1677096	0.916	0.7284172	null	hypothetical protein	PA0855	330	95.47
1677099	0.950	0.6686003	null	probable oxidoreductase	PA0840	527	92.45
1677102	1.133	0.9621382	null	probable oxidoreductase	PA0863	130	94.43
1677105	0.600	0.41517606	null	probable ATP-binding/permease fusion ABC	PA0860	40	93.03
1677108	0.675	0.655996	hpd	4-hydroxyphenylpyruvate dioxygenase	PA0865	5	93.23
1677111	0.502	2.0418274	null	conserved hypothetical protein	PA0868	12	92.49
1677114	1.163	1.1099087	null	conserved hypothetical protein	PA0858	15	92.75
1677117	0.873	0.75207007	null	hypothetical protein	PA0861	34	92.97
1677120	0.703	0.3280585	null	probable transcriptional regulator	PA0864	102	95.85
1677123	1.147	0.9996193	phhB	pterin-4-alpha-carbinolamine dehydratase	PA0871	1	95.49
1677126	1.457	1.2658665	pta	phosphate acetyltransferase	PA0835	90	92.3
1677129	1.195	0.4099543	null	hypothetical protein	PA0867	131	92.51
1677131	1.097	0.8771906	pbpG	D-alanyl-D-alanine-endopeptidase	PA0869	16	93.75
1677134	1.009	-2.7399383	null	hypothetical protein	PA0878	389	93.19
1677137	1.484	0.361039	null	hypothetical protein	PA0874	219	92.52
1677139	1.009	0.87479174	phhC	aromatic amino acid aminotransferase	PA0870	709	93.03
1677142	0.846	0.7011986	null	probable transcriptional regulator	PA0876	408	93.93
1677145	2.143	-0.41558447	cbpD	chitin-binding protein CbpD precursor	PA0852	67	96.04
1677148	1.009	1.5666668	null	probable acyl-CoA dehydrogenase	PA0879	245	92.56

1677151	1.289	1.0699862	null	hypothetical protein	PA0881	71	93.65
1677154	1.071	3.1083333	null	probable ring-cleaving dioxygenase	PA0880	176	95.34
1677157	0.179	0.91883355	aroP2	aromatic amino acid transport protein AroP2	PA0866	1211	92.39
1677161	1.244	0.23961325	null	probable C4-dicarboxylate-binding periplasmic	PA0884	447	97.2
1677165	1.101	0.824314	null	probable C4-dicarboxylate transporter	PA0885	321	94.74
1677166	0.829	0.54485124	null	probable transcriptional regulator	PA0877	20	94.58
1677169	0.906	0.68991405	null	hypothetical protein	PA0882	174	92.33
1677172	1.038	0.9182642	aotQ	arginine/ornithine transport protein AotQ	PA0889	34	95.9
1677175	1.009	-0.3436975	null	probable acyl-CoA lyase beta chain	PA0883	462	92.7
1677178	0.614	0.19235775	phhR	transcriptional regulator PhhR	PA0873	360	95.01
1677181	1.184	1.0316538	aotJ	arginine/ornithine binding protein AotJ	PA0888	220	92.76
1677184	1.312	1.1991231	null	hypothetical protein	PA0894	74	92.09
1677186	0.983	0.7914239	argR	transcriptional regulator ArgR	PA0893	1	95.71
1677189	1.010	0.7622485	aotM	arginine/ornithine transport protein AotM	PA0890	542	96.07
1677192	0.751	0.49963084	aruC	N-succinylglutamate 5-semialdehyde	PA0895	1	93.62
1677195	1.020	0.7164188	null	hypothetical protein	PA0891	1	94.64
1677198	0.348	0.30206507	acsA	acetyl-coenzyme A synthetase	PA0887	299	94.34
1677201	0.892	0.66130525	aruG	arginine/ornithine succinyltransferase AII	PA0897	224	96.08
1677204	0.786	0.68445456	aruF	arginine/ornithine succinyltransferase AI	PA0896	276	92.82
1677208	1.116	0.8794759	null	hypothetical protein	PA0900	200	93.68
1677209	1.898	1.0585097	aruB	succinylarginine dihydrolase	PA0899	58	94.22
1677212	0.793	0.7741712	rsmA	RsmA, regulator of secondary metabolites	PA0905	55	93.75
1677213	0.712	0.5667154	null	hypothetical protein	PA0902	562	93.98
1677216	0.949	0.82277954	aruD	succinylglutamate 5-semialdehyde dehydrogenase	PA0898	412	94.03
1677219	1.043	0.31225276	null	probable transcriptional regulator	PA0906	27	97.89
1677222	1.197	1.1753582	phhA	phenylalanine-4-hydroxylase	PA0872	243	92.51
1677225	1.160	0.9975351	lysC	aspartate kinase alpha and beta chain	PA0904	8	92.39
1677228	0.970	0.70006394	aotP	arginine/ornithine transport protein AotP	PA0892	338	95.31
1677231	1.073	0.7603835	aruE	succinylglutamate desuccinylase	PA0901	328	96.1
1677234	1.176	1.0407413	null	conserved hypothetical protein	PA0875	28	97.49
1677237	1.731	0.955441	null	hypothetical protein	PA0908	107	93.14
1677239	1.016	0.8652086	null	hypothetical protein	PA0909	25	93.38
1677241	1.135	0.94404393	null	hypothetical protein	PA0907	335	95.95
1677244	1.009	2.1322856	null	hypothetical protein	PA0911	37	94.4
1677247	0.945	0.6795248	null	probable C4-dicarboxylate transporter	PA0886	1217	92.18
1677250	0.887	1.9198529	null	hypothetical protein	PA0912	79	97.74
1677253	1.385	0.5161702	null	probable alkyl hydroperoxide reductase	PA0848	93	92.22
1677256	0.579	0.2566878	null	hypothetical protein	PA0914	208	96.16
1677259	0.832	0.54691404	null	conserved hypothetical protein	PA0916	9	93.02
1677262	0.927	0.35471472	null	hypothetical protein	PA0922	19	92.58
1677265	0.986	0.848863	alaS	alanyl-tRNA synthetase	PA0903	77	94.82
1677268	1.034	0.55782384	null	conserved hypothetical protein	PA0915	301	92.34
1677271	1.004	-0.26890758	kup	potassium uptake protein Kup	PA0917	1	94.26
1677274	0.791	0.6804397	mgtE	probable Mg transporter MgtE	PA0913	117	92.73
1677277	1.190	0.8950526	null	hypothetical protein	PA0919	1	92.95
1677280	0.351	-3.1765447	null	hypothetical protein	PA0925	62	93.99
1677283	1.189	1.0405346	null	cytochrome b561	PA0918	131	92.71
1677286	0.662	0.30334538	ldhA	D-lactate dehydrogenase (fermentative)	PA0927	124	92.24
1677289	1.045	0.7014829	dinP	DNA damage inducible protein P	PA0923	220	92.6
1677294	0.814	0.71532583	slyD	peptidyl-prolyl cis-trans isomerase SlyD	PA0837	382	92.62
1677295	0.980	0.9696995	null	hypothetical protein	PA0921	189	93.49
1677298	1.027	0.7501112	null	hypothetical protein	PA0926	272	94.25
1677301	0.969	0.7550117	cysM	cysteine synthase B	PA0932	2	92.23
1677304	1.106	0.7212763	null	two-component response regulator	PA0929	72	93.58
1677307	0.603	0.39093828	ygcA	probable RNA methyltransferase	PA0933	405	97.17
1677310	1.356	0.85008276	null	hypothetical protein	PA0939	152	92.35
1677313	0.661	0.58986557	null	hypothetical protein	PA0938	60	92.46
1677316	0.760	0.6227547	null	conserved hypothetical protein	PA0937	298	92.3
1677319	0.798	0.59172	null	two-component sensor	PA0930	48	93.04
1677322	2.872	1.2866614	null	hypothetical protein	PA0940	109	93.08
1677324	0.986	0.6592126	null	probable transcriptional regulator	PA0942	120	92.15
1677327	1.039	0.7730242	lpxO2	lipopolysaccharide biosynthetic protein LpxO2	PA0936	152	92.09
1677330	1.235	0.9297311	null	conserved hypothetical protein	PA0935	40	94.86
1677333	1.294	1.09338	null	hypothetical protein	PA0941	146	95.49
1677334	1.778	1.0836498	null	hypothetical protein	PA0910	218	95
1677337	0.839	0.68290454	purN	phosphoribosylaminoimidazole synthetase	PA0944	202	93.92
1677340	1.118	0.9390429	pirA	ferric enterobactin receptor PirA	PA0931	22	94.82
1677343	1.006	0.79835355	gacS	sensor/response regulator hybrid	PA0928	64	92.95
1677346	1.291	1.0050339	null	hypothetical protein	PA0943	95	93.58
1677349	0.933	0.72066337	purM	phosphoribosylaminoimidazole synthetase	PA0945	12	92.36
1677352	0.931	0.8057162	relA	GTP pyrophosphokinase	PA0934	65	95.48
1677355	1.312	0.89504796	null	hypothetical protein	PA0952	4	93.34
1677358	0.566	0.309919	null	probable arsenate reductase	PA0950	204	93.3
1677361	0.730	0.6013073	null	probable acylphosphatase	PA0954	78	92.38
1677364	0.941	0.8085502	null	hypothetical protein	PA0924	164	92.25
1677367	0.716	0.6317884	null	conserved hypothetical protein	PA0947	71	92.46
1677370	0.592	0.42344603	null	probable thioredoxin	PA0953	322	93.56
1677373	1.009	0.61605895	null	hypothetical protein	PA0959	7	92.36
1677376	1.028	3.8161767	null	hypothetical protein	PA0957	243	92.83
1677378	0.956	0.76621664	null	probable ribonuclease	PA0951	26	92.72
1677381	0.731	0.605889	null	probable cold-shock protein	PA0961	44	92.35
1677382	1.241	0.8897079	null	hypothetical protein	PA0946	50	93.34
1677385	0.474	0.42384917	oprD	Basic amino acid, basic peptide and imipenem	PA0958	63	94.51
1677388	1.036	0.9061977	null	conserved hypothetical protein	PA0964	72	92.67
1677391	0.879	0.6272737	null	hypothetical protein	PA0948	1	94.51
1677394	1.427	1.331119	null	probable dna-binding stress protein	PA0962	9	92.84
1677397	0.947	0.7513209	null	hypothetical protein	PA0920	1	95.92
1677400	0.321	-1.5557351	null	hypothetical protein	PA0960	93	94.23
1677402	0.852	0.6171739	aspS	aspartyl-tRNA synthetase	PA0963	2	92.84
1677405	0.805	0.7107636	null	conserved hypothetical protein	PA0968	156	93.71
1677407	0.729	0.53234655	ruvB	Holliday junction DNA helicase RuvB	PA0967	90	94.68
1677410	1.056	0.90784913	ruvA	Holliday junction DNA helicase RuvA	PA0966	81	92.09
1677413	0.764	0.58107346	null	hypothetical protein	PA0955	74	96.04
1677416	0.823	0.68605864	ruvC	Holliday junction resolvase RuvC	PA0965	109	92.06
1677419	1.359	1.0352173	wrbA	Trp repressor binding protein WrbA	PA0949	9	92.56
1677422	1.058	0.89224863	tolR	TolR protein	PA0970	284	92.33
1677424	0.997	0.8595953	proS	prolyl-tRNA synthetase	PA0956	98	96.08
1677427	0.726	0.7609866	null	conserved hypothetical protein	PA0974	39	92.93
1677430	0.541	1.4791224	null	probable radical activating enzyme	PA0975	35	92.92
1677433	1.548	0.8353809	null	hypothetical protein	PA0980	86	92.63
1677434	1.155	0.80876416	null	hypothetical protein	PA0982	68	93.46
1677437	0.927	0.7653518	tolQ	TolQ protein	PA0969	35	92.01
1677440	0.966	0.83410853	tolA	TolA protein	PA0971	85	94.24
1677443	0.906	0.8931669	oprL	Peptidoglycan associated lipoprotein OprL	PA0973	246	92.55
1677446	1.073	0.88156307	null	conserved hypothetical protein	PA0976	177	94.17
1677449	1.066	1.830253	null	conserved hypothetical protein	PA0983	160	92.41
1677452	0.815	-4.4444027	null	hypothetical protein	PA0977	1	92.91
1677453	1.105	0.7481714	null	hypothetical protein	PA0981	245	92.19
1677454	1.210	1.1138417	null	hypothetical protein	PA0988	174	93.52

1677456	0.934	0.5849445	null	hypothetical protein	PA0991	168	92.54
1677457	1.009	2.5145097	null	conserved hypothetical protein	PA0990	81	94.6
1677460	1.699	1.0968463	ogt	methylated-DNA--protein-cysteine	PA0995	15	92.26
1677463	1.009	1.9614284	cupC2	chaperone CupC2	PA0993	52	93.37
1677466	1.640	1.4242887	null	conserved hypothetical protein	PA0979	55	92.52
1677467	0.688	2.4969738	cupC3	usher CupC3	PA0994	56	92.13
1677470	1.255	1.0155308	null	pyocin S5	PA0985	271	92.34
1677473	1.154	0.91087943	null	conserved hypothetical protein	PA0978	23	92.26
1677477	1.071	0.5973474	null	conserved hypothetical protein	PA0986	215	92.01
1677478	2.579	-0.05137843	null	probable aldehyde dehydrogenase	PA0747	1041	94.64
1677481	0.095	0.06369672	pqsC	Homologous to beta-keto-acyl-acyl-carrier	PA0998	36	92.5
1677484	0.171	0.12029295	pqsE	Quinolone signal response protein	PA1000	8	92.73
1677487	1.069	0.65408504	cupC1	fimbrial subunit CupC1	PA0992	90	92.94
1677490	1.078	-0.15517113	null	hypothetical protein	PA0989	82	92.92
1677493	4.664	1.3807693	null	conserved hypothetical protein	PA0987	732	93.1
1677496	0.717	0.253223	null	conserved hypothetical protein	PA1006	42	92.34
1677498	0.114	0.06302825	pqsA	probable coenzyme A ligase	PA0996	51	92.31
1677501	0.155	0.11143477	phnB	anthranilate synthase component II	PA1002	308	94.07
1677504	0.834	0.60556597	mvfR	Transcriptional regulator	PA1003	30	92.24
1677507	0.846	0.7328257	dapA	dihydrodipicolinate synthase	PA1010	33	94.21
1677510	0.145	0.059638113	pqsB	Homologous to beta-keto-acyl-acyl-carrier	PA0997	270	94.47
1677513	0.634	0.6100217	null	conserved hypothetical protein	PA1012	144	93.52
1677516	0.057	0.03874857	phnA	anthranilate synthase component I	PA1001	6	93.94
1677519	0.907	0.7848731	null	hypothetical protein	PA1011	103	95.26
1677522	1.451	0.6195904	null	probable glycosyl transferase	PA1014	1	96.41
1677525	1.301	0.95305556	null	conserved hypothetical protein	PA1005	75	94.12
1677528	1.562	-0.33683407	null	probable transcriptional regulator	PA1015	5	92.75
1677531	1.099	0.98380244	purC	phosphoribosylaminoimidazole-succinocarboxamide	PA1013	237	92.42
1677534	0.739	1.3176924	null	hypothetical protein	PA1018	11	92.43
1677537	1.219	1.0029699	bcp	bacterioferritin comigratory protein	PA1008	45	95.3
1677540	1.269	0.90434074	mucK	cis,cis-muconate transporter MucK	PA1019	258	93.56
1677543	1.141	0.97247714	null	hypothetical protein	PA1016	158	97.43
1677546	0.862	-0.3375	null	probable enoyl-CoA hydratase/isomerase	PA1021	410	96.3
1677549	1.009	1.9043767	null	probable short-chain dehydrogenase	PA1023	3	92.46
1677552	1.640	1.0024183	null	probable acyl-CoA dehydrogenase	PA1020	1	94.91
1677555	1.013	1.9140285	null	probable acyl-CoA dehydrogenase	PA1022	17	92.01
1677558	1.127	0.97382975	null	hypothetical protein	PA1029	232	92.63
1677560	1.052	0.67714286	null	probable oxidoreductase	PA1028	2	93.11
1677563	2.260	0.55969924	null	hypothetical protein	PA1026	74	94.79
1677566	1.009	0.43389624	null	probable porin	PA1025	271	92.62
1677569	0.862	0.7689646	null	hypothetical protein	PA1030	174	92.21
1677572	0.525	0.45831418	null	hypothetical protein	PA1034	1	92.46
1677575	0.828	0.46482745	null	conserved hypothetical protein	PA1031	37	92.81
1677579	0.951	0.6743876	null	hypothetical protein	PA1036	122	96.05
1677581	1.124	0.936629	nadA	quinolinate synthetase A	PA1004	126	92.36
1677584	0.815	0.6222163	pauA	pimeloyl-CoA synthetase	PA1017	8	92.65
1677587	1.068	0.73699766	null	probable glutathione S-transferase	PA1033	218	92.13
1677590	0.893	0.78173304	null	hypothetical protein	PA1035	102	92.53
1677593	1.248	0.99848783	null	probable penicillin amidase	PA1032	1	95.8
1677596	1.116	0.84247625	null	probable aldehyde dehydrogenase	PA1027	402	92.17
1677599	1.009	2.0856838	null	probable outer membrane protein precursor	PA1041	42	94.87
1677602	0.802	-1.7616513	null	hypothetical protein	PA1024	187	94.68
1677605	0.978	0.7997009	null	hypothetical protein	PA1038	193	92.77
1677608	0.982	0.8905568	tolB	TolB protein	PA0972	276	92.24
1677611	0.867	-0.21497393	null	hypothetical protein	PA1043	87	94.57
1677614	0.759	0.500825	null	conserved hypothetical protein	PA1037	127	96.48
1677617	0.977	0.8180497	null	hypothetical protein	PA1009	44	96.96
1677620	0.468	-0.21778543	null	hypothetical protein	PA1040	107	94.47
1677623	0.627	0.3989729	null	conserved hypothetical protein	PA1042	208	92.65
1677625	0.819	0.48360318	null	hypothetical protein	PA1044	245	92.38
1677628	1.139	0.82751787	pdxH	pyridoxine 5'-phosphate oxidase	PA1049	216	92.01
1677631	0.746	0.53394663	null	probable esterase	PA1047	1	95.29
1677634	1.510	1.3540169	null	conserved hypothetical protein	PA1053	57	94.3
1677637	1.026	0.8927019	null	conserved hypothetical protein	PA1055	81	92.02
1677641	0.715	0.57237184	null	conserved hypothetical protein	PA1050	504	97.98
1677643	0.928	0.7356349	null	probable NADH dehydrogenase	PA1056	318	92.97
1677646	1.515	1.0391095	null	conserved hypothetical protein	PA1052	14	92.64
1677649	1.285	1.0256389	null	probable transporter	PA1051	31	92.45
1677652	1.123	0.98170537	null	conserved hypothetical protein	PA1058	103	94.8
1677654	0.886	0.77019423	null	conserved hypothetical protein	PA1059	20	93.82
1677657	0.878	-0.86982095	null	hypothetical protein	PA1063	26	95.9
1677658	1.181	1.0461164	null	conserved hypothetical protein	PA1061	105	92.36
1677661	0.926	0.8002666	null	hypothetical protein	PA1060	42	95.12
1677664	0.878	0.54736936	null	hypothetical protein	PA1064	46	95.97
1677667	1.109	0.74692714	null	conserved hypothetical protein	PA1065	223	92.95
1677669	0.610	2.8455203	null	hypothetical protein	PA1045	1	92.38
1677672	0.862	1.5415685	null	probable short-chain dehydrogenase	PA1066	120	95.54
1677675	0.921	0.8363085	null	probable NADH dehydrogenase	PA1054	218	94.32
1677678	1.272	2.6855974	null	probable transcriptional regulator	PA1067	68	93.36
1677681	0.897	0.7856535	null	probable outer membrane protein precursor	PA1048	9	92.18
1677684	1.280	0.6961206	null	hypothetical protein	PA1046	101	92.39
1677687	1.042	2.9912193	null	conserved hypothetical protein	PA1039	34	94.65
1677690	0.736	0.48854512	braD	branched-chain amino acid transport protein	PA1073	228	92.49
1677693	1.152	1.1687446	braG	branched-chain amino acid transport protein	PA1070	192	94.98
1677696	0.971	0.84105414	null	conserved hypothetical protein	PA1057	20	92.33
1677699	1.178	0.9823189	braF	branched-chain amino acid transport protein	PA1071	304	96.93
1677702	0.929	0.61800253	null	hypothetical protein	PA1075	194	93.71
1677704	1.303	1.1242573	flgB	flagellar basal-body rod protein FlgB	PA1077	255	94.26
1677707	1.192	0.9671899	flgC	flagellar basal-body rod protein FlgC	PA1078	84	94.06
1677710	0.880	0.6312467	null	hypothetical protein	PA1069	140	93.93
1677713	0.703	0.5367861	null	probable heat shock protein (hsp90 family)	PA1068	27	92.61
1677716	0.650	0.45746046	null	hypothetical protein	PA1062	184	94.16
1677719	1.076	0.94604385	null	hypothetical protein	PA1076	245	92.62
1677722	1.467	1.2153343	flgG	flagellar basal-body rod protein FlgG	PA1082	108	96.36
1677725	0.162	0.11611308	pqsD	3-oxoacyl-[acyl-carrier-protein] synthase III	PA0999	214	92.56
1677728	1.210	1.0106214	flgH	flagellar L-ring protein precursor FlgH	PA1083	137	92.82
1677731	1.098	0.88237685	flgF	flagellar basal-body rod protein FlgF	PA1081	35	93.6
1677734	1.471	1.2987093	flgE	flagellar hook protein FlgE	PA1080	286	92.5
1677737	0.948	0.7786814	null	hypothetical protein	PA1088	76	92.6
1677740	0.858	0.5889887	braE	branched-chain amino acid transport protein	PA1072	27	92.11
1677743	0.924	0.74335515	null	hypothetical protein	PA1090	33	93.85
1677746	0.954	0.79938436	flgI	flagellar P-ring protein precursor FlgI	PA1084	155	93.46
1677749	0.960	0.8905522	null	hypothetical protein	PA1095	31	93.1
1677751	0.973	0.80462134	flgJ	flagellar protein FlgJ	PA1085	30	94.2
1677754	0.850	0.8499076	null	hypothetical protein	PA1093	243	92.81
1677757	0.934	0.8180393	null	conserved hypothetical protein	PA1089	1	93.24
1677760	1.509	1.7149798	fliC	flagellin type B	PA1092	91	92.54
1677763	1.020	0.99601287	null	hypothetical protein	PA1096	61	92.76
1677766	0.977	0.8582859	fliD	flagellar capping protein FliD	PA1094	96	93.03

1677769	0.909	0.6572296	null	probable flagellar assembly protein	PA1103	160	96.68
1677772	1.235	1.0457202	flgD	flagellar basal-body rod modification protein	PA1079	142	94.05
1677775	1.049	0.89810497	fliG	flagellar motor switch protein FliG	PA1102	361	94.18
1677778	0.916	0.7820188	fleR	two-component response regulator	PA1099	491	92.69
1677781	1.018	0.8610121	fleS	two-component sensor	PA1098	331	95.31
1677784	1.306	1.1051495	flgK	flagellar hook-associated protein 1 FlgK	PA1086	44	92.82
1677787	0.928	0.693937	fleQ	transcriptional regulator FleQ	PA1097	77	92.31
1677790	1.763	0.3281578	null	hypothetical protein	PA1106	74	93.92
1677793	1.082	0.89392793	fliE	flagellar hook-basal body complex protein FliE	PA1100	118	92.26
1677795	1.258	0.9291666	null	hypothetical protein	PA1111	1	92.36
1677799	0.597	0.47966185	fliJ	flagellar protein FliJ	PA1105	200	92.84
1677801	1.217	1.0566965	null	conserved hypothetical protein	PA1107	30	94.87
1677804	0.632	1.4259377	null	probable transcriptional regulator	PA1109	113	94.03
1677807	2.009	0.7335471	null	hypothetical protein	PA1110	101	92.33
1677810	0.922	0.75528955	null	probable major facilitator superfamily (MFS)	PA1108	33	96.06
1677813	1.075	0.83503896	fliF	Flagella M-ring outer membrane protein	PA1101	166	95.43
1677816	1.153	0.8701352	null	hypothetical protein	PA1115	81	95.04
1677819	0.715	0.4420374	null	hypothetical protein	PA1091	43	95.73
1677822	1.357	1.3199726	null	hypothetical protein	PA1114	282	92.32
1677824	0.924	-0.10628971	null	hypothetical protein	PA1117	6	93.05
1677827	0.704	0.4916277	fliI	flagellum-specific ATP synthase FliI	PA1104	422	96.21
1677830	1.183	0.82648087	null	hypothetical protein	PA1118	85	94.55
1677833	1.170	1.0073918	null	probable outer membrane protein precursor	PA1119	146	94.14
1677836	0.987	0.73728406	null	probable ATP-binding/permease fusion ABC	PA1113	151	93.84
1677839	0.977	0.7738163	null	hypothetical protein	PA1116	133	92
1677842	1.602	1.3446242	null	hypothetical protein	PA1123	33	94.1
1677845	0.807	0.60421306	null	probable oxidoreductase	PA1127	5	92.36
1677848	1.011	0.6217878	null	conserved hypothetical protein	PA1120	155	92.14
1677851	1.019	0.6595321	null	conserved hypothetical protein	PA1121	1	95.48
1677854	0.821	0.48720646	null	probable cobalamin biosynthetic protein	PA1125	173	96.98
1677857	0.814	0.50099456	null	conserved hypothetical protein	PA1112	1	95.35
1677860	0.978	0.67943615	null	hypothetical protein	PA1132	54	95.7
1677863	1.052	0.79025114	null	probable peptide deformylase	PA1122	265	93.39
1677866	0.782	0.56077033	dgt	deoxyguanosinetriphosphate triphosphohydrolase	PA1124	83	92.25
1677869	1.000	0.61426187	rhlC	rhamnosyltransferase 2	PA1130	83	94.42
1677872	1.060	0.3376009	null	hypothetical protein	PA1133	50	94.83
1677874	0.722	0.43445572	null	probable fosfomycin resistance protein	PA1129	99	93.6
1677877	0.698	0.5754693	null	hypothetical protein	PA1126	56	93.18
1677880	0.848	0.20646301	null	probable transcriptional regulator	PA1138	203	95.42
1677883	1.045	0.8026614	null	conserved hypothetical protein	PA1135	41	94.47
1677886	0.762	0.6694541	null	probable major facilitator superfamily (MFS)	PA1131	53	92.88
1677889	1.009	1.14873	null	hypothetical protein	PA1139	1	94.17
1677892	1.092	0.052570533	null	conserved hypothetical protein	PA1140	51	94.47
1677895	1.028	0.81471795	null	probable transcriptional regulator	PA1142	422	92.63
1677898	1.009	3.3894699	null	probable transcriptional regulator	PA1141	130	95.17
1677901	0.331	-0.05231328	null	probable oxidoreductase	PA1137	615	92.91
1677904	1.016	0.8915903	null	probable major facilitator superfamily (MFS)	PA1144	136	93.54
1677907	0.915	0.32206637	null	probable transcriptional regulator	PA1145	262	94.81
1677910	2.429	1.6779732	null	hypothetical protein	PA1149	238	93.92
1677913	1.374	0.9885561	null	probable amino acid permease	PA1147	75	93.18
1677916	1.009	1.4218841	null	hypothetical protein	PA1143	149	96.03
1677919	1.471	1.0955533	pys2	pyocin S2	PA1150	717	92.02
1677922	0.998	0.7286706	null	hypothetical protein	PA1152	11	95.21
1677923	1.361	1.018789	null	hypothetical protein	PA1153	62	92.77
1677926	1.009	-0.9116667	null	probable iron-containing alcohol dehydrogenase	PA1146	175	92.87
1677929	1.009	4.2200856	null	hypothetical protein	PA1134	50	95.54
1677931	0.910	0.53790474	null	probable two-component response regulator	PA1157	453	95.6
1677934	1.715	0.95522594	null	conserved hypothetical protein	PA1154	253	95.27
1677937	0.165	-0.4599139	null	probable transcriptional regulator	PA1136	153	92.61
1677940	1.129	1.0938685	nrdA	ribonucleoside reductase, large chain	PA1156	108	92.23
1677943	0.759	-2.518601	null	probable transcriptional regulator	PA1128	24	94.35
1677946	1.484	-3.0680919	null	probable two-component sensor	PA1158	624	93
1677949	1.210	1.0932108	nrdB	ribonucleoside reductase, small chain	PA1155	246	93.87
1677952	0.990	1.006135	pcpS	PcpS	PA1165	2	96.9
1677955	1.386	0.97103643	null	hypothetical protein	PA1167	1	93.56
1677958	0.899	0.6410046	rrmA	rRNA methyltransferase	PA1161	248	94.55
1677961	1.320	1.1982851	flgL	flagellar hook-associated protein type 3 FlgL	PA1087	29	92.42
1677964	1.199	1.0994215	braC	branched-chain amino acid transport protein	PA1074	30	95.16
1677967	0.905	0.61544394	dapE	succinyl-diaminopimelate desuccinylase	PA1162	142	96.13
1677970	1.056	0.6452286	null	hypothetical protein	PA1168	95	94.21
1677972	0.584	0.37734908	null	conserved hypothetical protein	PA1170	16	94.39
1677975	0.663	0.48807588	null	conserved hypothetical protein	PA1164	172	92.34
1677978	1.009	-2.162228	toxA	exotoxin A precursor	PA1148	75	93.92
1677981	1.880	1.3000749	null	probable lipoxygenase	PA1169	3	92.38
1677984	0.784	0.59371406	null	hypothetical protein	PA1160	172	95.74
1677987	1.204	1.0158473	napC	cytochrome c-type protein NapC	PA1172	18	97.55
1677990	0.986	0.94000006	napF	ferredoxin protein NapF	PA1176	426	96.17
1677993	1.629	1.645519	oprH	PhoP/Q and low Mg2+ inducible outer membrane	PA1178	215	92.34
1677996	1.216	0.92172605	napE	periplasmic nitrate reductase protein NapE	PA1177	99	93.27
1677997	1.334	1.1751435	phoP	two-component response regulator PhoP	PA1179	97	93.09
1678000	0.997	0.6890403	null	probable transcriptional regulator	PA1182	146	92.88
1678003	1.149	0.958367	null	probable glucosyl transferase	PA1163	46	95.88
1678006	1.047	0.4091453	null	probable glutathione S-transferase	PA1185	68	94.69
1678009	1.008	0.95155466	null	probable transglycolase	PA1171	1	95.69
1678012	1.009	0.69102883	null	hypothetical protein	PA1186	168	94.8
1678015	1.383	0.96137476	napD	NapD protein of periplasmic nitrate reductase	PA1175	11	94.96
1678018	0.599	-0.21371794	null	probable transcriptional regulator	PA1184	26	94.27
1678021	0.712	0.5279101	napA	periplasmic nitrate reductase protein NapA	PA1174	293	93.13
1678024	0.699	3.0272808	null	probable acyl-CoA dehydrogenase	PA1187	32	92.75
1678027	1.153	0.8892738	null	hypothetical protein	PA1188	160	94.5
1678030	0.976	0.6576358	null	conserved hypothetical protein	PA1189	346	94.87
1678033	0.383	0.6607693	napB	cytochrome c-type protein NapB precursor	PA1173	152	92.97
1678035	1.556	1.0273215	null	conserved hypothetical protein	PA1190	18	92.68
1678038	1.002	0.7222346	null	hypothetical protein	PA1193	11	92.28
1678041	0.888	0.8364247	null	conserved hypothetical protein	PA1198	131	93.41
1678044	0.847	0.20548633	null	hypothetical protein	PA1191	483	97.1
1678047	0.709	0.49306017	dctA	C4-dicarboxylate transport protein	PA1183	312	93.79
1678050	1.009	1.1831809	null	hypothetical protein	PA1195	415	92.61
1678053	0.790	0.6667736	null	probable lipoprotein	PA1199	442	92.72
1678056	1.016	0.81189764	null	conserved hypothetical protein	PA1192	80	92.38
1678059	0.802	0.6589097	null	conserved hypothetical protein	PA1200	20	92.57
1678062	1.285	0.5185134	null	hypothetical protein	PA1166	44	96.3
1678065	1.413	0.46665898	null	hypothetical protein	PA1203	218	92.54
1678068	1.114	0.90248126	null	probable transcriptional regulator	PA1196	34	95.26
1678071	1.291	1.2676222	null	probable hydrolase	PA1202	62	96.38
1678074	0.742	0.56623155	null	conserved hypothetical protein	PA1204	414	92.78
1678077	1.034	0.7539312	null	hypothetical protein	PA1206	55	95.82
1678080	0.505	0.20769231	null	conserved hypothetical protein	PA1007	581	93.55
1678083	1.250	0.74633884	null	conserved hypothetical protein	PA1210	1	93.11

1678086	1.348	0.8855836	null	conserved hypothetical protein	PA1208	117	97.74
1678091	0.986	-0.55804	null	hypothetical protein	PA1211	276	94.69
1678092	1.401	-1.5141277	null	probable amino acid permease	PA1194	157	92.71
1678095	0.992	0.8023571	null	conserved hypothetical protein	PA1205	1	92.13
1678098	1.508	0.89713657	null	probable major facilitator superfamily (MFS)	PA1212	611	96.24
1678101	1.118	0.9261131	null	hypothetical protein	PA1209	9	94.54
1678104	1.184	3.5864704	null	hypothetical protein	PA1197	87	94.56
1678107	1.102	0.75715435	null	hypothetical protein	PA1215	730	95.69
1678110	2.009	5.038141	null	hypothetical protein	PA1216	37	92.48
1678113	0.969	0.49989206	null	hypothetical protein	PA1219	1	92.79
1678116	1.353	1.0166423	null	probable transcriptional regulator	PA1201	9	92.49
1678119	1.148	0.8624547	null	probable NAD(P)H dehydrogenase	PA1224	2	92.63
1678122	0.917	0.08756854	null	probable membrane-bound lytic murein	PA1222	106	95.94
1678125	0.717	0.37635675	null	probable transcriptional regulator	PA1226	392	92.37
1678128	1.326	0.9572973	null	hypothetical protein	PA1227	226	93.32
1678132	1.102	0.83075887	null	hypothetical protein	PA1228	274	94.95
1678133	1.066	0.72200847	null	probable transcriptional regulator	PA1223	442	95.54
1678136	1.009	3.6632774	null	probable 2-isopropylmalate synthase	PA1217	323	94.29
1678139	1.239	0.8757418	null	probable NAD(P)H dehydrogenase	PA1225	285	92.22
1678142	2.570	2.5833154	null	hypothetical protein	PA1230	1	92.64
1678144	1.064	0.53003275	null	hypothetical protein	PA1218	256	92.97
1678147	1.357	0.863449	null	probable transcriptional regulator	PA1229	151	97.6
1678150	1.039	0.7401786	null	hypothetical protein	PA1220	942	96.44
1678153	1.009	-0.6166666	null	hypothetical protein	PA1233	69	93.54
1678156	0.295	0.01414423	null	hypothetical protein	PA1234	344	92.12
1678158	4.071	0.31335777	null	conserved hypothetical protein	PA1231	2	92.73
1678161	1.318	0.92173415	null	hypothetical protein	PA1214	6	93.46
1678164	1.009	5.1853848	null	probable major facilitator superfamily (MFS)	PA1236	61	96.8
1678167	1.163	1.102381	null	hypothetical protein	PA1239	251	94.59
1678170	1.059	0.86080873	null	probable transcriptional regulator	PA1235	1	93.38
1678175	0.948	1.2753737	null	probable enoyl-CoA hydratase/isomerase	PA1240	323	93.61
1678176	1.116	0.9740194	phoQ	two-component sensor PhoQ	PA1180	249	92.49
1678179	1.133	1.0037158	null	hypothetical protein	PA1242	3	97.55
1678182	0.506	-0.053480387	null	probable sensor/response regulator hybrid	PA1243	55	94.81
1678185	0.871	0.40062252	null	probable multidrug resistance efflux pump	PA1237	131	96.72
1678188	1.139	0.81014615	null	probable transcriptional regulator	PA1241	103	92.66
1678191	0.998	0.31506124	null	probable outer membrane component of multidrug	PA1238	155	94.48
1678194	1.202	0.95834315	kefB	glutathione-regulated potassium-efflux system	PA1207	7	92.27
1678197	0.882	0.73812443	null	conserved hypothetical protein	PA1181	7	97.13
1678200	0.625	0.34487882	aprI	alkaline proteinase inhibitor AprI	PA1250	70	92.37
1678202	1.533	1.119868	null	hypothetical protein	PA1244	180	93.71
1678204	0.854	-0.5283404	aprE	alkaline protease secretion protein AprE	PA1247	265	96.14
1678207	1.078	0.81666666	null	probable ATP-binding component of ABC	PA1256	34	92.35
1678210	1.009	1.5708333	aprA	alkaline metalloproteinase precursor	PA1249	138	95.96
1678213	1.019	3.5263157	null	probable dihydrodipicolinate synthetase	PA1254	1	93.82
1678216	1.009	0.6741177	aprF	Alkaline protease secretion outer membrane	PA1248	978	93.72
1678219	1.068	0.86045027	null	hypothetical protein	PA1232	111	92.92
1678222	0.593	0.00376457	null	hypothetical protein	PA1245	128	94.11
1678225	1.315	1.0195558	null	probable permease of ABC transporter	PA1257	20	96.33
1678228	1.183	0.97495097	null	probable transcriptional regulator	PA1261	114	94.78
1678231	1.200	0.908838	null	probable binding protein component of ABC	PA1260	2	92.47
1678234	1.115	0.9211823	null	probable permease of ABC transporter	PA1258	240	95.87
1678237	1.274	0.84731466	null	hypothetical protein	PA1263	36	92.61
1678240	2.153	3.5616841	null	hypothetical protein	PA1265	56	93.91
1678243	1.032	0.6744685	null	probable L-malate dehydrogenase	PA1252	112	94.86
1678246	1.093	0.6666293	null	hypothetical protein	PA1255	253	95.38
1678249	1.291	1.0168545	null	hypothetical protein	PA1267	634	93.52
1678253	1.133	0.82890034	null	probable transcriptional regulator	PA1264	15	92.5
1678255	1.099	0.5447001	null	hypothetical protein	PA1268	185	96.5
1678258	0.507	-1.1542825	null	probable transcriptional regulator	PA1269	122	92.81
1678261	1.262	0.8136362	null	probable oxidoreductase	PA1266	273	96.54
1678264	0.922	0.5722208	cobB	cobyrinic acid a,c-diamide synthase	PA1273	424	96.53
1678267	1.039	0.774854	cobO	cob(I)alamin adenosyltransferase	PA1272	169	92.33
1678270	0.925	0.66522765	null	probable tonB-dependent receptor	PA1271	561	93.09
1678273	1.406	1.1755478	null	hypothetical protein	PA1259	930	92.18
1678276	1.174	0.93845195	null	conserved hypothetical protein	PA1274	153	93.84
1678279	1.015	0.801987	cobP	cobinamide kinase	PA1278	258	94.32
1678281	0.743	0.4670557	cobC	cobalamin biosynthetic protein CobC	PA1276	532	95.85
1678284	1.172	1.146904	cobD	cobalamin biosynthetic protein CobD	PA1275	508	92.84
1678287	1.035	0.48571435	null	probable major facilitator superfamily (MFS)	PA1262	129	93.45
1678292	0.892	0.65698177	cobV	cobalamin (5'-phosphate) synthase	PA1281	600	96.54
1678293	0.831	4.184058	null	hypothetical protein	PA1280	214	92.41
1678296	1.149	0.70367974	cobU	nicotinate-nucleotide--dimethylbenzimidazole	PA1279	865	97.42
1678299	1.074	8.230502	null	probable transcriptional regulator	PA1285	34	92.72
1678301	0.831	-0.09144421	null	probable transcriptional regulator	PA1283	159	93.38
1678304	2.053	0.9586687	null	probable major facilitator superfamily (MFS)	PA1282	122	93.47
1678307	0.881	1.0456657	null	probable outer membrane protein precursor	PA1288	23	92.23
1678310	1.007	0.4154614	null	probable transcriptional regulator	PA1290	181	93.62
1678313	1.009	0.40194613	cobQ	cobyric acid synthase	PA1277	311	95.96
1678316	0.585	0.2176394	null	probable glutathione peroxidase	PA1287	241	92.23
1678319	1.741	1.251136	null	hypothetical protein	PA1291	139	96.54
1678322	1.036	0.7944666	null	probable 3-mercaptopyruvate sulfurtransferase	PA1292	74	96.12
1678325	1.111	1.6194872	null	probable chemotaxis transducer	PA1251	242	94.08
1678328	1.207	0.48031792	null	hypothetical protein	PA1289	8	92.92
1678331	1.009	2.0074825	null	hypothetical protein	PA1213	237	95.57
1678334	0.700	0.40018702	null	conserved hypothetical protein	PA1295	162	93.36
1678337	0.997	0.7426134	null	conserved hypothetical protein	PA1298	226	93.59
1678338	0.937	0.7746705	null	probable 2-hydroxyacid dehydrogenase	PA1296	2	92.62
1678341	0.747	-0.043213032	rnd	ribonuclease D	PA1294	1	94.11
1678344	0.815	0.44467655	null	hypothetical protein	PA1293	58	96.63
1678347	0.714	0.47674903	null	probable sigma-70 factor, ECF subfamily	PA1300	1	92.48
1678350	1.074	1.0785689	null	probable heme utilization protein precursor	PA1302	161	97.55
1678353	1.056	0.6747216	null	hypothetical protein	PA1270	140	94.22
1678356	1.013	0.2095004	null	probable transmembrane sensor	PA1301	24	92.41
1678359	1.009	3.0571427	null	probable semialdehyde dehydrogenase	PA1253	999	92.71
1678362	1.096	1.0016434	null	hypothetical protein	PA1305	103	92.84
1678365	0.819	0.70869696	null	probable HIT family protein	PA1306	3	92.24
1678368	1.202	2.7943318	null	probable signal peptidase	PA1303	88	93.7
1678371	0.804	0.64423853	null	probable metal transporter	PA1297	3	97.91
1678374	1.059	0.7443765	null	conserved hypothetical protein	PA1307	95	96.79
1678377	1.009	1.3022323	phnX	2-phosphonoacetaldehyde hydrolase	PA1311	71	93.38
1678380	1.574	1.1867436	phnW	2-aminoethylphosphonate:pyruvate	PA1310	36	95.83
1678383	0.309	-0.03934797	null	hypothetical protein	PA1308	50	95.72
1678386	0.751	2.643046	null	probable transcriptional regulator	PA1312	1	93.58
1678389	0.946	0.5352146	null	probable transcriptional regulator	PA1315	80	94.96
1678392	1.004	0.8477694	null	hypothetical protein	PA1314	1	94.2
1678395	0.986	0.809714	null	hypothetical protein	PA1221	30	92.08
1678398	1.358	-3.6763802	cyoA	cytochrome o ubiquinol oxidase subunit II	PA1317	9	94.44
1678401	1.753	-0.93923205	cyoC	cytochrome o ubiquinol oxidase subunit III	PA1319	44	93.72

1678404	0.770	0.23051128	cyoD	cytochrome o ubiquinol oxidase subunit IV	PA1320	198	92.16
1678407	0.781	1.0847766	null	probable major facilitator superfamily (MFS)	PA1316	25	92.62
1678410	1.628	1.3876548	null	hypothetical protein	PA1323	237	92.7
1678413	1.009	2.5272727	null	probable acyl-CoA dehydrogenase	PA1284	121	93.7
1678416	1.573	1.3691335	null	hypothetical protein	PA1324	207	92
1678419	0.862	1.7358773	null	conserved hypothetical protein	PA1325	164	97.62
1678421	0.666	0.29395586	cyoB	cytochrome o ubiquinol oxidase subunit I	PA1318	1	92.42
1678424	1.235	0.99430704	null	conserved hypothetical protein	PA1329	1	92.62
1678426	1.112	0.880301	null	probable major facilitator superfamily (MFS)	PA1286	1	95.82
1678429	0.743	0.44814315	null	conserved hypothetical protein	PA1299	1	93.12
1678432	1.502	1.0165267	null	probable major facilitator superfamily (MFS)	PA1313	736	95.5
1678435	1.146	0.5064695	null	probable transcriptional regulator	PA1328	195	94.33
1678438	1.364	0.8684505	ilvA2	threonine dehydratase, biosynthetic	PA1326	1314	93.55
1678441	1.511	1.3025924	null	hypothetical protein	PA1333	76	93.23
1678443	1.953	1.6645769	null	hypothetical protein	PA1332	1	94
1678446	1.082	-0.9901298	cyoE	cytochrome o ubiquinol oxidase protein CyoE	PA1321	206	97.75
1678449	1.392	-2.3026996	null	probable short-chain dehydrogenase	PA1330	508	93.82
1678452	0.376	1.5252154	null	conserved hypothetical protein	PA1331	288	94
1678455	1.182	0.6010317	null	probable transcriptional regulator	PA1309	110	95.26
1678458	0.759	-0.24404939	null	probable permease of ABC transporter	PA1340	30	92.62
1678461	0.989	0.7372605	null	probable oligopeptidase	PA1304	649	93.76
1678464	1.302	1.079984	null	probable two-component sensor	PA1336	9	96.55
1678467	0.887	-1.2323273	null	probable protease	PA1327	30	92.49
1678470	0.729	0.50807834	null	probable permease of ABC transporter	PA1341	17	93.62
1678473	1.778	1.1157006	null	hypothetical protein	PA1343	71	92.35
1678476	1.744	0.485291	null	probable transcriptional regulator	PA1347	155	93.51
1678479	1.168	0.8375635	null	probable short-chain dehydrogenase	PA1344	99	96.26
1678482	1.009	2.463492	null	probable TonB-dependent receptor	PA1322	502	95.09
1678486	1.609	0.6107958	null	conserved hypothetical protein	PA1349	216	93.2
1678487	1.407	0.78235006	null	hypothetical protein	PA1348	56	92.36
1678490	1.076	0.44960037	null	hypothetical protein	PA1346	91	92.59
1678493	1.555	0.24349944	null	hypothetical protein	PA1350	423	92.58
1678496	2.478	1.403396	null	hypothetical protein	PA1353	125	92.91
1678499	1.009	1.1217476	null	hypothetical protein	PA1354	259	92.41
1678502	1.009	2.3896968	null	hypothetical protein	PA1355	75	93.19
1678504	0.879	0.8547143	null	hypothetical protein	PA1345	32	97.92
1678507	1.009	0.2849074	null	probable oxidoreductase	PA1334	842	94.28
1678510	0.712	0.49168903	null	probable two-component response regulator	PA1335	922	92.44
1678513	1.111	0.7472583	null	probable sigma-70 factor, ECF subfamily	PA1351	284	94.94
1678516	3.301	0.8206912	null	hypothetical protein	PA1356	281	93.3
1678519	1.134	1.021649	null	probable binding protein component of ABC	PA1342	30	96.26
1678522	0.923	6.09418	null	probable transcriptional regulator	PA1359	432	96.19
1678525	1.075	0.78048015	null	hypothetical protein	PA1358	46	92.48
1678528	0.707	-0.20992509	null	conserved hypothetical protein	PA1357	419	94.28
1678531	1.021	1.0831589	null	probable sigma-70 factor, ECF subfamily	PA1363	85	96.56
1678534	1.009	1.5825169	null	conserved hypothetical protein	PA1352	49	94.71
1678538	1.009	1.1155068	null	hypothetical protein	PA1362	201	92.66
1678539	0.697	0.5495583	null	hypothetical protein	PA1369	132	93.05
1678540	0.878	0	null	hypothetical protein	PA1367	116	93.47
1678543	1.009	0.27193627	null	hypothetical protein	PA1368	31	92.5
1678546	1.005	0.84707004	null	hypothetical protein	PA1366	31	93.36
1678549	0.718	0.24352796	null	probable siderophore receptor	PA1365	7	94.15
1678552	0.868	0.8156298	null	probable transporter	PA1361	1	97.98
1678555	0.539	0.28557515	null	hypothetical protein	PA1370	361	92.02
1678558	1.057	1.2612879	null	hypothetical protein	PA1374	54	93.7
1678561	0.790	0.75168335	ggt	gamma-glutamyltranspeptidase precursor	PA1338	675	92.68
1678564	1.058	0.94601935	null	hypothetical protein	PA1372	648	92.45
1678567	0.582	0.30651224	null	hypothetical protein	PA1378	156	93.29
1678570	1.414	1.1840866	null	hypothetical protein	PA1381	71	93.21
1678573	0.869	0.47862822	aceK	isocitrate dehydrogenase kinase/phosphatase	PA1376	34	93.2
1678576	1.227	0.8028401	pdxB	erythronate-4-phosphate dehydrogenase	PA1375	837	94.93
1678579	1.009	2.179762	galE	UDP-glucose 4-epimerase	PA1384	300	92.35
1678582	1.009	1.317417	null	probable transcriptional regulator	PA1380	33	95.92
1678585	0.484	-0.56065387	null	probable type II secretion system protein	PA1382	1	94.65
1678588	1.009	1.2369444	null	probable glycosyl transferase	PA1385	679	93.13
1678590	1.190	0.50130093	fabF2	3-oxoacyl-acyl carrier protein synthase II	PA1373	210	92.27
1678593	1.133	1.3366826	null	hypothetical protein	PA1383	14	92.39
1678596	1.009	1.5557345	null	probable ATP-binding component of ABC	PA1386	193	92.53
1678599	1.009	1.160733	null	probable short-chain dehydrogenase	PA1379	249	92.04
1678602	0.959	1.3890152	null	hypothetical protein	PA1388	81	93.07
1678605	3.429	-0.27321428	null	probable glycosyl transferase	PA1389	1	93.77
1678608	1.009	1.552417	null	probable glycosyl transferase	PA1390	18	92.72
1678611	1.065	3.366013	cysC	adenosine 5'-phosphosulfate (APS) kinase	PA1393	89	92.17
1678614	1.009	0.9438034	null	hypothetical protein	PA1392	94	95.36
1678617	0.469	0.18773463	null	conserved hypothetical protein	PA1377	401	92.92
1678620	0.566	1.3773155	null	probable glycosyl transferase	PA1391	39	93.71
1678623	0.792	1.0623738	null	hypothetical protein	PA1395	195	92.06
1678626	1.430	6.9003325	null	hypothetical protein	PA1387	277	92.26
1678629	0.711	0.2570725	null	probable two-component response regulator	PA1397	28	92.38
1678632	0.890	0.23583718	null	hypothetical protein	PA1398	196	92.25
1678635	1.082	0.9757055	null	hypothetical protein	PA1404	3	92.7
1678637	1.093	0.75268376	null	hypothetical protein	PA1402	128	92.14
1678640	0.803	0.19591992	null	hypothetical protein	PA1401	76	92.53
1678642	0.509	1.0267346	null	probable helicase	PA1405	113	93.34
1678645	0.831	-0.3644445	null	hypothetical protein	PA1406	258	92.6
1678648	1.009	-0.75324667	null	probable transcriptional regulator	PA1403	43	95.04
1678651	0.820	1.3050593	null	probable ATP-binding component of ABC	PA1339	21	92.07
1678654	0.892	0.25796595	null	hypothetical protein	PA1407	321	92.64
1678657	1.034	0.65767336	null	probable transmembrane sensor	PA1364	1	93.73
1678660	1.078	1.0685087	null	probable transcriptional regulator	PA1399	256	95.11
1678663	1.031	0.8912088	aphA	acetylpolyamine aminohydrolase	PA1409	942	93.09
1678666	1.267	0.7461608	aprD	alkaline protease secretion protein AprD	PA1246	833	93.16
1678669	1.336	0.7187006	null	hypothetical protein	PA1415	45	92.04
1678672	1.335	1.1220647	null	conserved hypothetical protein	PA1360	41	94.68
1678675	1.269	1.2549484	null	hypothetical protein	PA1414	161	95.75
1678676	1.272	1.3669231	null	probable transcriptional regulator	PA1413	628	93.7
1678679	1.042	0.89254236	gbuA	guanidinobutyrase	PA1421	132	97.15
1678682	1.067	0.77914464	null	conserved hypothetical protein	PA1416	1	92.15
1678685	0.699	-0.91428566	null	probable periplasmic	PA1410	212	94.38
1678688	1.009	0.6658996	null	probable two-component sensor	PA1396	32	94.36
1678691	1.111	1.3990349	gbuR	GbuR	PA1422	72	94.72
1678694	1.072	0.8462016	null	hypothetical protein	PA1424	32	97.26
1678697	1.009	-5.949662	null	probable chemotaxis transducer	PA1423	17	93.79
1678700	0.915	1.1528708	null	hypothetical protein	PA1411	33	92.06
1678703	0.792	0.5203866	null	hypothetical protein	PA1426	1	92.95
1678706	1.777	1.2947649	null	hypothetical protein	PA1427	211	92.94
1678709	1.009	0.85225296	null	conserved hypothetical protein	PA1428	25	94.07
1678712	0.640	0.593648	rsaL	regulatory protein RsaL	PA1431	3	92.44
1678714	1.083	0.9103991	lasR	transcriptional regulator LasR	PA1430	213	92.46

1678717	1.159	1.2384583	lasI	autoinducer synthesis protein LasI	PA1432	187	92.24
1678720	1.383	1.175967	null	probable transporter	PA1419	142	95.13
1678723	0.906	0.4437572	null	hypothetical protein	PA1408	4	95.71
1678726	0.905	0.62271726	null	hypothetical protein	PA1434	238	92.93
1678729	1.016	0.4352455	null	probable pyruvate carboxylase	PA1400	106	96.25
1678732	1.075	-0.82222223	null	probable Resistance-Nodulation-Cell Division	PA1435	194	92.36
1678735	0.613	0.29257375	null	hypothetical protein	PA1420	92	92.53
1678738	0.996	0.41709274	null	probable two-component response regulator	PA1437	17	94.48
1678741	1.086	0.7517645	null	probable two-component sensor	PA1438	83	92.42
1678744	0.764	0.6598961	null	conserved hypothetical protein	PA1439	284	97.09
1678747	0.446	0.06884993	null	probable sodium:solute symport protein	PA1418	363	92.18
1678750	1.082	0.9407873	null	conserved hypothetical protein	PA1442	381	92.48
1678753	0.762	0.50684077	fliM	flagellar motor switch protein FliM	PA1443	72	92.39
1678756	0.895	0.72601855	null	hypothetical protein	PA1440	40	92.42
1678759	0.917	0.73711836	fliO	flagellar protein FliO	PA1445	325	96
1678761	1.361	1.0557501	ansB	glutaminase-asparaginase	PA1337	370	92.3
1678764	1.086	0.5662147	null	probable Resistance-Nodulation-Cell Division	PA1436	132	94.55
1678767	0.811	0.5525363	fliQ	flagellar biosynthetic protein FliQ	PA1447	205	92.04
1678770	0.984	0.82461643	fliP	flagellar biosynthetic protein FliP	PA1446	301	92.99
1678773	1.009	0.33482146	null	conserved hypothetical protein	PA1433	53	94.8
1678776	1.000	0.8128997	null	hypothetical protein	PA1441	1	95.71
1678779	1.009	1.5888889	null	probable ATP-binding component of ABC	PA1425	499	95.71
1678783	1.309	1.0374311	fliR	flagellar biosynthetic protein FliR	PA1448	432	92.3
1678785	1.029	0.82647896	cheZ	chemotaxis protein CheZ	PA1457	99	95.59
1678788	0.979	0.8060067	fleN	flagellar synthesis regulator FleN	PA1454	16	92.93
1678791	0.699	0.23127921	flhB	flagellar biosynthetic protein FlhB	PA1449	339	94.21
1678794	0.947	0.8415251	cheY	two-component response regulator CheY	PA1456	48	92.02
1678796	1.065	0.8749546	fliN	flagellar motor switch protein FliN	PA1444	289	92.58
1678799	0.731	0.3063218	null	probable cation-transporting P-type ATPase	PA1429	127	97.62
1678802	0.607	-0.18821391	flhF	flagellar biosynthesis protein FlhF	PA1453	15	93.91
1678805	0.928	0.79144347	motD	MotD	PA1461	11	95.3
1678808	0.574	0.23213187	null	conserved hypothetical protein	PA1451	336	93.48
1678811	0.684	0.5453898	null	hypothetical protein	PA1465	196	97.48
1678812	0.816	0.5409925	flhA	flagellar biosynthesis protein FlhA	PA1452	566	93.26
1678815	0.898	0.7596053	null	probable plasmid partitioning protein	PA1462	118	92.33
1678818	1.185	1.0515401	fliA	sigma factor FliA	PA1455	2	92.67
1678821	1.005	0.8945252	null	probable purine-binding chemotaxis protein	PA1464	371	92.34
1678824	0.876	0.7242508	motC	MotC	PA1460	600	92.35
1678827	0.908	0.79258996	null	probable methyltransferase	PA1459	268	94.18
1678830	1.215	2.0225868	null	hypothetical protein	PA1468	27	97.09
1678833	0.853	0.6832341	null	probable two-component sensor	PA1458	84	93.42
1678836	1.351	1.1218482	null	hypothetical protein	PA1471	54	95.62
1678839	1.065	3.5840225	null	hypothetical protein	PA1469	68	93.96
1678842	0.978	0.8062178	null	hypothetical protein	PA1463	14	95.02
1678845	0.960	0.6573128	null	hypothetical protein	PA1467	180	94.07
1678848	1.351	0.97966874	null	hypothetical protein	PA1473	188	94.24
1678851	1.198	0.31154615	null	hypothetical protein	PA1466	28	95.5
1678854	1.035	0.83019006	ccmA	heme exporter protein CcmA	PA1475	175	95.7
1678857	1.786	0.9322853	null	conserved hypothetical protein	PA1472	75	92.54
1678860	0.370	0.9861294	ccmB	heme exporter protein CcmB	PA1476	115	95.48
1678863	1.048	0.86843425	null	hypothetical protein	PA1478	34	94.28
1678864	0.873	0.7659647	ccmH	cytochrome C-type biogenesis protein CcmH	PA1482	251	92.98
1678867	0.891	0.63313854	null	hypothetical protein	PA1474	750	94.02
1678870	0.971	0.7789947	ccmG	cytochrome C biogenesis protein CcmG	PA1481	312	92.95
1678873	1.766	1.0298488	null	probable transcriptional regulator	PA1484	1	95.32
1678876	0.927	0.7552676	ccmE	cytochrome C-type biogenesis protein CcmE	PA1479	6	92.24
1678879	1.009	2.8322127	null	hypothetical protein	PA1486	86	95.86
1678882	0.668	0.3592957	null	probable transcriptional regulator	PA1490	69	94.28
1678885	0.965	-0.5282737	null	probable carbohydrate kinase	PA1487	75	92.98
1678888	0.594	-6.8359656	null	hypothetical protein	PA1488	571	95.69
1678891	0.859	0.65744835	null	hypothetical protein	PA1489	229	97.52
1678894	1.222	1.0168453	null	probable transporter	PA1491	162	93.3
1678898	1.802	1.6337662	null	hypothetical protein	PA1492	2	92.38
1678900	0.731	0.6353017	cysP	sulfate-binding protein of ABC transporter	PA1493	227	93.76
1678903	0.923	0.75799215	cycH	cytochrome c-type biogenesis protein	PA1483	446	94.34
1678906	0.918	0.609062	null	probable amino acid permease	PA1485	54	93.69
1678909	2.882	7.2960687	null	probable potassium channel	PA1496	21	92.96
1678912	0.895	0.40096387	null	hypothetical protein	PA1495	101	96.92
1678915	1.009	1.2321582	ccmC	heme exporter protein CcmC	PA1477	236	93.38
1678918	1.098	0.95159817	null	probable transporter	PA1497	2	93.47
1678921	1.009	1.03	pykF	pyruvate kinase I	PA1498	245	95.81
1678924	1.189	1.1181086	ccmF	cytochrome C-type biogenesis protein CcmF	PA1480	300	93.12
1678927	1.107	0.5474693	null	conserved hypothetical protein	PA1501	1	93.95
1678930	1.248	0.9114075	null	conserved hypothetical protein	PA1499	7	92.56
1678933	1.161	1.1635997	null	probable transcriptional regulator	PA1504	248	95.41
1678936	1.589	1.4084204	null	hypothetical protein	PA1503	228	94.55
1678939	1.012	0.8216039	moaA2	molybdopterin biosynthetic protein A2	PA1505	93	92.56
1678942	0.875	0.6335626	null	hypothetical protein	PA1508	178	92.32
1678943	0.591	-0.7756144	null	hypothetical protein	PA1506	231	92.03
1678945	1.074	0.1625	null	conserved hypothetical protein	PA1494	278	95.88
1678948	0.876	-1.471948	null	hypothetical protein	PA1509	495	93.58
1678951	0.764	0.6156765	null	probable transporter	PA1507	1	92.9
1678954	1.009	0.87707853	null	hypothetical protein	PA1510	59	92.83
1678957	1.126	0.8678028	null	probable oxidoreductase	PA1500	67	97.33
1678960	1.155	0.73621505	null	hypothetical protein	PA1513	33	92.68
1678963	1.028	0.88795996	gcl	glyoxylate carboligase	PA1502	238	95.65
1678968	0.792	0.6016675	hcpA	secreted protein Hcp	PA1512	402	92.55
1678969	1.299	0.85856307	null	conserved hypothetical protein	PA1514	326	94.12
1678972	0.849	0.27390394	null	hypothetical protein	PA1516	84	92.77
1678975	1.221	0.7916415	null	conserved hypothetical protein	PA1518	14	93.35
1678978	0.729	0.108910345	null	conserved hypothetical protein	PA1511	2256	92.02
1678981	0.856	0.46091574	alc	allantoicase	PA1515	744	92.52
1678984	1.321	0.9653797	xdhB	xanthine dehydrogenase	PA1523	45	97.56
1678987	0.993	0.8166544	null	probable transcriptional regulator	PA1520	60	92.02
1678990	1.129	0.787067	null	hypothetical protein	PA1522	33	92.54
1678993	0.981	0.7156007	null	probable transcriptional regulator	PA1526	202	94.32
1678996	1.380	2.346407	alkB2	alkane-1-monooxygenase 2	PA1525	71	95.25
1678999	1.042	-3.8390477	null	probable short-chain dehydrogenase	PA1470	198	94.76
1679002	1.191	1.3676465	null	hypothetical protein	PA1531	7	92.75
1679005	1.221	0.14315788	null	conserved hypothetical protein	PA1450	263	92.54
1679008	1.024	0.9007963	null	conserved hypothetical protein	PA1533	59	93.05
1679010	1.055	0.8763878	null	hypothetical protein	PA1530	216	94.38
1679013	0.619	0.50616854	null	conserved hypothetical protein	PA1517	31	96.2
1679016	1.750	1.3879544	xdhA	xanthine dehydrogenase	PA1524	2	92.18
1679019	0.544	0.3519283	null	probable acyl-CoA dehydrogenase	PA1535	30	92.34
1679022	0.838	0.6752614	null	conserved hypothetical protein	PA1527	15	92.36
1679025	1.086	0.8681137	null	probable transporter	PA1519	62	93.37
1679028	0.985	0.85741246	zipA	cell division protein ZipA	PA1528	184	93.47
1679031	1.052	0.7003174	null	probable guanine deaminase	PA1521	282	92.42

1679034	0.759	0.5561199	lig	DNA ligase	PA1529	81	92.22
1679037	1.090	0.909955	null	hypothetical protein	PA1539	183	92.14
1679040	0.709	0.61317927	null	conserved hypothetical protein	PA1536	1	92.7
1679043	0.476	0.17138371	null	probable drug efflux transporter	PA1541	70	97.48
1679045	3.018	3.0566666	null	conserved hypothetical protein	PA1540	60	92
1679047	1.257	1.0349463	null	hypothetical protein	PA1545	125	95.15
1679048	1.009	2.3068182	null	hypothetical protein	PA1542	255	92.19
1679051	2.435	0.52436817	null	probable short-chain dehydrogenase	PA1537	398	93.03
1679054	0.688	0.45655838	null	conserved hypothetical protein	PA1548	81	95.39
1679057	5.377	0.85990894	null	hypothetical protein	PA1412	482	93.47
1679060	0.963	0.7270751	apt	adenine phosphoribosyltransferase	PA1543	308	93.08
1679063	1.124	0.9671693	null	hypothetical protein	PA1550	8	92.41
1679066	1.313	1.1643736	hemN	oxygen-independent coproporphyrinogen III	PA1546	1	93.06
1679069	0.672	0.47523594	null	hypothetical protein	PA1547	636	97.89
1679072	1.345	1.1640428	null	probable ferredoxin	PA1551	3	92.1
1679075	0.968	0.6661207	null	probable cation-transporting P-type ATPase	PA1549	157	96.66
1679078	1.392	1.4993914	null	probable cytochrome c	PA1555	27	92.03
1679081	1.006	0.886804	anr	transcriptional regulator Anr	PA1544	120	92.62
1679084	1.355	1.1010817	null	probable cytochrome c	PA1552	299	93.35
1679087	1.099	0.4898352	null	hypothetical protein	PA1559	297	96.85
1679092	1.309	1.1413758	null	probable cytochrome c oxidase subunit	PA1556	410	94.5
1679094	0.797	0.004524159	null	hypothetical protein	PA1560	183	92.02
1679096	1.065	0.5803031	null	conserved hypothetical protein	PA1563	31	93.33
1679099	0.914	0.6430519	null	conserved hypothetical protein	PA1564	37	92.08
1679102	0.739	1.4699074	null	probable flavin-containing monooxygenase	PA1538	21	92.81
1679105	1.323	1.1528547	aer	aerotaxis receptor Aer	PA1561	1	92.95
1679108	0.681	0.56504506	recR	recombination protein RecR	PA1534	375	93.96
1679111	1.142	0.8243607	null	hypothetical protein	PA1558	131	96.93
1679114	1.418	1.2346741	acnA	aconitate hydratase 1	PA1562	7	92.19
1679117	1.433	1.0333245	null	hypothetical protein	PA1571	133	95.19
1679119	0.842	0.62603647	null	conserved hypothetical protein	PA1572	153	92.68
1679124	0.911	0.7978738	null	conserved hypothetical protein	PA1566	892	94
1679125	0.909	-3.7244105	null	hypothetical protein	PA1575	1	93.38
1679128	1.009	1.0054286	null	conserved hypothetical protein	PA1568	181	92.83
1679131	1.009	0.64828503	null	probable oxidoreductase	PA1565	738	92.5
1679134	1.114	0.7875792	null	conserved hypothetical protein	PA1574	197	95.14
1679136	1.406	1.0114024	null	conserved hypothetical protein	PA1567	221	95.32
1679139	0.561	0.3078079	null	probable 3-hydroxyisobutyrate dehydrogenase	PA1576	50	92.55
1679142	1.231	1.0025688	null	probable cytochrome c oxidase subunit	PA1553	445	92.36
1679145	1.280	0.68422097	null	hypothetical protein	PA1577	4	92.46
1679148	1.140	0.9808752	null	hypothetical protein	PA1579	2	93.61
1679151	1.111	1.6034092	null	hypothetical protein	PA1578	125	97.58
1679154	0.818	0.7234758	sdhC	succinate dehydrogenase (C subunit)	PA1581	168	95.78
1679157	1.264	1.1010817	sdhB	succinate dehydrogenase (B subunit)	PA1584	43	92.25
1679160	1.289	1.1310333	gltA	citrate synthase	PA1580	42	93.92
1679163	0.912	0.529697	null	conserved hypothetical protein	PA1573	224	95.68
1679166	1.372	1.1974676	null	probable cytochrome oxidase subunit (cbb3-type)	PA1554	226	92.28
1679169	1.083	0.8624331	sucB	dihydrolipoamide succinyltransferase (E2	PA1586	7	92.95
1679172	0.761	0.7063877	sdhD	succinate dehydrogenase (D subunit)	PA1582	107	93
1679175	1.124	0.93310386	null	probable cytochrome oxidase subunit (cbb3-type)	PA1557	374	92.3
1679178	1.206	0.91012746	lpdG	lipoamide dehydrogenase-glc	PA1587	260	93.44
1679181	1.101	1.1143777	sucC	succinyl-CoA synthetase beta chain	PA1588	162	92.68
1679184	1.039	0.29446214	null	hypothetical protein	PA1591	8	92.75
1679187	0.943	0.91446763	sdhA	succinate dehydrogenase (A subunit)	PA1583	105	93.42
1679190	1.243	0.81869173	null	hypothetical protein	PA1594	220	92.48
1679193	0.983	0.7197006	braB	branched chain amino acid transporter	PA1590	97	96.57
1679197	1.237	1.2182069	null	hypothetical protein	PA1592	162	97.96
1679198	1.726	-0.3583913	null	probable transcriptional regulator	PA1570	90	95.95
1679201	1.244	1.2278755	sucD	succinyl-CoA synthetase alpha chain	PA1589	192	94.41
1679204	0.947	0.67263925	null	conserved hypothetical protein	PA1598	13	95.13
1679207	1.389	1.0520418	null	hypothetical protein	PA1593	28	97.12
1679210	1.017	0.9823038	sucA	2-oxoglutarate dehydrogenase (E1 subunit)	PA1585	18	92.62
1679213	0.871	0.82546747	null	probable transcriptional regulator	PA1599	98	95.72
1679216	0.939	0.7682152	htpG	heat shock protein HtpG	PA1596	200	92.47
1679219	1.150	0.76102656	null	probable transcriptional regulator	PA1603	1	94.22
1679221	1.083	0.78733623	null	hypothetical protein	PA1595	37	92.47
1679224	1.009	-0.08619046	null	hypothetical protein	PA1606	99	92.52
1679227	0.968	0.44689518	null	conserved hypothetical protein	PA1607	1	93.53
1679230	0.821	0.52861583	null	hypothetical protein	PA1605	239	93.14
1679233	1.009	1.3284849	null	hypothetical protein	PA1604	504	92.07
1679236	0.950	0.85756385	fabA	beta-hydroxydecanoyl-ACP dehydrase	PA1610	163	92.25
1679239	1.402	1.2844956	null	probable chemotaxis transducer	PA1608	18	97.24
1679242	1.113	1.0374126	fabB	beta-ketoacyl-ACP synthase I	PA1609	1	92.48
1679245	1.015	0.30052692	null	hypothetical protein	PA1597	5	92.34
1679248	1.048	0.78956795	null	probable lipase	PA1615	193	92.68
1679251	0.999	0.6484252	null	hypothetical protein	PA1612	182	94.98
1679254	0.900	0.6298386	null	conserved hypothetical protein	PA1616	328	96.64
1679257	0.904	0.8284277	null	probable sensor/response regulator hybrid	PA1611	70	92.34
1679260	1.026	0.7110327	null	conserved hypothetical protein	PA1618	207	93.15
1679263	1.027	0.8379601	gpsA	glycerol-3-phosphate dehydrogenase,	PA1614	141	93.36
1679266	1.424	0.7712842	null	probable hydrolase	PA1621	119	93.76
1679269	0.915	1.406111	null	probable major facilitator superfamily (MFS)	PA1569	143	92.84
1679272	0.485	0.18657649	null	probable hydrolase	PA1622	183	97.87
1679276	1.057	0.8675114	null	hypothetical protein	PA1620	148	93.73
1679278	0.993	0.7957412	null	conserved hypothetical protein	PA1623	68	94.35
1679281	0.825	0.6107564	null	probable transcriptional regulator	PA1619	49	93.82
1679284	1.027	0.6926495	null	probable aldehyde dehydrogenase	PA1601	214	92.73
1679287	0.354	0.13004097	null	hypothetical protein	PA1624	105	93.71
1679290	0.585	0.4853409	null	probable AMP-binding enzyme	PA1617	47	93.31
1679293	1.103	1.1827836	null	hypothetical protein	PA1613	1	97.03
1679296	2.175	1.6082354	null	probable oxidoreductase	PA1602	400	94.15
1679299	1.133	1.0616554	null	probable transcriptional regulator	PA1627	258	97.98
1679302	1.009	1.0972806	null	probable acyl-CoA dehydrogenase	PA1631	59	93.25
1679305	1.319	0.95010406	null	probable major facilitator superfamily (MFS)	PA1626	26	96.08
1679308	1.528	0.8762971	null	probable enoyl-CoA hydratase/isomerase	PA1629	21	92.43
1679311	1.088	0.61043537	null	probable transcriptional regulator	PA1630	54	92.33
1679314	1.038	0.89607096	null	hypothetical protein	PA1639	1	94.41
1679317	1.009	-0.569394	kdpC	potassium-transporting ATPase, C chain	PA1635	441	96.98
1679320	1.070	0.7729123	null	conserved hypothetical protein	PA1638	250	97.02
1679323	0.593	-0.96237355	null	probable decarboxylase	PA1417	353	93.59
1679326	0.811	0.3112997	kdpE	two-component response regulator KdpE	PA1637	42	92.54
1679329	1.047	0.658214	null	conserved hypothetical protein	PA1640	80	92.87
1679332	0.855	0.64895034	null	conserved hypothetical protein	PA1625	188	95
1679337	1.009	0.38298613	null	hypothetical protein	PA1641	209	93.31
1679338	1.115	0.7974618	null	hypothetical protein	PA1645	1	94.04
1679341	1.387	0.9546712	null	conserved hypothetical protein	PA1643	69	93.01
1679344	1.236	0.87718564	selD	selenophosphate synthetase	PA1642	42	92.78
1679347	0.974	4.8105006	null	probable 3-hydroxyacyl-CoA dehydrogenase	PA1628	2	92.18
1679350	1.599	0.27548355	null	probable short-chain dehydrogenase	PA1649	447	94.93

1679353	1.079	1.0485661	null	probable transporter	PA1650	1	94.04
1679356	1.082	-0.75741756	null	probable oxidoreductase	PA1648	313	95.5
1679359	2.004	0.91138744	kdpA	potassium-transporting ATPase, A chain	PA1633	60	92.69
1679362	1.222	0.9386883	null	hypothetical protein	PA1652	287	93.62
1679365	1.176	0.843753	null	probable transcriptional regulator	PA1653	109	96.47
1679368	1.205	0.92465436	null	probable transporter	PA1651	1	95.82
1679371	0.725	0.62152207	null	hypothetical protein	PA1656	222	93.13
1679374	1.202	0.9881803	null	probable glutathione S-transferase	PA1655	85	93.95
1679377	0.947	0.7037886	null	conserved hypothetical protein	PA1644	25	96.85
1679380	0.439	0.32961497	null	conserved hypothetical protein	PA1657	13	92.11
1679383	0.502	0.36408523	null	hypothetical protein	PA1659	255	93.73
1679386	0.906	0.6200925	null	probable sulfate transporter	PA1647	1	95.25
1679389	1.052	0.80534136	null	probable aminotransferase	PA1654	241	95.22
1679392	0.086	-0.10756645	null	conserved hypothetical protein	PA1658	1	95.22
1679395	1.013	0.8772337	null	hypothetical protein	PA1664	1	97.27
1679396	1.009	2.5348485	null	probable transcriptional regulator	PA1663	114	93.58
1679399	0.870	0.56425107	null	probable ClpA/B-type protease	PA1662	112	93.37
1679402	1.065	-0.03499999	null	hypothetical protein	PA1668	35	95.94
1679405	0.317	1.5728447	null	hypothetical protein	PA1666	62	93.75
1679408	1.009	-1.3142362	null	hypothetical protein	PA1665	37	92.3
1679411	1.109	0.63843	kdpD	two-component sensor KdpD	PA1636	169	96.27
1679414	1.376	0.9685818	dnaX	DNA polymerase subunits gamma and tau	PA1532	16	92.31
1679417	0.628	0.43726245	null	hypothetical protein	PA1660	1	95.1
1679420	1.227	-1.7660475	stp1	serine/threonine phosphoprotein phosphatase	PA1670	47	97.36
1679423	0.994	0.64330876	null	hypothetical protein	PA1672	3	92.59
1679426	0.594	0.22625001	null	hypothetical protein	PA1661	162	95.53
1679429	0.661	-5.5170307	null	hypothetical protein	PA1667	65	92.17
1679432	0.753	0.6775674	null	hypothetical protein	PA1676	212	94.74
1679433	1.033	0.9627226	null	hypothetical protein	PA1673	134	92.74
1679436	1.046	1.0338953	null	conserved hypothetical protein	PA1677	278	94.27
1679439	0.366	4.140918	null	conserved hypothetical protein	PA1675	220	94.57
1679443	0.747	0.5624178	folE2	GTP cyclohydrolase I precursor	PA1674	3	92.14
1679445	1.158	0.85868347	null	hypothetical protein	PA1679	144	97.83
1679448	0.937	0.59688413	null	hypothetical protein	PA1669	28	92.78
1679451	0.923	-0.077223495	stk1	serine-threonine kinase Stk1	PA1671	513	92.13
1679454	0.704	0.31350678	null	probable sugar aldolase	PA1683	200	95.88
1679457	1.139	1.2979523	alkA	DNA-3-methyladenine glycosidase II	PA1686	10	93.82
1679460	0.897	0.7133106	null	hypothetical protein	PA1680	55	97.2
1679463	0.734	0.31038618	masA	enolase-phosphatase E-1	PA1685	41	92.07
1679466	1.138	0.9470722	null	probable MFS metabolite transporter	PA1682	287	96.27
1679469	1.009	1.1608334	null	probable chemotaxis transducer	PA1646	367	94.22
1679472	1.371	-0.22107434	speE	spermidine synthase	PA1687	38	93.73
1679475	0.911	0.7153486	null	probable oxidase	PA1684	204	94.42
1679478	1.182	0.8518101	null	probable translocation protein in type III	PA1692	6	93.54
1679481	1.082	1.1145239	pscU	translocation protein in type III secretion	PA1690	54	92.42
1679484	1.426	1.58042	null	hypothetical protein	PA1688	75	97.94
1679487	1.198	0.86724836	pscR	translocation protein in type III secretion	PA1693	171	93.88
1679490	0.218	0.07339081	pscO	translocation protein in type III secretion	PA1696	410	93.1
1679491	1.266	0.89055884	pscQ	translocation protein in type III secretion	PA1694	132	96.42
1679494	1.073	0.63622683	null	conserved hypothetical protein	PA1689	335	92.19
1679497	1.000	0.72222227	aroC	chorismate synthase	PA1681	1	93.62
1679500	0.602	0.30581245	null	conserved hypothetical protein in type III	PA1700	68	95.52
1679502	1.093	1.0308032	null	ATP synthase in type III secretion system	PA1697	8	93.63
1679505	0.111	-0.48022085	null	conserved hypothetical protein in type III	PA1701	306	92.82
1679507	0.308	0.001039328	null	conserved hypothetical protein in type III	PA1702	218	96.93
1679509	0.416	10.783365	popN	Type III secretion outer membrane protein PopN	PA1698	63	97.91
1679512	0.426	0.04608631	null	conserved hypothetical protein in type III	PA1699	4	92.13
1679515	0.310	-0.8426407	pcrG	regulator in type III secretion	PA1705	1	92.6
1679516	0.266	-0.07401938	pscP	translocation protein in type III secretion	PA1695	335	95.08
1679519	0.779	0.4954752	pcrR	transcriptional regulator protein PcrR	PA1704	72	97.55
1679521	0.245	-0.07944648	exsC	ExsC, exoenzyme S synthesis protein C	PA1710	10	92.56
1679524	0.370	0.21305175	pcrH	regulatory protein PcrH	PA1707	70	95.4
1679527	0.260	0.21714191	exsE	ExsE	PA1711	23	97.89
1679529	0.499	0.35257635	pcrV	type III secretion protein PcrV	PA1706	11	92.47
1679532	0.410	0.30513468	popB	translocator protein PopB	PA1708	1	92.55
1679535	0.190	0.15435147	exsB	exoenzyme S synthesis protein B	PA1712	135	92.11
1679538	1.009	1.2202238	pscT	translocation protein in type III secretion	PA1691	250	95.35
1679541	0.316	0.23478267	exsA	transcriptional regulator ExsA	PA1713	572	93.95
1679544	0.326	0.22142279	pscE	type III export protein PscE	PA1718	148	96.02
1679545	0.042	0.016030086	pscB	type III export apparatus protein	PA1715	37	94.14
1679547	0.290	0.2453607	exsD	ExsD	PA1714	81	95.59
1679550	0.905	0.64742815	null	probable cytochrome c	PA1600	342	92.3
1679554	0.980	0.7483549	pscG	type III export protein PscG	PA1720	178	97.86
1679556	0.247	0.1215026	pscI	type III export protein PscI	PA1722	135	94.04
1679559	0.278	0.17111099	pscH	type III export protein PscH	PA1721	61	96.24
1679562	0.663	0.5172643	pscJ	type III export protein PscJ	PA1723	44	93.58
1679566	0.088	8.406623	pscF	type III export protein PscF	PA1719	98	95.93
1679568	0.233	-0.4572204	pscL	type III export protein PscL	PA1725	220	92.39
1679571	0.988	2.034474	null	hypothetical protein	PA1728	82	92.28
1679574	0.229	0.16224073	popD	Translocator outer membrane protein PopD	PA1709	276	92.44
1679577	1.727	1.0939152	null	probable DNA methylase	PA1678	67	94.96
1679580	1.116	0.8375767	null	conserved hypothetical protein	PA1730	181	96.11
1679583	1.665	0.9343523	null	conserved hypothetical protein	PA1731	302	93.86
1679586	0.895	0.7716288	null	conserved hypothetical protein	PA1729	443	94.69
1679589	0.175	-0.98383486	pscC	Type III secretion outer membrane protein PscC	PA1716	66	95.52
1679592	1.108	1.0873287	null	hypothetical protein	PA1734	20	97.34
1679595	0.339	0.039860666	pscD	type III export protein PscD	PA1717	612	94.44
1679598	1.009	1.1489724	null	hypothetical protein	PA1735	68	92.74
1679601	1.047	-0.35999998	null	conserved hypothetical protein	PA1732	7	92.01
1679604	1.009	2.4977777	null	probable transcriptional regulator	PA1738	239	95.35
1679607	1.031	1.0563242	null	hypothetical protein	PA1740	10	92.23
1679610	0.870	0.71991384	pscK	type III export protein PscK	PA1724	1	95.23
1679613	0.849	0.5950035	null	probable oxidoreductase	PA1739	324	94.16
1679616	1.016	0.8523704	null	hypothetical protein	PA1741	121	93.4
1679619	1.290	0.9723398	null	probable amidotransferase	PA1742	58	92.66
1679622	170.205	3.7018745	null	hypothetical protein	PA1743	36	92.4
1679623	1.009	1.7630107	kdpB	potassium-transporting ATPase, B chain	PA1634	27	92.4
1679626	0.839	1.2869937	null	probable acyl-CoA thiolase	PA1736	218	95.96
1679629	1.074	1.0086396	null	hypothetical protein	PA1747	5	93.01
1679631	0.948	0.45149904	bglX	periplasmic beta-glucosidase	PA1726	3	93.29
1679634	1.105	-0.08325791	null	hypothetical protein	PA1745	10	93.9
1679637	0.995	0.7874007	null	probable enoyl-CoA hydratase/isomerase	PA1748	37	92.88
1679640	1.170	0.9987742	null	hypothetical protein	PA1746	200	92.58
1679643	3.611	2.644508	null	hypothetical protein	PA1744	41	93.9
1679645	0.728	0.50084376	null	hypothetical protein	PA1751	25	92.36
1679648	0.699	0.43206128	null	hypothetical protein	PA1752	238	95.44
1679651	1.055	0.82297504	cysB	transcriptional regulator CysB	PA1754	20	92.05
1679654	1.009	6.5220957	null	hypothetical protein	PA1755	115	96.46
1679657	1.288	0.8601781	thrH	homoserine kinase	PA1757	21	93.46

1679660	0.967	0.7805072	null	phospho-2-dehydro-3-deoxyheptonate aldolase	PA1750	126	94.24
1679663	0.767	0.55960435	pcrD	type III secretory apparatus protein PcrD	PA1703	149	92.66
1679666	0.960	0.83781064	null	conserved hypothetical protein	PA1753	21	92.42
1679669	0.696	0.609089	null	hypothetical protein	PA1749	196	92.49
1679672	1.317	1.1386551	null	hypothetical protein	PA1761	15	92.99
1679675	1.555	0.38155368	pabB	para-aminobenzoate synthase component I	PA1758	36	92.69
1679678	4.703	1.441843	null	conserved hypothetical protein	PA1727	30	92.88
1679681	1.009	2.4459524	null	hypothetical protein	PA1763	154	93.6
1679684	1.049	0.84962064	null	hypothetical protein	PA1766	17	92.59
1679687	0.889	-0.23667093	null	hypothetical protein	PA1762	26	92.08
1679690	0.973	0.8069557	null	probable transcriptional regulator	PA1759	1	94.09
1679693	1.009	3.1180851	null	probable 3-hydroxyacyl-CoA dehydrogenase	PA1737	41	95.33
1679696	0.422	-3.6898391	cysH	3'-phosphoadenosine-5'-phosphosulfate reductase	PA1756	44	97.22
1679699	1.114	0.931502	null	conserved hypothetical protein	PA1769	11	92.45
1679702	0.951	0.8209863	ppsA	phosphoenolpyruvate synthase	PA1770	59	94.07
1679705	1.009	1.9577011	null	hypothetical protein	PA1764	180	97.13
1679708	2.350	0.9482342	null	probable methyltransferase	PA1772	37	92.36
1679711	0.637	0.35866332	estX	EstX	PA1771	44	92.35
1679714	1.189	0.50733906	null	hypothetical protein	PA1768	44	94.69
1679717	0.997	0.6790986	cmaX	CmaX protein	PA1773	67	95.5
1679720	1.294	0.93541384	null	probable transcriptional regulator	PA1760	2	96.98
1679723	1.166	1.279835	oprF	Major porin and structural outer membrane porin	PA1777	11	92.77
1679726	0.768	0.5559849	sigX	ECF sigma factor SigX	PA1776	1	92.23
1679729	0.374	0.95796573	cobA	uroporphyrin-III C-methyltransferase	PA1778	101	92.46
1679732	0.913	0.7528538	cfrX	CfrX protein	PA1774	1	93.73
1679735	1.033	0.2012963	nirD	assimilatory nitrite reductase small subunit	PA1780	160	93.02
1679738	1.279	1.1599907	null	hypothetical protein	PA1765	1	94
1679741	0.724	0.75868404	null	hypothetical protein	PA1784	6	92.19
1679744	0.981	0.80654544	null	hypothetical protein	PA1767	154	93.99
1679747	1.375	1.1989301	null	hypothetical protein	PA1789	13	92.19
1679750	1.009	1.7686071	null	conserved hypothetical protein	PA1785	13	93.36
1679753	1.018	0.7368697	null	conserved hypothetical protein	PA1792	60	96.5
1679756	1.204	0.8728651	null	hypothetical protein	PA1790	14	92.78
1679759	1.139	0.84402734	null	assimilatory nitrate reductase	PA1779	1	94.94
1679762	1.009	3.3104336	nirB	assimilatory nitrite reductase large subunit	PA1781	389	96.92
1679765	1.210	1.047495	ppiB	peptidyl-prolyl cis-trans isomerase B	PA1793	159	95.06
1679768	0.168	0.15269141	null	hypothetical protein	PA1791	98	92.3
1679771	0.969	0.51046187	null	hypothetical protein	PA1788	24	92.29
1679774	1.171	1.1186283	acnB	aconitate hydratase 2	PA1787	1	92.01
1679777	0.922	0.6724504	folD	5,10-methylene-tetrahydrofolate dehydrogenase /	PA1796	34	96.34
1679780	1.009	0.24000001	null	probable serine/threonine-protein kinase	PA1782	316	95.81
1679783	0.939	0.44415265	null	probable two-component sensor	PA1798	254	92.53
1679786	0.887	0.86571705	clpP	ATP-dependent Clp protease proteolytic subunit	PA1801	246	93.36
1679789	0.767	0.53900045	cmpX	conserved cytoplasmic membrane protein, CmpX	PA1775	12	92.31
1679792	1.016	5.101262	null	probable two-component response regulator	PA1799	78	93.5
1679795	1.009	1.188965	nasA	nitrate transporter	PA1783	119	95.26
1679798	0.873	0.6181976	glnS	glutaminyl-tRNA synthetase	PA1794	212	93.23
1679801	0.896	0.85373	clpX	ATP-dependent Clp protease ATP-binding subunit	PA1802	124	92.53
1679804	1.263	1.0437535	cysS	cysteinyl-tRNA synthetase	PA1795	86	94.44
1679807	0.822	0.8014797	hupB	DNA-binding protein HU	PA1804	71	93.46
1679809	1.009	2.2553334	null	conserved hypothetical protein	PA1786	35	95.93
1679812	1.013	0.81599665	lon	Lon protease	PA1803	44	93.84
1679815	0.716	0.09655068	null	conserved hypothetical protein	PA1733	368	92.63
1679818	0.857	0.7361971	mltD	membrane-bound lytic murein transglycosylase D	PA1812	42	97.94
1679821	1.093	0.8159269	null	probable permease of ABC transporter	PA1808	26	96.85
1679824	0.899	1.8456867	null	hypothetical protein	PA1797	108	92.53
1679827	0.466	-0.1949254	null	hypothetical protein	PA1817	3	92.66
1679830	0.962	0.77708256	null	hypothetical protein	PA1814	125	92.08
1679833	1.311	1.0116447	fabI	NADH-dependent enoyl-ACP reductase	PA1806	25	95.08
1679836	1.305	0.4150638	null	probable ATP-binding component of ABC	PA1807	185	97.36
1679839	0.986	0.8227953	dnaQ	DNA polymerase III, epsilon chain	PA1816	97	94.53
1679842	0.965	0.8021819	ppiD	peptidyl-prolyl cis-trans isomerase D	PA1805	78	95.53
1679845	0.616	0.4508726	null	conserved hypothetical protein	PA1823	310	94.65
1679848	0.969	0.73825324	null	probable permease of ABC transporter	PA1809	317	94.5
1679851	0.819	0.5773095	null	probable binding protein component of ABC	PA1810	61	93.24
1679854	0.803	3.0025651	null	conserved hypothetical protein	PA1824	2	92.05
1679857	1.046	0.91124547	tig	trigger factor	PA1800	56	92.41
1679860	1.145	0.82095164	null	hypothetical protein	PA1825	1	92.76
1679863	0.989	0.70509654	null	probable enoyl-CoA hydratase/isomerase	PA1821	11	92.86
1679866	1.455	1.2764566	null	hypothetical protein	PA1830	60	92.5
1679869	0.699	0.1698752	null	probable transcriptional regulator	PA1826	136	95.96
1679872	0.782	0.6321012	fimL	hypothetical protein	PA1822	1	93.21
1679875	1.253	0.9053518	null	probable short-chain dehydrogenase	PA1828	98	94.42
1679878	1.352	1.1044079	null	hypothetical protein	PA1831	1	92.55
1679881	1.146	0.6983673	null	probable short-chain dehydrogenase	PA1827	332	94.27
1679884	0.624	0.41749993	null	hypothetical protein	PA1834	41	96.69
1679887	1.002	0.62089574	null	probable hydroxyacylglutathione hydrolase	PA1813	127	93.68
1679890	0.787	0.68457955	null	probable amino acid permease	PA1819	56	92.37
1679893	1.315	0.3395098	null	probable protease	PA1832	8	93.09
1679896	1.019	0.8078675	null	probable oxidoreductase	PA1833	1	94.31
1679899	1.803	1.0008676	null	hypothetical protein	PA1835	369	92.34
1679902	0.751	1.0406833	null	hypothetical protein	PA1840	23	93.36
1679904	1.009	0.7106188	null	probable transcriptional regulator	PA1836	80	92.2
1679907	0.916	0.75667304	rnhA	ribonuclease H	PA1815	171	93.28
1679910	0.840	0.54161423	null	hypothetical protein	PA1842	314	92.71
1679913	0.956	0.6360695	null	hypothetical protein	PA1837	218	92.52
1679916	0.731	0.6019999	cysI	sulfite reductase	PA1838	118	92.18
1679919	1.264	1.1006584	null	conserved hypothetical protein	PA1847	2	92.83
1679922	1.193	0.98074263	null	hypothetical protein	PA1829	53	94.33
1679925	0.900	0.71310246	null	hypothetical protein	PA1841	378	92.71
1679928	1.143	0.82744443	null	hypothetical protein	PA1845	54	92.72
1679931	1.448	1.0604513	null	conserved hypothetical protein	PA1849	59	94.66
1679932	0.892	0.69962984	null	hypothetical protein	PA1844	402	92.83
1679935	1.190	0.87364495	null	hypothetical protein	PA1852	46	94.96
1679938	1.155	1.1287916	null	hypothetical protein	PA1855	1	96.5
1679940	1.281	1.3071808	null	conserved hypothetical protein	PA1854	478	93.22
1679943	1.059	0.8682173	null	probable transcriptional regulator	PA1850	7	92.32
1679946	0.926	0.7588655	null	probable major facilitator superfamily (MFS)	PA1848	37	94.64
1679949	1.661	1.4888351	str	streptomycin 3''-phosphotransferase	PA1858	139	92.35
1679952	0.968	0.8327778	null	conserved hypothetical protein	PA1857	4	95.46
1679955	1.603	1.0084518	null	hypothetical protein	PA1860	313	92.14
1679958	0.941	0.6980939	modB	molybdenum transport protein ModB	PA1862	1	94.76
1679961	1.259	1.9530084	metH	methionine synthase	PA1843	32	93.76
1679964	1.164	0.869841	null	probable cytochrome oxidase subunit	PA1856	419	93.39
1679967	1.012	0.6516155	cti	cis/trans isomerase	PA1846	77	92.02
1679970	1.009	1.7950588	null	probable transcriptional regulator	PA1859	765	92.61
1679973	1.007	2.520702	modA	molybdate-binding periplasmic protein precursor	PA1863	117	93.04
1679976	1.147	0.08212249	modC	molybdenum transport protein ModC	PA1861	912	94.23
1679979	1.000	-0.5340909	null	probable transcriptional regulator	PA1864	450	92.69

1679982	0.213	0.15994743	null	probable acyl carrier protein	PA1869	92	92.37
1679985	2.375	1.2727058	nhaB	sodium/proton antiporter NhaB	PA1820	1	92.65
1679988	1.009	1.3328947	null	hypothetical protein	PA1870	204	92
1679991	0.319	-0.51326615	null	hypothetical protein	PA1872	49	93.86
1679994	1.008	0.71530044	null	hypothetical protein	PA1867	1	97.7
1679995	1.408	0.77912664	null	probable transcriptional regulator	PA1853	513	92.09
1679998	1.009	1.1919048	lasA	LasA protease precursor	PA1871	118	92.98
1680001	1.309	0.9232895	null	hypothetical protein	PA1865	2	92.94
1680004	1.517	1.053932	null	hypothetical protein	PA1873	305	92.82
1680007	0.946	0.7734698	null	hypothetical protein	PA1866	125	97
1680010	2.383	0.8625542	null	hypothetical protein	PA1874	87	92.39
1680013	0.931	0.6680075	null	hypothetical protein	PA1879	236	95.2
1680016	4.170	0.9263493	null	probable outer membrane protein precursor	PA1875	157	96.04
1680019	1.385	0.9687476	null	hypothetical protein	PA1878	408	95.96
1680022	1.423	0.61036813	null	probable oxidoreductase	PA1881	1	93.3
1680025	1.557	2.7500134	null	probable NADH-ubiquinone/plastoquinone	PA1883	162	93.44
1680028	1.009	0.28749996	xqhA	secretion protein XqhA	PA1868	163	92.07
1680031	0.750	0.5701133	null	probable transporter	PA1882	21	93.67
1680034	1.158	0.81418216	null	hypothetical protein	PA1887	77	96.41
1680037	1.096	0.79468536	polB	DNA polymerase II	PA1886	2	93.02
1680040	1.923	1.1247603	null	probable glutathione S-transferase	PA1890	117	97
1680043	1.009	6.0250573	null	hypothetical protein	PA1888	90	92.53
1680046	0.887	-2.6401205	null	hypothetical protein	PA1889	79	93.52
1680049	1.009	0.8312715	null	probable transcriptional regulator	PA1884	1	93.52
1680052	1.062	0.8012843	null	conserved hypothetical protein	PA1885	211	95.55
1680055	2.134	1.4448475	null	hypothetical protein	PA1891	303	94.1
1680057	1.009	-0.44214287	null	hypothetical protein	PA1892	50	92.17
1680060	1.031	-1.6539682	null	hypothetical protein	PA1894	227	92.84
1680063	0.651	0.9725677	null	probable secretion protein	PA1877	169	93.57
1680066	0.792	9.637179	phzA2	probable phenazine biosynthesis protein	PA1899	236	92.04
1680068	1.143	0.6056211	null	hypothetical protein	PA1895	48	94.59
1680071	1.531	1.1893531	qscR	quorum-sensing control repressor	PA1898	220	93.28
1680074	1.009	1.4879366	null	hypothetical protein	PA1896	65	92.39
1680077	1.273	0.89385635	null	hypothetical protein	PA1897	88	92.15
1680080	1.009	1.6026075	phzB2	probable phenazine biosynthesis protein	PA1900	35	93.4
1680084	1.009	0.6657249	phzF2	probable phenazine biosynthesis protein	PA1904	247	94.38
1680086	1.388	1.117427	phzD2	phenazine biosynthesis protein PhzD	PA1902	456	93.73
1680091	1.009	-0.6759259	phzG2	probable pyridoxamine 5'-phosphate oxidase	PA1905	598	94.29
1680092	0.942	0.76199746	null	probable Orn/Arg/Lys decarboxylase	PA1818	354	93.56
1680095	1.309	0.7570349	null	hypothetical protein	PA1909	56	92.88
1680098	0.932	0.9037446	null	probable major facilitator superfamily (MFS)	PA1908	253	93.31
1680101	1.052	-3.8396823	null	hypothetical protein	PA1893	200	94.47
1680104	1.246	0.99983007	null	hypothetical protein	PA1913	1	93.7
1680107	1.145	0.8030492	null	hypothetical protein	PA1906	108	95.35
1680110	1.009	0.69653845	null	hypothetical protein	PA1907	1132	92.55
1680113	0.850	0.66391927	null	probable sigma-70 factor, ECF subfamily	PA1912	14	94.14
1680116	1.009	0.33726794	null	conserved hypothetical protein	PA1914	97	94.44
1680119	1.261	1.2315382	null	hypothetical protein	PA1915	1	96.55
1680122	1.009	0.8906666	null	hypothetical protein	PA1917	178	92.01
1680125	1.009	0.9107143	null	probable oxidoreductase	PA1880	171	94.38
1680128	8.166	0.9011612	null	hypothetical protein	PA1921	1	93.6
1680131	0.965	2.4664853	phzC2	phenazine biosynthesis protein PhzC	PA1901	196	94.7
1680134	1.635	1.0695071	null	probable tonB-dependent receptor protein	PA1910	322	97.53
1680137	1.009	3.0115077	null	probable radical-activating enzyme	PA1919	479	94.77
1680140	1.467	0.83291304	null	hypothetical protein	PA1839	141	95.74
1680143	1.009	4.922222	null	conserved hypothetical protein	PA1920	45	92.56
1680146	1.009	0.4665385	null	hypothetical protein	PA1925	101	92.92
1680148	0.644	0.32454342	null	hypothetical protein	PA1918	408	96.71
1680151	0.930	0.6410564	null	hypothetical protein	PA1929	138	95.88
1680153	0.965	0.47529107	null	hypothetical protein	PA1923	56	96.44
1680158	1.074	1.2537879	phzE2	phenazine biosynthesis protein PhzE	PA1903	809	94.68
1680159	1.009	-0.042532466	rimJ	ribosomal protein alanine acetyltransferase	PA1928	254	96.02
1680162	0.517	0.69811743	null	probable chemotaxis transducer	PA1930	86	92.15
1680165	1.052	1.400876	null	probable ferredoxin	PA1931	430	93.73
1680168	1.290	0.3641841	null	hypothetical protein	PA1936	14	92.22
1680170	1.009	0.66761905	null	conserved hypothetical protein	PA1937	181	93.61
1680172	0.887	-4.838437	null	probable hydroxylase molybdopterin-containing	PA1932	79	93.55
1680175	1.486	1.5835714	null	hypothetical protein	PA1935	89	92.3
1680178	2.025	-2.4667304	null	hypothetical protein	PA1934	102	95.91
1680181	0.690	0.46947122	null	hypothetical protein	PA1939	70	92.22
1680184	9.689	9.620763	null	hypothetical protein	PA1942	101	92.17
1680185	0.816	1.4253633	null	hypothetical protein	PA1940	95	93.32
1680188	0.645	1.109756	null	conserved hypothetical protein	PA1926	427	95.09
1680191	0.908	0.13937353	null	conserved hypothetical protein	PA1938	85	93.14
1680194	0.746	-0.33272046	null	hypothetical protein	PA1851	185	92.92
1680197	3.602	3.17863	null	hypothetical protein	PA1941	150	93.49
1680200	1.050	0.69021535	null	hypothetical protein	PA1943	48	92.26
1680203	1.582	0.95630705	null	hypothetical protein	PA1924	387	94.86
1680206	0.710	0.69670606	rbsB	binding protein component precursor of ABC	PA1946	32	94.72
1680209	0.568	7.4078507	rbsC	membrane protein component of ABC ribose	PA1948	117	92.75
1680212	0.895	0.6225652	rbsR	ribose operon repressor RbsR	PA1949	189	92.35
1680215	0.946	0.6554667	null	hypothetical protein	PA1952	8	94.76
1680218	1.420	1.1047643	null	hypothetical protein	PA1953	260	94.01
1680221	1.009	2.3369963	null	probable hydroxylase large subunit	PA1933	503	96.56
1680224	1.172	0.60287845	null	hypothetical protein	PA1951	1	94.34
1680227	0.736	1.0299283	null	probable transmembrane sensor	PA1911	1	95.3
1680230	1.160	0.75894475	null	hypothetical protein	PA1954	24	96.06
1680233	1.495	1.0555032	null	probable transcriptional regulator	PA1945	62	96.98
1680236	1.469	0.8774997	null	hypothetical protein	PA1944	195	97.77
1680239	1.036	0.6118321	rbsK	ribokinase	PA1950	161	95.75
1680242	1.009	1.2596492	null	hypothetical protein	PA1955	140	94.63
1680245	0.970	1.2899829	null	hypothetical protein	PA1956	124	96.68
1680248	0.811	0.6469849	null	hypothetical protein	PA1963	33	94.25
1680250	13.190	4.0378833	null	probable transporter	PA1958	150	92.39
1680253	2.035	0.3808772	null	hypothetical protein	PA1960	45	92.93
1680256	0.899	0.7005935	bacA	bacitracin resistance protein	PA1959	1	92.7
1680259	1.009	-0.48512825	null	conserved hypothetical protein	PA1962	79	94.64
1680262	1.160	1.2235682	null	hypothetical protein	PA1965	1	93.79
1680264	1.004	0.6860378	null	hypothetical protein	PA1968	3	92.76
1680266	1.132	0.804887	null	hypothetical protein	PA1967	33	93.1
1680269	0.504	-0.85628635	null	hypothetical protein	PA1966	4	92.69
1680273	1.063	0.8121401	null	hypothetical protein	PA1969	209	96.16
1680275	1.664	1.4392198	null	hypothetical protein	PA1970	51	95.11
1680277	1.009	2.0185966	null	probable transcriptional regulator	PA1961	165	94.88
1680280	1.065	0.8185684	braZ	branched chain amino acid transporter BraZ	PA1971	55	95.44
1680283	3.057	0.6049168	null	probable ATP-binding/permease fusion ABC	PA1876	644	96.54
1680286	0.428	-1.047439	null	hypothetical protein	PA1974	253	94.38
1680289	1.133	0.7928051	pqqF	pyrroloquinoline quinone biosynthesis protein F	PA1973	3	97.07
1680292	1.009	4.182231	null	probable TonB-dependent receptor	PA1922	270	97.37

1680295	1.009	2.975	null	hypothetical protein	PA1957	1	93.8
1680298	1.035	0.3845614	null	probable two-component sensor	PA1979	102	94.37
1680300	1.081	0.9145895	null	probable amino acid permease	PA1916	165	92.06
1680303	0.994	0.86124355	null	hypothetical protein	PA1977	1	96.39
1680306	1.154	0.7003668	null	probable transcriptional regulator	PA1978	36	93.9
1680309	1.332	1.0366148	null	probable ATP-binding component of ABC	PA1964	527	93.78
1680312	1.113	0.91036433	pqqA	pyrroloquinoline quinone biosynthesis protein A	PA1985	23	92.6
1680313	0.957	0.7954801	null	conserved hypothetical protein	PA1972	1	95.79
1680316	0.387	1.9186814	null	probable two-component response regulator	PA1980	213	92.87
1680319	0.854	0.52191013	null	hypothetical protein	PA1975	103	92.39
1680322	0.739	0.40735298	pqqC	pyrroloquinoline quinone biosynthesis protein C	PA1987	66	92.21
1680325	1.231	0.98433006	pqqD	pyrroloquinoline quinone biosynthesis protein D	PA1988	64	95.03
1680327	1.009	1.5165714	exaA	quinoprotein alcohol dehydrogenase	PA1982	189	92.97
1680330	1.009	1.3797319	pqqB	pyrroloquinoline quinone biosynthesis protein B	PA1986	867	94.28
1680333	1.009	1.6580349	null	probable two-component sensor	PA1976	81	94.81
1680336	0.422	0.028351653	null	probable iron-containing alcohol dehydrogenase	PA1991	63	95.68
1680339	0.669	4.801795	pqqE	pyrroloquinoline quinone biosynthesis protein E	PA1989	54	93.58
1680342	1.252	1.0673015	null	probable aldehyde dehydrogenase	PA1984	24	93.4
1680345	1.167	1.9982141	ppiC1	peptidyl-prolyl cis-trans isomerase C1	PA1996	155	93.74
1680347	1.061	-6.646434	exaB	cytochrome c550	PA1983	116	96.56
1680350	1.006	0.8280522	null	hypothetical protein	PA1995	218	97.7
1680352	1.027	0.7470213	null	hypothetical protein	PA1994	162	94.64
1680355	0.527	0.22792432	null	probable CoA transferase, subunit A	PA1999	132	96.6
1680358	1.316	0.9474762	null	probable major facilitator superfamily (MFS)	PA1993	751	97.45
1680361	1.499	1.1095688	null	probable transcriptional regulator	PA1998	241	93.4
1680364	1.009	1.1823392	null	hypothetical protein	PA1981	589	93.9
1680367	1.009	1.5938132	bdhA	3-hydroxybutyrate dehydrogenase	PA2003	278	96.94
1680370	0.635	-3.435561	null	probable AMP-binding enzyme	PA1997	40	96.09
1680375	0.749	0.31571364	atoB	acetyl-CoA acetyltransferase	PA2001	1082	94.39
1680376	0.492	-0.3311802	null	probable CoA transferase, subunit B	PA2000	34	92.28
1680379	9.688	-0.19849458	maiA	maleylacetoacetate isomerase	PA2007	335	95.04
1680382	1.198	0.8353141	null	conserved hypothetical protein	PA2004	476	92.38
1680385	0.885	0.48522034	null	probable transcriptional regulator	PA2005	177	97.9
1680388	1.215	0.6797306	null	conserved hypothetical protein	PA2002	1	92.79
1680391	1.133	1.083925	gnyL	3-hydroxy-gamma-carboxygeranoyl-CoA lyase, GnyL	PA2011	41	94.9
1680394	1.283	0.071123004	gnyH	gamma-carboxygeranoyl-CoA hydratase, GnyH	PA2013	98	93.42
1680397	0.822	2.3879166	null	probable two-component sensor	PA1992	1083	92.45
1680400	1.623	1.3261621	hmgA	homogentisate 1,2-dioxygenase	PA2009	1	92.24
1680403	0.608	0.5566191	gnyR	Regulatory gene of gnyRDBHAL cluster, GnyR	PA2016	318	92.24
1680406	0.157	-0.07275465	gnyD	Citronelloyl-CoA dehydrogenase, GnyD	PA2015	98	92.55
1680409	1.237	0.8770319	null	hypothetical protein	PA2017	253	92.72
1680412	1.497	0.9808529	gnyA	alpha subunit of geranoyl-CoA carboxylase, GnyA	PA2012	591	92.43
1680415	1.084	0.8460909	null	probable transcriptional regulator	PA2020	118	94.8
1680418	1.009	2.2745752	null	hypothetical protein	PA2021	14	92.51
1680420	0.862	-1.3261904	null	probable peptidase	PA1990	1154	96.94
1680423	2.734	-0.4524828	null	Resistance-Nodulation-Cell Division (RND)	PA2019	960	93.06
1680426	0.622	0.45326108	gnyB	beta subunit of geranoyl-CoA carboxylase, GnyB	PA2014	1	92.81
1680429	0.996	1.0875152	null	probable nucleotide sugar dehydrogenase	PA2022	1	92.83
1680432	1.098	1.0414683	galU	UTP--glucose-1-phosphate uridylyltransferase	PA2023	29	97.45
1680436	1.709	1.0027885	null	probable ring-cleaving dioxygenase	PA2024	135	92.87
1680438	1.009	0.55151516	null	probable transcriptional regulator	PA2028	261	93.43
1680441	1.012	0.76208484	null	hypothetical protein	PA2029	5	92
1680443	1.039	2.2914286	null	hypothetical protein	PA2027	56	93.21
1680446	1.217	1.0576346	null	conserved hypothetical protein	PA2026	43	95.08
1680449	0.923	0.3535294	null	hypothetical protein	PA2030	1	95.15
1680452	1.372	0.9672595	null	hypothetical protein	PA2031	139	92.55
1680453	0.558	0.022256445	null	hypothetical protein	PA2036	113	92.46
1680456	0.725	0.72279036	null	hypothetical protein	PA2034	55	95.59
1680459	1.816	0.6869346	null	hypothetical protein	PA2037	109	94.15
1680462	1.218	0.6539817	fahA	fumarylacetoacetase	PA2008	31	93.8
1680465	1.009	1.3161517	null	hypothetical protein	PA2033	522	93.19
1680468	0.908	0.7487703	null	hypothetical protein	PA2038	1	92.15
1680471	1.286	0.8927407	null	hypothetical protein	PA2039	307	94.66
1680474	1.019	5.1193304	null	probable transcriptional regulator	PA2032	180	96.79
1680477	1.102	1.1552296	null	Resistance-Nodulation-Cell Division (RND)	PA2018	318	94.39
1680480	1.138	0.6587768	null	hypothetical protein	PA2043	72	95.51
1680483	0.976	0.74226725	null	probable transporter (membrane subunit)	PA2042	7	92.11
1680486	0.737	0.47325045	null	conserved hypothetical protein	PA2045	20	92.04
1680487	1.161	0.868824	gor	glutathione reductase	PA2025	442	92.38
1680490	3.055	2.9466665	null	probable decarboxylase	PA2035	57	92.21
1680493	0.786	0.16520165	null	probable amino acid permease	PA2041	15	93.77
1680496	1.009	0.59066665	null	hypothetical protein	PA2046	242	95.69
1680499	1.009	2.34	null	hypothetical protein	PA2048	78	93.91
1680501	2.561	1.1367196	metE	5-methyltetrahydropteroyltriglutamate-	PA1927	312	93.23
1680504	0.671	1.2456157	null	probable sigma-70 factor, ECF subfamily	PA2050	27	92.7
1680507	1.182	1.1085712	null	probable transcriptional regulator	PA2047	62	92.23
1680510	3.644	0.7737013	cynS	cyanate lyase	PA2052	375	92.05
1680513	0.630	-0.30933335	null	probable transmembrane sensor	PA2051	145	96.04
1680516	1.270	0.60033685	cynT	carbonate dehydratase	PA2053	43	97.26
1680519	1.072	0.91293633	null	probable transcriptional regulator	PA2056	174	97.25
1680522	0.684	0.50099653	rbsA	ribose transport protein RbsA	PA1947	283	95.45
1680525	1.009	0.855024	null	hypothetical protein	PA2057	65	92.43
1680528	0.880	0.6657873	null	hypothetical protein	PA2049	98	97.92
1680531	1.140	0.7607213	null	probable pyridoxal-phosphate dependent enzyme	PA2062	41	96.55
1680534	1.649	0.5383626	null	probable permease of ABC transporter	PA2060	451	97.87
1680537	0.706	0.43256956	null	probable transcriptional regulator	PA2010	45	94.02
1680540	0.395	0.6586716	null	hypothetical protein	PA2044	155	95.25
1680543	1.009	1.9324944	pcoB	copper resistance protein B precursor	PA2064	320	97.32
1680546	0.963	0.72373945	null	probable major facilitator superfamily (MFS)	PA2068	418	97.65
1680549	1.272	0.60940176	pcoA	copper resistance protein A precursor	PA2065	1	94.09
1680552	1.009	0.35921326	null	probable hydrolase	PA2067	554	94.18
1680555	1.279	0.98609173	null	probable glutamine synthetase	PA2040	647	93.16
1680558	0.845	0.38971215	null	probable permease of ABC transporter	PA2059	373	93.33
1680561	1.009	1.9371698	null	probable binding protein component of ABC	PA2058	94	92.42
1680564	1.009	3.0300148	null	hypothetical protein	PA2070	100	93.62
1680567	73.093	1.8820512	null	hypothetical protein	PA2074	1	93.75
1680570	0.521	-1.8685389	null	hypothetical protein	PA2063	67	94.43
1680573	1.246	0.6808943	null	probable carbamoyl transferase	PA2069	936	95.55
1680576	1.914	0.46731907	null	hypothetical protein	PA2075	31	92.54
1680579	1.009	0.0382635	null	probable major facilitator superfamily (MFS)	PA2055	219	96.98
1680582	0.759	-4.7191916	null	probable transporter (membrane subunit)	PA2073	35	92.04
1680585	0.887	0.5041084	null	probable amino acid permease	PA2079	687	97.44
1680588	1.421	1.3402153	null	conserved hypothetical protein	PA2072	1	92.83
1680591	0.614	0.3958392	null	probable transcriptional regulator	PA2076	259	92.61
1680594	0.869	0.7245596	kynB	kynurenine formamidase, KynB	PA2081	210	93.7
1680597	1.009	0.96369284	null	probable transcriptional regulator	PA2082	9	93.68
1680600	1.096	0.23464286	null	hypothetical protein	PA2078	671	97.99
1680603	0.355	-0.14589655	null	probable ring-hydroxylating dioxygenase subunit	PA2083	72	93.33
1680606	1.260	0.2456923	fusA2	elongation factor G	PA2071	62	94

1680609	1.009	0.82928574	null	hypothetical protein	PA2087	118	96.86
1680612	1.069	0.2966667	null	hypothetical protein	PA2088	176	96.81
1680615	1.080	0.9312253	null	probable epoxide hydrolase	PA2086	438	95.1
1680618	1.084	0.96161544	null	hypothetical protein	PA2090	167	97.98
1680621	1.022	0.9470714	kynU	hypothetical protein	PA2080	755	95.16
1680624	1.256	0.77382696	null	probable ring-hydroxylating dioxygenase small	PA2085	321	92.65
1680628	1.009	-1.3520927	null	probable sigma-70 factor, ECF subfamily	PA2093	359	94.94
1680630	0.876	-0.13669065	null	probable major facilitator superfamily (MFS)	PA2092	144	93.7
1680633	0.843	0.14099026	null	hypothetical protein	PA2095	259	92.46
1680636	1.571	1.1059377	null	probable transcriptional regulator	PA2096	221	95.85
1680639	1.129	3.5559907	null	probable transmembrane sensor	PA2094	135	96.07
1680642	0.777	0.056609333	null	hypothetical protein	PA2077	1	92.24
1680645	1.076	0.8615235	null	probable flavin-binding monooxygenase	PA2097	173	92.03
1680648	1.009	1.3192858	null	hypothetical protein	PA2066	232	95.33
1680651	0.827	-0.12818184	null	hypothetical protein	PA2102	51	92.49
1680654	1.009	1.2841667	null	probable transcriptional regulator	PA2100	43	92.64
1680657	1.025	-0.6190475	null	probable short-chain dehydrogenase	PA2099	300	94.43
1680660	1.281	0.5610197	null	probable asparagine synthetase	PA2084	263	97.53
1680663	2.779	1.258334	null	probable acetyltransferase	PA2105	4	95.43
1680666	1.137	0.7105001	null	probable cysteine synthase	PA2104	152	93.39
1680669	1.019	0.7956702	null	hypothetical protein	PA2106	122	94.99
1680673	1.047	-0.6706294	null	probable esterase/deacetylase	PA2098	787	95.47
1680675	1.141	0.5256177	null	conserved hypothetical protein	PA2101	90	94.21
1680678	1.397	0.9787286	null	hypothetical protein	PA2107	8	94.02
1680680	1.009	1.5912087	null	hypothetical protein	PA2109	152	92.84
1680683	1.009	-0.80070174	cynR	transcriptional regulator CynR	PA2054	347	93.23
1680686	1.761	1.3138901	null	probable molybdopterin biosynthesis protein	PA2103	1	92.92
1680690	1.349	0.8218362	null	hypothetical protein	PA2110	583	94
1680692	1.438	1.1666667	null	hypothetical protein	PA2089	1	93.61
1680695	1.088	0.81951106	null	probable major facilitator superfamily (MFS)	PA2114	31	93.46
1680698	0.948	0.2680155	null	hypothetical protein	PA2117	23	92.43
1680701	1.296	0.5627	null	hypothetical protein	PA2120	322	92.69
1680704	1.028	0.8285217	null	hypothetical protein	PA2111	163	95.09
1680707	1.448	1.0879164	null	alcohol dehydrogenase (Zn-dependent)	PA2119	37	95.36
1680710	1.009	1.495044	null	probable decarboxylase	PA2108	5	92.4
1680713	1.435	1.0423708	null	probable transcriptional regulator	PA2121	244	95.07
1680716	0.600	-5.220832	null	probable transcriptional regulator	PA2123	381	92.54
1680719	1.240	0.9633104	ada	O6-methylguanine-DNA methyltransferase	PA2118	1	93.62
1680722	1.079	0.7644838	null	conserved hypothetical protein	PA2126	94	96.71
1680725	1.438	0.56787884	null	probable transcriptional regulator	PA2115	56	93.04
1680728	1.082	0.8038982	null	hypothetical protein	PA2122	2	92.61
1680731	1.009	2.8316128	null	probable aldehyde dehydrogenase	PA2125	1	93.31
1680734	1.460	1.3182623	cupA1	fimbrial subunit CupA1	PA2128	505	93.7
1680737	1.242	0.9083826	null	conserved hypothetical protein	PA2127	3	93.76
1680740	1.009	2.1410508	cupA2	chaperone CupA2	PA2129	1	92.41
1680743	1.327	0.90242445	cupA5	chaperone CupA5	PA2132	372	96.89
1680746	0.862	0.70476186	cupA4	fimbrial subunit CupA4	PA2131	85	93.89
1680749	1.009	0.39000002	null	hypothetical protein	PA2133	149	94.77
1680752	0.886	0.4354941	null	conserved hypothetical protein	PA2116	117	92.66
1680755	1.146	0.25273627	null	conserved hypothetical protein	PA2112	665	97.62
1680758	1.009	0.07952382	null	hypothetical protein	PA2136	304	96.1
1680761	2.899	1.8424416	null	probable metallothionein	PA2140	2	93.56
1680763	1.799	0.9814561	null	hypothetical protein	PA2137	35	94.85
1680766	1.111	1.2861942	null	probable porin	PA2113	646	92.63
1680769	1.565	1.1254534	null	hypothetical protein	PA2139	29	94.23
1680770	2.177	0.40666667	null	hypothetical protein	PA2141	56	95.24
1680773	1.438	1.9517857	null	hypothetical protein	PA2143	128	95.23
1680774	0.759	0.027660813	null	probable dehydrogenase	PA2124	129	93.84
1680777	1.009	0.3659524	null	conserved hypothetical protein	PA2146	87	96.12
1680778	0.781	-0.3475	cupA3	usher CupA3	PA2130	69	94.72
1680781	1.009	1.2963889	null	hypothetical protein	PA2145	57	94.04
1680783	1.147	0.8881651	null	probable transporter	PA2135	284	95.15
1680786	1.009	1.0063026	null	hypothetical protein	PA2149	105	92.91
1680788	1.009	0.93913424	null	probable short-chain dehydrogenase	PA2142	199	92.43
1680791	1.009	1.1091721	null	conserved hypothetical protein	PA2150	124	92.6
1680794	1.009	-1.3675295	null	conserved hypothetical protein	PA2148	288	96.23
1680797	8.593	6.3271794	glgP	glycogen phosphorylase	PA2144	2	95.15
1680802	0.952	0.6837303	null	hypothetical protein	PA2091	1122	95.11
1680803	1.009	1.437143	null	probable ATP-dependent DNA ligase	PA2138	93	96.03
1680806	1.009	-1.7664633	null	conserved hypothetical protein	PA2154	114	93.46
1680809	1.037	0.8492692	null	conserved hypothetical protein	PA2151	35	94.52
1680812	1.009	2.55746	null	probable ATP-binding component of ABC	PA2061	536	94.65
1680815	1.009	0.8593116	null	hypothetical protein	PA2157	51	93.86
1680818	1.173	0.7349817	null	probable phospholipase	PA2155	44	92.67
1680823	1.009	1.7875223	null	conserved hypothetical protein	PA2159	2	92.32
1680824	1.889	2.8330398	null	conserved hypothetical protein	PA2156	117	94.42
1680827	1.009	1.2074604	katE	catalase HPII	PA2147	38	96.72
1680830	1.009	3.0729003	null	hypothetical protein	PA2161	19	92.4
1680831	6.513	2.3527553	null	probable glycosyl hydrolase	PA2160	61	94.92
1680834	1.111	1.4060606	null	probable glycosyl hydrolase	PA2164	171	96.87
1680837	1.249	0.9887096	null	probable solute-binding protein	PA1811	53	93.16
1680840	1.009	3.4672828	null	hypothetical protein	PA2168	528	92.38
1680845	1.347	0.80826914	null	hypothetical protein	PA2166	289	93.65
1680846	0.974	-0.20888889	null	hypothetical protein	PA2169	227	93.48
1680849	1.009	1.6335208	null	hypothetical protein	PA2171	208	92.68
1680851	0.412	-0.31179485	null	hypothetical protein	PA2173	166	94.58
1680852	1.047	-3.057143	null	hypothetical protein	PA2174	107	92.18
1680854	1.078	0.14821312	null	hypothetical protein	PA2170	34	93.85
1680856	0.855	0.14328691	null	hypothetical protein	PA2175	196	95.41
1680859	0.052	0.14119086	null	hypothetical protein	PA2172	54	95.65
1680862	1.089	0.8900474	null	probable glycosyl hydrolase	PA2162	44	93.79
1680866	1.061	0.8506592	null	hypothetical protein	PA2176	206	96.38
1680868	219.075	0.95699006	null	hypothetical protein	PA2178	3	92.31
1680871	1.009	2.3974025	glgB	1,4-alpha-glucan branching enzyme	PA2153	561	94.27
1680874	1.009	1.2384126	null	hypothetical protein	PA2182	176	93.23
1680876	1.009	1.3541666	null	hypothetical protein	PA2183	143	93.04
1680877	1.515	-6.005481	null	hypothetical protein	PA2181	49	92.67
1680880	0.539	-1.399072	null	hypothetical protein	PA2186	7	94.44
1680881	1.226	0.622372	null	probable major facilitator superfamily (MFS)	PA2006	789	92.8
1680884	1.009	1.5196339	null	conserved hypothetical protein	PA2184	119	93.48
1680887	0.210	1.2500808	null	hypothetical protein	PA2187	45	93.11
1680890	0.851	0.7814154	null	hypothetical protein	PA2180	1	94.77
1680893	1.009	0.7412981	katN	non-heme catalase KatN	PA2185	486	92.44
1680896	0.878	0.022424484	null	hypothetical protein	PA2179	53	94.19
1680899	2.051	0.09420955	null	hypothetical protein	PA2189	44	92.38
1680902	1.009	4.329861	null	hypothetical protein	PA2167	248	93.81
1680905	0.244	0.8927428	null	probable trehalose synthase	PA2152	215	94.27
1680909	1.009	-4.0768986	null	conserved hypothetical protein	PA2190	257	93.93
1680911	0.923	0.10968256	null	conserved hypothetical protein	PA2192	310	94.24

1680913	0.653	0.2240061	hcnA	hydrogen cyanide synthase HcnA	PA2193	16	92.01
1680916	0.593	-0.36298704	exoY	adenylate cyclase ExoY	PA2191	11	92.64
1680919	1.370	1.1268978	null	hypothetical protein	PA2198	268	94.08
1680921	0.629	-1.0413768	hcnB	hydrogen cyanide synthase HcnB	PA2194	294	94.93
1680924	1.311	0.90662974	null	probable transcriptional regulator	PA2196	45	93.14
1680927	0.721	0.09678225	null	conserved hypothetical protein	PA2197	61	92.26
1680930	1.306	0.8786646	null	probable amino acid permease	PA2202	243	92.42
1680933	0.883	0.69751185	null	probable dehydrogenase	PA2199	583	92.65
1680936	1.008	0.7543137	null	hypothetical protein	PA2205	401	92.6
1680939	0.846	0.43157953	null	hypothetical protein	PA2163	596	96.71
1680942	0.247	0.7400011	null	probable amino acid permease	PA2203	250	96.24
1680945	0.685	0.50110435	null	conserved hypothetical protein	PA2200	234	95.18
1680948	0.523	0.39730445	null	probable binding protein component of ABC	PA2204	86	97.79
1680951	1.009	1.0108225	null	hypothetical protein	PA2201	570	93.8
1680954	1.227	4.952321	null	conserved hypothetical protein	PA2211	68	95.4
1680957	2.042	0.74608976	null	hypothetical protein	PA2208	324	92.5
1680960	9.160	-3.7105262	null	probable sensor/response regulator hybrid	PA2177	40	96.94
1680963	1.009	1.1570411	null	probable glycogen synthase	PA2165	79	92.78
1680966	1.280	1.0732085	null	hypothetical protein	PA2207	26	95.79
1680969	0.911	0.111685395	null	probable transcriptional regulator	PA2206	368	92.25
1680972	0.849	4.5406504	null	conserved hypothetical protein	PA2212	277	96.74
1680975	1.009	0.7595238	null	hypothetical protein	PA2134	119	92.75
1680978	1.078	0.95693904	null	hypothetical protein	PA2209	87	96.68
1680981	0.968	0.7328978	null	probable porin	PA2213	4	96.13
1680984	1.209	1.9026655	null	hypothetical protein	PA2222	154	92.57
1680985	1.076	0.7714286	null	conserved hypothetical protein	PA2221	1	93.2
1680988	0.963	0.16225031	null	hypothetical protein	PA2218	2	93.11
1680991	1.009	0.068530485	opdE	membrane protein OpdE	PA2219	7	93.04
1680994	2.762	1.3912742	null	hypothetical protein	PA2223	65	92.39
1680997	1.116	0.13600287	null	probable major facilitator superfamily (MFS)	PA2210	19	97.01
1681000	1.609	1.1398213	null	hypothetical protein	PA2225	39	93.92
1681003	1.009	0.78474855	null	conserved hypothetical protein	PA2216	321	94.62
1681006	0.986	0.5436601	null	probable aldehyde dehydrogenase	PA2217	94	95.19
1681009	0.549	0.50694805	null	hypothetical protein	PA2224	226	92.06
1681011	1.117	0.60200304	null	probable transcriptional regulator	PA2227	758	93.16
1681012	1.003	0.76269805	null	probable transcriptional regulator	PA2220	6	92.39
1681015	1.009	-1.6256536	null	hypothetical protein	PA2215	82	92.26
1681018	1.009	1.91625	null	hypothetical protein	PA2228	448	92.61
1681021	0.992	0.4702734	null	conserved hypothetical protein	PA2229	153	93.94
1681024	1.066	0.57765436	null	hypothetical protein	PA2230	6	92.81
1681027	1.009	-2.0174298	null	probable alcohol dehydrogenase (Zn-dependent)	PA2188	1	94.46
1681030	1.081	0.86638385	pslA	probable glycosyl transferase	PA2231	23	92
1681033	1.302	0.97675955	null	probable major facilitator superfamily (MFS)	PA2214	315	92.65
1681036	1.025	-2.9570508	pslD	probable exopolysaccharide transporter	PA2234	57	96.65
1681039	1.101	0.6601221	pslL	hypothetical protein	PA2242	7	93.04
1681042	0.652	0.19436005	pslH	hypothetical protein	PA2238	326	92.4
1681045	1.065	1.2430403	pslO	hypothetical protein	PA2245	23	95.13
1681048	0.610	1.7602012	pslK	hypothetical protein	PA2241	1137	96.47
1681051	1.210	0.6235287	pslM	hypothetical protein	PA2243	1	94.11
1681054	1.138	0.90633994	pslB	probable phosphomannose isomerase/GDP-mannose	PA2232	1	96.92
1681057	1.018	0.761143	hcnC	hydrogen cyanide synthase HcnC	PA2195	157	94.67
1681060	1.093	1.062425	bkdA1	2-oxoisovalerate dehydrogenase (alpha subunit)	PA2247	1	92.55
1681063	1.339	1.0190456	bkdA2	2-oxoisovalerate dehydrogenase (beta subunit)	PA2248	3	92.06
1681066	1.055	0.81681836	bkdR	transcriptional regulator BkdR	PA2246	290	92.09
1681069	0.593	0.57521474	pslF	hypothetical protein	PA2236	721	92.49
1681072	1.007	0.2880432	pslN	hypothetical protein	PA2244	178	95.23
1681075	1.009	1.8851492	null	hypothetical protein	PA2251	48	92.66
1681078	1.473	1.1256638	pvcA	pyoverdine biosynthesis protein PvcA	PA2254	45	93.02
1681081	1.348	0.98121345	null	probable AGCS sodium/alanine/glycine symporter	PA2252	60	92.23
1681084	1.440	0.9852241	pvcB	pyoverdine biosynthesis protein PvcB	PA2255	2	92.49
1681087	0.866	0.5860454	pslE	hypothetical protein	PA2235	151	92.29
1681090	1.187	0.8848537	bkdB	branched-chain alpha-keto acid dehydrogenase	PA2249	54	92.22
1681093	1.904	1.3319048	pvcC	pyoverdine biosynthesis protein PvcC	PA2256	426	93.94
1681096	2.801	0.59572256	ptxR	transcriptional regulator PtxR	PA2258	150	95.37
1681099	1.310	1.1328765	ptxS	transcriptional regulator PtxS	PA2259	3	92.78
1681102	1.009	-0.99530554	pvcD	pyoverdine biosynthesis protein PvcD	PA2257	215	93.04
1681105	0.851	0.08814169	pslG	probable glycosyl hydrolase	PA2237	158	92.36
1681108	1.457	1.1514975	null	probable 2-ketogluconate transporter	PA2262	430	93.02
1681111	1.118	0.9222808	null	hypothetical protein	PA2260	2	92.22
1681114	0.962	0.78220046	ansA	L-asparaginase I	PA2253	589	96.81
1681117	0.974	0.6779365	null	probable 2-ketogluconate kinase	PA2261	9	92.1
1681120	0.972	0.44228512	null	probable transcriptional regulator	PA2267	14	93.25
1681123	1.035	-0.5744444	null	probable 2-hydroxyacid dehydrogenase	PA2263	510	92.56
1681126	1.194	0.73555154	null	conserved hypothetical protein	PA2264	118	93.87
1681130	0.872	0.6254293	null	probable acetyltransferase	PA2271	337	96.72
1681133	1.115	0.916407	null	probable transcriptional regulator	PA2273	351	97.64
1681135	1.394	1.1670624	null	gluconate dehydrogenase	PA2265	116	93.18
1681138	1.075	0.7412907	null	hypothetical protein	PA2268	279	96.28
1681141	0.447	-0.43737158	pbpC	penicillin-binding protein 3A	PA2272	74	92.33
1681145	0.895	0.5873641	null	probable transcriptional regulator	PA2270	243	96.2
1681147	1.009	1.1994247	null	hypothetical protein	PA2274	12	94.69
1681150	1.176	1.2114956	pslI	probable transferase	PA2239	379	92.31
1681153	1.031	5.325	null	conserved hypothetical protein	PA2280	190	94.59
1681156	1.093	0.8160062	arsC	ArsC protein	PA2279	34	94.43
1681159	1.031	0.59763575	null	conserved hypothetical protein	PA2269	96	92
1681162	1.293	0.7310054	null	probable transcriptional regulator	PA2276	119	95.08
1681165	1.923	-6.206954	null	probable alcohol dehydrogenase (Zn-dependent)	PA2275	89	92.71
1681168	1.201	0.728335	arsR	ArsR protein	PA2277	182	95.19
1681170	1.367	0.6752381	null	hypothetical protein	PA2282	172	95.09
1681173	1.994	0.24046731	arsB	ArsB protein	PA2278	462	92.35
1681176	1.168	0.844442	null	hypothetical protein	PA2288	66	97.22
1681177	1.371	1.1058524	null	hypothetical protein	PA2284	365	92.92
1681180	1.159	0.8285821	null	probable transcriptional regulator	PA2281	168	94.29
1681183	1.226	1.0985553	null	hypothetical protein	PA2285	1	94.18
1681186	1.333	0.9873274	null	hypothetical protein	PA2287	356	94.1
1681189	1.108	0.96008813	gcd	glucose dehydrogenase	PA2290	215	96.7
1681192	1.662	0.28572312	null	hypothetical protein	PA2292	218	92.55
1681194	1.305	0.98368293	null	conserved hypothetical protein	PA2289	206	94.59
1681197	1.009	1.674609	null	probable alcohol dehydrogenase (Zn-dependent)	PA2158	390	93.24
1681200	0.671	10.019411	null	probable ferredoxin	PA2297	162	92.62
1681202	1.104	0.8432293	null	probable permease of ABC transporter	PA2295	340	93.75
1681205	1.035	1.2982538	null	hypothetical protein	PA2286	667	94.65
1681208	1.133	0.6391234	null	hypothetical protein	PA2283	2	92.38
1681211	1.009	1.4900253	null	probable ATP-binding component of ABC	PA2294	207	97.46
1681214	1.174	0.95267373	null	probable cytochrome c precursor	PA2266	444	95.93
1681217	1.204	1.1866766	null	probable transcriptional regulator	PA2299	1	94.15
1681220	1.324	-0.016310167	chiC	chitinase	PA2300	214	94.58
1681224	0.458	0.29005584	null	hypothetical protein	PA2301	483	94.61
1681225	1.801	0.701816	null	hypothetical protein	PA2293	197	96.78

1681228	1.262	0.69407564	null	hypothetical protein	PA2303	179	95.15
1681231	1.073	0.8496208	null	probable glucose-sensitive porin	PA2291	151	92.37
1681234	0.924	3.305678	null	conserved hypothetical protein	PA2306	44	92.9
1681237	1.009	3.882381	null	hypothetical protein	PA2296	188	94.02
1681240	0.533	0.3518706	null	hypothetical protein	PA2311	1	93.44
1681241	0.556	0.4853882	null	probable non-ribosomal peptide synthetase	PA2305	6	92.14
1681244	0.992	0.40036845	null	hypothetical protein	PA2304	348	92.34
1681247	0.905	0.72581875	pslC	probable glycosyl transferase	PA2233	454	92.6
1681250	0.319	0.2385687	null	hypothetical protein	PA2310	22	97.8
1681253	3.051	1.3207264	null	hypothetical protein	PA2313	242	93.3
1681256	1.076	-0.2722222	null	hypothetical protein	PA2309	192	93.89
1681259	0.893	0.9913982	null	probable permease of ABC transporter	PA2307	434	93.2
1681262	1.100	0.6735179	null	hypothetical protein	PA2318	164	92.93
1681264	1.565	1.8482202	null	probable ATP-binding component of ABC	PA2308	256	95.97
1681267	1.432	1.1010634	null	gluconokinase	PA2321	1	94.88
1681271	1.545	1.095269	null	probable major facilitator superfamily (MFS)	PA2314	183	94.99
1681273	0.849	0.7146858	null	probable transposase	PA2319	45	92.38
1681276	0.566	0.8060533	gntR	transcriptional regulator GntR	PA2320	9	92.53
1681279	1.161	0.99744415	null	hypothetical protein	PA2324	1	96.21
1681282	0.637	0.449534	null	probable transcriptional regulator	PA2312	178	94.1
1681285	0.790	0.6717953	null	probable glyceraldehyde-3-phosphate	PA2323	1	94.53
1681288	1.009	-0.6533333	null	probable oxidoreductase	PA2317	312	92.51
1681291	0.887	1.2334616	null	hypothetical protein	PA2315	748	95.39
1681294	1.009	1.3313978	null	hypothetical protein	PA2325	170	93.77
1681297	1.052	2.2549708	null	probable permease of ABC transporter	PA2327	154	92.42
1681300	0.907	-0.46023807	null	probable ATP-binding component of ABC	PA2329	613	95.68
1681303	0.621	1.1580882	null	hypothetical protein	PA2326	15	92.55
1681306	0.862	-0.12924497	null	probable non-ribosomal peptide synthetase	PA2302	1	94.13
1681309	0.736	-2.1983366	null	probable sulfatase	PA2333	16	92.21
1681312	0.503	0.32384175	null	probable transcriptional regulator	PA2334	348	92.35
1681315	1.426	-1.2266319	null	probable transcriptional regulator	PA2332	39	92.6
1681318	1.009	1.221922	null	hypothetical protein	PA2328	816	93.98
1681321	4.787	2.8324773	mtlR	transcriptional regulator MtlR	PA2337	2	92.19
1681324	0.459	-0.85738623	null	hypothetical protein	PA2330	239	92.61
1681327	1.700	1.1576754	null	hypothetical protein	PA2331	507	93.17
1681330	1.499	1.0582528	null	probable binding protein component of ABC	PA2338	667	93.66
1681333	1.309	0.9417534	null	probable binding-protein-dependent	PA2339	361	93.74
1681336	0.830	0.3992639	null	hypothetical protein	PA2336	336	95.63
1681339	1.141	0.9191154	null	probable TonB-dependent receptor	PA2335	1	96.43
1681342	1.241	0.96071273	null	probable binding-protein-dependent	PA2340	161	93.11
1681345	1.074	0.5946859	mtlZ	fructokinase	PA2344	9	92.32
1681348	0.934	0.10105262	null	probable ATP-binding component of ABC	PA2341	1029	93.68
1681351	1.173	1.2697828	null	conserved hypothetical protein	PA2345	100	94.83
1681354	1.009	0.31566963	null	conserved hypothetical protein	PA2349	289	92.63
1681357	1.133	0.5822222	null	conserved hypothetical protein	PA2346	1	93.74
1681360	1.009	0.86642826	mtlY	xylulose kinase	PA2343	15	94.52
1681363	0.768	-0.9889706	null	gluconate permease	PA2322	415	95.05
1681366	1.298	0.7077901	mtlD	mannitol dehydrogenase	PA2342	543	93.95
1681370	0.766	0.24187064	null	probable ATP-binding component of ABC	PA2350	190	95.79
1681372	1.009	0.050961494	null	probable permease of ABC transporter	PA2351	5	92.12
1681375	1.078	1.1515516	null	probable glycerophosphoryl diester	PA2352	489	94.85
1681378	0.853	0.37545747	null	probable oxidoreductase	PA2298	141	94.19
1681382	1.042	0.79285717	null	hypothetical protein	PA2347	840	93.47
1681384	0.918	0.79560596	null	hypothetical protein	PA2358	1	96.98
1681387	1.009	1.6390194	pslJ	hypothetical protein	PA2240	185	93.11
1681390	0.833	0.46802807	msuE	NADH-dependent FMN reductase MsuE	PA2357	384	95.22
1681393	1.374	1.2177981	null	probable transcriptional regulator	PA2316	1	94.31
1681396	1.100	0.98110247	null	conserved hypothetical protein	PA2353	199	92.68
1681399	2.041	0.8900732	msuD	methanesulfonate sulfonatase MsuD	PA2356	61	97.83
1681402	0.948	0.7188125	null	probable transcriptional regulator	PA2354	1	93.17
1681405	1.007	0.24800654	null	probable transcriptional regulator	PA2359	639	97.7
1681408	0.829	0.27233464	null	hypothetical protein	PA2362	147	96.97
1681411	1.340	1.0405604	null	hypothetical protein	PA2364	290	95.36
1681414	0.429	0.60363257	null	hypothetical protein	PA2367	139	94.19
1681416	1.221	0.07470757	null	conserved hypothetical protein	PA2348	89	96.07
1681419	1.091	0.81559145	null	conserved hypothetical protein	PA2366	21	93.62
1681422	1.009	1.6226294	null	hypothetical protein	PA2372	56	92.3
1681424	0.051	2.6964285	null	probable FMNH2-dependent monooxygenase	PA2355	220	93.33
1681427	1.231	3.2172222	null	hypothetical protein	PA2363	2	93.06
1681430	0.416	-2.010266	null	hypothetical protein	PA2375	109	93.81
1681433	1.065	1.3012409	null	hypothetical protein	PA2369	522	95.06
1681436	1.258	1.0079606	null	hypothetical protein	PA2374	150	95.26
1681439	1.250	0.94058734	null	hypothetical protein	PA2368	341	94.84
1681441	1.009	0.24339399	null	hypothetical protein	PA2370	262	96.03
1681444	0.994	-0.08942946	null	probable ClpA/B-type protease	PA2371	61	92.18
1681447	1.132	0.6978283	null	hypothetical protein	PA2380	49	93.65
1681449	0.755	2.3701262	null	probable oxidoreductase	PA2379	35	92.01
1681452	1.009	1.32533	null	hypothetical protein	PA2384	55	94.88
1681454	1.140	0.99214613	null	probable transcriptional regulator	PA2376	67	97.94
1681457	0.586	-0.23434606	null	probable transcriptional regulator	PA2383	359	92.51
1681460	1.976	0.8656863	lldA	L-lactate dehydrogenase	PA2382	49	94.48
1681463	1.205	0.84574956	pvdA	L-ornithine N5-oxygenase	PA2386	20	94.21
1681466	0.912	0.13726068	null	probable transmembrane sensor	PA2388	19	95.75
1681469	1.922	1.7432575	null	probable sigma-70 factor, ECF subfamily	PA2387	21	94.96
1681472	1.544	1.1163425	null	hypothetical protein	PA2381	101	96.18
1681475	1.221	-0.3107359	null	hypothetical protein	PA2377	53	92.62
1681478	1.009	0.9080952	null	hypothetical protein	PA2361	76	95.29
1681481	0.370	1.9707407	pvdQ	PvdQ	PA2385	274	93.45
1681484	0.688	0.4587615	null	probable aldehyde dehydrogenase	PA2378	2	92.04
1681487	1.009	0.38058037	pvdF	pyoverdine synthetase F	PA2396	10	92.26
1681490	0.989	0.32349828	opmQ	probable outer membrane protein precursor	PA2391	312	95.64
1681493	0.866	0.9004852	null	conserved hypothetical protein	PA2373	1	95.58
1681496	1.123	0.9963337	pvdO	PvdO	PA2395	483	96.29
1681499	1.090	0.53305674	fpvA	ferripyoverdine receptor	PA2398	43	93
1681502	0.349	1.1643275	null	conserved hypothetical protein	PA2389	71	92.85
1681505	1.108	0.9251334	null	probable dipeptidase precursor	PA2393	1	92.42
1681508	1.665	0.8914714	null	hypothetical protein	PA2403	8	92.3
1681511	1.375	0.8584936	pvdE	pyoverdine biosynthesis protein PvdE	PA2397	36	95.58
1681514	1.145	0.73914796	null	hypothetical protein	PA2404	51	92.06
1681517	0.980	0.51779664	lpdV	lipoamide dehydrogenase-Val	PA2250	345	96.7
1681520	0.831	-2.5526137	null	hypothetical protein	PA2406	106	96.53
1681523	1.272	0.91131574	pvdP	PvdP	PA2392	1	93.79
1681526	0.977	0.8310604	pvdN	PvdN	PA2394	154	92.67
1681529	0.795	0.5335798	null	hypothetical protein	PA2410	593	93.28
1681532	0.909	0.35	null	probable ATP-binding component of ABC	PA2408	288	95.33
1681535	0.913	0.8878797	null	conserved hypothetical protein	PA2365	191	92.52
1681538	1.009	0.3387943	null	conserved hypothetical protein	PA2412	132	92.67
1681539	1.009	0.8378317	null	hypothetical protein	PA2405	67	95.56
1681541	1.202	0.86005247	null	probable adhesion protein	PA2407	781	92.04

1681544	0.428	2.9727967	pvdJ	PvdJ	PA2400	12	95.8
1681547	1.254	0.52205884	null	hypothetical protein	PA2418	96	93.81
1681550	1.009	2.3202507	pvdH	L-2,4-diaminobutyrate:2-ketoglutarate	PA2413	318	93.25
1681553	0.838	0.50478065	null	L-sorbosone dehydrogenase	PA2414	110	94.94
1681558	1.336	0.96224415	pvdD	pyoverdine synthetase D	PA2399	190	94.09
1681559	1.260	0.4200039	null	probable thioesterase	PA2411	523	97.5
1681562	1.009	-0.027522307	null	hypothetical protein	PA2360	330	96.72
1681565	0.165	0.19435157	null	probable transcriptional regulator	PA2417	167	94.35
1681568	0.996	0.9493097	null	hypothetical protein	PA2422	57	96.51
1681571	1.058	0.63908184	null	probable ATP-binding/permease fusion ABC	PA2390	277	92.04
1681574	1.163	1.890818	null	hypothetical protein	PA2421	128	93.79
1681577	2.192	1.3340366	null	hypothetical protein	PA2427	254	95.48
1681580	0.042	1.0513208	null	hypothetical protein	PA2423	58	92.11
1681583	1.323	0.8995722	null	hypothetical protein	PA2429	64	93.6
1681585	1.009	1.9307938	null	hypothetical protein	PA2428	29	93.06
1681588	1.954	1.4450006	null	probable hydrolase	PA2419	260	93.87
1681591	0.978	0.40222222	null	hypothetical protein	PA2433	76	96.01
1681593	1.189	-0.10554962	null	probable porin	PA2420	24	95.75
1681596	1.009	1.597285	null	probable transcriptional regulator	PA2432	478	96.88
1681599	19.206	10.806289	null	conserved hypothetical protein	PA2430	58	97.13
1681602	1.018	0.7610439	null	hypothetical protein	PA2436	204	93.25
1681605	1.009	1.6333334	null	probable non-ribosomal peptide synthetase	PA2402	82	92.52
1681608	0.752	0.06916509	null	hypothetical protein	PA2437	391	92.36
1681611	0.810	0.6432174	pvdS	sigma factor PvdS	PA2426	22	92.09
1681614	1.009	-1.4314655	null	hypothetical protein	PA2441	1	95.16
1681617	1.501	1.0269514	null	hypothetical protein	PA2438	1	93.29
1681620	2.186	2.488528	null	hypothetical protein	PA2431	390	92.69
1681623	3.708	7.11173	null	hypothetical protein	PA2440	3	92.58
1681626	0.410	1.1069435	sdaA	L-serine dehydratase	PA2443	85	97.98
1681629	1.078	0.5625	null	probable transcriptional regulator	PA2447	371	93
1681632	1.669	2.9916668	null	hypothetical protein	PA2434	237	93.8
1681635	0.760	-0.71828705	gcvH2	glycine cleavage system protein H2	PA2446	3	93.06
1681638	1.092	0.9327833	gcvP2	glycine cleavage system protein P2	PA2445	130	94.05
1681641	1.417	1.5171788	pvdL	PvdL	PA2424	208	95.59
1681644	1.093	2.561111	null	hypothetical protein	PA2451	191	94.27
1681648	0.800	0.004809833	glyA2	serine hydroxymethyltransferase	PA2444	63	93.21
1681650	1.007	0.9584099	null	hypothetical protein	PA2453	1	94.69
1681652	1.009	1.4968688	null	hypothetical protein	PA2448	366	93.64
1681655	0.997	0.7660257	null	hypothetical protein	PA2456	109	92.2
1681656	0.942	0.66691893	null	hypothetical protein	PA2452	300	96.23
1681659	0.043	2.3197122	null	hypothetical protein	PA2457	84	92.42
1681662	1.199	0.9002423	pvdG	PvdG	PA2425	3	92.52
1681665	1.024	0.53564537	null	hypothetical protein	PA2450	1	95.02
1681668	1.031	0.2580313	null	hypothetical protein	PA2455	52	92.56
1681671	1.009	-3.217607	gcvT2	glycine cleavage system protein T2	PA2442	143	93.91
1681674	0.849	0.59167165	null	hypothetical protein	PA2454	474	92.33
1681677	0.986	0.7505795	null	probable transcriptional regulator	PA2449	1	92.4
1681680	1.009	1.5482353	null	hypothetical protein	PA2458	1	92.24
1681684	0.709	0.5035977	null	hypothetical protein	PA2463	1010	92.7
1681686	1.320	0.97444785	null	hypothetical protein	PA2465	27	93.55
1681689	1.042	1.4331169	null	hypothetical protein	PA2462	1404	95.88
1681692	1.136	0.98241174	null	probable transcriptional regulator	PA2469	83	97.93
1681695	0.699	0.4224442	null	probable transmembrane sensor	PA2467	162	95.18
1681698	1.082	-0.6544231	null	conserved hypothetical protein	PA2471	1	92.59
1681701	1.054	0.91033316	null	probable TonB-dependent receptor	PA2466	6	96.34
1681704	1.065	0.7818182	treA	periplasmic trehalase precursor	PA2416	186	94.62
1681709	1.111	5.361212	null	probable glutathione S-transferase	PA2473	198	95.49
1681710	1.009	0.6731868	null	hypothetical protein	PA2474	123	92.33
1681713	1.138	0.815506	null	probable major facilitator superfamily (MFS)	PA2472	64	92.84
1681718	1.031	-3.336863	null	hypothetical protein	PA2439	1296	92.45
1681719	1.486	0.9560612	null	probable sigma-70 factor, ECF subfamily	PA2468	1	95.82
1681722	0.945	0.6919535	null	probable cytochrome P450	PA2475	76	92.02
1681725	1.009	2.6904762	gtdA	gentisate 1,2-dioxygenase	PA2470	533	92.95
1681728	0.610	0.5067742	null	probable thiol:disulfide interchange protein	PA2477	541	93.18
1681731	1.009	0.43506497	null	probable two-component response regulator	PA2479	433	97.39
1681734	1.106	0.93587077	null	probable cytochrome c	PA2482	233	94.96
1681737	1.009	0.90370405	null	probable thiol:disulfide interchange protein	PA2478	82	95.08
1681741	1.018	0.81679916	null	hypothetical protein	PA2464	1	95.95
1681743	0.623	0.48689857	null	hypothetical protein	PA2460	76	97.28
1681744	1.220	0.9995383	null	hypothetical protein	PA2481	189	92.34
1681747	1.404	1.3065544	null	hypothetical protein	PA2485	84	92.89
1681749	1.216	0.87657166	null	conserved hypothetical protein	PA2484	132	94.05
1681752	9.078	7.6809907	null	hypothetical protein	PA2486	1	96.79
1681754	1.100	5.538249	null	hypothetical protein	PA2487	1	93.14
1681756	10.795	14.868254	null	probable oxidoreductase	PA2491	22	92.18
1681759	1.039	-5.9546824	null	probable cation-transporting P-type ATPase	PA2435	958	94.15
1681762	1.258	0.44208378	null	conserved hypothetical protein	PA2490	56	93.39
1681764	1.305	0.9786679	null	conserved hypothetical protein	PA2483	275	96.31
1681767	1.243	0.6994856	mexT	transcriptional regulator MexT	PA2492	83	93.03
1681770	9.017	7.9336004	oprN	Multidrug efflux outer membrane protein OprN	PA2495	27	96.7
1681773	1.635	1.7894493	mexF	Resistance-Nodulation-Cell Division (RND)	PA2494	53	92.85
1681776	0.585	0.50011265	null	hypothetical protein	PA2496	356	92.3
1681779	1.349	0.5382623	null	probable transcriptional regulator	PA2488	162	96.22
1681782	0.962	0.47350255	null	probable transcriptional regulator	PA2497	4	92.12
1681785	0.985	0.8479973	null	hypothetical protein	PA2501	82	92.92
1681786	0.965	1.0669508	null	conserved hypothetical protein	PA2498	357	96.24
1681789	1.009	0.6549373	null	probable deaminase	PA2499	111	96.58
1681791	1.089	0.7213509	null	hypothetical protein	PA2504	29	92.16
1681794	61.360	32.184677	mexE	Resistance-Nodulation-Cell Division (RND)	PA2493	107	92.87
1681797	1.039	0.7548077	null	probable porin	PA2505	78	92.44
1681800	1.052	0.9788059	null	hypothetical protein	PA2506	64	94.03
1681801	1.009	3.116883	null	probable two-component sensor	PA2480	205	94.6
1681804	1.009	0.7169643	catC	muconolactone delta-isomerase	PA2508	8	92.42
1681807	1.283	-0.002857685	null	probable major facilitator superfamily (MFS)	PA2500	488	95.72
1681810	1.009	0.95277774	catR	transcriptional regulator CatR	PA2510	88	94.52
1681813	0.915	-3.581905	null	hypothetical protein	PA2502	29	93.22
1681816	1.608	0.96501446	antB	anthranilate dioxygenase small subunit	PA2513	153	92.9
1681819	1.009	1.8756756	catA	catechol 1,2-dioxygenase	PA2507	14	92.58
1681822	0.478	-0.049519397	null	hypothetical protein	PA2503	260	94.05
1681825	1.157	0.9365193	antC	anthranilate dioxygenase reductase	PA2514	488	93.72
1681828	0.263	0.06407672	null	probable transcriptional regulator	PA2511	414	92.24
1681831	1.009	2.4627707	xylL	cis-1,2-dihydroxycyclohexa-3,4-diene carboxylate	PA2515	613	92.32
1681834	1.208	1.2528538	xylY	toluate 1,2-dioxygenase beta subunit	PA2517	307	95.52
1681837	1.009	1.5581685	czcA	Resistance-Nodulation-Cell Division (RND)	PA2520	72	95.39
1681840	1.093	1.2515874	xylX	toluate 1,2-dioxygenase alpha subunit	PA2518	129	93.44
1681843	1.009	1.8692858	catB	muconate cycloisomerase I	PA2509	5	92.18
1681846	1.009	1.2405901	czcC	outer membrane protein precursor CzcC	PA2522	260	96.18
1681849	1.009	0.68908817	antA	anthranilate dioxygenase large subunit	PA2512	49	94.65
1681852	0.981	-0.79053026	null	probable two-component response regulator	PA2523	496	92.06

1681855	1.028	1.5869048	xylZ	toluate 1,2-dioxygenase electron transfer	PA2516	590	93.73
1681858	1.305	5.3368483	null	probable Resistance-Nodulation-Cell Division	PA2527	199	92.08
1681861	0.971	0.17105034	null	probable two-component sensor	PA2524	1178	96.9
1681864	1.050	0.7747203	null	probable Resistance-Nodulation-Cell Division	PA2528	3	92.2
1681867	0.510	-0.004365064	null	probable outer membrane protein precursor	PA2525	201	93.64
1681870	1.446	1.0065079	null	probable aminotransferase	PA2531	663	92.82
1681873	0.958	0.22678363	null	hypothetical protein	PA2529	31	94.8
1681876	1.216	1.0541532	tpx	thiol peroxidase	PA2532	134	94.52
1681879	1.579	0.63937277	xylS	transcriptional regulator XylS	PA2519	25	97.01
1681882	63.466	-0.36507934	null	probable oxidoreductase	PA2535	116	92.55
1681885	1.009	0.8357895	null	probable transcriptional regulator	PA2534	233	96.54
1681888	0.266	-0.108095646	null	hypothetical protein	PA2530	486	92.25
1681891	0.694	0.41200933	null	probable acyltransferase	PA2537	76	92.32
1681894	0.919	0.5073414	null	probable sodium:alanine symporter	PA2533	3	92.58
1681897	1.314	0.54491174	czcB	Resistance-Nodulation-Cell Division (RND)	PA2521	527	94.61
1681900	1.025	0.7939251	null	conserved hypothetical protein	PA2539	123	92.7
1681903	0.943	-0.9155556	null	hypothetical protein	PA2538	240	96.84
1681907	0.757	0.655392	null	probable CDP-alcohol phosphatidyltransferase	PA2541	508	95.85
1681909	1.173	-0.7084733	xthA	exodeoxyribonuclease III	PA2545	143	95.31
1681912	0.999	0.7824887	null	probable phosphatidate cytidylyltransferase	PA2536	141	92.98
1681915	0.907	0.78966266	null	hypothetical protein	PA2544	74	92.14
1681918	1.280	1.0559763	null	conserved hypothetical protein	PA2542	3	92.61
1681921	0.751	-3.620612	null	probable ring-cleaving dioxygenase	PA2546	227	92.98
1681924	1.525	1.111488	null	hypothetical protein	PA2548	21	94.38
1681927	1.173	0.50931114	null	probable Resistance-Nodulation-Cell Division	PA2526	98	96.28
1681930	1.015	0.84809464	null	probable transcriptional regulator	PA2551	85	94.84
1681933	1.115	0.83298	null	probable acyl-CoA thiolase	PA2553	528	97.73
1681936	1.169	0.8256341	null	probable transcriptional regulator	PA2547	6	92.89
1681939	1.174	0.5530902	null	probable AMP-binding enzyme	PA2555	1	97.05
1681942	1.226	0.848735	null	conserved hypothetical protein	PA2543	379	96.31
1681945	1.039	-1.8695238	null	probable short-chain dehydrogenase	PA2554	279	92.63
1681948	0.476	0.19334519	null	probable transcriptional regulator	PA2556	4	93.28
1681951	1.129	-1.6348801	null	probable acyl-CoA dehydrogenase	PA2550	114	94.58
1681954	1.191	0.9493868	null	conserved hypothetical protein	PA2540	351	96.94
1681957	1.763	1.122987	null	probable transport protein	PA2558	66	96.35
1681960	1.807	0.91345376	null	probable acyl-CoA dehydrogenase	PA2552	238	93.11
1681963	0.053	2.7083652	null	hypothetical protein	PA2564	93	94.76
1681966	1.185	0.31102306	null	hypothetical protein	PA2560	1	93.45
1681969	0.775	0.21419291	null	hypothetical protein	PA2559	188	92.26
1681972	0.970	0.6482982	null	conserved hypothetical protein	PA2549	403	92.24
1681975	0.996	0.77034074	null	hypothetical protein	PA2568	32	95.51
1681978	3.304	3.4324276	null	conserved hypothetical protein	PA2566	335	95.42
1681981	1.300	1.0198967	null	hypothetical protein	PA2562	94	92.56
1681984	1.009	0.8929073	null	hypothetical protein	PA2569	74	92.06
1681987	1.298	0.79792434	pa1L	PA-I galactophilic lectin	PA2570	146	92.94
1681989	1.339	0.91136944	null	probable two-component sensor	PA2571	10	94.65
1681994	1.770	1.3311903	null	hypothetical protein	PA2565	290	96.92
1681995	1.928	-7.815507	null	probable chemotaxis transducer	PA2561	48	95.06
1681998	1.919	1.5164647	alkB1	alkane-1-monooxygenase	PA2574	66	93.03
1682001	1.122	0.97364694	null	hypothetical protein	PA2575	7	92.22
1682004	0.363	0.88366526	null	probable AMP-binding enzyme	PA2557	2	92.14
1682007	1.086	0.7746171	null	hypothetical protein	PA2567	1	93.31
1682010	1.568	1.1252563	kynA	hypothetical protein	PA2579	20	93.44
1682013	0.578	2.468472	null	conserved hypothetical protein	PA2580	27	93.72
1682016	1.350	0.97739947	null	probable transcriptional regulator	PA2577	23	92.53
1682019	1.375	0.9951852	null	probable acetyltransferase	PA2578	23	92.58
1682022	1.155	2.5529153	null	hypothetical protein	PA2581	1	93.26
1682025	1.171	0.7489172	null	hypothetical protein	PA2576	34	93.45
1682028	0.934	0.5998785	null	probable two-component response regulator	PA2572	149	96.39
1682031	0.883	0.7944156	null	hypothetical protein	PA2582	117	93.77
1682034	0.919	0.7982257	gacA	response regulator GacA	PA2586	93	93.63
1682037	1.939	0.32761908	null	probable transcriptional regulator	PA2588	36	93.14
1682040	0.859	0.6773554	pgsA	CDP-diacylglycerol--glycerol-3-phosphate	PA2584	58	92.49
1682043	1.178	0.8515625	null	probable sensor/response regulator hybrid	PA2583	35	95.28
1682046	0.546	0.3878537	pqsH	probable FAD-dependent monooxygenase	PA2587	204	92.28
1682049	0.579	0.27411526	null	probable periplasmic	PA2592	13	92.11
1682052	0.245	-0.5878871	null	probable transcriptional regulator	PA2591	23	94.11
1682055	1.009	1.5315657	null	hypothetical protein	PA2589	354	92.67
1682058	0.903	-0.006195257	null	hypothetical protein	PA2597	78	94.35
1682061	1.198	0.7487942	null	conserved hypothetical protein	PA2595	133	92.19
1682064	1.058	0.8092457	null	conserved hypothetical protein	PA2594	1	93.8
1682067	1.250	0.927673	null	hypothetical protein	PA2593	1	93.87
1682070	0.892	0.7206844	uvrC	excinuclease ABC subunit C	PA2585	11	92.84
1682073	0.661	0.1227605	null	hypothetical protein	PA2598	29	97.86
1682076	1.127	1.0608594	null	conserved hypothetical protein	PA2599	16	92.44
1682079	0.965	-1.7151515	null	hypothetical protein	PA2600	236	96.08
1682082	1.452	1.0659806	null	probable chemotaxis transducer	PA2573	257	94.17
1682085	1.042	2.0534506	null	conserved hypothetical protein	PA2596	141	92.57
1682088	0.579	0.29729474	null	hypothetical protein	PA2602	141	97.05
1682091	1.047	0.14878786	null	probable transcriptional regulator	PA2601	99	95.21
1682094	1.223	0.94128305	null	conserved hypothetical protein	PA2604	31	94.57
1682097	1.103	0.8141328	null	probable sulfate transporter	PA2563	318	96.54
1682100	0.596	0.33309585	null	conserved hypothetical protein	PA2610	26	92.47
1682103	0.791	0.59613574	null	hypothetical protein	PA2609	1	92.47
1682106	0.291	-4.479768	null	conserved hypothetical protein	PA2608	219	95.04
1682108	0.975	0.7585417	null	conserved hypothetical protein	PA2607	1	93.4
1682111	1.492	0.5497494	null	probable thiosulfate sulfurtransferase	PA2603	1	94.19
1682114	0.970	0.83896846	lolA	periplasmic chaperone LolA	PA2614	199	95.91
1682117	0.985	0.8092926	serS	seryl-tRNA synthetase	PA2612	55	94.39
1682120	0.833	0.48314816	null	conserved hypothetical protein	PA2605	92	92.43
1682121	0.726	0.56056243	null	hypothetical protein	PA2618	1	92.34
1682124	1.009	2.9415872	null	hypothetical protein	PA2590	149	93.22
1682127	1.192	1.055755	aat	leucyl/phenylalanyl-tRNA-protein transferase	PA2617	1	92.64
1682130	1.182	0.9720648	trxB1	thioredoxin reductase 1	PA2616	3	92.27
1682133	0.917	0.7875102	null	conserved hypothetical protein	PA2613	40	97.15
1682136	1.097	1.0573318	infA	initiation factor	PA2619	78	92.75
1682138	1.142	0.953339	null	conserved hypothetical protein	PA2621	125	95
1682139	0.953	0.7663333	cspD	cold-shock protein CspD	PA2622	133	92.37
1682141	1.284	1.1406993	icd	isocitrate dehydrogenase	PA2623	76	92.16
1682145	0.968	0.7079204	null	conserved hypothetical protein	PA2625	222	94.84
1682147	0.945	0.6895257	cysG	siroheme synthase	PA2611	951	93.94
1682152	0.973	0.8519087	null	conserved hypothetical protein	PA2606	86	94.34
1682153	0.659	0.4574531	null	hypothetical protein	PA2628	97	94.04
1682156	0.934	0.8029617	trmU	tRNA methyltransferase	PA2626	251	92.33
1682159	1.138	1.0347625	ftsK	cell division protein FtsK	PA2615	15	93.03
1682162	1.162	0.95404893	clpA	ATP-binding protease component ClpA	PA2620	253	96.28
1682165	0.717	0.56025755	null	conserved hypothetical protein	PA2627	389	93.49
1682168	1.051	0.76506674	null	hypothetical protein	PA2633	59	97.47
1682171	0.657	0.47743955	null	hypothetical protein	PA2632	33	95.1

1682174	0.923	0.46156698	null	probable permease of ABC transporter	PA2409	221	96.52
1682177	0.809	0.72213227	nuoA	NADH dehydrogenase I chain A	PA2637	25	92.34
1682179	1.065	0.40857142	null	probable acetyl transferase	PA2631	7	92.19
1682182	0.908	0.78280544	nuoB	NADH dehydrogenase I chain B	PA2638	21	92.04
1682185	1.067	0.5523711	null	hypothetical protein	PA2636	1	92.84
1682188	3.030	2.3228142	null	hypothetical protein	PA2415	26	92.34
1682191	1.045	0.86203194	nuoE	NADH dehydrogenase I chain E	PA2640	97	93.91
1682194	1.009	-2.9013157	null	hypothetical protein	PA2635	22	92.26
1682197	1.082	0.7963249	purB	adenylosuccinate lyase	PA2629	42	94.03
1682200	0.829	0.72007173	idh	isocitrate dehydrogenase	PA2624	28	95.1
1682203	1.149	0.8321997	null	conserved hypothetical protein	PA2630	280	94.63
1682206	1.065	0.9268524	nuoH	NADH dehydrogenase I chain H	PA2643	2	92.08
1682209	1.082	0.98495245	nuoK	NADH dehydrogenase I chain K	PA2646	246	92.33
1682212	1.167	1.0478646	nuoG	NADH dehydrogenase I chain G	PA2642	19	92.22
1682215	1.263	1.0978243	nuoJ	NADH dehydrogenase I chain J	PA2645	31	95.18
1682218	1.344	1.1680435	null	probable isocitrate lyase	PA2634	150	92.64
1682221	1.069	0.775872	null	conserved hypothetical protein	PA2650	1	92.97
1682224	0.892	0.6149577	null	conserved hypothetical protein	PA2651	249	95.73
1682227	1.165	0.17424908	null	hypothetical protein	PA2655	50	93.93
1682228	1.132	1.016082	nuoM	NADH dehydrogenase I chain M	PA2648	9	92.38
1682231	1.401	-0.09951077	null	probable chemotaxis transducer	PA2652	152	94.26
1682234	1.334	0.7045016	null	probable two-component sensor	PA2656	229	92.67
1682237	1.075	0.82653826	nuoF	NADH dehydrogenase I chain F	PA2641	655	93.35
1682240	1.073	0.88654184	null	hypothetical protein	PA2658	105	93.42
1682243	1.338	1.0763111	null	hypothetical protein	PA2659	144	95.39
1682244	1.382	1.4163641	null	hypothetical protein	PA2660	296	95.82
1682247	1.247	0.93088293	null	probable transporter	PA2653	25	93.26
1682250	1.150	0.992648	null	probable two-component response regulator	PA2657	2	93.93
1682253	1.393	0.4637645	null	probable 6-pyruvoyl tetrahydrobiopterin	PA2666	37	97.79
1682257	1.640	1.5641515	null	hypothetical protein	PA2663	87	92.59
1682258	1.771	1.4397078	fhp	flavohemoprotein	PA2664	58	93.25
1682261	1.298	0.9619625	null	hypothetical protein	PA2668	24	95.87
1682262	1.009	1.0778017	null	hypothetical protein	PA2669	1	92.49
1682265	2.094	1.8485597	null	hypothetical protein	PA2670	14	94.17
1682268	0.676	0.59733677	null	conserved hypothetical protein	PA2667	15	93.86
1682271	1.612	1.4507335	null	conserved hypothetical protein	PA2662	164	93.32
1682274	1.436	0.97113866	null	hypothetical protein	PA2671	22	95.09
1682277	1.435	0.7388439	null	probable type II secretion system protein	PA2672	154	93.73
1682280	0.558	0.12514094	null	hypothetical protein	PA2661	497	93.99
1682283	1.009	1.8462186	null	probable type II secretion system protein	PA2673	80	93.34
1682286	1.270	0.606302	null	probable type II secretion system protein	PA2674	19	94.13
1682289	1.133	-2.4278789	null	probable type II secretion system protein	PA2675	45	92.95
1682292	17.099	0.8899806	null	probable type II secretion system protein	PA2676	6	92.8
1682295	1.559	1.0903904	null	probable permease of ABC-2 transporter	PA2678	1	92.28
1682298	1.197	0.98340654	nuoL	NADH dehydrogenase I chain L	PA2647	54	94.95
1682301	1.009	-0.5159091	null	probable quinone oxidoreductase	PA2680	308	94.84
1682304	0.734	1.0025362	null	probable type II secretion protein	PA2677	13	97.59
1682307	0.550	0.43732104	null	hypothetical protein	PA2679	149	94.43
1682310	1.152	0.8880559	null	probable transcriptional regulator	PA2681	88	97.79
1682313	1.024	0.89917403	null	probable serine/threonine dehydratase,	PA2683	419	96.12
1682316	0.965	0.73900855	pfeS	two-component sensor PfeS	PA2687	56	94.47
1682319	1.009	0.4157731	pfeR	two-component response regulator PfeR	PA2686	109	92.13
1682322	1.009	0.89479697	null	conserved hypothetical protein	PA2684	4	94.21
1682325	1.016	0.4741966	null	conserved hypothetical protein	PA2682	85	92.82
1682328	1.338	1.179781	null	hypothetical protein	PA2689	155	96.84
1682331	1.224	0.9098829	null	conserved hypothetical protein	PA2691	123	96.87
1682334	1.057	0.8493036	null	probable transcriptional regulator	PA2665	2	95.85
1682337	1.234	0.7269262	null	probable transcriptional regulator	PA2692	62	94.45
1682340	0.807	0.6934968	null	probable transposase	PA2690	45	92.38
1682343	1.669	1.2964213	null	probable thioredoxin	PA2694	58	95.46
1682347	1.022	0.6020452	null	conserved hypothetical protein	PA2693	86	94.44
1682349	1.009	0.27324557	null	probable hydrolase	PA2698	63	92.47
1682352	1.570	4.023042	null	conserved hypothetical protein	PA2695	152	95.01
1682355	1.017	0.9085482	nuoD	NADH dehydrogenase I chain C,D	PA2639	57	93.3
1682358	1.161	1.5721601	null	hypothetical protein	PA2697	189	96.17
1682360	0.779	0.65960443	null	conserved hypothetical protein	PA2685	8	92.96
1682363	1.468	1.2514093	null	probable transcriptional regulator	PA2696	478	92.84
1682366	1.185	0.77306765	null	hypothetical protein	PA2703	2	92.5
1682368	1.009	-0.5140837	null	probable porin	PA2700	54	97.92
1682371	1.092	0.7842916	null	probable chemotaxis transducer	PA2654	477	95.16
1682374	1.076	0.93119717	null	hypothetical protein	PA2706	48	95.13
1682377	1.094	1.0571386	null	hypothetical protein	PA2707	500	92.96
1682380	1.009	0.6318487	null	hypothetical protein	PA2699	591	92.84
1682383	0.600	-0.60916865	cysK	cysteine synthase A	PA2709	1	93.06
1682386	1.095	0.76897556	null	hypothetical protein	PA2705	137	92.39
1682389	1.003	0.860943	null	hypothetical protein	PA2710	42	94.8
1682392	0.883	0.75548494	null	probable major facilitator superfamily (MFS)	PA2701	6	97.96
1682395	0.871	-0.8975604	null	hypothetical protein	PA2708	456	93.6
1682398	1.055	0.46938148	null	conserved hypothetical protein	PA2713	220	96.08
1682401	1.009	1.1967707	null	probable ferredoxin	PA2715	226	96.48
1682403	1.172	0.8911147	null	probable molybdopterin oxidoreductase	PA2714	4	92.17
1682406	0.164	0.7692157	cpo	chloroperoxidase precursor	PA2717	78	92.47
1682409	1.261	1.1211197	null	probable transcriptional regulator	PA2704	14	95.32
1682412	0.884	0.7838537	null	probable periplasmic	PA2711	1	94.59
1682415	1.474	1.1139401	null	hypothetical protein	PA2720	24	92.31
1682418	0.965	0.011793089	null	hypothetical protein	PA2712	574	93.04
1682421	1.115	1.0485324	null	hypothetical protein	PA2719	2	93.46
1682424	0.880	0.5411412	null	probable transcriptional regulator	PA2718	107	93.44
1682427	1.189	0.87737703	null	hypothetical protein	PA2723	144	92.23
1682429	1.223	0.8578302	null	hypothetical protein	PA2722	258	95.08
1682431	1.440	0.43602568	null	hypothetical protein	PA2721	46	94.49
1682434	1.693	-1.407212	null	probable FMN oxidoreductase	PA2716	11	92.45
1682437	1.303	1.1515131	nuoN	NADH dehydrogenase I chain N	PA2649	196	92.64
1682440	0.988	0.83303624	null	hypothetical protein	PA2730	61	92.3
1682443	0.689	0.3006906	null	probable radical activating enzyme	PA2726	58	97.43
1682446	0.875	0.73064095	null	hypothetical protein	PA2724	210	96.34
1682448	1.043	0.8581716	null	hypothetical protein	PA2731	79	95.12
1682450	1.036	0.6614831	null	conserved hypothetical protein	PA2733	7	92.94
1682453	1.173	0.9675873	null	hypothetical protein	PA2736	79	93.4
1682455	0.871	1.1802089	null	hypothetical protein	PA2728	29	97.69
1682458	0.983	0.85736513	null	hypothetical protein	PA2734	374	93.45
1682461	0.925	0.67595255	null	hypothetical protein	PA2732	7	92.59
1682464	1.106	0.7937994	null	hypothetical protein	PA2702	22	92
1682467	0.813	0.7859679	himA	integration host factor, alpha subunit	PA2738	182	94.12
1682469	0.873	0.85945225	null	conserved hypothetical protein	PA2737	5	93.91
1682472	0.967	1.0579267	rpmI	50S ribosomal protein L35	PA2742	29	92.62
1682474	1.117	0.97499466	pheS	phenylalanyl-tRNA synthetase, alpha-subunit	PA2740	16	92.48
1682477	1.009	3.125	pfeA	Ferric enterobactin receptor, outer membrane	PA2688	587	95.65
1682480	1.210	1.0262057	null	probable restriction-modification system	PA2735	78	92.47

1682483	0.972	0.79019326	null	probable chaperone	PA2725	52	93.33
1682486	0.947	1.0335703	rplT	50S ribosomal protein L20	PA2741	113	92.48
1682488	0.787	0.78879035	infC	translation initiation factor IF-3	PA2743	68	92.19
1682491	0.716	-1.6046919	null	probable methionine aminopeptidase	PA2748	77	96.54
1682494	0.981	2.869317	null	probable hydrolase	PA2745	90	93.15
1682497	1.145	0.48773164	null	hypothetical protein	PA2750	302	97.82
1682500	1.144	0.954384	thrS	threonyl-tRNA synthetase	PA2744	13	92.49
1682503	1.100	-4.68E-04	null	conserved hypothetical protein	PA2751	38	92.3
1682506	0.701	0.31822556	null	conserved hypothetical protein	PA2752	141	94.11
1682509	1.009	-4.630784	null	hypothetical protein	PA2729	5	92.07
1682512	1.387	1.1416792	null	hypothetical protein	PA2753	29	94.23
1682516	1.066	-2.7308774	null	hypothetical protein	PA2746	9	93.52
1682518	1.355	1.2098782	null	conserved hypothetical protein	PA2754	120	93.05
1682521	1.038	0.8354988	pheT	phenylalanyl-tRNA synthetase, beta subunit	PA2739	25	93.97
1682524	1.500	0.068546295	endA	DNA-specific endonuclease I	PA2749	54	94.32
1682528	1.004	0.8735612	eco	ecotin precursor	PA2755	302	96.62
1682530	0.610	0.53336424	null	hypothetical protein	PA2756	32	92.57
1682533	0.763	0.075862244	null	hypothetical protein	PA2763	9	92.73
1682536	1.163	0.8307411	null	hypothetical protein	PA2761	97	94.29
1682539	0.320	2.6983156	null	hypothetical protein	PA2747	28	93.02
1682541	1.118	0.9129275	null	probable outer membrane protein precursor	PA2760	127	92.05
1682544	1.066	1.6075646	null	hypothetical protein	PA2764	1	93.22
1682547	1.009	1.9153153	null	probable transcriptional regulator	PA2758	126	96.06
1682550	1.900	1.9715385	null	hypothetical protein	PA2762	10	92.51
1682553	18.404	16.82683	null	hypothetical protein	PA2759	237	95.22
1682555	1.689	0.9303221	null	hypothetical protein	PA2727	34	92.57
1682558	1.153	0.2975294	null	hypothetical protein	PA2765	5	92.73
1682561	0.824	0.47285375	null	hypothetical protein	PA2772	74	93.68
1682564	1.009	0.3277778	null	hypothetical protein	PA2768	327	93.1
1682567	1.226	1.0021601	null	hypothetical protein	PA2770	194	97.34
1682570	1.516	0.99660236	null	probable transcriptional regulator	PA2766	93	95.6
1682574	0.954	0.7461552	null	hypothetical protein	PA2757	274	94.82
1682576	0.634	0.48800343	null	hypothetical protein	PA2773	49	92.6
1682579	0.857	0.6847471	null	hypothetical protein	PA2769	41	97.98
1682582	1.009	-0.3702463	null	probable enoyl-CoA hydratase/isomerase	PA2767	2	92.04
1682585	0.865	0.31528044	null	hypothetical protein	PA2775	4	92.67
1682588	0.703	0.53406847	null	hypothetical protein	PA2774	2	92.78
1682591	1.047	0.7365491	null	hypothetical protein	PA2778	106	93.1
1682594	1.047	0.5213187	null	conserved hypothetical protein	PA2777	220	94.07
1682597	1.160	0.6787467	null	conserved hypothetical protein	PA2776	416	94.21
1682600	0.749	0.54351616	null	hypothetical protein	PA2781	19	92.22
1682603	0.662	-0.31133786	null	hypothetical protein	PA2780	87	92.9
1682606	0.751	0.3200292	null	hypothetical protein	PA2786	166	94.69
1682610	0.997	0.595646	null	conserved hypothetical protein	PA2785	1	93.86
1682611	0.992	-0.15580952	null	hypothetical protein	PA2782	356	97.3
1682613	0.831	0.6666381	null	hypothetical protein	PA2791	146	95.41
1682615	2.004	1.142241	null	hypothetical protein	PA2784	387	95.77
1682618	1.597	1.2949913	null	hypothetical protein	PA2790	1	96.93
1682621	1.009	1.1834457	null	hypothetical protein	PA2779	77	93.86
1682623	1.393	1.1431421	cpg2	carboxypeptidase G2 precursor	PA2787	644	96.73
1682626	1.732	1.571655	null	hypothetical protein	PA2794	172	92.39
1682629	0.960	0.4923505	null	hypothetical protein	PA2792	74	95.36
1682632	0.492	0.060288936	null	hypothetical protein	PA2793	52	92.99
1682635	0.985	0.8741156	null	hypothetical protein	PA2797	65	94.48
1682638	1.102	0.97905636	null	conserved hypothetical protein	PA2795	27	95.77
1682641	1.129	0.82475436	null	probable chemotaxis transducer	PA2788	252	97.43
1682644	0.813	0.93006885	null	hypothetical protein	PA2789	2	93.67
1682647	1.401	0.7223711	null	hypothetical protein	PA2799	3	92.5
1682650	1.161	0.8369257	null	hypothetical protein	PA2801	3	92.03
1682653	0.895	0.6326727	null	conserved hypothetical protein	PA2800	8	93.58
1682656	1.351	0.94940054	null	probable transcriptional regulator	PA2802	107	94.94
1682659	0.836	0.65295637	null	probable two-component response regulator	PA2798	357	96.39
1682662	1.020	0.7671477	null	hypothetical protein	PA2803	1	94.87
1682665	1.222	0.82639486	null	conserved hypothetical protein	PA2806	28	92.33
1682668	1.000	0.86358535	null	hypothetical protein	PA2808	1	92.99
1682670	1.061	5.4653845	null	hypothetical protein	PA2807	8	95.4
1682673	1.104	0.94853795	null	hypothetical protein	PA2805	125	95.82
1682677	1.019	0.7694056	null	probable two-component response regulator	PA2809	431	96.2
1682679	4.516	2.8014405	null	probable permease of ABC-2 transporter	PA2811	198	92.76
1682682	3.772	3.2473228	null	probable glutathione S-transferase	PA2813	3	92.35
1682685	0.695	0.48216075	tal	transaldolase	PA2796	400	94.88
1682688	1.009	0.73545235	null	hypothetical protein	PA2783	463	94.38
1682691	1.380	0.08888888	null	hypothetical protein	PA2804	160	94.55
1682694	1.483	-3.122199	null	hypothetical protein	PA2814	3	92.46
1682697	1.009	1.0583333	null	hypothetical protein	PA2819	232	92.42
1682700	13.575	0.8415127	null	probable ATP-binding component of ABC	PA2812	198	96.83
1682704	1.405	1.1699054	null	hypothetical protein	PA2816	2	92.96
1682705	1.076	0.6352886	null	probable acyl-CoA dehydrogenase	PA2815	230	95.8
1682708	2.102	1.145141	null	hypothetical protein	PA2817	104	92.26
1682711	1.054	0.7525366	null	conserved hypothetical protein	PA2822	281	96.59
1682714	1.201	0.9051921	arr	aminoglycoside response regulator	PA2818	3	92.39
1682717	0.813	0.18170401	null	probable sensor/response regulator hybrid	PA2824	49	92.77
1682720	0.870	0.56477356	null	probable transcriptional regulator	PA2825	149	93.94
1682723	1.262	1.0020163	null	conserved hypothetical protein	PA2827	37	92.24
1682726	1.247	0.86819535	null	probable glutathione peroxidase	PA2826	261	93.65
1682728	1.315	0.45027572	null	probable two-component sensor	PA2810	75	92.12
1682731	1.157	0.95445365	null	hypothetical protein	PA2820	141	94.99
1682734	1.261	0.7812622	null	conserved hypothetical protein	PA2823	458	94.83
1682737	1.329	0.9966017	tpm	thiopurine methyltransferase	PA2832	22	93.76
1682740	1.032	0.7503005	null	hypothetical protein	PA2829	131	93.41
1682744	1.399	1.0156759	null	conserved hypothetical protein	PA2833	198	97.85
1682745	1.005	0.7420145	htpX	heat shock protein HtpX	PA2830	216	95.13
1682748	1.009	1.3113053	null	conserved hypothetical protein	PA2831	6	92.02
1682751	0.493	0.58868694	null	probable transcriptional regulator	PA2834	195	97.48
1682754	2.531	0.7579351	null	probable aminotransferase	PA2828	16	92.7
1682757	1.019	-1.3827838	null	conserved hypothetical protein	PA2839	109	95.51
1682760	0.998	0.594197	null	probable transcriptional regulator	PA2489	1	97.97
1682763	1.009	0.5588466	null	probable transcriptional regulator	PA2838	182	92.16
1682766	1.009	0.11076925	null	probable aldolase	PA2843	1	96.1
1682769	1.052	1.5504658	null	probable secretion protein	PA2836	380	94.55
1682772	1.007	0.37206852	null	probable enoyl-CoA hydratase/isomerase	PA2841	503	93.51
1682775	1.498	0.45718032	null	hypothetical protein	PA2845	13	92.49
1682777	1.800	0.9915966	null	probable outer membrane protein precursor	PA2837	968	93.14
1682780	1.413	1.342119	nuoI	NADH Dehydrogenase I chain I	PA2644	14	93.1
1682783	1.118	0.8814025	null	probable glutathione S-transferase	PA2821	203	92.73
1682786	0.694	-1.4307575	ohr	organic hydroperoxide resistance protein	PA2850	166	93.9
1682789	0.955	0.25143114	null	probable transcriptional regulator	PA2849	1	94.95
1682792	0.385	-2.193919	null	probable transcriptional regulator	PA2846	109	92.42
1682795	1.192	1.4359586	oprI	Outer membrane lipoprotein OprI precursor	PA2853	45	93.13

1682797	0.994	0.91001874	null	probable major facilitator superfamily (MFS)	PA2835	110	92.27
1682800	1.004	-2.962055	null	conserved hypothetical protein	PA2847	565	97.36
1682803	0.732	0.5953869	null	hypothetical protein	PA2855	205	92.4
1682806	0.440	-0.06061563	tesA	acyl-CoA thioesterase I precursor	PA2856	62	92.84
1682809	0.839	0.7447976	null	probable transcriptional regulator	PA2848	634	93.84
1682812	0.864	0.6428477	null	hypothetical protein	PA2852	171	97.28
1682815	1.086	0.86222523	null	hypothetical protein	PA2842	98	95.86
1682818	0.939	0.8819502	null	probable ATP-binding component of ABC	PA2857	38	92.42
1682821	1.160	0.8918929	null	hypothetical protein	PA2860	87	92.04
1682824	1.213	0.7861285	null	probable ATP-dependent RNA helicase	PA2840	59	95.13
1682827	0.825	0.6834129	greB	transcription elongation factor GreB	PA2859	362	96.67
1682830	1.145	1.0179855	lipA	lactonizing lipase precursor	PA2862	30	95.12
1682833	0.984	0.8198808	null	conserved hypothetical protein	PA2864	47	92.3
1682836	0.901	0.6584075	null	probable glycosylase	PA2865	144	94.97
1682839	1.031	0.9893629	mttC	secretion protein MttC	PA2866	283	93.4
1682842	0.946	0.7557371	null	conserved hypothetical protein	PA2854	393	96.88
1682845	1.025	0.7317461	ligT	2'-5' RNA ligase	PA2861	326	94.23
1682848	1.070	0.30360252	null	hypothetical protein	PA2871	490	96.61
1682851	1.126	0.90856725	null	hypothetical protein	PA2869	290	92.07
1682854	0.704	-0.37559813	lipH	lipase modulator protein	PA2863	36	95.41
1682857	0.784	0.5845509	null	hypothetical protein	PA2872	341	97.82
1682860	0.964	0.8982974	null	probable chemotaxis transducer	PA2867	459	92.55
1682863	1.132	1.1153319	null	hypothetical protein	PA2874	208	92.78
1682866	0.832	0.58889616	null	conserved hypothetical protein	PA2875	11	93.46
1682869	0.992	1.297924	null	hypothetical protein	PA2878	42	92.61
1682872	1.626	4.084326	null	hypothetical protein	PA2868	198	96.9
1682875	1.009	1.6921717	null	hypothetical protein	PA2880	7	92.38
1682878	1.221	0.4847065	null	probable transcriptional regulator	PA2879	73	95.33
1682881	0.625	0.21851707	null	probable transcriptional regulator	PA2877	338	93.71
1682884	1.201	0.9629752	null	probable two-component response regulator	PA2881	224	92.6
1682887	0.885	0.72467554	null	hypothetical protein	PA2883	98	92.37
1682889	0.954	0.8070885	null	probable short-chain dehydrogenase	PA2887	294	96.46
1682892	0.684	1.4740744	null	conserved hypothetical protein	PA2858	536	96.59
1682895	0.920	0.62945795	null	probable transcriptional regulator	PA2885	71	95.49
1682898	0.845	0.6430501	pyrF	orotidine 5'-phosphate decarboxylase	PA2876	239	96.18
1682901	1.082	0.73135936	null	hypothetical protein	PA2886	382	95.29
1682904	0.604	-2.8697278	dsbG	thiol:disulfide interchange protein DsbG	PA2476	381	92.49
1682907	1.009	-0.2268908	null	probable acyl-CoA dehydrogenase	PA2889	54	92.67
1682910	1.009	1.3828571	null	probable two-component sensor	PA2882	279	94.26
1682913	1.487	1.6551136	null	probable enoyl-CoA hydratase/isomerase	PA2890	57	95.85
1682916	1.009	2.356994	null	probable biotin-dependent carboxylase	PA2888	932	92.41
1682919	0.886	0.7692577	null	hypothetical protein	PA2884	3	92.56
1682922	1.536	1.6399086	null	probable short-chain dehydrogenase	PA2892	472	94.86
1682925	0.943	0.14122757	null	hypothetical protein	PA2873	260	92.97
1682928	0.720	0.28449005	null	probable transcriptional regulator	PA2899	205	96.52
1682931	1.030	0.70269364	null	hypothetical protein	PA2894	154	96.44
1682933	1.028	0.85318154	null	probable transcriptional regulator	PA2897	88	94.4
1682936	23.753	0.33842823	null	hypothetical protein	PA2870	141	92.88
1682939	1.041	0.8416413	null	hypothetical protein	PA2895	282	97.97
1682942	0.975	0.86252534	null	hypothetical protein	PA2898	6	96.51
1682945	0.814	0.51878726	null	hypothetical protein	PA2901	262	93.8
1682948	1.030	0.5793975	null	probable sigma-70 factor, ECF subfamily	PA2896	310	94.51
1682951	0.830	0.71331024	null	probable outer membrane protein precursor	PA2900	218	92.2
1682954	0.767	-0.47228542	cobH	precorrin isomerase CobH	PA2905	80	93.98
1682957	0.940	0.6402824	cobI	precorrin-2 methyltransferase CobI	PA2904	650	97.71
1682960	1.196	0.20611763	cobL	precorrin-6y-dependent methyltransferase CobL	PA2907	796	96.66
1682963	0.570	2.479425	null	hypothetical protein	PA2902	321	92.43
1682966	0.903	0.3285177	cobJ	precorrin-3 methylase CobJ	PA2903	205	95.72
1682969	0.815	0.5618305	null	hypothetical protein	PA2909	193	95.86
1682972	1.194	0.8997785	null	probable ATP-binding component of ABC	PA2912	1	95.56
1682975	1.009	1.959389	cbiD	cobalamin biosynthetic protein CbiD	PA2908	568	92.43
1682979	0.982	0.7457492	null	probable permease of ABC transporter	PA2914	737	94.36
1682981	0.895	0.74086535	null	hypothetical protein	PA2916	427	93.88
1682984	1.009	1.6803215	null	hypothetical protein	PA2913	220	93.09
1682987	0.871	0.6883062	null	hypothetical protein	PA2915	3	92.54
1682990	1.009	4.201818	null	probable short-chain dehydrogenase	PA2918	2	92.32
1682994	1.004	0.946572	null	hypothetical protein	PA2919	34	92.93
1682995	0.777	0.5106826	null	probable transcriptional regulator	PA2917	81	92.02
1683000	1.139	0.89444906	null	conserved hypothetical protein	PA2910	6	92.5
1683001	1.009	1.6279719	hisJ	periplasmic histidine-binding protein HisJ	PA2923	180	96.07
1683004	0.477	-1.131345	null	probable hydrolase	PA2922	34	93.99
1683007	1.829	1.2436156	null	conserved hypothetical protein	PA2844	115	93.67
1683010	1.397	0.78410876	hisQ	histidine transport system permease HisQ	PA2924	173	92.14
1683013	1.462	1.2424877	null	hypothetical protein	PA2927	106	92.52
1683016	1.071	-0.59583336	hisM	histidine transport system permease HisM	PA2925	6	92.8
1683019	0.561	2.1363094	null	probable transcriptional regulator	PA2921	493	94.21
1683022	0.840	0.54779196	null	probable transcriptional regulator	PA2930	373	92.33
1683025	1.045	0.67737395	null	probable TonB-dependent receptor	PA2911	1	97.66
1683028	1.009	-0.3221445	null	hypothetical protein	PA2929	387	92.01
1683031	2.483	1.0264676	hisP	histidine transport protein HisP	PA2926	416	94.05
1683034	0.634	1.8432486	null	probable transcriptional regulator	PA2931	457	93.45
1683037	1.069	-0.17039183	null	probable oxidoreductase	PA2906	562	92.41
1683040	1.191	1.1860108	null	hypothetical protein	PA2935	355	93.15
1683041	1.163	1.6783335	null	probable hydrolase	PA2934	70	93.99
1683044	1.377	0.98379385	null	probable biotin carboxylase/biotin carboxyl	PA2891	148	97.91
1683047	1.137	0.62313604	null	probable chemotaxis transducer	PA2920	241	93.12
1683050	0.549	0.2631701	null	hypothetical protein	PA2936	263	92.09
1683053	1.061	0.6795979	null	probable major facilitator superfamily (MFS)	PA2933	30	94.29
1683056	0.934	0.39346406	null	hypothetical protein	PA2937	144	96.45
1683059	1.076	1.5845613	null	probable acyl-CoA thiolase	PA2940	104	93.54
1683062	0.815	0.61260736	null	hypothetical protein	PA2941	162	96.55
1683065	1.221	2.288158	null	probable aminopeptidase	PA2939	60	94.13
1683068	1.016	0.72541857	null	phospho-2-dehydro-3-deoxyheptonate aldolase	PA2943	5	92.11
1683071	0.712	0.6670513	cobN	cobalamin biosynthetic protein CobN	PA2944	160	95.79
1683074	0.686	0.6086256	null	hypothetical protein	PA2946	59	94.77
1683077	0.852	0.66411495	null	conserved hypothetical protein	PA2945	53	93.3
1683080	0.889	0.26240966	null	probable transporter	PA2938	10	92.67
1683083	1.364	0.78479725	null	probable lipase	PA2949	164	92.26
1683086	1.038	0.85203373	null	probable magnesium chelatase	PA2942	800	93.01
1683089	2.399	-1.5617143	morB	morphinone reductase	PA2932	156	94.86
1683092	1.090	1.0552326	etfB	electron transfer flavoprotein beta-subunit	PA2952	72	92.39
1683095	1.059	0.87386954	null	hypothetical protein	PA2950	122	92.26
1683098	1.249	0.987549	null	conserved hypothetical protein	PA2956	56	93.89
1683101	0.741	0.48412544	null	hypothetical protein	PA2955	240	92.45
1683104	1.741	1.1563715	null	hypothetical protein	PA2958	42	96.88
1683107	1.408	1.2890216	etfA	electron transfer flavoprotein alpha-subunit	PA2951	188	94.03
1683110	0.843	0.6254583	cobM	precorrin-3 methylase	PA2948	367	93.12
1683113	0.679	0.5459188	null	hypothetical protein	PA2947	193	94.97
1683115	0.967	0.7147329	pilZ	type 4 fimbrial biogenesis protein PilZ	PA2960	156	92.43

1683117	0.820	0.536068	null	hypothetical protein	PA2954	159	92.06
1683120	1.096	0.81885624	null	probable transcriptional regulator	PA2957	217	93.95
1683123	0.730	0.49857172	holB	DNA polymerase III, delta prime subunit	PA2961	315	95.87
1683126	0.852	0.9627636	acpP	acyl carrier protein	PA2966	94	95.42
1683127	0.722	0.56914467	tmk	thymidylate kinase	PA2962	273	92.34
1683130	0.658	0.66491085	rpmF	50S ribosomal protein L32	PA2970	46	92.22
1683131	0.729	0.71157295	plsX	fatty acid biosynthesis protein PlsX	PA2969	11	92.61
1683134	0.825	0.6519572	fabD	malonyl-CoA-[acyl-carrier-protein] transacylase	PA2968	3	92.63
1683137	0.733	0.5513452	null	conserved hypothetical protein	PA2959	454	93.41
1683140	0.870	0.8070734	fabF1	beta-ketoacyl-acyl carrier protein synthase II	PA2965	47	93.65
1683143	1.017	0.90906733	fabG	3-oxoacyl-[acyl-carrier-protein] reductase	PA2967	172	94.52
1683146	0.680	0.5594967	null	conserved hypothetical protein	PA2963	290	94.04
1683149	0.508	0.37945542	pabC	4-amino-4-deoxychorismate lyase	PA2964	579	97.29
1683152	0.849	0.79531974	null	conserved hypothetical protein	PA2971	192	92.45
1683155	0.871	0.75287455	null	probable peptidase	PA2973	403	93.52
1683158	0.898	0.7879084	null	conserved hypothetical protein	PA2980	3	92.39
1683159	0.792	0.3529152	null	conserved hypothetical protein	PA2972	354	94.92
1683162	0.919	0.8082452	kdsB	3-deoxy-manno-octulosonate cytidylyltransferase	PA2979	10	94.59
1683165	1.174	0.93686473	rluC	ribosomal large subunit pseudouridine synthase	PA2975	5	94.8
1683168	1.291	1.5642015	ptpA	phosphotyrosine protein phosphatase	PA2978	172	96.16
1683171	0.724	0.5558119	null	probable hydrolase	PA2974	102	96.44
1683174	0.792	0.64295655	murB	UDP-N-acetylpyruvoylglucosamine reductase	PA2977	226	93.24
1683177	1.209	1.0681885	efp	translation elongation factor P	PA2851	359	92.58
1683180	1.311	1.1670971	null	electron transfer flavoprotein-ubiquinone	PA2953	91	92.94
1683183	0.537	0.39623904	lpxK	tetraacyldisaccharide 4*-kinase	PA2981	609	92.46
1683186	0.781	0.634239	null	conserved hypothetical protein	PA2982	383	94.6
1683189	0.913	0.49398622	null	probable tolQ-type transport protein	PA2983	132	94.36
1683192	0.803	0.61086076	sth	soluble pyridine nucleotide transhydrogenase	PA2991	5	92.22
1683195	0.846	0.7107075	null	hypothetical protein	PA2992	169	92.18
1683197	0.833	1.3633881	null	probable phosphodiesterase	PA2990	72	94.12
1683200	1.887	0.64152455	null	hypothetical protein	PA2989	66	92.19
1683203	0.972	0.77001834	null	hypothetical protein	PA2985	1	94.18
1683206	0.783	0.58000684	null	conserved hypothetical protein	PA2993	196	92.83
1683209	1.104	0.7949437	nqrE	Na+-translocating NADH:quinone oxidoreductase	PA2995	486	93.81
1683212	0.869	0.71405804	null	probable ATP-binding component of ABC	PA2987	267	92.42
1683215	0.803	0.7252995	rne	ribonuclease E	PA2976	82	92.47
1683218	1.233	1.0111676	nqrB	Na+-translocating NADH:ubiquinone oxidoreductase	PA2998	51	93.11
1683221	1.249	0.9438038	nqrC	Na+-translocating NADH:ubiquinone oxidoreductase	PA2997	421	95.1
1683224	0.965	0.79953605	null	conserved hypothetical protein	PA2986	32	92.17
1683227	1.019	0.69442225	nqrF	Na+-translocating NADH:quinone oxidoreductase,	PA2994	85	92.84
1683230	1.089	0.88328505	nqrA	Na+-translocating NADH:ubiquinone oxidoreductase	PA2999	173	92.77
1683233	1.310	1.2162486	psrA	transcriptional regulator PsrA	PA3006	62	93.01
1683236	1.081	0.8984827	null	probable nucleoside phosphorylase	PA3004	132	95.69
1683239	1.048	0.87471455	lexA	repressor protein LexA	PA3007	1	92.44
1683242	1.490	1.1605573	nqrD	Na+-translocating NADH:uniquinone oxidoreductase	PA2996	93	93.61
1683245	0.811	0.67324084	null	hypothetical protein	PA3009	98	92.23
1683247	1.089	1.0319341	null	hypothetical protein	PA3003	1	96.35
1683250	0.934	0.6444598	nagZ	beta-N-acetyl-D-glucosaminidase	PA3005	84	92.61
1683253	1.013	0.8372744	null	hypothetical protein	PA3010	19	92.88
1683256	0.921	0.79556024	null	probable glyceraldehyde-3-phosphate	PA3001	38	92.31
1683259	1.393	0.9951857	null	hypothetical protein	PA3008	21	96.42
1683262	0.862	0.65373707	mfd	transcription-repair coupling protein Mfd	PA3002	211	92.93
1683265	0.657	0.44529456	topA	DNA topoisomerase I	PA3011	3	92
1683268	1.116	0.9679252	null	hypothetical protein	PA3015	12	92.49
1683271	0.740	0.5951426	null	hypothetical protein	PA3012	13	92.9
1683274	1.196	1.0569607	foaB	fatty-acid oxidation complex beta-subunit	PA3013	54	97.13
1683277	1.243	1.0273807	null	conserved hypothetical protein	PA3017	109	95.44
1683280	1.256	0.8773731	aroP1	aromatic amino acid transport protein AroP1	PA3000	760	92.56
1683283	0.549	0.3891922	null	hypothetical protein	PA3018	104	95.92
1683286	1.066	0.8767805	null	hypothetical protein	PA3022	3	92.23
1683290	0.651	0.4662436	null	hypothetical protein	PA3021	342	95.81
1683291	1.114	0.96905994	null	hypothetical protein	PA2984	119	96.19
1683294	1.168	1.0551554	null	hypothetical protein	PA3016	223	93.71
1683297	1.009	2.3304543	null	probable carbohydrate kinase	PA3024	11	92.61
1683300	1.305	1.1402025	null	probable soluble lytic transglycosylase	PA3020	63	94.13
1683303	1.165	0.6389706	null	conserved hypothetical protein	PA2988	193	95.54
1683306	1.099	0.75727165	null	conserved hypothetical protein	PA3023	378	93.28
1683309	1.026	0.67574877	null	probable FAD-dependent glycerol-3-phosphate	PA3025	157	95.3
1683312	0.795	0.5821599	null	hypothetical protein	PA3033	1	92.84
1683314	1.284	0.93672436	moaB2	molybdopterin biosynthetic protein B2	PA3029	143	92.38
1683317	1.031	0.8130592	mobA	molybdopterin-guanine dinucleotide biosynthesis	PA3030	130	94.76
1683320	1.145	0.9424162	null	probable transcriptional regulator	PA3027	198	96.31
1683323	1.076	1.542146	null	probable glutathione S-transferase	PA3035	83	94.86
1683326	0.852	0.47708297	snr1	cytochrome c Snr1	PA3032	1	97.28
1683329	1.048	0.83861667	null	probable transcriptional regulator	PA3034	236	92.53
1683332	1.261	0.62756264	null	hypothetical protein	PA3037	341	96.1
1683335	1.638	1.3143265	null	conserved hypothetical protein	PA3040	166	92.21
1683337	0.513	2.0830586	null	conserved hypothetical protein	PA3026	7	92.55
1683340	1.256	1.1553863	faoA	fatty-acid oxidation complex alpha-subunit	PA3014	96	92.27
1683343	0.916	0.70016485	null	hypothetical protein	PA3042	135	96.9
1683344	1.272	0.47396612	null	probable transporter	PA3039	9	92.33
1683347	0.600	0.42659378	null	probable porin	PA3038	582	95.31
1683350	1.063	1.0494251	null	hypothetical protein	PA3031	46	94.91
1683352	1.605	1.2226989	null	probable two-component response regulator	PA3045	103	97.09
1683355	1.058	0.7456933	moeA2	molybdenum cofactor biosynthesis protein A2	PA3028	68	95.21
1683358	1.246	1.0379255	null	hypothetical protein	PA3041	87	92.79
1683361	1.765	1.5473211	rmf	ribosome modulation factor	PA3049	76	92.16
1683363	0.983	0.7087456	null	probable D-alanyl-D-alanine carboxypeptidase	PA3047	5	92.58
1683366	0.912	0.7946137	null	conserved hypothetical protein	PA3046	162	93.31
1683368	0.789	-0.42590865	null	hypothetical protein	PA3051	164	96.23
1683369	1.083	0.8718114	null	probable hydrolytic enzyme	PA3053	107	92.37
1683372	1.013	0.79437715	null	conserved hypothetical protein	PA3043	32	94.22
1683375	1.272	0.3825052	null	hypothetical protein	PA3036	79	97.53
1683378	1.409	0.8591124	null	hypothetical protein	PA3057	135	92.96
1683380	1.009	0.7908852	null	probable ATP-binding component of ABC	PA3019	28	93.08
1683383	0.794	3.2981544	null	hypothetical protein	PA3052	72	94.21
1683386	0.607	0.4258555	null	hypothetical protein	PA3055	102	92.5
1683389	1.326	1.1539388	null	hypothetical protein	PA3056	63	96.13
1683392	1.040	0.84404624	pyrD	dihydroorotate dehydrogenase	PA3050	13	94.19
1683395	1.056	0.8671309	pelE	hypothetical protein	PA3060	163	94.91
1683398	0.952	0.76051563	pelD	hypothetical protein	PA3061	136	95.57
1683401	1.009	-2.657738	pelC	hypothetical protein	PA3062	313	93.26
1683403	0.604	-3.351049	null	hypothetical protein	PA3066	40	92.94
1683406	1.009	0.8702631	pelF	hypothetical protein	PA3059	168	92.79
1683409	1.117	0.767816	null	hypothetical protein	PA3065	338	93.21
1683412	1.612	0.98395693	null	probable transcriptional regulator	PA3067	87	94.26
1683415	1.009	0.8922222	pelB	hypothetical protein	PA3063	289	93.85
1683418	1.198	0.65938234	null	hypothetical protein	PA3069	92	92.12
1683421	0.930	0.2561428	pelG	hypothetical protein	PA3058	22	92.49

1683424	1.076	0.64380956	null	probable two-component sensor	PA3044	84	92.83
1683427	0.893	0.75963175	null	hypothetical protein	PA3072	1	95.66
1683429	0.725	-3.1775367	null	hypothetical protein	PA3054	202	92.94
1683432	0.965	0.94136715	gdhB	NAD-dependent glutamate dehydrogenase	PA3068	20	92.8
1683435	1.281	1.1656013	null	hypothetical protein	PA3071	56	97.11
1683438	0.981	0.70790255	null	hypothetical protein	PA3076	105	97.85
1683441	1.208	0.9741685	null	conserved hypothetical protein	PA3070	2	95
1683444	1.167	0.89231664	pelA	hypothetical protein	PA3064	1	93.87
1683447	0.977	0.74386865	null	probable two-component sensor	PA3078	478	94.91
1683450	0.613	0.48164827	null	hypothetical protein	PA3080	47	96.77
1683453	0.888	0.6657356	null	hypothetical protein	PA3084	1	93.45
1683457	0.570	4.06918	null	hypothetical protein	PA3073	362	92.05
1683459	1.292	-0.4070452	pepN	aminopeptidase N	PA3083	264	96.29
1683462	0.973	0.7893616	null	hypothetical protein	PA3085	215	92.15
1683463	0.691	0.40777016	null	probable two-component response regulator	PA3077	456	96.36
1683466	0.631	0.09303279	null	conserved hypothetical protein	PA3088	86	92.11
1683469	1.296	1.0491236	null	conserved hypothetical protein	PA3081	327	94.46
1683472	0.577	0.31225967	null	hypothetical protein	PA3079	93	94.93
1683475	1.065	0.7624531	null	hypothetical protein	PA3087	608	95.18
1683478	1.083	-2.7760317	null	hypothetical protein	PA3090	104	95.59
1683481	0.970	0.82781935	null	hypothetical protein	PA3075	1	93.58
1683484	0.784	0.5367503	null	probable transcriptional regulator	PA3094	305	92.02
1683487	1.446	0.4855877	null	hypothetical protein	PA3089	369	92.81
1683490	0.949	0.6901676	null	hypothetical protein	PA3093	415	93.52
1683493	1.019	1.0430548	null	hypothetical protein	PA2928	183	95.35
1683496	0.750	0.5382789	null	hypothetical protein	PA3091	92	92.31
1683499	0.822	0.6209979	null	hypothetical protein	PA3086	1	93.54
1683502	1.111	0.8011341	xcpY	general secretion pathway protein L	PA3096	46	96.16
1683505	0.182	-0.51826525	xcpW	general secretion pathway protein J	PA3098	69	93.54
1683508	0.942	0.33944944	xcpZ	general secretion pathway protein M	PA3095	1	94
1683511	0.886	0.35444182	xcpX	general secretion pathway protein K	PA3097	298	95.66
1683514	0.839	0.7209007	xcpU	General secretion pathway outer membrane protein	PA3100	381	92.58
1683517	1.142	0.66548884	xcpT	general secretion pathway protein G	PA3101	1	93.36
1683520	0.797	0.33720198	xcpS	general secretion pathway protein F	PA3102	280	94.87
1683523	0.813	0.59076804	null	hypothetical protein	PA3074	110	97.65
1683526	0.971	0.9106587	xcpP	secretion protein XcpP	PA3104	36	92.27
1683529	0.717	0.48999864	null	probable short-chain dehydrogenase	PA3106	101	95.83
1683532	1.287	0.9856612	purF	amidophosphoribosyltransferase	PA3108	45	95.6
1683535	0.847	0.4305055	xcpR	general secretion pathway protein E	PA3103	225	97.76
1683538	0.671	0.59425884	null	hypothetical protein	PA3109	30	92.09
1683541	0.869	-0.1440205	accD	acetyl-CoA carboxylase beta subunit	PA3112	39	92.79
1683544	0.492	0.28286347	trpF	N-(5'phosphoribosyl)anthranilate (PRA)	PA3113	2	92.16
1683547	0.988	0.7683671	null	hypothetical protein	PA3110	148	97.94
1683550	0.724	0.36059585	null	probable aspartate-semialdehyde dehydrogenase	PA3116	1	94.44
1683553	0.733	0.52170527	truA	tRNA-pseudouridine synthase I	PA3114	61	94.55
1683556	0.775	0.5704039	folC	folylpolyglutamate synthetase	PA3111	14	92.13
1683559	0.978	0.84176904	metZ	o-succinylhomoserine sulfhydrylase	PA3107	186	93.88
1683562	1.067	0.53213924	leuB	3-isopropylmalate dehydrogenase	PA3118	29	93.46
1683565	1.186	0.86799735	asd	aspartate semialdehyde dehydrogenase	PA3117	1	92.58
1683568	1.165	0.5066074	null	conserved hypothetical protein	PA3119	252	95.87
1683571	0.891	0.77007854	xcpQ	general secretion pathway protein D	PA3105	1	95.19
1683574	1.209	0.77910197	leuD	3-isopropylmalate dehydratase small subunit	PA3120	107	93.95
1683577	1.516	1.3123586	null	conserved hypothetical protein	PA3123	110	94.23
1683580	1.266	0.9315713	leuC	3-isopropylmalate dehydratase large subunit	PA3121	459	93.56
1683583	0.857	0.7168085	fimV	Motility protein FimV	PA3115	26	97.54
1683586	1.173	0.5543586	null	hypothetical protein	PA3125	1	93.51
1683589	1.004	1.4153054	null	probable transcriptional regulator	PA3124	100	95.88
1683592	1.102	0.8652713	ibpA	heat-shock protein IbpA	PA3126	21	92.35
1683595	0.966	0.70436746	null	hypothetical protein	PA3127	1	94.93
1683598	0.724	0.19662885	null	conserved hypothetical protein	PA3129	12	93.49
1683601	0.671	0.5521543	null	probable aldolase	PA3131	202	95.7
1683604	0.923	1.1792314	null	probable hydrolase	PA3132	66	94.71
1683607	0.852	0.61422837	null	hypothetical protein	PA3130	5	92.37
1683610	0.974	0.52917457	null	probable transcriptional regulator	PA3135	184	92.81
1683613	0.862	0.8383117	null	probable short-chain dehydrogenase	PA3128	483	95.18
1683616	1.206	1.0122659	gltX	glutamyl-tRNA synthetase	PA3134	42	92.63
1683619	1.070	0.9252666	gbt	glycine betaine transmethylase	PA3082	288	93.82
1683622	0.675	0.075574234	null	probable secretion protein	PA3136	1	94.58
1683625	1.060	0.7251814	null	probable transcriptional regulator	PA3133	1	93.99
1683628	0.822	-2.4714372	null	probable major facilitator superfamily (MFS)	PA3137	39	92.03
1683631	0.678	0.06608357	null	probable transcriptional regulator	PA3122	386	92.57
1683634	0.832	0.7235467	null	probable amino acid aminotransferase	PA3139	13	92.97
1683637	0.727	0.6132663	wbpL	glycosyltransferase WbpL	PA3145	96	92.69
1683640	0.920	0.7845611	null	hypothetical protein	PA3142	236	92.68
1683643	0.982	0.48999104	fadH1	2,4-dienoyl-CoA reductase FadH1	PA3092	1085	94.9
1683646	2.873	-0.07179447	null	hypothetical protein	PA3140	205	94.39
1683648	0.989	0.8553771	wbpK	probable NAD-dependent epimerase/dehydratase	PA3146	2	92.62
1683653	0.715	0.57026213	null	hypothetical protein	PA3143	46	92.22
1683654	0.986	3.8785992	null	conserved hypothetical protein	PA2771	273	93.84
1683657	0.667	0.5736518	hisH2	glutamine amidotransferase	PA3152	232	93.09
1683658	0.782	0.76890105	wbpG	LPS biosynthesis protein WbpG	PA3150	100	92.05
1683661	1.048	0.9409912	wbpJ	probable glycosyl transferase WbpJ	PA3147	2	93.29
1683664	1.047	0.998995	wbpI	probable UDP-N-acetylglucosamine 2-epimerase	PA3148	280	92.22
1683667	1.009	1.4420451	uvrB	excinuclease ABC subunit B	PA3138	1	93.54
1683670	0.979	0.5955107	null	conserved hypothetical protein	PA3048	295	96.49
1683673	0.821	0.7133633	hisF2	imidazoleglycerol-phosphate synthase, cyclase	PA3151	1	92.91
1683676	0.759	0.6279326	wzy	B-band O-antigen polymerase	PA3154	288	93.18
1683679	0.921	0.7972928	wbpH	probable glycosyltransferase WbpH	PA3149	982	92.38
1683680	0.805	0.743799	wbpD	probable acetyltransferase WbpD	PA3156	91	94.09
1683683	1.043	0.917518	wbpE	probable aminotransferase WbpE	PA3155	266	92.55
1683686	0.900	0.79001963	himD	integration host factor beta subunit	PA3161	118	93.35
1683688	0.858	0.7238447	cmk	cytidylate kinase	PA3163	85	93.27
1683691	0.987	0.87347317	wbpM	nucleotide sugar epimerase/dehydratase WbpM	PA3141	63	92.35
1683694	0.553	0.4856225	null	probable acetyltransferase	PA3157	45	93.3
1683697	0.882	0.8617135	wbpA	probable UDP-glucose/GDP-mannose dehydrogenase	PA3159	271	92.72
1683700	0.833	0.85612595	rpsA	30S ribosomal protein S1	PA3162	116	93.1
1683703	0.807	0.78702384	wbpB	probable oxidoreductase WpbB	PA3158	21	94.95
1683706	1.019	0.74712384	pheA	chorismate mutase	PA3166	37	92.45
1683709	0.937	0.8603159	wzz	O-antigen chain length regulator	PA3160	162	92.18
1683712	0.822	0.5964562	serC	3-phosphoserine aminotransferase	PA3167	51	96.08
1683715	1.263	0.9514679	null	probable hydrolase	PA3172	43	95.98
1683718	1.020	0.5966724	null	conserved hypothetical protein	PA3170	396	92.18
1683721	1.017	0.8823182	ubiG	3-demethylubiquinone-9 3-methyltransferase	PA3171	423	92.36
1683724	1.002	0.15189593	null	probable transcriptional regulator	PA3174	47	96.93
1683727	1.056	0.8394796	null	probable initiation factor 2 subunit	PA3169	378	93.67
1683730	0.729	0.32575122	null	probable arginase family protein	PA3175	483	97.67
1683733	0.893	0.744887	hisC2	histidinol-phosphate aminotransferase	PA3165	309	95.67
1683736	0.838	0.68192446	null	probable short-chain dehydrogenase	PA3173	1	94.93
1683739	0.175	1.9855556	null	hypothetical protein	PA3178	2	92.33

1683742	0.646	0.5533186	wzx	O-antigen translocase	PA3153	503	92.41
1683745	2.562	2.9283333	null	hypothetical protein	PA3180	353	95.8
1683748	1.718	1.1861966	gltS	sodium/glutamate symporter GltS	PA3176	173	95.15
1683751	0.851	0.76769894	null	2-keto-3-deoxy-6-phosphogluconate aldolase	PA3181	268	96.81
1683754	0.912	0.52557737	null	hypothetical protein	PA3185	9	92.93
1683757	0.781	0.6087257	null	still frameshift 3-PHOSPHOSHIKIMATE	PA3164	101	92.91
1683760	0.410	1.3529959	null	conserved hypothetical protein	PA3179	207	93.19
1683763	0.880	0.72311145	gyrA	DNA gyrase subunit A	PA3168	119	94.65
1683766	0.919	0.76486415	zwf	glucose-6-phosphate 1-dehydrogenase	PA3183	2	93.02
1683769	0.730	0.48673677	null	probable permease of ABC sugar transporter	PA3189	1	94.22
1683772	1.006	0.74829197	oprB	Glucose/carbohydrate outer membrane porin OprB	PA3186	157	92.37
1683775	0.654	0.43356127	null	probable permease of ABC sugar transporter	PA3188	285	94.88
1683778	0.912	0.6780079	null	hypothetical protein	PA3177	245	94.66
1683781	1.079	0.95540714	null	probable binding protein component of ABC sugar	PA3190	6	92.55
1683784	1.130	0.96055955	gapA	glyceraldehyde 3-phosphate dehydrogenase	PA3195	31	93.86
1683787	0.638	0.62100774	pgl	6-phosphogluconolactonase	PA3182	365	95.07
1683790	1.018	0.82460535	edd	phosphogluconate dehydratase	PA3194	1	96.79
1683793	0.878	0.60597485	null	conserved hypothetical protein	PA3198	101	92.44
1683796	0.597	0.4925391	gltR	two-component response regulator GltR	PA3192	39	92.55
1683799	0.791	0.5123757	null	probable ATP-binding component of ABC	PA3187	46	93.07
1683802	0.919	0.8058788	null	conserved hypothetical protein	PA3202	1	93
1683804	3.104	1.338927	null	conserved hypothetical protein	PA3200	149	92.81
1683807	0.742	0.6462199	null	probable two-component sensor	PA3191	160	95.14
1683810	0.664	0.48574096	glk	glucokinase	PA3193	870	94.69
1683813	1.079	0.913764	null	conserved hypothetical protein	PA3199	83	93.03
1683816	1.002	0.68898726	null	conserved hypothetical protein	PA3201	157	93.03
1683819	0.947	0.6742196	null	probable transcriptional regulator	PA3184	52	95.14
1683822	0.854	0.60818684	null	hypothetical protein	PA3197	76	96.35
1683825	0.848	0.69467056	null	hypothetical protein	PA3203	160	92.52
1683828	1.009	1.7424027	null	conserved hypothetical protein	PA3209	119	92.92
1683830	1.076	0.7562238	null	hypothetical protein	PA3205	132	95.16
1683833	0.846	0.58860207	null	hypothetical protein	PA3196	125	92.82
1683836	0.977	0.6990762	null	hypothetical protein	PA3207	1	92.49
1683838	1.416	1.020297	null	hypothetical protein	PA3214	89	94.35
1683841	0.788	0.53668106	null	hypothetical protein	PA3213	484	95.28
1683844	1.009	0.40077695	null	hypothetical protein	PA3218	106	94.98
1683847	0.875	0.70180464	null	probable two-component response regulator	PA3204	439	92.51
1683850	0.769	0.5524105	null	probable permease of ABC transporter	PA3211	44	95.77
1683853	0.950	0.59326553	cyaB	CyaB	PA3217	26	93.93
1683856	1.716	0.5141114	null	conserved hypothetical protein	PA3208	281	97.32
1683859	1.009	1.1247205	null	hypothetical protein	PA3219	31	94.56
1683862	0.901	0.4380764	csaA	CsaA protein	PA3221	187	93.67
1683864	1.094	1.0075397	null	hypothetical protein	PA3224	30	94.99
1683866	0.707	0.617654	null	probable ATP-binding component of ABC	PA3212	278	94.48
1683869	1.093	0.8853634	null	probable transcriptional regulator	PA3215	223	95.84
1683872	1.562	-0.3041956	null	hypothetical protein	PA3216	227	96
1683873	1.179	0.69162565	null	probable transcriptional regulator	PA3220	2	92.24
1683876	0.822	-3.2039843	null	hypothetical protein	PA3222	220	92.73
1683879	4.242	0.819219	null	probable two-component sensor	PA3206	205	92.2
1683882	1.501	1.1247345	acpD	acyl carrier protein phosphodiesterase	PA3223	60	92.25
1683885	1.413	1.021257	null	hypothetical protein	PA3231	51	96.27
1683887	80.684	98.306564	null	hypothetical protein	PA3229	137	92.93
1683888	0.965	0.6868047	null	probable hydrolase	PA3226	1	93.71
1683891	1.398	0.94309187	null	conserved hypothetical protein	PA3230	1	94.88
1683894	1.437	0.9876984	null	probable glycine betaine-binding protein	PA3236	7	93.76
1683897	1.009	5.080682	null	hypothetical protein	PA3237	137	92.41
1683898	1.404	1.218811	ppiA	peptidyl-prolyl cis-trans isomerase A	PA3227	139	92.18
1683901	0.797	0.56423914	null	probable transcriptional regulator	PA3225	78	93.31
1683904	0.169	0.08992321	null	conserved hypothetical protein	PA3235	35	93.92
1683907	0.994	0.7202546	null	hypothetical protein	PA3238	81	94.34
1683910	1.023	0.78110075	null	probable nuclease	PA3232	333	92.64
1683913	1.020	0.779462	null	conserved hypothetical protein	PA3239	1	93.75
1683916	0.259	0.19494918	null	probable sodium:solute symporter	PA3234	90	93.51
1683919	1.077	0.9320078	null	probable lauroyl acyltransferase	PA3242	10	94.79
1683924	0.810	0.23379374	xcpV	general secretion pathway protein I	PA3099	234	97.73
1683925	0.853	0.7371393	minE	cell division topological specificity factor	PA3245	101	92.28
1683926	0.865	0.62925804	minC	cell division inhibitor MinC	PA3243	5	92.01
1683929	0.935	0.7105254	null	conserved hypothetical protein	PA3240	1	93.28
1683932	1.084	0.9305023	null	hypothetical protein	PA3241	115	94.66
1683935	0.984	0.7022245	rluA	pseudouridine synthase RluA	PA3246	1	92.47
1683938	1.272	1.0207672	null	hypothetical protein	PA3251	30	96.74
1683941	1.009	1.447381	null	hypothetical protein	PA3250	190	92.4
1683944	0.742	0.6186669	minD	cell division inhibitor MinD	PA3244	531	96.24
1683947	1.249	0.45858726	null	probable transcriptional regulator	PA3249	429	93.16
1683950	1.009	0.20891681	null	probable permease of ABC transporter	PA3253	663	93.2
1683953	1.173	0.8935421	null	hypothetical protein	PA3255	242	95.64
1683956	1.025	0.93904763	null	probable permease of ABC transporter	PA3252	288	92.54
1683959	1.063	0.830619	null	probable oxidoreductase	PA3256	11	92.26
1683962	0.711	0.47614875	null	hypothetical protein	PA3258	38	92.44
1683965	1.131	0.8678347	null	hypothetical protein	PA3247	145	93.92
1683968	0.912	0.7507293	null	hypothetical protein	PA3259	93	96.57
1683971	1.416	1.3855318	null	probable transcriptional regulator	PA3260	27	96.17
1683974	1.050	0.9449113	null	probable peptidyl-prolyl cis-trans isomerase,	PA3262	9	93.06
1683977	1.116	0.845788	null	hypothetical protein	PA3261	1	92.69
1683980	0.795	0.47554764	null	probable transporter	PA3265	28	92.57
1683982	0.711	0.6674042	capB	cold acclimation protein B	PA3266	127	92.87
1683984	1.046	0.7582295	null	probable ATP-binding/permease fusion ABC	PA3228	184	96.78
1683987	0.944	0.87663364	null	probable transporter	PA3264	28	93.79
1683990	0.444	0.31128633	null	probable transcriptional regulator	PA3269	1	95.51
1683993	1.078	-1.8030907	null	probable ATP-binding component of ABC	PA3254	32	92.02
1683996	1.030	0.9758768	null	hypothetical protein	PA3273	32	92.78
1683999	1.223	1.0682163	null	conserved hypothetical protein	PA3263	2	92.28
1684002	0.681	0.46384507	null	hypothetical protein	PA3270	7	96.1
1684005	1.297	1.130295	null	probable ATP-dependent DNA helicase	PA3272	296	93.14
1684008	1.009	3.6396823	null	hypothetical protein	PA3274	72	94.12
1684010	0.947	0.7819546	null	hypothetical protein	PA3233	18	96.73
1684013	1.000	0.9697663	null	probable two-component sensor	PA3271	89	97.66
1684016	0.863	0.7496117	prc	periplasmic tail-specific protease	PA3257	174	93.2
1684019	1.083	1.0348895	null	hypothetical protein	PA3278	102	92.55
1684022	0.864	0.15668462	null	hypothetical protein	PA3267	191	92.25
1684025	0.915	0.7584531	null	conserved hypothetical protein	PA3275	194	92.98
1684028	1.159	0.83046544	null	hypothetical protein	PA3276	170	92.06
1684031	0.711	0.455873	null	probable short-chain dehydrogenase	PA3277	276	93.55
1684034	0.567	-0.10313134	null	probable TonB-dependent receptor	PA3268	170	95.74
1684037	1.176	1.0181116	null	hypothetical protein	PA3284	132	95.08
1684040	0.822	-0.08403678	null	hypothetical protein	PA3282	210	93.87
1684043	1.211	0.9826298	null	hypothetical protein	PA3281	277	92.97
1684046	0.376	-0.20896225	oprO	Pyrophosphate-specific outer membrane porin OprO	PA3280	301	92.84
1684049	0.855	0.8575964	null	conserved hypothetical protein	PA3283	167	93.43

1684052	33.983	12.240916	null	hypothetical protein	PA3291	67	94.56
1684053	0.950	0.6484982	null	hypothetical protein	PA3286	161	95.89
1684056	1.009	-0.15666667	null	hypothetical protein	PA3289	269	93.41
1684059	0.484	-4.2818537	null	hypothetical protein	PA3292	108	92.32
1684062	1.009	1.0903021	null	hypothetical protein	PA3293	54	92.69
1684065	1.009	1.1745098	oprP	Phosphate-specific outer membrane porin OprP	PA3279	3	92.16
1684068	0.793	0.4603768	null	probable sigma-70 factor, ECF subfamily	PA3285	166	97.03
1684071	0.648	0.41913596	null	conserved hypothetical protein	PA3287	30	92.32
1684074	1.128	0.8924936	null	probable HIT family protein	PA3295	89	93.95
1684077	0.354	-0.071671896	null	hypothetical protein	PA3298	5	97.09
1684080	0.905	0.7321464	null	hypothetical protein	PA3288	437	94.22
1684083	1.131	0.9149588	null	probable ATP-dependent helicase	PA3297	1	93.02
1684086	1.215	1.033398	null	hypothetical protein	PA3290	671	92.18
1684089	0.922	0.8828527	phoA	alkaline phosphatase	PA3296	6	93.97
1684092	1.054	0.8349606	null	hypothetical protein	PA3301	392	92.77
1684095	0.965	-0.6509504	null	conserved hypothetical protein	PA3304	268	93.87
1684098	1.244	0.925007	null	hypothetical protein	PA3307	134	94.48
1684102	0.042	-0.3326087	null	hypothetical protein	PA3294	1767	92.14
1684103	0.882	0.70575535	null	hypothetical protein	PA3306	104	93.07
1684106	0.841	0.56995976	fadD2	long-chain-fatty-acid--CoA ligase	PA3300	140	95
1684109	1.085	0.86696655	null	conserved hypothetical protein	PA3302	1	92.57
1684112	1.000	0.5854393	null	conserved hypothetical protein	PA3310	122	94.2
1684115	1.197	1.1802949	null	conserved hypothetical protein	PA3309	126	94.46
1684118	1.177	1.0216982	hepA	RNA helicase HepA	PA3308	177	92.42
1684121	0.865	0.58304244	null	probable 3-hydroxyisobutyrate dehydrogenase	PA3312	249	95.99
1684124	1.081	0.6830394	null	hypothetical protein	PA3317	132	96.31
1684127	0.852	-0.6583099	null	probable permease of ABC transporter	PA3316	569	92.78
1684131	1.009	0.649192	null	probable ATP-binding component of ABC	PA3314	399	92.94
1684133	0.900	0.87189263	fadD1	long-chain-fatty-acid--CoA ligase	PA3299	93	93.58
1684136	0.887	0.770613	null	hypothetical protein	PA3318	137	96.96
1684139	1.092	0.7521943	null	probable permease of ABC transporter	PA3315	646	94.86
1684143	2.339	0.6711081	null	hypothetical protein	PA3320	102	97.92
1684145	0.899	0.44533578	plcN	non-hemolytic phospholipase C precursor	PA3319	288	94.43
1684148	1.601	1.1	null	conserved hypothetical protein	PA3311	43	96.31
1684151	1.115	0.8744861	null	probable major facilitator superfamily (MFS)	PA3303	429	94.85
1684154	0.593	0.17209692	null	hypothetical protein	PA3322	211	95.86
1684157	1.258	0.7389148	null	probable transcriptional regulator	PA3321	724	92.28
1684160	1.065	-2.5625	null	conserved hypothetical protein	PA3325	61	96.12
1684163	0.289	-0.23009253	null	probable Clp-family ATP-dependent protease	PA3326	60	92.16
1684166	1.811	-0.29055554	null	probable short-chain dehydrogenase	PA3324	105	93.9
1684169	1.000	0.8783515	null	conserved hypothetical protein	PA3323	1	93.38
1684172	1.009	0.24166667	null	probable short chain dehydrogenase	PA3330	186	92.83
1684175	1.009	-3.0933335	null	conserved hypothetical protein	PA3332	54	95.27
1684178	1.116	0.8092495	null	probable acyl carrier protein	PA3334	3	92.64
1684181	2.290	2.0308785	null	hypothetical protein	PA3335	15	92.12
1684184	0.638	0.12240076	null	cytochrome P450	PA3331	247	93.55
1684187	1.122	0.87778026	null	hypothetical protein	PA3305	1	95.22
1684190	1.222	1.0669715	rfaD	ADP-L-glycero-D-mannoheptose 6-epimerase	PA3337	148	92.32
1684193	0.998	1.3825257	null	hypothetical protein	PA3338	4	92.29
1684196	0.899	2.6772227	null	probable FAD-dependent monooxygenase	PA3328	498	97.55
1684199	1.392	0.7890476	fabH2	3-oxoacyl-[acyl-carrier-protein] synthase III	PA3333	74	97.43
1684202	1.061	0.50424904	null	hypothetical protein	PA3329	253	93.68
1684205	1.314	0.4435149	null	probable major facilitator superfamily (MFS)	PA3336	940	93.45
1684208	0.871	0.8633747	null	hypothetical protein	PA3313	1	93.82
1684211	1.200	0.9641015	null	hypothetical protein	PA3342	31	92.65
1684214	1.037	0.86041296	null	probable transcriptional regulator	PA3341	18	92.11
1684217	0.763	0.62422276	null	hypothetical protein	PA3345	6	93.58
1684220	1.050	0.9151853	null	hypothetical protein	PA3340	2	93.41
1684223	0.753	0.5495766	null	probable two-component response regulator	PA3346	14	93.07
1684226	1.296	2.30423	null	hypothetical protein	PA3350	126	92.43
1684229	0.851	0.6692168	null	hypothetical protein	PA3347	55	96.09
1684232	1.032	1.0657209	null	hypothetical protein	PA3351	163	92.81
1684235	1.027	0.92996854	null	hypothetical protein	PA3343	70	92.26
1684238	0.840	0.6897343	null	hypothetical protein	PA3353	10	93.22
1684241	0.825	0.6814899	null	probable chemotaxis protein methyltransferase	PA3348	51	92.32
1684244	0.868	0.83389986	null	hypothetical protein	PA3352	109	93.43
1684247	1.301	0.8393257	null	hypothetical protein	PA3354	441	95.44
1684250	0.892	0.81117487	null	probable chemotaxis protein	PA3349	22	92.23
1684253	0.515	0.4189218	null	probable non-ribosomal peptide synthetase	PA3327	3	92.43
1684256	1.009	0.5701754	null	hypothetical protein	PA3359	43	93.07
1684259	0.976	0.8352955	null	conserved hypothetical protein	PA3356	131	96.89
1684262	0.974	0.4471429	lecB	fucose-binding lectin PA-IIL	PA3361	8	93.89
1684265	1.260	1.0481539	null	hypothetical protein	PA3358	591	96.65
1684268	1.451	1.6774781	null	hypothetical protein	PA3362	64	93.38
1684271	1.973	1.7936153	null	probable chaperone	PA3365	14	94.92
1684274	1.015	0.9536912	null	hypothetical protein	PA3367	7	93.2
1684277	1.545	1.2371376	amiE	aliphatic amidase	PA3366	2	92.54
1684280	1.152	0.8827961	amiC	aliphatic amidase expression-regulating protein	PA3364	297	92.32
1684283	11.629	2.4936695	amiR	aliphatic amidase regulator	PA3363	33	94.2
1684286	1.143	0.54648495	null	probable acetyltransferase	PA3368	55	92.61
1684289	1.355	-0.71160686	null	hypothetical protein	PA3369	37	92.19
1684292	1.075	-5.583611	null	hypothetical protein	PA3370	54	92.03
1684293	1.209	0.74752474	null	hypothetical protein	PA3371	140	94.2
1684294	0.959	0.8854345	null	conserved hypothetical protein	PA3372	158	95.86
1684297	0.898	0.6735876	null	conserved hypothetical protein	PA3373	216	92.11
1684300	1.361	-0.23895068	dsdA	D-serine dehydratase	PA3357	630	94.1
1684303	1.009	1.4780124	null	probable ATP-binding component of ABC	PA3376	7	95.56
1684306	1.147	1.1500571	null	hypothetical protein	PA3339	1158	92.34
1684309	1.009	0.55665195	null	conserved hypothetical protein	PA3377	44	92.4
1684312	0.849	0.69338566	recQ	ATP-dependent DNA helicase RecQ	PA3344	415	94.13
1684315	1.009	1.1862129	null	conserved hypothetical protein	PA3374	1	92.6
1684318	1.240	1.0374043	null	conserved hypothetical protein	PA3379	568	96.88
1684321	0.966	0.7284788	null	hypothetical protein	PA3355	564	97.72
1684324	1.941	0.7747303	null	conserved hypothetical protein	PA3380	1	95.21
1684326	1.177	0.9085096	trkH	potassium uptake protein TrkH	PA3210	35	92.64
1684329	1.100	0.41688314	null	probable transcriptional regulator	PA3381	400	92.1
1684332	1.275	1.2432295	amrZ	alginate and motility regulator Z	PA3385	32	92.17
1684335	1.318	0.76129407	null	conserved hypothetical protein	PA3378	341	93.45
1684338	1.009	1.5704763	null	binding protein component of ABC phosphonate	PA3383	132	92.37
1684341	1.009	1.493634	phnC	ATP-binding component of ABC phosphonate	PA3384	6	92.73
1684344	2.586	-8.34473	null	probable ATP-binding component of ABC	PA3375	60	92.47
1684347	0.992	0.772536	null	hypothetical protein	PA3390	124	92.28
1684350	0.848	0.7315407	null	conserved hypothetical protein	PA3386	225	93.29
1684353	1.389	0.8059371	phnE	phosphonate transport protein PhnE	PA3382	283	92.67
1684356	0.506	-1.2454873	null	conserved hypothetical protein	PA3388	23	92.39
1684359	1.096	-3.1631868	null	probable secretion protein	PA3360	561	95.3
1684362	1.009	0.7210567	null	probable ring-cleaving dioxygenase	PA3389	63	92.7
1684365	1.055	1.0590122	nosZ	nitrous-oxide reductase precursor	PA3392	183	93.94
1684368	0.721	0.6289377	fpr	ferredoxin--NADP+ reductase	PA3397	125	95.49

1684371	0.821	0.59210014	null	hypothetical protein	PA3399	6	94.78
1684376	0.897	0.44298244	nosL	NosL protein	PA3396	225	95.6
1684377	1.196	0.9304756	null	hypothetical protein	PA3400	54	94.63
1684380	1.087	0.7943115	null	hypothetical protein	PA3401	225	94.38
1684383	0.931	0.78242373	nosF	NosF protein	PA3394	677	97.69
1684386	0.999	0.8670887	nosY	NosY protein	PA3395	628	93.98
1684389	0.894	0.16802278	null	hypothetical protein	PA3403	134	94.37
1684391	0.506	2.2637877	null	hypothetical protein	PA3402	190	94.48
1684394	0.935	0.81055105	null	probable outer membrane protein precursor	PA3404	1	95.07
1684397	3.502	0.32964912	hasAp	heme acquisition protein HasAp	PA3407	473	92.9
1684400	1.009	1.1278789	hasE	metalloprotease secretion protein	PA3405	757	97.81
1684403	1.247	0.96356	null	hypothetical protein	PA3411	9	94.33
1684404	0.960	1.8945633	null	probable sigma-70 factor, ECF subfamily	PA3410	9	92.31
1684407	1.241	0.8619127	null	conserved hypothetical protein	PA3413	26	92.27
1684410	1.009	-0.5163691	null	hypothetical protein	PA3412	63	94.44
1684413	1.088	0.9516875	nosD	NosD protein	PA3393	431	95.77
1684416	1.473	3.4644184	null	hypothetical protein	PA3414	198	93.91
1684419	1.000	1.0362873	null	probable transmembrane sensor	PA3409	146	96.01
1684422	1.009	1.0410255	null	probable pyruvate dehydrogenase E1 component,	PA3416	270	92.28
1684425	0.803	0.7031013	nosR	regulatory protein NosR	PA3391	202	95.26
1684428	1.009	5.1096296	null	probable dihydrolipoamide acetyltransferase	PA3415	24	93.57
1684431	0.907	0.5246396	null	probable pyruvate dehydrogenase E1 component,	PA3417	1	92.3
1684434	1.483	-0.039219856	ldh	leucine dehydrogenase	PA3418	55	94.87
1684437	1.232	0.91412604	null	hypothetical protein	PA3419	1	94.64
1684440	1.009	1.1374872	null	probable transcriptional regulator	PA3420	259	97.14
1684443	0.416	-0.32580298	hasR	Haem uptake outer membrane receptor HasR	PA3408	69	97.22
1684446	0.793	0.042685192	null	hypothetical protein	PA3425	267	92.31
1684449	1.310	1.1684113	null	hypothetical protein	PA3424	1	92.87
1684452	1.047	1.2125399	null	hypothetical protein	PA3428	220	93.86
1684455	0.744	0.5023325	null	probable enoyl CoA-hydratase/isomerase	PA3426	354	93.62
1684458	1.009	-3.842036	null	hypothetical protein	PA3422	71	92.56
1684461	2.715	1.0103365	null	probable epoxide hydrolase	PA3429	50	92.88
1684464	1.323	1.0514138	null	probable short-chain dehydrogenases	PA3427	421	95.41
1684467	1.009	0.60730237	null	probable transcriptional regulator	PA3398	453	92.34
1684470	1.790	1.5884247	null	conserved hypothetical protein	PA3431	61	96.76
1684473	0.915	0.6322384	null	probable transcriptional regulator	PA3423	148	95.69
1684476	1.529	1.3281257	null	probable aldolase	PA3430	1	96.23
1684479	1.009	1.1960088	null	probable transcriptional regulator	PA3433	62	96.48
1684482	1.340	1.1673911	null	hypothetical protein	PA3432	276	94.79
1684484	0.893	0.79492396	null	hypothetical protein	PA3436	450	92.44
1684487	0.896	0.804403	null	conserved hypothetical protein	PA3440	116	92.95
1684490	1.009	2.372602	null	conserved hypothetical protein	PA3421	198	92.01
1684493	1.052	0.42035717	null	probable molybdopterin-binding protein	PA3441	97	92.62
1684494	0.891	0.76543075	folX	d-erythro-7,8-dihydroneopterin triphosphate	PA3439	225	92.13
1684497	0.884	0.7550974	null	probable transposase	PA3434	45	92.38
1684500	0.987	0.8115846	null	conserved hypothetical protein	PA3435	266	96.48
1684503	0.695	0.5335286	null	probable short-chain dehydrogenase	PA3437	198	96.2
1684506	0.235	0.17688046	null	conserved hypothetical protein	PA3445	168	94.08
1684509	1.009	0.2697198	null	probable ATP-binding component of ABC	PA3442	257	97.8
1684512	0.946	0.6248965	null	conserved hypothetical protein	PA3444	185	94.35
1684515	1.118	1.2219031	null	probable antioxidant protein	PA3450	87	97.73
1684518	0.706	0.4934908	null	hypothetical protein	PA3451	1	95.26
1684519	0.844	0.6067101	folE1	GTP cyclohydrolase I precursor	PA3438	5	92.61
1684522	0.791	0.48181504	null	conserved hypothetical protein	PA3449	405	96.19
1684525	1.244	0.5351914	hasD	transport protein HasD	PA3406	832	94.9
1684528	0.899	0.3644231	null	probable permease of ABC transporter	PA3443	258	94.54
1684531	1.639	1.2646554	null	probable permease of ABC transporter	PA3448	465	96.73
1684534	1.009	1.5757387	null	probable ATP-binding component of ABC	PA3447	12	93.38
1684537	0.907	0.9198774	null	conserved hypothetical protein	PA3453	33	96.06
1684540	0.258	1.2533509	null	conserved hypothetical protein	PA3455	11	92.36
1684543	0.258	-0.49727613	null	conserved hypothetical protein	PA3446	1	94.45
1684546	0.720	0.41257986	mqoA	malate:quinone oxidoreductase	PA3452	223	92.46
1684549	1.260	0.9687155	null	conserved hypothetical protein	PA3461	1	93.4
1684552	0.387	1.7025642	null	probable acetyltransferase	PA3460	102	96.13
1684555	0.887	0.62144697	null	hypothetical protein	PA3457	3	92.41
1684559	1.641	1.453694	null	conserved hypothetical protein	PA3463	12	92.4
1684560	1.320	0.5402564	null	hypothetical protein	PA3464	49	94.8
1684563	1.637	1.2838523	null	probable transcriptional regulator	PA3458	72	94.61
1684566	1.009	0.81512797	null	probable very-long-chain acyl-CoA synthetase	PA2893	2	93.05
1684569	0.979	0.78462917	null	conserved hypothetical protein	PA3465	1	94.15
1684572	0.138	-1.4225423	null	probable acyl-CoA thiolase	PA3454	232	93.64
1684575	1.114	0.9366544	null	conserved hypothetical protein	PA3468	222	97.1
1684578	1.244	1.0435287	null	hypothetical protein	PA3472	87	92.54
1684581	1.244	1.0234867	null	probable malic enzyme	PA3471	1	93.12
1684584	0.994	0.40875435	null	probable sensor/response regulator hybrid	PA3462	268	94.78
1684587	1.018	0.87641394	null	hypothetical protein	PA3473	39	95.48
1684590	0.305	0.11592404	pheC	cyclohexadienyl dehydratase precursor	PA3475	125	93.1
1684593	0.541	0.29787976	rhlR	transcriptional regulator RhlR	PA3477	141	94.85
1684596	0.336	0.29095212	rhlI	autoinducer synthesis protein RhlI	PA3476	53	92.12
1684599	1.370	1.2957098	null	probable ATP-dependent RNA helicase	PA3466	2	92.81
1684602	4.532	-0.031111062	null	probable major facilitator superfamily (MFS)	PA3467	327	92.7
1684605	1.682	0.9234684	rhlB	rhamnosyltransferase chain B	PA3478	131	95.36
1684608	0.650	0.36208993	rhlA	rhamnosyltransferase chain A	PA3479	1	93.47
1684611	1.343	0.9945196	null	conserved hypothetical protein	PA3469	481	92.21
1684614	0.904	0.7949503	null	conserved hypothetical protein	PA3474	14	92.1
1684617	1.083	0.7006582	null	probable glutamine amidotransferase	PA3459	40	97.2
1684620	0.799	0.70362216	null	hypothetical protein	PA3483	416	95.06
1684623	1.127	0.8236372	null	conserved hypothetical protein	PA3481	1	93.66
1684626	0.520	0.42640203	null	hypothetical protein	PA3484	256	92.62
1684629	0.673	1.3136418	null	hypothetical protein	PA3488	193	94.12
1684632	0.776	0.21482734	null	hypothetical protein	PA3456	361	97.55
1684635	0.755	0.5561967	null	probable deoxycytidine triphosphate deaminase	PA3480	13	92.77
1684638	4.471	2.8208547	null	probable ferredoxin	PA3490	459	93.84
1684641	1.258	1.0571244	null	probable ferredoxin	PA3491	246	96.96
1684644	0.116	1.049301	null	conserved hypothetical protein	PA3493	416	93.93
1684647	0.882	0.8165945	metG	methionyl-tRNA synthetase	PA3482	1	92.1
1684650	1.623	1.2389458	nth	endonuclease III	PA3495	25	93.3
1684653	0.990	0.77990454	null	conserved hypothetical protein	PA3494	143	96.31
1684656	0.860	0.3212667	null	conserved hypothetical protein	PA3489	359	93.01
1684661	0.238	-0.4102736	null	hypothetical protein	PA3485	340	96.93
1684662	1.076	-0.39674798	null	probable oxidoreductase	PA3498	182	95.7
1684665	0.935	0.6478143	null	hypothetical protein	PA3496	59	94.5
1684667	1.133	8.375	null	hypothetical protein	PA3497	173	93.3
1684670	1.198	1.0334874	null	conserved hypothetical protein	PA3492	371	93.32
1684673	1.009	4.7789373	null	hypothetical protein	PA3499	209	92.36
1684676	1.014	-0.041937236	null	hypothetical protein	PA3503	1	94.4
1684679	1.061	0.7416363	null	hypothetical protein	PA3502	98	94.15
1684681	1.039	0.90476847	null	hypothetical protein	PA3501	1	93.27
1684683	2.276	0.59685105	null	conserved hypothetical protein	PA3500	1	92.93

1684686	0.281	-1.4011071	null	hypothetical protein	PA3510	1	93.6
1684689	1.016	1.5025375	null	probable transcriptional regulator	PA3508	13	92.48
1684692	0.822	-3.2322807	null	probable decarboxylase	PA3506	72	92.19
1684695	1.009	2.7107143	null	hypothetical protein	PA3505	71	92.64
1684698	1.177	1.0244242	null	probable permease of ABC transporter	PA3512	256	95.47
1684701	1.173	1.1847222	null	probable short-chain dehydrogenase	PA3507	310	93.9
1684704	0.585	0.770121	null	hypothetical protein	PA3513	394	92.17
1684707	1.017	0.5486957	null	hypothetical protein	PA3515	212	93.6
1684710	4.421	0.78377193	null	probable hydrolase	PA3509	778	96.12
1684713	1.009	0.3637987	null	probable short-chain dehydrogenase	PA3511	293	92.15
1684716	1.009	2.5716667	null	probable aldehyde dehydrogenase	PA3504	188	92.95
1684719	1.009	2.7632117	null	hypothetical protein	PA3518	19	93.15
1684722	3.575	3.9535255	null	probable lyase	PA3517	55	92.03
1684725	1.133	2.356922	null	hypothetical protein	PA3520	29	95.09
1684727	1.390	1.1168252	null	probable ATP-binding component of ABC	PA3514	281	93.22
1684730	1.173	0.65119046	null	probable Resistance-Nodulation-Cell Division	PA3523	163	96.61
1684733	1.334	1.1584743	gloA1	lactoylglutathione lyase	PA3524	65	92.53
1684736	0.878	1.4532796	pldA	phospholipase D	PA3487	72	93.29
1684739	1.009	0.49932733	null	hypothetical protein	PA3519	56	92.24
1684742	1.167	0.36870798	null	probable Resistance-Nodulation-Cell Division	PA3522	126	97.54
1684745	1.053	0.85438263	null	probable outer membrane protein precursor	PA3526	122	95.37
1684748	0.871	0.7558738	argG	argininosuccinate synthase	PA3525	202	92.2
1684751	1.182	0.7394126	null	probable lyase	PA3516	3	92.57
1684754	1.184	0.9382858	null	conserved hypothetical protein	PA3530	6	92.4
1684756	0.186	0.4532168	null	probable outer membrane protein precursor	PA3521	158	96.49
1684759	1.317	1.0584779	null	conserved hypothetical protein	PA3486	1967	95.95
1684762	0.976	0.84613705	pyrC	dihydroorotase	PA3527	1	95.24
1684765	0.918	0.940359	bfrB	bacterioferritin	PA3531	122	92.49
1684768	1.280	0.88040984	null	probable oxidoreductase	PA3534	1	93.4
1684771	0.790	0.53936493	null	hypothetical protein	PA3536	169	94.17
1684774	1.436	1.3607398	null	probable peroxidase	PA3529	20	93.33
1684777	1.073	0.74101293	null	probable ATP-binding component of ABC	PA3538	478	93.52
1684780	0.995	0.8708106	rnt	ribonuclease T	PA3528	158	92.59
1684783	1.156	1.0216246	null	conserved hypothetical protein	PA3533	33	92.28
1684786	0.682	0.5471942	null	conserved hypothetical protein	PA3539	19	97.91
1684789	0.806	0.69397116	argF	ornithine carbamoyltransferase, anabolic	PA3537	106	93.01
1684792	1.009	-1.3097746	alg44	alginate biosynthesis protein Alg44	PA3542	6	92.53
1684795	0.987	0.04166875	null	hypothetical protein	PA3532	385	93.55
1684798	0.963	0.776882	algD	GDP-mannose 6-dehydrogenase AlgD	PA3540	18	93.06
1684801	5.439	2.4167585	algL	poly(beta-d-mannuronate) lyase precursor AlgL	PA3547	175	92.73
1684804	1.009	2.6943157	algG	alginate-c5-mannuronan-epimerase AlgG	PA3545	649	94.37
1684807	0.453	0.33233765	algF	alginate o-acetyltransferase AlgF	PA3550	176	97.73
1684810	1.430	1.1959003	algJ	alginate o-acetyltransferase AlgJ	PA3549	64	96.84
1684813	0.909	1.2771614	algX	alginate biosynthesis protein AlgX	PA3546	137	93.18
1684816	1.339	0.6383394	algA	phosphomannose isomerase / guanosine	PA3551	1	92.36
1684819	0.675	0.124885656	algE	Alginate production outer membrane protein AlgE	PA3544	1	96.89
1684822	1.187	0.86675626	null	conserved hypothetical protein	PA3555	319	97.39
1684825	1.511	1.2622762	null	conserved hypothetical protein	PA3552	2	92.73
1684828	0.815	0.72564054	null	probable serine protease	PA3535	1	95.02
1684831	1.136	0.72287476	algI	alginate o-acetyltransferase AlgI	PA3548	27	92.59
1684835	1.918	1.6659908	null	conserved hypothetical protein	PA3557	40	97.48
1684837	1.092	0.79353464	null	hypothetical protein	PA3558	280	92.57
1684840	1.093	0.83246565	fruK	1-phosphofructokinase	PA3561	197	96.16
1684843	1.730	1.2980622	null	probable glycosyl transferase	PA3553	253	93.06
1684846	1.102	0.9656641	fruR	fructose transport system repressor FruR	PA3563	188	94.68
1684849	1.674	1.0091293	null	probable nucleotide sugar dehydrogenase	PA3559	50	92.9
1684852	1.163	0.87761897	null	probable phosphotransferase system enzyme I	PA3562	1	95.86
1684856	0.910	0.72047174	null	conserved hypothetical protein	PA3564	184	92.69
1684858	1.010	0.7182725	null	conserved hypothetical protein	PA3566	133	92.56
1684859	1.256	0.6343342	algK	alginate biosynthetic protein AlgK precursor	PA3543	405	92.53
1684862	1.526	1.074137	null	probable transcriptional regulator	PA3565	1	92.25
1684865	0.904	0.78977746	null	probable oxidoreductase	PA3567	6	92.14
1684868	1.237	0.982872	null	hypothetical protein	PA3572	2	93.84
1684870	1.206	0.4524971	arnT	inner membrane L-Ara4N transferase ArnT	PA3556	57	92.77
1684873	1.528	1.2345933	null	conserved hypothetical protein	PA3554	3	92.03
1684876	1.221	1.0799999	mmsR	transcriptional regulator MmsR	PA3571	6	92.63
1684879	1.009	1.1388547	alg8	alginate biosynthesis protein Alg8	PA3541	248	95.04
1684882	1.228	1.2034552	null	hypothetical protein	PA3576	147	95.84
1684885	0.942	0.6344109	null	probable transcriptional regulator	PA3574	159	92.01
1684888	1.233	0.99898416	null	conserved hypothetical protein	PA3578	1	93.68
1684891	0.917	0.67863727	null	hypothetical protein	PA3577	14	93.81
1684892	0.663	0.2227674	fruA	phosphotransferase system, fructose-specific	PA3560	87	97.09
1684895	1.009	0.39743596	mmsB	3-hydroxyisobutyrate dehydrogenase	PA3569	61	93.21
1684898	1.105	0.7362343	null	probable major facilitator superfamily (MFS)	PA3573	317	96.99
1684901	0.957	0.68871105	null	conserved hypothetical protein	PA3580	37	96.22
1684904	1.189	0.9837596	null	hypothetical protein	PA3575	1	93.53
1684907	0.852	0.69152653	null	hypothetical protein	PA3470	235	95.31
1684909	1.260	1.1000466	glpM	membrane protein GlpM	PA3585	82	92.14
1684910	1.486	1.2984383	glpD	glycerol-3-phosphate dehydrogenase	PA3584	18	94.92
1684913	0.941	0.6449245	glpR	glycerol-3-phosphate regulon repressor	PA3583	43	92.5
1684916	1.378	1.0328349	glpK	glycerol kinase	PA3582	146	93.31
1684919	0.943	-0.6598139	null	probable porin	PA3588	472	93
1684922	1.510	1.3336976	null	probable acyl-CoA thiolase	PA3589	530	95.95
1684925	1.298	1.1010336	glpF	glycerol uptake facilitator protein	PA3581	306	92.98
1684928	1.337	1.1999552	null	probable hydrolase	PA3586	11	93.79
1684931	1.009	5.9583335	null	conserved hypothetical protein	PA3592	169	92
1684934	9.373	1.4588772	null	probable enoyl-CoA hydratase/isomerase	PA3591	61	97.7
1684937	1.009	1.4610102	null	probable acyl-CoA dehydrogenase	PA3593	239	93.15
1684940	1.047	-0.4583333	null	probable acetyl-coa synthetase	PA3568	231	97.6
1684943	1.219	1.0634519	null	probable methylated-DNA--protein-cysteine	PA3596	2	92.69
1684946	1.263	1.0091965	null	probable hydroxyacyl-CoA dehydrogenase	PA3590	61	95.55
1684949	1.230	0.8252219	null	probable carbohydrate kinase	PA3579	295	92.91
1684952	1.125	0.7141408	null	conserved hypothetical protein	PA3600	70	92.46
1684954	0.952	0.64069164	null	conserved hypothetical protein	PA3598	105	94.81
1684957	1.088	0.81692874	null	conserved hypothetical protein	PA3601	6	93
1684959	1.496	0.7470457	null	probable transcriptional regulator	PA3599	90	93.1
1684962	0.644	1.2935431	null	probable transcriptional regulator	PA3594	451	92.61
1684965	0.848	0.6779803	null	probable two-component response regulator	PA3604	441	95.09
1684968	0.762	0.18455933	dgkA	diacylglycerol kinase	PA3603	265	92.09
1684971	1.009	2.1291614	null	hypothetical protein	PA3248	52	95.92
1684974	1.077	0.7593323	null	conserved hypothetical protein	PA3606	297	94.66
1684977	1.425	0.7556302	potC	polyamine transport protein PotC	PA3609	344	92.54
1684980	1.182	0.9522065	potB	polyamine transport protein PotB	PA3608	70	94.29
1684983	1.062	0.87651414	null	probable major facilitator superfamily (MFS)	PA3595	173	94.42
1684986	1.192	1.0114774	null	hypothetical protein	PA3611	87	93.44
1684989	0.697	0.57864505	null	conserved hypothetical protein	PA3612	41	93.64
1684991	0.830	0.58898574	null	hypothetical protein	PA3605	27	95.19
1684994	1.009	0.42833334	mmsA	methylmalonate-semialdehyde dehydrogenase	PA3570	1051	95.42
1684997	0.734	0.64244586	null	conserved hypothetical protein	PA3616	165	92.11

1685000	2.978	2.246249	null	conserved hypothetical protein	PA3602	182	92.6
1685003	1.030	0.75838864	null	hypothetical protein	PA3615	9	92.29
1685006	1.277	1.0707804	null	hypothetical protein	PA3613	112	96.63
1685009	1.064	0.519249	null	hypothetical protein	PA3619	136	93.31
1685012	0.773	0.80659837	rpoS	sigma factor RpoS	PA3622	90	93.75
1685015	0.760	0.6594265	fdxA	ferredoxin I	PA3621	227	92.59
1685017	0.722	0.5987843	null	conserved hypothetical protein	PA3623	32	95.66
1685020	1.240	0.89968395	null	probable amino acid permease	PA3597	798	92.41
1685023	1.022	0.8370274	null	hypothetical protein	PA3614	27	92.06
1685026	1.159	1.0301201	recA	RecA protein	PA3617	103	96.71
1685029	1.010	0.88462603	pcm	L-isoaspartate protein carboxylmethyltransferase	PA3624	27	92.57
1685032	0.809	0.5910133	null	conserved hypothetical protein	PA3618	151	92.23
1685035	1.294	1.1083076	null	conserved hypothetical protein	PA3626	99	93.57
1685038	0.942	0.82154626	surE	survival protein SurE	PA3625	351	95.64
1685041	0.883	0.71954566	null	probable esterase	PA3628	159	92.14
1685044	1.047	0.7167084	potA	polyamine transport protein PotA	PA3607	48	92.32
1685047	1.144	0.9347757	adhC	alcohol dehydrogenase class III	PA3629	116	94.43
1685050	1.233	0.57792604	null	probable transcriptional regulator	PA3630	171	94.23
1685053	0.824	0.61104125	ygbB	2C-methyl-D-erythritol 2,4-cyclodiphosphate	PA3627	392	94.17
1685057	0.825	-0.13170865	null	conserved hypothetical protein	PA3632	201	94.01
1685058	0.756	0.7271522	null	conserved hypothetical protein	PA3634	4	92.09
1685060	0.780	0.6429812	ygbP	4-diphosphocytidyl-2-C-methylerythritol	PA3633	3	96.07
1685063	0.943	0.7873236	pyrG	CTP synthase	PA3637	45	92.11
1685066	0.787	0.66474366	accA	acetyl-coenzyme A carboxylase carboxyl	PA3639	186	93.07
1685069	0.923	0.81509113	kdsA	2-dehydro-3-deoxyphosphooctonate aldolase	PA3636	174	94.2
1685072	0.462	-0.44870275	rhlG	beta-ketoacyl reductase	PA3387	502	94.34
1685075	0.633	0.5342382	rnhB	ribonuclease HII	PA3642	7	92.06
1685078	0.731	0.63641655	lpxA	UDP-N-acetylglucosamine acyltransferase	PA3644	87	94.11
1685081	0.563	0.46330124	lpxB	lipid A-disaccharide synthase	PA3643	172	94.8
1685084	1.065	0.69703376	null	conserved hypothetical protein	PA3631	159	92.7
1685087	0.628	0.56493485	lpxD	UDP-3-O-[3-hydroxylauroyl] glucosamine	PA3646	645	92.2
1685090	0.770	0.61212605	null	probable outer membrane protein precursor	PA3648	25	92.44
1685093	0.878	0.6597815	null	conserved hypothetical protein	PA3638	83	95.61
1685096	0.850	0.72396165	null	probable outer membrane protein precursor	PA3647	103	93.82
1685099	0.644	0.45967907	dxr	1-deoxy-d-xylulose 5-phosphate reductoisomerase	PA3650	450	94.78
1685102	0.993	0.74862325	cdsA	phosphatidate cytidylyltransferase	PA3651	187	94.64
1685105	0.836	0.70435023	null	conserved hypothetical protein	PA3649	39	92.8
1685108	0.838	0.73007554	uppS	undecaprenyl pyrophosphate synthetase	PA3652	151	95.45
1685111	0.909	0.70765275	pyrH	uridylate kinase	PA3654	69	92.48
1685114	0.983	0.5860216	null	probable amino acid permease	PA3641	119	93.36
1685117	1.000	0.96831834	tsf	elongation factor Ts	PA3655	37	94.23
1685120	1.035	0.8967749	map	methionine aminopeptidase	PA3657	70	92.34
1685123	1.055	1.158318	rpsB	30S ribosomal protein S2	PA3656	40	92.24
1685126	0.699	0.5337745	dnaE	DNA polymerase III, alpha chain	PA3640	252	92.61
1685129	1.249	1.1970044	frr	ribosome recycling factor	PA3653	111	92.28
1685132	0.720	0.57324666	glnD	protein-PII uridylyltransferase	PA3658	1	92.95
1685135	1.093	1.7921053	null	hypothetical protein	PA3661	210	92.9
1685138	1.132	1.0732355	null	hypothetical protein	PA3662	99	92.17
1685141	0.506	0.3610223	null	hypothetical protein	PA3663	12	92.13
1685144	0.748	0.63772094	null	probable aminotransferase	PA3659	6	92.79
1685147	0.817	0.64812714	eno	enolase	PA3635	26	95.72
1685150	0.844	0.7360501	null	hypothetical protein	PA3665	232	94.95
1685153	0.424	0.042462364	null	conserved hypothetical protein	PA3668	41	96.54
1685157	0.926	0.7304977	null	conserved hypothetical protein	PA3664	29	93.25
1685158	0.947	0.7460544	dapD	tetrahydrodipicolinate succinylase	PA3666	119	96.95
1685161	0.467	0.30948222	null	probable sodium/hydrogen antiporter	PA3660	40	96.5
1685166	3.776	0.35850817	null	probable permease of ABC transporter	PA3671	596	95.97
1685167	0.610	0.101576075	null	hypothetical protein	PA3669	138	96.37
1685170	0.733	0.20491162	null	probable pyridoxal-phosphate dependent enzyme	PA3667	254	93.56
1685173	1.463	1.0531385	null	hypothetical protein	PA3674	40	93.63
1685176	0.693	0.31315443	null	hypothetical protein	PA3675	576	92.86
1685179	0.750	0.34410852	null	hypothetical protein	PA3681	618	96.92
1685182	0.868	0.63682663	null	probable transcriptional regulator	PA3678	139	92.52
1685185	1.005	0.5797544	null	probable ATP-binding component of ABC	PA3672	3	92.37
1685188	1.066	0.94123095	null	conserved hypothetical protein	PA3680	13	97.55
1685191	0.517	-0.31550306	null	hypothetical protein	PA3682	253	95.18
1685194	0.911	0.80514824	null	hypothetical protein	PA3684	265	95.13
1685195	0.784	0.5854407	null	conserved hypothetical protein	PA3683	110	92.56
1685198	0.836	0.8094512	adk	adenylate kinase	PA3686	227	95.19
1685201	0.633	0.21232924	null	probable Resistance-Nodulation-Cell Division	PA3676	5	92.11
1685204	1.965	1.4361111	null	hypothetical protein	PA3688	172	92.13
1685206	0.759	0.6084792	null	conserved hypothetical protein	PA3685	1	92.95
1685209	1.577	1.4703363	null	hypothetical protein	PA3691	165	95.26
1685212	0.926	0.7667542	null	probable transcriptional regulator	PA3689	132	93.56
1685215	0.924	0.6357788	plsB	glycerol-3-phosphate acyltransferase	PA3673	121	93.56
1685218	0.772	0.53677976	null	hypothetical protein	PA3679	425	94.16
1685221	0.990	0.6652519	null	hypothetical protein	PA3694	60	92.63
1685224	1.918	1.6735195	null	probable outer membrane protein precursor	PA3692	188	93.61
1685227	0.796	0.5635418	null	conserved hypothetical protein	PA3693	5	95.45
1685230	1.061	0.79467314	null	hypothetical protein	PA3697	82	93.73
1685233	0.328	-0.08145668	null	conserved hypothetical protein	PA3696	1	93.26
1685236	0.842	0.50913775	null	probable metal-transporting P-type ATPase	PA3690	128	94.55
1685239	0.896	0.78385943	null	hypothetical protein	PA3698	18	93.18
1685242	0.976	0.7392643	null	probable transcriptional regulator	PA3699	237	92.38
1685245	0.919	0.64924634	wspF	probable methylesterase	PA3703	2	92.63
1685248	0.955	0.83089316	null	hypothetical protein	PA3695	39	93.39
1685251	1.181	0.8263639	lysS	lysyl-tRNA synthetase	PA3700	518	94.75
1685254	0.999	0.8179579	wspB	hypothetical protein	PA3707	28	92.44
1685257	0.985	0.7318284	ppc	phosphoenolpyruvate carboxylase	PA3687	212	94.2
1685260	0.620	0.3795069	wspD	hypothetical protein	PA3705	24	92.55
1685263	0.651	0.52133596	wspC	probable protein methyltransferase	PA3706	276	96.52
1685266	1.279	0.99423105	wspA	probable chemotaxis transducer	PA3708	72	92.68
1685269	0.974	-2.7709594	null	probable transcriptional regulator	PA3711	229	93.9
1685272	0.720	0.45677972	metR	transcriptional regulator MetR	PA3587	299	92.08
1685275	0.655	0.3601941	wspE	probable chemotaxis sensor/effector fusion	PA3704	1	92.9
1685278	0.929	0.37077156	wspR	probable two-component response regulator	PA3702	70	92.76
1685281	0.702	0.49554914	null	hypothetical protein	PA3712	627	94.95
1685284	1.092	0.70395726	null	probable two-component response regulator	PA3714	27	94.29
1685287	1.364	1.0232809	null	probable peptidyl-prolyl cis-trans isomerase,	PA3717	38	92.91
1685290	0.535	0.041287873	null	hypothetical protein	PA3719	115	95.25
1685292	1.013	0.3838158	null	hypothetical protein	PA3715	82	97.43
1685295	1.365	0.7198342	null	hypothetical protein	PA3713	344	95.76
1685298	0.807	0.40374175	null	probable GMC-type oxidoreductase	PA3710	119	93.45
1685301	1.009	0.70895827	null	hypothetical protein	PA3722	111	95
1685304	1.028	0.8585059	null	hypothetical protein	PA3720	104	92.51
1685307	1.030	0.18184423	null	probable transcriptional regulator	PA3721	56	92.37
1685310	0.963	0.55374706	null	hypothetical protein	PA3670	637	95.02
1685313	0.404	0.31293103	null	probable major facilitator superfamily (MFS)	PA3709	1	92.08
1685316	1.208	-0.032637335	null	probable major facilitator superfamily (MFS)	PA3718	161	93.49

1685319	1.009	0.5166503	null	probable FMN oxidoreductase	PA3723	50	95.55
1685322	1.009	-0.6738095	lasB	elastase LasB	PA3724	74	94.91
1685325	0.997	0.86604595	null	hypothetical protein	PA3716	1	93.63
1685328	0.600	0.49542913	null	hypothetical protein	PA3730	240	92.23
1685331	1.106	0.8094447	recJ	single-stranded-DNA-specific exonuclease RecJ	PA3725	286	94.64
1685334	0.769	0.6374597	null	conserved hypothetical protein	PA3731	392	93.28
1685337	0.842	0.70449305	null	hypothetical protein	PA3727	327	92.25
1685340	0.647	0.3064054	null	conserved hypothetical protein	PA3726	429	92.7
1685343	1.009	-0.5473856	null	hypothetical protein	PA3734	1	94.81
1685346	0.574	0.50019276	null	conserved hypothetical protein	PA3732	100	92.2
1685349	0.945	0.7637297	thrC	threonine synthase	PA3735	20	96.01
1685352	0.931	0.6446042	xerD	integrase/recombinase XerD	PA3738	8	92.75
1685355	0.704	0.47518712	mutS	DNA mismatch repair protein MutS	PA3620	448	92.78
1685358	0.949	0.81069136	dsbC	thiol:disulfide interchange protein DsbC	PA3737	205	92.38
1685361	1.052	3.706337	null	hypothetical protein	PA3740	24	92.86
1685364	1.266	0.9206847	null	hypothetical protein	PA3733	461	94.01
1685367	0.672	0.69219214	trmD	tRNA (guanine-N1)-methyltransferase	PA3743	9	92.83
1685370	0.994	0.80752295	null	hypothetical protein	PA3741	168	92.7
1685373	0.884	0.8359148	rpsP	30S ribosomal protein S16	PA3745	36	93.23
1685375	1.040	0.9092698	hom	homoserine dehydrogenase	PA3736	3	92.59
1685378	0.956	0.8414547	rplS	50S ribosomal protein L19	PA3742	176	94.23
1685381	0.669	0.70527554	rimM	16S rRNA processing protein	PA3744	33	92.5
1685384	0.704	0.47234663	null	conserved hypothetical protein	PA3729	69	92.24
1685387	0.657	0.50841343	null	hypothetical protein	PA3752	28	94.39
1685388	0.961	0.84217227	null	conserved hypothetical protein	PA3747	8	94.39
1685391	0.942	0.6198986	purT	phosphoribosylglycinamide formyltransferase 2	PA3751	205	92.84
1685394	1.130	0.98280317	null	hypothetical protein	PA3754	94	95.89
1685397	0.665	0.5327674	null	conserved hypothetical protein	PA3753	127	93.39
1685400	0.983	0.71609735	ffh	signal recognition particle protein Ffh	PA3746	9	92.41
1685403	1.093	0.9740489	null	hypothetical protein	PA3756	16	92.66
1685406	0.679	0.56068075	null	hypothetical protein	PA3750	156	94.96
1685409	0.632	2.2369266	null	probable transcriptional regulator	PA3757	121	93.02
1685412	0.970	0.7372337	null	conserved hypothetical protein	PA3755	390	93.29
1685415	0.811	0.48322812	null	hypothetical protein	PA3728	1	92.54
1685418	9.467	2.5791638	null	probable aminotransferase	PA3759	87	95.89
1685421	0.989	0.80957204	null	hypothetical protein	PA3762	81	95.89
1685424	0.654	0.38482323	null	conserved hypothetical protein	PA3748	728	92.38
1685427	1.593	0.44781852	null	hypothetical protein	PA3765	121	93.66
1685429	1.107	0.6771899	null	probable sodium/hydrogen antiporter	PA3739	492	92.22
1685432	0.898	0.6831032	null	conserved hypothetical protein	PA3764	28	96.15
1685435	0.822	0.51039183	null	probable N-acetylglucosamine-6-phosphate	PA3758	107	93.07
1685438	0.804	0.6454195	null	conserved hypothetical protein	PA3767	290	92.85
1685441	1.849	1.0162529	null	probable major facilitator superfamily (MFS)	PA3749	131	93.37
1685444	0.503	0.14225985	null	probable transcriptional regulator	PA3771	2	92.81
1685447	0.975	0.7184499	null	probable metallo-oxidoreductase	PA3768	386	95.61
1685450	1.164	0.6567248	null	probable aromatic amino acid transporter	PA3766	1	93.02
1685453	0.993	0.7628602	guaA	GMP synthase	PA3769	158	95.29
1685456	0.818	0.6580393	purL	phosphoribosylformylglycinamidine synthase	PA3763	19	95.5
1685459	1.009	2.177448	null	hypothetical protein	PA3772	492	94.87
1685462	1.179	1.7001709	null	probable phosphotransferase protein	PA3760	1	93.82
1685465	1.177	1.0524023	guaB	inosine-5'-monophosphate dehydrogenase	PA3770	108	92.19
1685468	0.712	0.6030881	fabZ	(3R)-hydroxymyristoyl-[acyl carrier protein]	PA3645	365	92.14
1685471	1.009	1.883501	null	hypothetical protein	PA3773	648	92.03
1685474	1.009	2.125	null	probable phosphotransferase system protein	PA3761	324	93.13
1685477	1.151	0.4314018	null	probable transcriptional regulator	PA3776	754	97.6
1685480	1.009	-0.84697664	null	probable acetylpolyamine aminohydrolase	PA3774	234	92.22
1685483	1.009	2.1962206	null	hypothetical protein	PA3783	62	92.24
1685486	0.951	0.76582164	null	probable transcriptional regulator	PA3782	29	94.72
1685489	1.259	0.48581457	null	hypothetical protein	PA3775	237	93.77
1685493	1.385	0.3456595	null	hypothetical protein	PA3784	129	92.93
1685495	0.704	0.54428065	null	probable transcriptional regulator	PA3778	19	93.26
1685498	1.183	1.127948	null	conserved hypothetical protein	PA3785	251	92.6
1685501	0.964	0.8556207	null	hypothetical protein	PA3786	163	93.63
1685504	0.566	0.23371239	null	conserved hypothetical protein	PA3787	225	96.48
1685507	1.009	0.7079961	xseA	exodeoxyribonuclease VII large subunit	PA3777	245	94.53
1685512	1.544	0.91314065	null	hypothetical protein	PA3780	114	92.09
1685513	1.181	1.0349729	null	hypothetical protein	PA3788	11	93.49
1685515	2.091	-1.9597416	null	hypothetical protein	PA3789	99	92.57
1685518	0.909	0.5542941	null	hypothetical protein	PA3779	640	93.1
1685521	1.071	0.47586495	null	hypothetical protein	PA3794	74	92.1
1685525	1.286	1.333947	null	hypothetical protein	PA3791	72	95.71
1685528	0.899	0.6542163	null	hypothetical protein	PA3793	70	93.85
1685529	1.153	0.8436888	null	hypothetical protein	PA3796	25	95.59
1685532	1.160	1.1232	null	probable aminotransferase	PA3798	66	92.39
1685535	1.019	0.86233026	null	conserved hypothetical protein	PA3797	33	95.95
1685538	0.838	0.6954	null	hypothetical protein	PA3804	2	95.21
1685541	1.292	1.0067661	null	probable oxidoreductase	PA3795	128	92.69
1685544	0.937	0.80987465	null	conserved hypothetical protein	PA3800	107	92.41
1685547	0.800	0.690908	null	conserved hypothetical protein	PA3801	148	92.52
1685550	0.899	0.7201846	null	conserved hypothetical protein	PA3799	1	94.01
1685553	0.587	0.4701303	null	conserved hypothetical protein	PA3808	27	92.3
1685555	0.755	0.6550501	pilF	type 4 fimbrial biogenesis protein PilF	PA3805	19	93.83
1685558	1.171	0.9790286	hisS	histidyl-tRNA synthetase	PA3802	117	93.15
1685561	0.962	0.8369935	leuA	2-isopropylmalate synthase	PA3792	23	92.15
1685564	1.103	1.1401482	iscA	probable iron-binding protein IscA	PA3812	59	96.93
1685567	0.893	0.88832885	null	conserved hypothetical protein	PA3806	54	92.4
1685570	0.844	0.7852403	fdx2	ferredoxin (2Fe-2S)	PA3809	1	93.88
1685573	0.992	0.9878014	hscB	heat shock protein HscB	PA3811	207	93.98
1685576	0.955	0.82804203	gcpE	probable isoprenoid biosynthetic protein GcpE	PA3803	294	95.96
1685579	1.313	1.4028064	iscU	probable iron-binding protein IscU	PA3813	159	92.41
1685582	0.916	0.8111693	hscA	heat shock protein HscA	PA3810	121	97.83
1685586	0.870	0.7554364	null	conserved hypothetical protein	PA3815	75	92.31
1685588	0.839	0.7111465	null	probable methyltransferase	PA3817	296	95.53
1685591	1.060	0.9397315	iscS	L-cysteine desulfurase (pyridoxal	PA3814	20	95.48
1685594	1.021	0.81844693	cysE	O-acetylserine synthase	PA3816	25	92.84
1685597	1.078	0.9642075	ndk	nucleoside diphosphate kinase	PA3807	97	92.94
1685600	0.946	0.8196298	secF	secretion protein SecF	PA3820	32	94.91
1685603	1.833	1.5906445	null	conserved hypothetical protein	PA3819	71	93.46
1685606	0.844	0.8214847	null	conserved hypothetical protein	PA3822	62	92.67
1685609	0.799	0.55861604	tgt	queuine tRNA-ribosyltransferase	PA3823	91	97.88
1685612	1.030	0.8437101	queA	S-adenosylmethionine:trna	PA3824	3	92.24
1685615	1.271	0.9993162	null	extragenic suppressor protein SuhB	PA3818	263	93.95
1685618	0.887	1.1385715	oprC	Putative copper transport outer membrane porin	PA3790	149	94.68
1685621	1.276	0.52791727	null	hypothetical protein	PA3826	126	92.36
1685624	0.986	-0.42809525	null	probable transcriptional regulator	PA3830	1	96.92
1685627	0.621	-1.5534465	null	conserved hypothetical protein	PA3828	249	95.39
1685630	1.216	0.82367694	null	hypothetical protein	PA3829	163	92.05
1685633	0.888	0.772972	null	hypothetical protein	PA3833	81	92.67
1685635	0.980	0.72321796	null	conserved hypothetical protein	PA3827	457	94.49

1685638	2.308	0.6162837	null	hypothetical protein	PA3835	90	92.82
1685641	1.253	1.008947	null	hypothetical protein	PA3836	174	92.16
1685644	0.807	0.64960897	holC	DNA polymerase III, chi subunit	PA3832	291	94.69
1685647	1.000	0.9635972	secD	secretion protein SecD	PA3821	44	92.04
1685650	0.901	0.71199995	null	probable permease of ABC transporter	PA3837	45	92.29
1685653	0.624	1.7791836	null	conserved hypothetical protein	PA3840	55	96.2
1685656	1.051	0.6732748	null	probable chaperone	PA3842	36	92.43
1685659	0.015	-0.045893095	null	hypothetical protein	PA3843	158	93.79
1685662	1.009	1.0493464	null	hypothetical protein	PA3825	109	96.48
1685665	0.848	0.7015432	pepA	leucine aminopeptidase	PA3831	170	95.13
1685668	0.041	1.45E-04	null	hypothetical protein	PA3844	542	92.81
1685671	0.875	0.6951378	null	probable ATP-binding component of ABC	PA3838	2	92.78
1685674	0.516	0.43296868	exoS	exoenzyme S	PA3841	270	93.85
1685677	1.094	0.72761714	null	probable sodium:sulfate symporter	PA3839	101	92.64
1685680	0.630	0.37901458	null	hypothetical protein	PA3846	192	93.31
1685683	0.875	0.62775695	valS	valyl-tRNA synthetase	PA3834	9	92.28
1685686	1.105	0.79499775	null	probable transcriptional regulator	PA3845	256	97.64
1685689	1.121	0.80216867	null	conserved hypothetical protein	PA3849	2	92.17
1685692	2.000	1.0685589	null	conserved hypothetical protein	PA3847	30	96.86
1685695	0.826	0.7186223	null	hypothetical protein	PA3854	22	95.24
1685697	0.661	0.48754054	null	probable transferase	PA3853	231	92.05
1685700	0.956	0.8069366	null	hypothetical protein	PA3852	55	92.27
1685703	1.227	1.185154	null	hypothetical protein	PA3850	190	94
1685706	0.640	0.3003559	null	hypothetical protein	PA3856	267	95.84
1685708	0.704	0.43188438	null	hypothetical protein	PA3855	171	93.38
1685711	0.447	-0.7433203	null	hypothetical protein	PA3851	342	92.15
1685714	1.134	0.87304574	null	carboxylesterase	PA3859	1	94.44
1685717	0.918	0.65830386	null	probable amino acid-binding protein	PA3858	155	95.84
1685720	1.042	0.73472434	null	hypothetical protein	PA3864	1	94.28
1685723	1.289	0.9703601	null	probable amino acid binding protein	PA3865	272	96.71
1685726	1.069	8.235715	null	hypothetical protein	PA3862	206	93
1685729	1.100	1.4618183	null	hypothetical protein	PA3868	102	94.06
1685732	1.009	1.0022922	null	probable DNA invertase	PA3867	31	92.61
1685735	13.867	12.730555	null	hypothetical protein	PA3869	112	92.56
1685738	0.898	0.7809126	rhl	ATP-dependent RNA helicase RhlB	PA3861	49	94.85
1685741	0.652	0.44853765	null	probable AMP-binding enzyme	PA3860	71	95.08
1685744	1.333	1.1082151	null	pyocin protein	PA3866	623	93.06
1685747	1.620	1.147709	null	probable peptidyl-prolyl cis-trans isomerase,	PA3871	89	96.14
1685750	1.075	-1.3004186	null	hypothetical protein	PA3863	241	93.17
1685753	1.751	1.440956	narH	respiratory nitrate reductase beta chain	PA3874	51	92.59
1685756	0.977	0.5452946	pcs	phosphatidylcholine synthase	PA3857	15	93.08
1685759	2.487	1.852671	narJ	respiratory nitrate reductase delta chain	PA3873	652	93.51
1685762	1.101	0.9623356	moaA1	molybdopterin biosynthetic protein A1	PA3870	337	92.06
1685765	1.411	1.3177513	narI	respiratory nitrate reductase gamma chain	PA3872	567	94.68
1685768	0.900	0.8817709	null	hypothetical protein	PA3881	11	92.11
1685772	0.934	0.8140241	null	conserved hypothetical protein	PA3880	1	92.11
1685774	0.877	1.2581152	null	hypothetical protein	PA3848	354	95.85
1685777	1.643	1.3558598	narK2	nitrite extrusion protein 2	PA3876	328	96.07
1685780	0.966	0.80196506	null	hypothetical protein	PA3882	204	92.25
1685783	1.219	0.8356177	narX	two-component sensor NarX	PA3878	30	92.29
1685786	1.578	1.3751986	narK1	nitrite extrusion protein 1	PA3877	114	94.5
1685789	0.896	0.6548847	null	hypothetical protein	PA3884	293	93.88
1685791	0.442	0.1348042	null	probable short-chain dehydrogenase	PA3883	627	97.08
1685794	1.609	1.1883008	narG	respiratory nitrate reductase alpha chain	PA3875	55	97.67
1685797	1.009	1.8565476	potD	polyamine transport protein PotD	PA3610	96	92.19
1685800	0.915	0.6288654	nhaP	Na+/H+ antiporter NhaP	PA3887	124	92.79
1685803	1.143	0.8097229	null	hypothetical protein	PA3886	78	92.92
1685806	1.075	0.94424236	null	hypothetical protein	PA3885	110	95.43
1685809	1.895	0.43274918	null	probable binding protein component of ABC	PA3889	209	92.19
1685812	1.257	1.1026725	null	probable permease of ABC transporter	PA3888	563	92.95
1685815	1.493	0.9902719	null	probable permease of ABC transporter	PA3890	3	92.11
1685818	0.882	0.7126686	narL	two-component response regulator NarL	PA3879	381	95.77
1685821	1.781	0.62969667	null	probable transcriptional regulator	PA3898	3	92.43
1685824	1.009	-0.346345	null	hypothetical protein	PA3897	37	96.37
1685827	1.465	1.2896798	null	conserved hypothetical protein	PA3892	322	95.2
1685830	1.842	1.2230117	null	probable ATP-binding component of ABC	PA3891	51	93.39
1685833	1.009	1.984175	null	probable transmembrane sensor	PA3900	383	95.47
1685836	1.438	1.2368066	null	conserved hypothetical protein	PA3893	78	94.59
1685839	1.261	1.0283363	null	hypothetical protein	PA3902	222	93.47
1685842	0.862	0.5836245	null	probable 2-hydroxyacid dehydrogenase	PA3896	36	93.98
1685845	0.316	0.23855829	null	hypothetical protein	PA3905	275	93.82
1685848	0.419	0.326411	null	hypothetical protein	PA3904	152	92.07
1685851	0.965	-0.18566805	null	probable outer membrane protein precursor	PA3894	278	95.45
1685854	0.943	0.7117486	null	hypothetical protein	PA3906	216	97.39
1685855	0.685	0.5315796	null	hypothetical protein	PA3908	83	94.71
1685858	1.148	0.9376155	prfC	peptide chain release factor 3	PA3903	57	93.1
1685861	0.522	0.38730878	null	hypothetical protein	PA3907	328	94.58
1685864	0.991	0.8307272	null	probable protease	PA3913	170	94.63
1685867	1.009	-1.4426421	null	hypothetical protein	PA3909	68	92.22
1685870	1.572	0.89966094	null	probable transcriptional regulator	PA3895	3	92.54
1685873	1.069	0.9033333	null	probable sigma-70 factor, ECF subfamily	PA3899	458	94.05
1685876	0.865	0.6430403	moaE	molybdopterin converting factor, large subunit	PA3916	19	95.36
1685879	1.842	1.3757252	moaD	molybdopterin converting factor, small subunit	PA3917	145	94.59
1685882	0.842	0.6759492	null	conserved hypothetical protein	PA3911	368	95.45
1685884	1.094	0.8117483	null	conserved hypothetical protein	PA3912	371	95.42
1685887	2.289	1.9587042	moaB1	molybdopterin biosynthetic protein B1	PA3915	132	92.56
1685890	1.799	1.4640291	moeA1	molybdenum cofactor biosynthetic protein A1	PA3914	753	94.02
1685893	1.092	0.66226214	fecA	Fe(III) dicitrate transport protein FecA	PA3901	829	95.92
1685896	1.359	1.1276534	moaC	molybdopterin biosynthetic protein C	PA3918	196	93.25
1685899	1.040	0.77021605	null	probable transcriptional regulator	PA3921	3	93.03
1685902	1.349	0.6028935	null	hypothetical protein	PA3928	2	94.76
1685903	1.067	0.8306033	null	hypothetical protein	PA3923	1	93.82
1685906	0.618	0.21580735	null	probable metal transporting P-type ATPase	PA3920	81	97.89
1685909	1.009	1.0995971	null	probable medium-chain acyl-CoA ligase	PA3924	116	94.63
1685912	1.014	0.8776004	cioB	cyanide insensitive terminal oxidase	PA3929	231	92
1685915	1.154	1.0092033	null	conserved hypothetical protein	PA3919	1	95.72
1685918	0.773	0.49487048	null	conserved hypothetical protein	PA3922	440	92.73
1685921	1.206	0.88401383	null	probable acyl-CoA thiolase	PA3925	130	93.72
1685924	1.012	0.6853358	cioA	cyanide insensitive terminal oxidase	PA3930	6	92.44
1685927	0.502	0.43465933	null	conserved hypothetical protein	PA3931	9	95.46
1685930	1.035	3.3671978	null	probable major facilitator superfamily (MFS)	PA3926	36	92.22
1685933	0.370	-1.2756455	null	probable periplasmic taurine-binding protein	PA3938	274	92.51
1685936	1.161	0.55772567	null	probable transcriptional regulator	PA3927	167	94.42
1685939	0.326	0.09564934	null	hypothetical protein	PA3939	213	92.66
1685942	0.916	0.904668	null	probable DNA binding protein	PA3940	213	93.71
1685945	0.500	0.25884134	null	probable choline transporter	PA3933	96	97.29
1685949	1.096	0.6365258	tauD	taurine dioxygenase	PA3935	429	95.78
1685951	0.896	0.5967452	tesB	acyl-CoA thioesterase II	PA3942	24	92.5
1685954	1.279	0.8172178	null	probable permease of ABC taurine transporter	PA3936	1	93.44

1685957	1.050	0.7981472	null	conserved hypothetical protein	PA3944	478	93.53
1685960	1.287	0.9797368	null	conserved hypothetical protein	PA3943	16	94.52
1685963	0.943	0.67229855	null	conserved hypothetical protein	PA3945	127	92.17
1685966	3.161	2.211575	null	probable two-component response regulator	PA3947	45	93.94
1685969	0.807	0.554988	null	probable transcriptional regulator	PA3932	222	95.57
1685972	0.893	0.67908484	null	probable two-component response regulator	PA3948	156	92.29
1685976	0.729	0.51623905	null	hypothetical protein	PA3941	549	94.23
1685978	1.037	0.8365595	null	conserved hypothetical protein	PA3951	35	92.11
1685981	0.784	0.73590434	null	hypothetical protein	PA3949	495	97.75
1685984	1.032	0.72957456	null	hypothetical protein	PA3952	400	93.41
1685985	1.763	0.18276314	null	conserved hypothetical protein	PA3953	344	96.11
1685988	0.968	0.66824543	null	hypothetical protein	PA3956	7	93.35
1685991	1.074	-0.795671	null	hypothetical protein	PA3954	59	93
1685994	1.061	0.972019	null	hypothetical protein	PA3955	64	92.8
1685997	0.661	0.36287808	null	hypothetical protein	PA3959	440	92.19
1686000	0.772	0.007355964	null	probable ATP-binding component of ABC taurine	PA3937	737	93.93
1686003	1.009	2.1032043	null	probable two-component sensor	PA3946	82	93.01
1686006	0.893	0.7064959	null	hypothetical protein	PA3958	47	92.35
1686009	0.877	0.6582633	null	probable ATP-dependent RNA helicase	PA3950	401	93.81
1686012	1.213	0.812451	null	hypothetical protein	PA3964	26	94
1686015	0.738	0.5918711	null	hypothetical protein	PA3966	162	95.14
1686016	1.124	0.89008695	null	hypothetical protein	PA3962	1	95.65
1686019	0.471	-5.155476	null	hypothetical protein	PA3960	1	94.25
1686022	1.009	2.1966667	null	probable transcriptional regulator	PA3965	46	93.13
1686025	1.241	1.0235251	null	hypothetical protein	PA3967	10	94.2
1686028	2.448	-0.041612912	null	probable short-chain dehydrogenase	PA3957	486	92.43
1686031	0.768	0.6636771	null	hypothetical protein	PA3971	229	93.2
1686034	0.737	0.59360754	null	probable pseudouridine synthase	PA3968	382	93.61
1686037	0.220	1.7618306	null	conserved hypothetical protein	PA3969	97	93.25
1686040	1.153	0.86332244	null	probable transcriptional regulator	PA3973	54	96.98
1686045	0.777	0.6699266	thiE	thiamin-phosphate pyrophosphorylase	PA3976	372	94.66
1686046	1.000	0.96491164	null	probable transporter	PA3963	381	94.67
1686049	1.122	0.979229	ladS	Lost Adherence Sensor, LadS	PA3974	10	94.02
1686052	0.303	-1.1110606	null	probable ATP-dependent helicase	PA3961	235	97.36
1686055	1.179	0.9027632	thiD	phosphomethylpyrimidine kinase	PA3975	1	93.12
1686058	0.864	0.6934834	null	hypothetical protein	PA3978	243	94.66
1686061	0.960	0.671068	null	hypothetical protein	PA3979	93	92.06
1686063	1.072	0.903808	hemL	glutamate-1-semialdehyde 2,1-aminomutase	PA3977	116	92.34
1686066	1.177	0.99251634	null	probable acyl-CoA dehydrogenase	PA3972	54	94.12
1686069	1.244	0.9354681	null	conserved hypothetical protein	PA3980	68	92.58
1686072	0.781	0.6549346	null	conserved hypothetical protein	PA3983	30	92.88
1686075	1.179	0.65318525	null	hypothetical protein	PA3986	9	95.25
1686078	0.809	0.73755336	null	conserved hypothetical protein	PA3982	197	94.21
1686081	1.042	0.9035161	null	hypothetical protein	PA3988	143	92.38
1686084	0.779	0.5637045	null	conserved hypothetical protein	PA3990	21	94.59
1686085	0.809	0.602844	null	conserved hypothetical protein	PA3985	105	94.7
1686088	1.076	0.9925442	null	conserved hypothetical protein	PA3981	52	92.97
1686091	1.009	2.0703704	null	probable epoxide hydrolase	PA3994	74	96.44
1686094	0.967	0.8740002	leuS	leucyl-tRNA synthetase	PA3987	107	92.73
1686097	1.052	2.8161404	null	conserved hypothetical protein	PA3934	1	92.73
1686100	0.655	0.5500536	holA	DNA polymerase III, delta subunit	PA3989	69	94.31
1686103	1.396	1.1001518	null	hypothetical protein	PA3991	42	93.46
1686106	0.939	0.78252774	null	conserved hypothetical protein	PA3998	79	93.63
1686108	0.978	0.22999625	lnt	apolipoprotein N-acyltransferase	PA3984	204	92.15
1686111	0.759	0.6435975	lis	lipoate synthase	PA3996	80	92.44
1686114	0.926	0.76885384	null	probable transposase	PA3993	45	92.38
1686117	0.975	0.6759941	null	hypothetical protein	PA4000	63	96.92
1686120	0.727	0.58838475	lipB	lipoate-protein ligase B	PA3997	10	94.18
1686123	0.963	0.89408857	dacC	D-ala-D-ala-carboxypeptidase	PA3999	18	92.04
1686126	0.745	0.58197165	sltB1	soluble lytic transglycosylase B	PA4001	10	93.61
1686129	0.947	0.6636152	null	hypothetical protein	PA3992	348	93.29
1686132	1.009	3.5020492	null	probable transcriptional regulator	PA3995	365	92.52
1686135	0.602	0.37804264	rodA	rod shape-determining protein	PA4002	106	92.96
1686138	0.799	0.6847862	nadD	nicotinic acid mononucleotide	PA4006	21	92.76
1686141	0.471	0.68921274	null	hypothetical protein	PA4009	1	92.77
1686144	0.794	0.65703106	null	conserved hypothetical protein	PA4004	38	93.72
1686146	0.979	0.76922524	null	hypothetical protein	PA4010	1	95.89
1686149	0.957	0.70338225	null	conserved hypothetical protein	PA4005	39	92.31
1686151	1.113	0.93091375	amn	AMP nucleosidase	PA3970	266	97.5
1686154	1.229	1.0632225	null	hypothetical protein	PA4014	14	92.53
1686157	0.545	0.29211602	null	hypothetical protein	PA4012	73	94.39
1686160	1.261	1.119601	null	hypothetical protein	PA4011	49	94.06
1686163	1.067	0.88972986	null	conserved hypothetical protein	PA4013	197	97.07
1686166	1.340	0.7847934	null	conserved hypothetical protein	PA4015	12	92.97
1686170	0.773	0.6398586	null	hypothetical protein	PA4018	6	97.6
1686171	1.717	0.9415935	null	probable hydrolase	PA4008	36	92.08
1686174	0.847	0.5240907	null	probable aromatic acid decarboxylase	PA4019	60	95.21
1686177	0.992	0.48855552	null	conserved hypothetical protein	PA4017	194	92.54
1686180	0.788	0.6045287	proA	gamma-glutamyl phosphate reductase	PA4007	408	97.37
1686183	0.644	0.42581972	null	hypothetical protein	PA3910	102	97.15
1686186	1.028	0.6848961	null	probable ethanolamine ammonia-lyase light chain	PA4025	150	97.98
1686189	0.654	0.56708056	pbpA	penicillin-binding protein 2	PA4003	3	94.57
1686192	1.009	4.1239314	eutB	ethanolamine ammonia-lyase large subunit	PA4024	29	92.6
1686195	1.627	1.3623416	null	hypothetical protein	PA4028	75	97.43
1686196	1.181	1.0340723	null	probable acetyltransferase	PA4026	59	92.19
1686199	1.121	1.0599389	ppa	inorganic pyrophosphatase	PA4031	99	92.4
1686202	0.664	0.5340053	null	conserved hypothetical protein	PA4030	248	93.66
1686205	1.009	1.3958225	null	hypothetical protein	PA4033	8	93.31
1686207	0.898	0.6792637	null	conserved hypothetical protein	PA4029	268	94.41
1686210	1.169	0.8280407	aqpZ	aquaporin Z	PA4034	592	95.59
1686213	0.972	0.8389574	null	hypothetical protein	PA4035	1	92.49
1686216	1.384	1.4022534	mpl	UDP-N-acetylmuramate:L-alanyl-gamma-D-glutamyl-	PA4020	205	96.26
1686219	1.009	0.57651514	null	probable ATP-binding component of ABC	PA4037	28	92.61
1686222	0.595	0.35846713	null	hypothetical protein	PA4016	347	94.17
1686225	0.395	0.44168168	null	probable transport protein	PA4023	215	92.83
1686228	1.156	0.9729808	null	hypothetical protein	PA4038	54	92.85
1686231	2.230	1.1706682	null	hypothetical protein	PA4040	41	94.46
1686235	1.196	0.42748052	xseB	exodeoxyribonuclease VII small subunit	PA4042	25	92.7
1686236	0.862	0.6561621	ispA	geranyltranstransferase	PA4043	68	93.4
1686239	0.948	1.5556523	null	hypothetical protein	PA4041	1	93.45
1686242	0.930	0.8614995	dxs	1-deoxyxylulose-5-phosphate synthase	PA4044	199	93.18
1686245	0.714	0.51858294	null	conserved hypothetical protein	PA4045	207	93.99
1686248	0.905	0.6998172	null	hypothetical protein	PA4048	10	92.98
1686251	0.916	0.8035116	null	hypothetical protein	PA4046	79	92.83
1686254	0.903	0.32972616	null	probable aldehyde dehydrogenase	PA4022	1	92.8
1686257	0.613	0.4407043	null	hypothetical protein	PA4049	250	93.74
1686260	0.922	0.77015895	pgpA	phosphatidylglycerophosphatase A	PA4050	41	96.09
1686263	0.718	0.63085604	thiL	thiamine monophosphate kinase	PA4051	663	95.47
1686266	0.792	-0.2589627	null	hypothetical protein	PA4039	137	93.09

1686269	0.979	0.80313194	ribB	GTP cyclohydrolase II / 3,4-dihydroxy-2-butanone	PA4054	116	92.34
1686272	0.790	0.55071646	null	probable two-component response regulator	PA4032	245	92.72
1686275	1.285	1.3395644	ribA	GTP cyclohydrolase II	PA4047	414	95.06
1686278	0.877	0.75618637	ribE	6,7-dimethyl-8-ribityllumazine synthase	PA4053	157	96.63
1686281	0.808	0.3573821	null	probable transcriptional regulator	PA4021	425	94.67
1686284	1.007	0.7607783	ribC	riboflavin synthase alpha chain	PA4055	3	92.4
1686287	0.661	0.36669892	null	conserved hypothetical protein	PA4057	15	92.04
1686290	1.021	0.8515822	nusB	NusB protein	PA4052	137	95.96
1686293	0.974	1.2741477	null	hypothetical protein	PA4062	263	97.83
1686295	0.741	0.5611261	null	hypothetical protein	PA4060	141	94.56
1686298	0.297	-0.1924159	null	probable ATP-binding component of ABC	PA4064	45	93.69
1686301	0.931	0.69815576	null	hypothetical protein	PA4059	148	95.92
1686302	0.816	0.67864716	null	hypothetical protein	PA4063	412	92.4
1686305	0.727	0.55193394	ribD	riboflavin-specific deaminase/reductase	PA4056	504	97.91
1686308	0.990	0.25656956	null	hypothetical protein	PA4065	129	93.18
1686312	0.875	0.75564873	null	hypothetical protein	PA4058	276	97.3
1686314	0.925	0.7382054	null	probable thioredoxin	PA4061	44	92.05
1686317	1.242	1.334594	oprG	Outer membrane protein OprG precursor	PA4067	1	94.13
1686320	0.556	0.3855584	null	hypothetical protein	PA4069	617	95.4
1686323	2.399	1.0734575	null	probable transcriptional regulator	PA4070	104	93
1686326	1.009	1.2161404	null	hypothetical protein	PA4071	122	93.82
1686329	0.600	0.4392484	null	probable epimerase	PA4068	124	93.35
1686332	0.775	0.42573252	null	hypothetical protein	PA4066	378	92.3
1686335	0.985	0.5357481	null	probable transcriptional regulator	PA4077	105	96.23
1686336	0.662	0.45979944	null	hypothetical protein	PA4075	422	96.25
1686339	0.652	1.339275	null	probable response regulator	PA4080	147	92.4
1686342	1.009	1.4004864	cupB6	fimbrial subunit CupB6	PA4081	120	92.25
1686345	1.626	0.69457734	null	probable transcriptional regulator	PA4074	94	94.92
1686348	1.022	0.81681836	null	hypothetical protein	PA4076	240	92.62
1686351	0.968	0.65751684	null	probable dehydrogenase	PA4079	43	94.25
1686354	1.998	0.5347243	cupB1	probable fimbrial subunit CupB1	PA4086	8	93.59
1686357	0.910	0.47521144	cupB4	chaperone CupB4	PA4083	63	92.44
1686360	2.574	2.0185454	cupB2	chaperone CupB2	PA4085	131	94.87
1686363	1.191	0.8934167	cupB5	adhesive protein CupB5	PA4082	170	96.37
1686366	0.883	0.50563604	null	probable aldehyde dehydrogenase	PA4073	32	93.69
1686369	0.826	0.68862474	null	hypothetical protein	PA4090	153	92.03
1686372	1.134	0.8033168	cupB3	usher CupB3	PA4084	213	97.82
1686375	1.226	0.88538516	null	probable two-component sensor	PA4036	314	92
1686379	1.019	0.7191919	hpaC	4-hydroxyphenylacetate 3-monooxygenase small	PA4092	203	92.02
1686381	1.009	6.614174	null	probable aminotransferase	PA4088	193	95.58
1686384	1.009	-2.1772704	null	conserved hypothetical protein	PA4087	181	97.6
1686387	1.274	0.9526528	null	probable short-chain dehydrogenase	PA4089	240	93.36
1686390	4.373	-0.26135135	null	hypothetical protein	PA4093	15	92.71
1686393	0.792	0.5253711	null	probable transcriptional regulator	PA4094	7	95.75
1686396	1.009	1.8017803	null	hypothetical protein	PA4095	126	94.36
1686399	1.182	1.0318733	null	probable major facilitator superfamily (MFS)	PA4096	42	92.24
1686402	1.009	1.3733333	null	probable short-chain dehydrogenase	PA4098	152	92.62
1686405	0.726	0.5694084	hpaA	4-hydroxyphenylacetate 3-monooxygenase large	PA4091	2	93.39
1686408	1.515	1.0565804	null	probable two-component response regulator	PA4101	119	94.39
1686411	1.077	0.5449859	null	hypothetical protein	PA4099	131	93.02
1686414	1.141	1.1183841	null	hypothetical protein	PA4103	256	92.74
1686417	1.378	1.19015	null	probable alcohol dehydrogenase (Zn-dependent)	PA4097	112	94.87
1686420	1.009	1.8762964	null	hypothetical protein	PA4105	303	94.56
1686423	1.069	1.4710715	null	probable two-component sensor	PA4102	138	93.03
1686426	1.297	0.2897818	null	conserved hypothetical protein	PA4106	153	92.24
1686429	0.170	0.4110526	null	hypothetical protein	PA4107	239	93.98
1686432	0.676	0.3422913	null	probable dehydrogenase	PA4100	1	93.27
1686435	4.192	2.6233335	ampC	beta-lactamase precursor	PA4110	112	92.59
1686438	0.410	1.722151	ampR	transcriptional regulator AmpR	PA4109	246	93.6
1686442	1.061	0.6594751	null	hypothetical protein	PA4111	166	94.59
1686444	1.009	0.7662447	null	conserved hypothetical protein	PA4104	96	92.53
1686447	1.214	0.7737522	null	hypothetical protein	PA4108	10	92.17
1686450	0.884	0.6783269	null	spermidine acetyltransferase	PA4114	67	92.85
1686453	0.852	0.7373766	null	conserved hypothetical protein	PA4115	3	92.1
1686456	1.009	-0.20706627	aph	aminoglycoside 3'-phosphotransferase type IIb	PA4119	66	97.87
1686459	1.428	1.1209042	null	conserved hypothetical protein	PA4121	78	97.75
1686462	1.029	1.1427276	null	hypothetical protein	PA4116	370	94.6
1686465	0.986	0.41403723	null	probable transcriptional regulator	PA4120	1	94.12
1686468	0.679	0.5180578	null	hypothetical protein	PA4118	145	93.71
1686471	1.009	1.0458823	null	probable major facilitator superfamily (MFS)	PA4113	1	96.2
1686474	0.880	0.45833334	hpcB	homoprotocatechuate 2,3-dioxygenase	PA4124	1	92.62
1686477	1.065	0.5339103	null	probable Resistance-Nodulation-Cell Division	PA3677	1016	97.9
1686480	1.009	0.89229316	null	conserved hypothetical protein	PA4122	400	92.05
1686484	1.163	-0.2274499	hpcD	5-carboxymethyl-2-hydroxymuconate isomerase	PA4125	167	93.62
1686486	1.364	1.2855841	hpcG	2-oxo-hept-3-ene-1,7-dioate hydratase	PA4127	49	93.34
1686489	0.071	-0.25792816	null	hypothetical protein	PA4129	334	94.79
1686492	1.173	-0.11680671	hpcC	5-carboxy-2-hydroxymuconate semialdehyde	PA4123	22	92
1686495	1.009	2.0698538	null	probable iron-sulfur protein	PA4131	5	94.72
1686498	1.040	0.910024	null	conserved hypothetical protein	PA4132	168	97.09
1686501	0.649	0.48908424	null	hypothetical protein	PA4134	87	95.56
1686502	0.943	0.70100605	null	probable transcriptional regulator	PA4135	1	93.6
1686505	0.692	0.5428864	null	probable bacteriophytochrome	PA4117	395	94.37
1686508	1.339	1.2537302	null	probable major facilitator superfamily (MFS)	PA4126	381	92.28
1686511	1.009	0.7964905	null	probable major facilitator superfamily (MFS)	PA4136	1	93.45
1686514	1.240	1.1090826	null	probable sensor/response regulator hybrid	PA4112	163	94.85
1686517	1.350	0.9325779	null	hypothetical protein	PA4139	36	92.82
1686519	1.093	3.8273807	null	probable porin	PA4137	583	97.23
1686522	1.133	0.9770645	null	hypothetical protein	PA4141	197	93.49
1686525	1.420	1.0010325	null	hypothetical protein	PA4140	316	97.89
1686528	1.175	1.1549658	null	probable secretion protein	PA4142	53	92.33
1686531	1.149	0.834657	null	probable transcriptional regulator	PA4145	201	92.29
1686534	2.628	0.8425926	null	hypothetical protein	PA4146	175	95.25
1686537	1.009	1.3981268	tyrS	tyrosyl-tRNA synthetase	PA4138	1181	95.05
1686540	1.276	0.90423506	acoR	transcriptional regulator AcoR	PA4147	7	92.18
1686543	1.009	1.134513	null	probable toxin transporter	PA4143	365	97.01
1686546	1.260	1.0715601	null	probable short-chain dehydrogenase	PA4148	1	92.46
1686549	0.418	0.36815283	null	cytochrome c oxidase subunit (cbb3-type)	PA4133	76	94.04
1686552	1.009	1.894705	acoB	acetoin catabolism protein AcoB	PA4151	59	95.15
1686555	0.062	-0.06667169	null	probable sulfite or nitrite reductase	PA4130	239	92.95
1686558	1.009	0.49285713	null	probable dehydrogenase E1 component	PA4150	758	93.62
1686561	1.064	0.59200877	null	probable outer membrane protein precursor	PA4144	381	95.29
1686564	1.048	1.0414432	null	conserved hypothetical protein	PA4154	3	94.31
1686567	1.023	0.7558087	null	conserved hypothetical protein	PA4149	917	97.21
1686570	1.062	1.1299113	null	2,3-butanediol dehydrogenase	PA4153	144	94.67
1686573	1.009	2.13622	null	hypothetical protein	PA4155	69	94.41
1686576	1.554	1.5109489	fepC	ferric enterobactin transport protein FepC	PA4158	54	92.52
1686579	1.009	-0.3723077	null	probable TonB-dependent receptor	PA4156	243	92.61
1686582	1.116	0.8022536	null	probable short-chain dehydrogenase	PA4162	104	95.24
1686585	1.022	0.70962626	null	probable transcriptional regulator	PA4157	632	93.18

1686588	1.076	1.6349123	fepB	ferrienterobactin-binding periplasmic protein	PA4159	660	93.57
1686592	0.477	0.20393434	null	hypothetical protein	PA4164	100	97.31
1686594	2.118	-1.9440291	null	probable acetyltransferase	PA4166	1	92.9
1686597	1.009	-3.8667583	null	probable hydrolase	PA4152	66	93.38
1686600	0.362	-0.36305317	null	probable oxidoreductase	PA4167	253	93.35
1686603	1.108	0.86472905	null	conserved hypothetical protein	PA4169	8	92.6
1686606	0.484	-0.43596405	null	hypothetical protein	PA4170	41	92.5
1686609	1.287	0.8921298	fepD	ferric enterobactin transport protein FepD	PA4160	127	94.86
1686612	1.060	0.54519355	null	hypothetical protein	PA4163	219	93.28
1686615	1.310	1.1284852	fepG	ferric enterobactin transport protein FepG	PA4161	977	95.03
1686618	1.085	0.94603026	null	hypothetical protein	PA4027	183	95.3
1686621	1.302	0.97141427	null	probable transcriptional regulator	PA4165	5	94.79
1686624	3.310	0.504616	null	probable nuclease	PA4172	6	92.42
1686627	0.494	-0.107345484	null	conserved hypothetical protein	PA4173	196	94.85
1686629	1.472	0.90090907	null	hypothetical protein	PA4178	77	95.27
1686632	1.035	-0.2666666	null	probable protease	PA4171	289	95.18
1686635	0.768	0.09023235	null	probable transcriptional regulator	PA4174	338	92.83
1686638	1.009	1.7755411	null	hypothetical protein	PA4177	317	94.74
1686641	1.174	0.8882616	prpL	Pvds-regulated endoprotease, lysyl class	PA4175	9	92.23
1686644	1.231	1.1810024	fpvB	second ferric pyoverdine receptor FpvB	PA4168	16	94.61
1686647	1.007	0.013712073	null	hypothetical protein	PA4182	114	96.51
1686650	1.136	0.8377315	null	probable acetolactate synthase large subunit	PA4180	4	93.1
1686653	1.015	0.8071621	null	hypothetical protein	PA4183	31	94.83
1686656	0.540	0.30477673	null	hypothetical protein	PA4181	559	94.25
1686659	0.722	0.15980436	null	probable transcriptional regulator	PA4185	432	92.54
1686662	0.823	1.248046	null	hypothetical protein	PA4186	1	93.57
1686665	0.947	0.8290806	null	probable transcriptional regulator	PA4184	91	93.31
1686668	0.909	2.8578496	null	probable major facilitator superfamily (MFS)	PA4187	10	92.44
1686671	0.600	-4.821018	null	probable ATP-binding component of ABC	PA4192	5	93.01
1686674	0.233	-0.2958421	null	probable iron/ascorbate oxidoreductase	PA4191	175	94.9
1686677	1.009	0.9257143	null	conserved hypothetical protein	PA4188	76	92.79
1686680	0.890	0.6164452	null	probable permease of ABC transporter	PA4193	566	94.3
1686683	0.988	0.5006582	pqsL	probable FAD-dependent monooxygenase	PA4190	214	94.16
1686686	0.526	0.41875586	null	probable binding protein component of ABC	PA4195	222	94.02
1686689	0.260	0.15804696	null	probable permease of ABC transporter	PA4194	166	92.27
1686692	1.125	0.023435425	null	probable porin	PA4179	409	93.26
1686696	0.695	0.44371885	null	probable two-component response regulator	PA4196	495	92.84
1686698	0.631	0.40988836	null	hypothetical protein	PA4200	1	92.54
1686701	1.200	0.93024415	null	probable two-component sensor	PA4197	591	97.86
1686704	1.239	0.9758352	null	probable transcriptional regulator	PA4203	1	93.36
1686707	0.927	1.251508	null	conserved hypothetical protein	PA4204	162	96.14
1686710	0.997	0.74870574	ddlA	D-alanine-D-alanine ligase A	PA4201	701	94.32
1686713	1.009	1.6361907	null	conserved hypothetical protein	PA4128	399	94.42
1686716	1.396	0.96826756	null	probable acyl-CoA dehydrogenase	PA4199	318	92.45
1686719	1.009	0.3803143	mexH	probable Resistance-Nodulation-Cell Division	PA4206	239	96.45
1686722	1.133	0.76836306	null	hypothetical protein	PA4202	18	94.94
1686725	0.948	0.8122549	phzM	probable phenazine-specific methyltransferase	PA4209	45	92.82
1686730	1.173	0.7113997	phzA1	probable phenazine biosynthesis protein	PA4210	21	94.9
1686731	1.348	0.9483398	mexG	hypothetical protein	PA4205	261	92.3
1686734	0.803	1.481555	phzB1	probable phenazine biosynthesis protein	PA4211	126	93.25
1686736	1.349	1.0851419	phzD1	phenazine biosynthesis protein PhzD	PA4213	456	93.73
1686739	1.009	2.4575965	null	probable aldehyde dehydrogenase	PA4189	387	92.47
1686744	1.078	0.8090909	phzG1	probable pyridoxamine 5'-phosphate oxidase	PA4216	595	94.29
1686745	1.241	0.6932963	phzC1	phenazine biosynthesis protein PhzC	PA4212	196	94.7
1686749	1.078	-0.24285714	phzF1	probable phenazine biosynthesis protein	PA4215	247	94.38
1686751	2.510	1.8055226	null	hypothetical protein	PA4219	433	94.6
1686754	1.061	0.77301586	null	probable transporter	PA4218	171	92.57
1686758	1.039	0.9494233	null	hypothetical protein	PA4220	223	95.34
1686759	1.055	0.44738334	phzS	flavin-containing monooxygenase	PA4217	682	94.53
1686762	1.009	4.738011	fptA	Fe(III)-pyochelin outer membrane receptor	PA4221	129	92.66
1686765	0.696	0.102099575	null	probable AMP-binding enzyme	PA4198	640	94.18
1686768	1.392	1.1445181	null	probable ATP-binding component of ABC	PA4222	638	97.97
1686771	1.312	0.8007692	pchR	transcriptional regulator PchR	PA4227	56	95.81
1686774	0.725	0.463986	pchG	pyochelin biosynthetic protein PchG	PA4224	386	97.72
1686779	1.009	2.2236264	phzE1	phenazine biosynthesis protein PhzE	PA4214	809	94.68
1686780	1.027	0.9930951	pchD	pyochelin biosynthesis protein PchD	PA4228	324	97.76
1686783	0.860	0.62822115	mexI	probable Resistance-Nodulation-Cell Division	PA4207	94	93.7
1686786	1.009	0.9794437	pchB	salicylate biosynthesis protein PchB	PA4230	222	92.48
1686788	1.545	1.0063831	pchF	pyochelin synthetase	PA4225	553	93.42
1686791	1.036	0.8970666	ssb	single-stranded DNA-binding protein	PA4232	27	93.19
1686794	1.173	5.3377266	pchA	salicylate biosynthesis isochorismate synthase	PA4231	116	93.44
1686797	1.115	0.79796755	pchC	pyochelin biosynthetic protein PchC	PA4229	328	97.99
1686800	1.012	0.71864456	uvrA	excinuclease ABC subunit A	PA4234	11	92.24
1686803	1.321	1.3326762	katA	catalase	PA4236	246	92.2
1686806	1.062	1.0536813	rpsD	30S ribosomal protein S4	PA4239	26	92.47
1686809	1.035	1.0741899	rpsK	30S ribosomal protein S11	PA4240	84	92.52
1686811	1.017	0.9896116	rpoA	DNA-directed RNA polymerase alpha chain	PA4238	31	94.38
1686814	1.018	1.0574586	rpsM	30S ribosomal protein S13	PA4241	104	94.07
1686817	1.213	1.4151319	bfrA	bacterioferritin	PA4235	370	92.53
1686818	0.955	1.0119997	rpmD	50S ribosomal protein L30	PA4245	41	94.44
1686820	1.046	1.0160487	rplO	50S ribosomal protein L15	PA4244	35	96.91
1686823	1.628	1.0260141	null	probable ATP-binding component of ABC	PA4223	353	95.86
1686826	1.000	1.0934566	rplF	50S ribosomal protein L6	PA4248	174	94.69
1686829	1.002	0.64049053	null	probable major facilitator superfamily (MFS)	PA4233	3	92.58
1686832	1.072	1.1428965	rplR	50S ribosomal protein L18	PA4247	87	92.7
1686835	1.079	1.1235764	rpsE	30S ribosomal protein S5	PA4246	80	92.73
1686838	0.699	0.75361836	rpsN	30S ribosomal protein S14	PA4250	97	92.65
1686840	0.918	1.0945628	rplX	50S ribosomal protein L24	PA4252	48	92.8
1686843	0.944	0.9605589	rpsH	30S ribosomal protein S8	PA4249	270	92.73
1686844	0.709	0.6674834	secY	secretion protein SecY	PA4243	100	95.18
1686847	0.971	0.99317205	rplN	50S ribosomal protein L14	PA4253	104	93.22
1686850	0.900	1.0744189	rplE	50S ribosomal protein L5	PA4251	282	92.38
1686852	1.003	1.1514254	rpsQ	30S ribosomal protein S17	PA4254	67	92.22
1686854	0.784	0.8175991	rplV	50S ribosomal protein L22	PA4258	25	95.77
1686857	0.962	1.11721	rplP	50S ribosomal protein L16	PA4256	39	92.03
1686860	0.970	1.0670583	rpsC	30S ribosomal protein S3	PA4257	31	92.06
1686863	0.865	0.9922911	rpsS	30S ribosomal protein S19	PA4259	81	92.34
1686865	1.037	1.1774222	rplW	50S ribosomal protein L23	PA4261	23	93.38
1686868	1.036	1.2227852	rplB	50S ribosomal protein L2	PA4260	51	92.08
1686871	0.940	0.9859241	rplD	50S ribosomal protein L4	PA4262	15	92
1686874	1.089	1.1067013	rplC	50S ribosomal protein L3	PA4263	117	92.71
1686877	1.095	1.1688166	rpsJ	30S ribosomal protein S10	PA4264	82	93.28
1686878	1.080	1.1603416	rpsL	30S ribosomal protein S12	PA4268	61	95.63
1686881	1.205	1.4103261	rpsG	30S ribosomal protein S7	PA4267	208	95.3
1686884	0.656	0.71263766	rplL	50S ribosomal protein L7 / L12	PA4271	144	93.61
1686889	0.910	0.9831549	tufA	elongation factor Tu	PA4265	249	92.37
1686890	1.016	1.2276934	rplJ	50S ribosomal protein L10	PA4272	270	92.1
1686893	0.886	0.8947714	rplK	50S ribosomal protein L11	PA4274	64	97.32
1686896	0.998	0.97590196	rplA	50S ribosomal protein L1	PA4273	107	92.29

1686899	0.836	0.8255827	secE	secretion protein SecE	PA4276	180	93.08
1686900	0.754	0.6884874	rpoB	DNA-directed RNA polymerase beta chain	PA4270	84	93.24
1686903	0.696	0.665974	nusG	transcription antitermination protein NusG	PA4275	124	92.79
1686906	0.596	0.25941738	birA	BirA bifunctional protein	PA4280	58	93.02
1686909	0.904	0.74065626	null	hypothetical protein	PA4279	65	97.08
1686912	1.103	1.0858033	fusA1	elongation factor G	PA4266	183	92.51
1686915	0.778	0.37651393	null	hypothetical protein	PA4278	27	92.11
1686918	0.795	0.73488677	rpoC	DNA-directed RNA polymerase beta* chain	PA4269	65	92.76
1686921	0.851	0.44161385	sbcD	exonuclease SbcD	PA4281	111	93.7
1686924	1.202	0.9601529	recD	exodeoxyribonuclease V alpha chain	PA4283	456	93.13
1686927	1.741	1.8836868	null	probable nonribosomal peptide synthetase	PA4078	21	93.64
1686932	0.889	0.9584147	tufB	elongation factor Tu	PA4277	249	92.37
1686933	1.175	1.0252268	recB	exodeoxyribonuclease V beta chain	PA4284	27	95.5
1686936	0.726	0.558956	null	hypothetical protein	PA4286	77	97.63
1686939	0.796	0.5364816	null	hypothetical protein	PA4291	29	97.04
1686942	1.009	0.57566637	null	hypothetical protein	PA4287	413	93.75
1686945	1.391	0.8268981	null	probable transcriptional regulator	PA4288	21	92.1
1686948	1.171	0.5172428	null	probable exonuclease	PA4282	36	94.47
1686951	0.812	0.70838803	null	probable chemotaxis transducer	PA4290	1	93.93
1686954	0.616	0.24147263	null	probable two-component sensor	PA4293	36	95.8
1686957	1.075	1.0916793	null	hypothetical protein	PA4294	354	94.07
1686960	1.359	0.38005623	null	hypothetical protein	PA4295	258	93.53
1686961	1.516	1.0480998	null	hypothetical protein	PA4297	149	97.67
1686964	3.074	1.1866667	null	probable two-component response regulator	PA4296	79	92.17
1686967	1.081	0.80999184	recC	exodeoxyribonuclease V gamma chain	PA4285	16	92.68
1686970	1.009	1.5499507	pchE	dihydroaeruginoic acid synthetase	PA4226	291	94.68
1686973	1.009	-0.36777782	null	hypothetical protein	PA4298	225	92.78
1686975	1.150	0.9102613	null	probable phosphate transporter	PA4292	5	92.42
1686978	1.232	0.7934343	null	hypothetical protein	PA4299	169	96.84
1686981	1.009	1.1957049	null	hypothetical protein	PA4301	530	92.12
1686984	1.009	-2.5159771	null	hypothetical protein	PA4306	28	93.63
1686986	1.412	4.8823767	opmD	probable outer membrane protein precursor	PA4208	1342	93.31
1686989	1.322	0.9584387	null	hypothetical protein	PA4300	4	95.47
1686992	1.009	0.8571429	null	probable transporter	PA4289	530	94.5
1686995	1.285	0.9297066	pctC	chemotactic transducer PctC	PA4307	113	93.44
1686998	0.952	0.6129254	null	probable amino acid permease	PA4072	282	94.82
1687001	1.588	1.7798945	null	probable type II secretion system protein	PA4302	143	93.26
1687004	1.009	4.5199857	null	hypothetical protein	PA4305	446	92.68
1687007	1.244	0.8816401	null	conserved hypothetical protein	PA4312	101	92.83
1687010	1.009	5.2914286	null	hypothetical protein	PA4303	29	94.76
1687013	1.068	0.90064096	purU1	formyltetrahydrofolate deformylase	PA4314	34	92.61
1687016	0.712	0.0775641	null	conserved hypothetical protein	PA4311	61	92.59
1687019	1.254	1.3966142	mvaT	transcriptional regulator MvaT, P16 subunit	PA4315	140	92.06
1687021	0.972	0.8563124	sbcB	exodeoxyribonuclease I	PA4316	117	92.09
1687024	1.340	1.0090082	null	probable type II secretion system protein	PA4304	1	96.73
1687027	0.695	0.55146486	null	conserved hypothetical protein	PA4319	34	93.59
1687030	0.930	1.0846442	null	conserved hypothetical protein	PA4308	105	93.64
1687033	1.285	1.0301632	pctB	chemotactic transducer PctB	PA4310	404	95.76
1687036	0.638	0.5523494	null	hypothetical protein	PA4317	96	93.85
1687039	1.277	0.9573615	null	hypothetical protein	PA4324	219	93.63
1687041	0.658	0.54674655	null	hypothetical protein	PA4318	1	94.75
1687044	0.629	0.52610403	null	hypothetical protein	PA4321	601	93.3
1687047	1.093	0.9446747	null	hypothetical protein	PA4325	179	93.36
1687050	1.425	1.2413086	null	hypothetical protein	PA4313	646	92.58
1687053	1.429	0.95550096	null	hypothetical protein	PA4326	154	96.12
1687056	0.755	0.4898788	null	probable enoyl-CoA hydratase/isomerase	PA4330	191	94.04
1687059	0.891	0.9573957	rplQ	50S ribosomal protein L17	PA4237	109	96.47
1687062	1.032	0.77887505	ppiC2	peptidyl-prolyl cis-trans isomerase C2	PA4176	14	92.64
1687065	1.020	0.8512039	pykA	pyruvate kinase II	PA4329	204	93.15
1687068	1.444	1.0812364	null	hypothetical protein	PA4328	141	95.6
1687071	0.792	0.117078856	null	hypothetical protein	PA4327	213	92.12
1687074	0.661	0.50747114	null	probable ferredoxin reductase	PA4331	228	94.69
1687077	0.660	0.05078966	null	hypothetical protein	PA4323	229	93.66
1687080	0.539	0.17037442	null	conserved hypothetical protein	PA4322	310	95.49
1687083	1.103	0.8114335	null	conserved hypothetical protein	PA4336	182	93.54
1687086	1.079	0.8340346	null	probable fumarase	PA4333	125	95.85
1687089	0.795	-0.040509123	null	hypothetical protein	PA4337	240	92.6
1687092	0.756	0.35879096	null	hypothetical protein	PA4332	50	92.37
1687095	0.655	0.4924496	null	hypothetical protein	PA4320	202	92.38
1687098	1.118	-0.42627087	null	hypothetical protein	PA4346	134	94.59
1687100	1.537	1.271031	null	probable major facilitator superfamily (MFS)	PA4343	113	93.92
1687103	1.661	0.7668558	null	probable amidase	PA4342	355	93.47
1687106	1.192	1.0338233	null	conserved hypothetical protein	PA4348	27	92.61
1687109	1.668	2.3761122	null	probable transcriptional regulator	PA4341	83	92.77
1687112	0.755	0.64502686	null	hypothetical protein	PA4335	25	92.2
1687114	0.759	0.03371377	null	probable hydrolase	PA4344	68	95.28
1687117	1.111	1.4489768	null	hypothetical protein	PA4345	69	92.42
1687120	0.948	0.7256073	null	hypothetical protein	PA4347	284	96.52
1687123	1.011	0.62728536	null	hypothetical protein	PA4340	105	92.65
1687126	0.885	0.6324431	null	hypothetical protein	PA4349	279	93.12
1687129	1.369	1.0599698	null	conserved hypothetical protein	PA4350	328	96.47
1687132	0.926	0.49834102	null	hypothetical protein	PA4338	229	92.29
1687135	1.009	0.0883041	null	conserved hypothetical protein	PA4357	25	97.58
1687136	1.262	1.3132315	null	conserved hypothetical protein	PA4354	9	92.12
1687139	1.295	1.066863	null	conserved hypothetical protein	PA4359	48	94.25
1687141	0.729	0.41192466	null	hypothetical protein	PA4360	10	92.1
1687144	1.202	1.0257105	null	conserved hypothetical protein	PA4353	518	93.77
1687147	1.999	1.1107336	iciA	inhibitor of chromosome initiation IciA	PA4363	507	97.82
1687150	1.183	0.9055072	null	hypothetical protein	PA4362	214	95.33
1687153	1.207	0.83930796	null	hypothetical protein	PA4364	30	92.6
1687156	1.200	0.81377774	null	probable oxidoreductase	PA4361	268	96.2
1687161	1.074	-2.376891	null	probable transporter	PA4365	378	92.51
1687162	0.948	-4.4828844	null	probable acyltransferase	PA4351	211	92.42
1687165	11.000	-1.6806812	null	probable major facilitator superfamily (MFS)	PA4355	563	95.2
1687168	0.653	0.4911502	null	probable phospholipase	PA4339	48	92.56
1687171	1.127	1.3319683	sodB	superoxide dismutase	PA4366	17	92.51
1687174	6.119	4.979906	xenB	xenobiotic reductase	PA4356	695	92.38
1687177	1.911	0.7475601	null	probable ferrous iron transport protein	PA4358	292	93.95
1687180	1.226	0.84922373	null	hypothetical protein	PA4368	133	95.46
1687183	1.348	0.49541354	null	hypothetical protein	PA4369	104	95.06
1687186	1.146	1.1056937	null	hypothetical protein	PA4371	1	93.51
1687189	0.566	-1.3426014	null	hypothetical protein	PA4377	88	93.47
1687190	0.921	0.6222826	inaA	InaA protein	PA4378	151	93.31
1687193	1.051	0.6109697	null	conserved hypothetical protein	PA4379	87	96.97
1687196	0.938	0.7192834	null	hypothetical protein	PA4373	237	95.53
1687199	1.002	0.93627167	icmP	Insulin-cleaving metalloproteinase outer	PA4370	303	94.07
1687202	1.075	0.7904457	null	probable two-component response regulator	PA4381	57	94.58
1687205	0.866	0.74391204	null	probable Resistance-Nodulation-Cell Division	PA4374	57	92.4
1687208	1.837	1.497039	pctA	chemotactic transducer PctA	PA4309	146	92.89

1687211	0.732	0.50694525	null	probable Resistance-Nodulation-Cell Division	PA4375	73	93.11
1687214	0.929	0.7528337	null	conserved hypothetical protein	PA4383	43	96.27
1687217	1.009	0.26	null	hypothetical protein	PA4382	152	92.11
1687220	1.142	1.1871713	null	hypothetical protein	PA4384	102	97.8
1687223	0.743	0.5546282	null	probable two-component sensor	PA4380	1	92.02
1687226	1.058	0.91625535	null	conserved hypothetical protein	PA4387	3	92.88
1687229	0.928	0.74983853	null	hypothetical protein	PA4390	14	92.79
1687232	1.042	0.6734734	null	hypothetical protein	PA4388	45	92.7
1687235	2.768	2.8772728	null	conserved hypothetical protein	PA4392	12	92.55
1687237	1.281	1.3967946	groEL	GroEL protein	PA4385	95	94.91
1687240	1.274	1.005894	speA	arginine decarboxylase (ADC)	PA4389	94	93.11
1687243	3.183	0.7018425	null	hypothetical protein	PA4391	2	92.15
1687246	0.816	0.6101342	null	hypothetical protein	PA4372	283	95.03
1687249	1.114	0.96346414	null	conserved hypothetical protein	PA4395	204	92.48
1687252	1.480	1.4761802	groES	GroES protein	PA4386	92	94.8
1687255	0.378	0.20089726	null	probable pyrophosphohydrolase	PA4400	1	92.5
1687258	0.792	0.6598835	null	probable two-component response regulator	PA4396	97	94.61
1687261	0.866	-0.19638494	panE	ketopantoate reductase	PA4397	491	95.46
1687264	1.028	0.7479038	null	conserved hypothetical protein	PA4367	182	94.2
1687267	1.069	0.8060312	argJ	glutamate N-acetyltransferase	PA4402	22	95.41
1687270	0.786	0.62883395	null	probable permease	PA4393	174	94.81
1687273	0.939	0.91541255	lpxC	UDP-3-O-acyl-N-acetylglucosamine deacetylase	PA4406	17	92.05
1687276	0.955	0.6960817	null	hypothetical protein	PA4405	39	95.9
1687277	1.182	1.0457413	ftsZ	cell division protein FtsZ	PA4407	146	96.33
1687280	1.000	0.861551	ftsA	cell division protein FtsA	PA4408	86	93.41
1687283	0.889	0.76612294	null	probable glutathione S-transferase	PA4401	550	92.86
1687286	0.863	0.6684371	pncB2	nicotinate phosphoribosyltransferase	PA4376	561	94.47
1687289	1.252	0.8607578	null	hypothetical protein	PA4404	35	97.26
1687292	1.064	0.9233135	ddlB	D-alanine--D-alanine ligase	PA4410	5	92.7
1687295	0.880	0.7650187	ftsQ	cell division protein FtsQ	PA4409	109	92.04
1687298	0.822	0.37649086	null	conserved hypothetical protein	PA4399	250	95.22
1687301	0.932	0.75474775	murC	UDP-N-acetylmuramate--alanine ligase	PA4411	521	96.09
1687304	0.889	0.7737644	mraY	phospho-N-acetylmuramoyl-pentapeptide-	PA4415	106	93.32
1687307	0.739	0.6087858	secA	secretion protein SecA	PA4403	63	94.1
1687310	0.858	0.60394436	ftsL	cell division protein FtsL	PA4419	78	93.99
1687313	0.963	0.94674253	null	conserved hypothetical protein	PA4421	53	94.39
1687315	0.896	0.72349954	murD	UDP-N-acetylmuramoylalanine--D-glutamate ligase	PA4414	10	94.59
1687318	0.703	0.6081451	null	conserved hypothetical protein	PA4420	52	95.47
1687321	1.042	-0.9015177	null	conserved hypothetical protein	PA4422	10	92.07
1687324	0.837	0.7025109	ftsI	penicillin-binding protein 3	PA4418	98	92.02
1687327	0.915	0.79468024	murF	UDP-N-acetylmuramoylalanyl-D-glutamyl-2,	PA4416	393	92.44
1687330	0.766	0.6326705	null	conserved hypothetical protein	PA4424	95	92.02
1687333	1.136	1.071986	null	probable phosphoheptose isomerase	PA4425	2	92.79
1687336	1.250	1.0162604	null	conserved hypothetical protein	PA4394	406	93.66
1687339	1.403	0.6111895	null	probable two-component sensor	PA4398	375	92.86
1687342	0.937	0.8157212	sspA	stringent starvation protein A	PA4428	29	92.62
1687345	0.964	0.9241349	null	conserved hypothetical protein	PA4426	187	92.46
1687348	1.092	0.94359493	null	probable cytochrome c1 precursor	PA4429	70	92.12
1687351	0.800	0.6479683	sspB	stringent starvation protein B	PA4427	89	95.02
1687354	1.027	1.0125418	null	probable iron-sulfur protein	PA4431	77	94.73
1687357	0.932	1.0164466	rpsI	30S ribosomal protein S9	PA4432	42	92.43
1687360	1.004	1.1323012	rplM	50S ribosomal protein L13	PA4433	104	93.05
1687363	1.065	0.87807405	null	probable oxidoreductase	PA4434	21	94.92
1687366	1.165	1.0119097	null	probable transcriptional regulator	PA4436	193	95.12
1687369	3.678	1.1648238	null	hypothetical protein	PA4437	47	93.96
1687372	1.202	0.9923174	null	conserved hypothetical protein	PA4438	16	92.75
1687375	0.965	0.9177146	null	probable cytochrome b	PA4430	181	92.66
1687378	0.992	1.1205435	null	probable acyl-CoA dehydrogenase	PA4435	16	92.16
1687381	0.916	0.7036211	null	hypothetical protein	PA4440	1	92.36
1687384	0.905	0.7909128	trpS	tryptophanyl-tRNA synthetase	PA4439	4	92.19
1687387	0.948	0.7637011	null	conserved hypothetical protein	PA4423	1	93.68
1687390	1.092	0.801554	mltB1	soluble and membrane-bound lytic	PA4444	19	94.25
1687393	0.599	0.43191782	cysN	ATP sulfurylase GTP-binding subunit/APS kinase	PA4442	159	92.98
1687396	0.545	0.513264	murE	UDP-N-acetylmuramoylalanyl-D-glutamate-2,	PA4417	602	93.07
1687399	0.954	0.4908095	algW	AlgW protein	PA4446	141	94.79
1687402	0.805	0.7354381	hisG	ATP-phosphoribosyltransferase	PA4449	2	92.25
1687405	1.168	0.9681837	null	hypothetical protein	PA4441	123	92.87
1687408	0.986	-1.7806183	null	conserved hypothetical protein	PA4445	11	93.28
1687411	0.729	0.66205364	null	conserved hypothetical protein	PA4451	177	94.36
1687412	0.826	0.72881943	ftsW	cell division protein FtsW	PA4413	654	96.06
1687415	0.858	0.7434862	hisC1	histidinol-phosphate aminotransferase	PA4447	10	93.1
1687418	0.749	0.65698826	null	conserved hypothetical protein	PA4452	223	94.94
1687419	1.239	1.2111307	null	conserved hypothetical protein	PA4453	93	92.27
1687422	0.817	0.73460925	murA	UDP-N-acetylglucosamine	PA4450	95	95.92
1687425	0.738	0.61230624	hisD	histidinol dehydrogenase	PA4448	1	92.03
1687428	0.847	0.73181856	null	conserved hypothetical protein	PA4458	239	92.22
1687431	1.688	1.4690573	null	conserved hypothetical protein	PA4454	123	92.34
1687434	0.946	0.78608656	null	probable ATP-binding component of ABC	PA4456	1	92.07
1687437	0.897	0.8059702	null	probable permease of ABC transporter	PA4455	540	92.36
1687440	0.979	0.85487807	null	conserved hypothetical protein	PA4459	55	92.52
1687443	0.594	0.51382446	cysD	ATP sulfurylase small subunit	PA4443	83	92.5
1687446	0.965	1.0745789	null	conserved hypothetical protein	PA4463	45	94.27
1687448	1.196	1.0076232	null	conserved hypothetical protein	PA4457	357	94.28
1687451	1.146	1.021972	null	conserved hypothetical protein	PA4460	196	92.44
1687454	0.737	0.6647135	null	probable phosphoryl carrier protein	PA4466	2	93.33
1687457	0.838	0.7327801	rpoN	RNA polymerase sigma-54 factor	PA4462	6	92.3
1687460	1.209	0.37206286	null	hypothetical protein	PA4471	10	94.23
1687463	1.203	1.144352	ptsN	nitrogen regulatory IIA protein	PA4464	113	93.74
1687466	0.870	0.8776876	null	conserved hypothetical protein	PA4465	71	92.89
1687469	1.009	0.31695235	sodM	superoxide dismutase	PA4468	38	92.85
1687472	1.281	0.98179644	pmbA	PmbA protein	PA4472	1	94.19
1687475	0.916	0.64163846	null	conserved hypothetical protein	PA4475	213	94.43
1687478	1.009	1.1614516	null	hypothetical protein	PA4469	305	92.62
1687481	0.973	0.86705524	null	probable ATP-binding component of ABC	PA4461	173	94.84
1687484	1.371	0.9972679	null	hypothetical protein	PA4473	23	92.71
1687487	0.815	0.7333816	null	conserved hypothetical protein	PA4478	1	96.95
1687490	1.029	0.7101339	null	conserved hypothetical protein	PA4474	162	92.29
1687493	0.676	0.45558128	cafA	cytoplasmic axial filament protein	PA4477	54	92.99
1687496	1.105	0.954842	gatC	Glu-tRNA(Gln) amidotransferase subunit C	PA4482	66	93.21
1687497	1.169	0.9168576	mreB	rod shape-determining protein MreB	PA4481	82	94.85
1687500	1.903	0.50648606	null	conserved hypothetical protein	PA4485	151	97.04
1687501	1.161	2.9653847	null	hypothetical protein	PA4467	521	95.61
1687504	0.800	0.639861	murG	UDP-N-acetylglucosamine--N-acetylmuramyl-	PA4412	566	95.8
1687507	1.286	1.1913569	fumC1	fumarate hydratase	PA4470	162	97.23
1687510	0.796	0.6971577	mreD	rod shape-determining protein MreD	PA4479	241	92.26
1687513	1.149	0.95138025	gatB	Glu-tRNA(Gln) amidotransferase subunit B	PA4484	263	92.63
1687516	0.945	0.5000129	null	conserved hypothetical protein	PA4487	104	94.99
1687519	0.266	-1.7983911	null	conserved hypothetical protein	PA4486	83	92.22
1687522	1.031	0.9057754	mreC	rod shape-determining protein MreC	PA4480	36	94.19

1687525	0.826	0.7882277	null	conserved hypothetical protein	PA4492	27	95.38
1687528	1.000	0.76665336	null	hypothetical protein	PA4476	1	94.29
1687531	0.674	0.5571918	null	conserved hypothetical protein	PA4490	331	95.18
1687534	0.985	0.8065256	null	hypothetical protein	PA4495	132	92.29
1687537	0.978	0.848176	gatA	Glu-tRNA(Gln) amidotransferase subunit A	PA4483	37	94.44
1687541	0.901	0.7822529	null	probable two-component response regulator	PA4493	153	94.27
1687543	0.910	0.79990923	null	probable two-component sensor	PA4494	1	95.05
1687546	0.847	0.6673004	null	probable transcriptional regulator	PA4499	45	94.86
1687549	0.878	0.580829	null	probable metallopeptidase	PA4498	164	94.68
1687552	0.435	0.25031203	null	probable porin	PA4501	522	92.12
1687555	1.122	0.76482743	null	probable binding protein component of ABC	PA4496	1	92.41
1687558	1.346	1.1576127	null	probable ATP-binding component of ABC dipeptide	PA4506	7	92.22
1687561	1.232	1.0166026	null	probable permease of ABC transporter	PA4504	30	94.18
1687564	1.019	1.1619047	null	probable permease of ABC transporter	PA4503	330	94.39
1687567	1.700	1.2165905	null	probable ATP-binding component of ABC	PA4505	249	92.23
1687570	0.762	0.37835008	null	hypothetical protein	PA4507	20	95.01
1687573	0.137	-0.23750976	null	conserved hypothetical protein	PA4510	37	94.65
1687576	0.873	0.6504935	null	conserved hypothetical protein	PA4491	379	93.22
1687579	0.890	0.6234411	lpxO1	lipopolysaccharide biosynthetic protein LpxO1	PA4512	15	92.7
1687582	0.293	-1.8053608	null	hypothetical protein	PA4509	132	93.26
1687585	1.575	1.4107617	null	conserved hypothetical protein	PA4511	1	93.85
1687588	1.312	1.1430088	null	probable transcriptional regulator	PA4508	383	93.99
1687590	1.065	2.5835185	null	probable binding protein component of ABC	PA4502	428	92.17
1687593	0.231	1.80454	null	hypothetical protein	PA4516	128	94.69
1687596	1.217	0.78875095	null	probable binding protein component of ABC	PA4497	111	92.68
1687599	0.970	0.7401685	null	conserved hypothetical protein	PA4515	170	93.5
1687602	0.905	2.7926564	null	probable outer membrane receptor for iron	PA4514	17	92.6
1687605	1.162	1.1029104	null	hypothetical protein	PA4518	34	94.89
1687608	0.702	0.51125944	null	conserved hypothetical protein	PA4488	257	93.27
1687611	1.400	0.99938774	ampD	beta-lactamase expression regulator AmpD	PA4522	34	94.39
1687614	0.996	0.8237643	speC	ornithine decarboxylase	PA4519	99	92.49
1687617	0.968	1.0077178	pilA	type 4 fimbrial precursor PilA	PA4525	90	92.82
1687618	0.977	0.69829106	null	hypothetical protein	PA4521	179	93.55
1687621	1.048	0.784772	pilB	type 4 fimbrial biogenesis protein PilB	PA4526	1	92.36
1687624	1.100	1.2846154	null	probable oxidoreductase	PA4513	62	94.81
1687627	0.922	0.89939994	pilC	still frameshift type 4 fimbrial biogenesis	PA4527	557	92.23
1687630	0.960	0.72580135	null	hypothetical protein	PA4531	141	92.63
1687632	0.670	0.50016654	coaE	dephosphocoenzyme A kinase	PA4529	1	93.19
1687635	0.857	0.7520855	null	conserved hypothetical protein	PA4530	39	92.09
1687637	0.912	0.6986475	null	conserved hypothetical protein	PA4517	76	92.15
1687640	7.532	1.4307077	null	probable transporter	PA3781	685	96.45
1687643	0.555	0.35014397	null	probable chemotaxis transducer	PA4520	4	92.09
1687646	1.083	0.87764007	null	hypothetical protein	PA4534	77	92.39
1687649	0.944	0.7187169	null	hypothetical protein	PA4532	174	92.93
1687652	0.951	0.66651696	null	hypothetical protein	PA4533	70	95.04
1687655	0.864	0.52165055	null	hypothetical protein	PA4539	22	97.04
1687658	1.003	0.71472967	null	hypothetical protein	PA4537	1	93.16
1687660	1.163	1.0015566	nadC	nicotinate-nucleotide pyrophosphorylase	PA4524	1	92.88
1687663	1.376	1.0094358	null	hypothetical protein	PA4540	1	95.5
1687666	0.836	0.61319923	pilD	type 4 prepilin peptidase PilD	PA4528	93	93.8
1687670	0.672	0.5852232	null	probable binding protein component of ABC	PA4500	142	93.71
1687672	1.645	1.4560438	null	hypothetical protein	PA4523	109	93.04
1687675	0.899	0.7312076	rluD	pseudouridine synthase	PA4544	335	92.87
1687678	1.005	0.7221709	ndh	NADH dehydrogenase	PA4538	106	92.82
1687681	1.009	0.62666667	null	hypothetical protein	PA4541	21	93.16
1687684	1.265	0.87931377	null	hypothetical protein	PA4535	19	93.96
1687687	0.789	0.5746627	null	conserved hypothetical protein	PA4543	557	95.64
1687690	1.009	-0.640126	fimT	type 4 fimbrial biogenesis protein FimT	PA4549	100	94.67
1687693	0.994	0.81708544	comL	competence protein ComL	PA4545	34	93.11
1687696	0.946	0.8262895	fimU	type 4 fimbrial biogenesis protein FimU	PA4550	451	96.03
1687699	1.227	-0.6878139	null	probable D-amino acid oxidase	PA4548	304	96.5
1687702	1.034	0.81694955	pilW	type 4 fimbrial biogenesis protein PilW	PA4552	23	92.47
1687705	1.152	0.99448645	pilE	type 4 fimbrial biogenesis protein PilE	PA4556	155	94.84
1687708	1.220	0.9983538	pilY2	type 4 fimbrial biogenesis protein PilY2	PA4555	218	92.12
1687710	1.233	0.45033684	pilX	type 4 fimbrial biogenesis protein PilX	PA4553	72	93.84
1687713	1.219	1.0162328	clpB	ClpB protein	PA4542	7	92.81
1687716	0.880	0.72858465	lspA	prolipoprotein signal peptidase	PA4559	90	92.16
1687719	0.980	0.84635127	pilS	two-component sensor PilS	PA4546	35	93.76
1687722	0.913	0.7595824	lytB	LytB protein	PA4557	61	92.64
1687725	1.105	0.6481673	pilV	type 4 fimbrial biogenesis protein PilV	PA4551	23	92.03
1687728	0.860	0.7102114	null	probable peptidyl-prolyl cis-trans isomerase,	PA4558	1	95.74
1687731	0.463	0.15346915	ribF	riboflavin kinase/FAD synthase	PA4561	1	96.28
1687734	0.742	0.7108302	rpsT	30S ribosomal protein S20	PA4563	42	97.05
1687735	0.628	0.60779357	rpmA	50S ribosomal protein L27	PA4567	154	95.32
1687737	1.178	1.0342791	pilY1	type 4 fimbrial biogenesis protein PilY1	PA4554	135	92.7
1687740	1.217	0.6713955	null	conserved hypothetical protein	PA4562	14	93.13
1687743	1.081	0.82951784	obg	GTP-binding protein Obg	PA4566	65	92.04
1687746	0.834	0.6756034	null	conserved hypothetical protein	PA4564	63	93.51
1687749	0.938	0.38378787	null	hypothetical protein	PA4570	25	93.86
1687751	0.783	0.56334305	null	conserved hypothetical protein	PA4489	238	93.23
1687754	0.911	1.007026	rplU	50S ribosomal protein L21	PA4568	105	92.27
1687756	0.998	0.74908775	fklB	peptidyl-prolyl cis-trans isomerase FklB	PA4572	3	92.03
1687759	1.043	0.80405456	null	hypothetical protein	PA4573	27	94.46
1687761	1.032	0.9374544	ispB	octaprenyl-diphosphate synthase	PA4569	54	95.34
1687764	1.000	0.86960304	null	hypothetical protein	PA4577	118	96.97
1687765	0.831	0.5308348	pilR	two-component response regulator PilR	PA4547	174	95.2
1687768	1.056	0.9209753	null	probable cytochrome c	PA4571	22	94.67
1687771	1.077	0.7686108	null	conserved hypothetical protein	PA4580	89	92.74
1687774	0.845	0.74044406	proB	glutamate 5-kinase	PA4565	72	94.34
1687777	0.709	0.35490623	null	conserved hypothetical protein	PA4582	125	92.67
1687780	0.864	0.7025758	ileS	isoleucyl-tRNA synthetase	PA4560	66	92.27
1687783	1.255	0.88452375	null	conserved hypothetical protein	PA4352	13	95.5
1687786	1.400	0.94021714	null	conserved hypothetical protein	PA4574	287	92.3
1687790	1.086	0.8223219	null	hypothetical protein	PA4575	211	93.41
1687791	0.992	0.26301473	null	conserved hypothetical protein	PA4584	171	92.51
1687794	1.359	1.3434962	null	hypothetical protein	PA4578	244	92.62
1687797	1.019	1.3291503	null	hypothetical protein	PA4586	103	96.72
1687799	1.108	0.8086937	null	conserved hypothetical protein	PA4583	159	95.96
1687802	1.009	4.0733976	rtcA	RNA 3'-terminal phosphate cyclase	PA4585	632	94.93
1687805	1.109	0.9863256	null	probable ATP-dependent protease	PA4576	28	96.57
1687808	1.260	0.88116485	null	probable permease of ABC transporter	PA4593	1	97.46
1687811	1.044	0.66498697	null	probable ATP-binding component of ABC	PA4594	68	92.31
1687814	0.990	0.84893036	null	hypothetical protein	PA4579	48	97.93
1687817	1.112	0.72048914	null	probable transcriptional regulator	PA4596	109	92.74
1687820	1.093	-0.18333331	gdhA	glutamate dehydrogenase	PA4588	130	94.82
1687823	0.823	0.67937344	pra	protein activator	PA4590	11	92.58
1687826	5.502	3.1095483	rtcR	transcriptional regulator RtcR	PA4581	244	94.4
1687829	0.658	8.140238	null	probable outer membrane protein precursor	PA4592	60	97.35
1687832	1.009	-0.15794238	oprJ	Multidrug efflux outer membrane protein OprJ	PA4597	355	96.13

1687835	0.887	2.1661835	mexC	Resistance-Nodulation-Cell Division (RND)	PA4599	92	96.3
1687838	1.069	0.7951918	nfxB	transcriptional regulator NfxB	PA4600	384	93.98
1687841	1.369	1.286734	null	hypothetical protein	PA4591	2	96.61
1687844	1.133	-0.69666666	null	probable outer membrane protein precursor	PA4589	155	96.09
1687847	1.187	1.1335933	null	hypothetical protein	PA4608	79	95.65
1687850	0.970	0.8008153	null	hypothetical protein	PA4607	8	93.23
1687853	0.869	0.7157664	null	conserved hypothetical protein	PA4604	1	93.43
1687856	0.674	0.46843338	null	hypothetical protein	PA4603	236	92.57
1687859	1.199	1.0541252	null	hypothetical protein	PA4611	55	92.27
1687860	1.000	0.878101	null	hypothetical protein	PA4610	375	94.04
1687863	1.147	0.90881693	mscL	conductance mechanosensitive channel	PA4614	326	96.52
1687865	0.906	0.21000504	null	conserved hypothetical protein	PA4612	1	92.85
1687868	1.144	0.9983202	null	probable oxidoreductase	PA4615	1	92.64
1687871	1.001	0.6876875	glyA3	serine hydroxymethyltransferase	PA4602	26	94.2
1687874	0.798	2.526238	null	conserved hypothetical protein	PA4617	89	92.75
1687877	1.009	2.5771427	mexD	Resistance-Nodulation-Cell Division (RND)	PA4598	78	92.71
1687880	0.884	0.770665	null	hypothetical protein	PA4618	28	96.39
1687883	1.091	0.7710789	null	hypothetical protein	PA4620	212	93.92
1687886	0.927	0.2176969	null	probable c4-dicarboxylate-binding protein	PA4616	329	92.51
1687889	3.572	0.9934781	null	probable major facilitator superfamily (MFS)	PA4622	100	92.32
1687892	11.451	9.9394	null	hypothetical protein	PA4623	87	95.89
1687895	1.133	0.030769229	katB	catalase	PA4613	269	93.63
1687898	1.043	0.9270035	null	conserved hypothetical protein	PA4606	177	96.7
1687901	0.707	0.38659465	null	hypothetical protein	PA4625	23	93.17
1687904	0.981	0.68771017	null	hypothetical protein	PA4624	3	92.16
1687907	0.725	-7.3459954	null	probable c-type cytochrome	PA4619	252	94.27
1687910	1.202	0.95054567	null	probable oxidoreductase	PA4621	90	94
1687913	0.968	0.47279182	hprA	glycerate dehydrogenase	PA4626	79	96.84
1687916	1.646	1.3358247	ccpR	cytochrome c551 peroxidase precursor	PA4587	268	97
1687919	1.057	0.87325287	null	hypothetical protein	PA4629	6	92.22
1687922	0.739	-0.18541667	null	hypothetical protein	PA4630	218	93.89
1687925	0.754	0.6173183	null	hypothetical protein	PA4631	18	92.59
1687928	0.911	0.75307035	null	hypothetical protein	PA4632	323	93.98
1687931	1.044	0.78919345	lysP	lysine-specific permease	PA4628	213	92.64
1687934	0.933	0.65040433	null	hypothetical protein	PA4638	55	94.29
1687936	1.041	0.6295759	null	hypothetical protein	PA4637	188	92.28
1687937	3.582	1.7160972	null	conserved hypothetical protein	PA4635	172	94.12
1687940	0.798	0.18982455	null	hypothetical protein	PA4636	2	92.06
1687943	1.129	1.0135717	null	hypothetical protein	PA4639	1	95.14
1687946	1.354	1.0519084	null	probable ATP-binding component of ABC	PA4595	11	92.67
1687949	0.864	0.7002603	null	hypothetical protein	PA4642	132	94.14
1687950	0.791	0.5951538	null	hypothetical protein	PA4643	308	95.76
1687953	0.707	0.3602299	morA	motility regulator	PA4601	326	97.77
1687956	1.246	0.94368637	null	probable chemotaxis transducer	PA4633	332	92.43
1687959	0.679	2.7085361	null	hypothetical protein	PA4644	70	94.23
1687962	1.182	0.93984807	null	probable purine/pyrimidine phosphoribosyl	PA4645	199	92.3
1687965	0.869	0.6992882	mqoB	malate:quinone oxidoreductase	PA4640	124	93.87
1687968	1.009	0.009783531	null	hypothetical protein	PA4648	336	92.49
1687971	1.032	0.7907609	upp	uracil phosphoribosyltransferase	PA4646	127	92.65
1687974	1.289	1.0597079	null	hypothetical protein	PA4649	169	93.94
1687977	1.476	1.1540906	null	hypothetical protein	PA4650	1	92.21
1687980	1.009	1.4320238	null	probable pili assembly chaperone	PA4651	203	92.62
1687983	1.647	1.6610638	null	hypothetical protein	PA4653	155	94.08
1687986	0.339	0.15943386	null	conserved hypothetical protein	PA4656	276	97.72
1687989	0.656	0.41160998	null	probable major facilitator superfamily (MFS)	PA4654	11	92.71
1687992	1.071	0.7780343	null	hypothetical protein	PA4634	1	93.48
1687995	1.107	0.7889528	null	hypothetical protein	PA4657	43	94.94
1687998	1.010	0.7445816	uraA	uracil permease	PA4647	82	93.88
1688001	1.886	1.8147415	pagL	Lipid A 3-O-deacylase	PA4661	124	93.81
1688004	0.515	0.2496777	null	probable transcriptional regulator	PA4659	162	96.86
1688007	0.913	0.7254384	null	hypothetical protein	PA4658	1	93.52
1688010	1.004	0.83250225	hemH	ferrochelatase	PA4655	3	94
1688013	0.948	0.8240706	moeB	molybdopterin biosynthesis MoeB protein	PA4663	227	97.14
1688016	1.133	0.8659104	hemK	probable methyl transferase	PA4664	12	92.57
1688019	0.929	0.7782316	null	conserved hypothetical protein	PA4627	193	96.05
1688022	0.824	0.5271594	phr	deoxyribodipyrimidine photolyase	PA4660	21	92.51
1688025	1.425	1.4385803	null	hypothetical protein	PA4652	55	97.33
1688028	0.833	0.6757165	prfA	peptide chain release factor 1	PA4665	23	92.17
1688031	0.893	0.8617222	null	probable lipoprotein localization protein LolB	PA4668	323	92.05
1688034	0.886	0.787735	prs	ribose-phosphate pyrophosphokinase	PA4670	235	93
1688037	1.274	1.0389503	null	conserved hypothetical protein	PA4674	136	92.02
1688038	0.637	0.49128515	ipk	isopentenyl monophosphate kinase	PA4669	57	94.47
1688041	0.725	0.60305613	murI	glutamate racemase	PA4662	47	95.57
1688044	0.491	0.3413871	null	hypothetical protein	PA4677	35	95.83
1688047	0.917	0.8847216	null	probable ribosomal protein L25	PA4671	15	92.85
1688050	0.769	0.4950425	rimI	peptide n-acetyltransferase RimI	PA4678	63	92.51
1688053	0.983	0.71384466	null	probable carbonic anhydrase	PA4676	2	92.86
1688056	0.792	0.6058626	hemA	glutamyl-tRNA reductase	PA4666	143	92.11
1688059	1.228	0.39619046	null	hypothetical protein	PA4682	51	97.71
1688062	0.997	0.8563121	null	conserved hypothetical protein	PA4673	72	92.67
1688065	0.718	0.6461638	null	peptidyl-tRNA hydrolase	PA4672	150	92
1688068	0.811	0.6762035	null	hypothetical protein	PA4683	295	93.22
1688071	0.372	-5.0308332	null	hypothetical protein	PA4680	364	94.09
1688074	1.009	0.06845239	null	hypothetical protein	PA4685	39	92.46
1688077	1.055	0.779168	null	hypothetical protein	PA4667	3	92.63
1688080	0.953	0.5074843	null	hypothetical protein	PA4689	23	92.63
1688083	0.944	0.1662441	null	hypothetical protein	PA4684	16	96.98
1688086	1.152	0.95851135	hitA	ferric iron-binding periplasmic protein HitA	PA4687	43	96.47
1688089	1.176	0.90226376	null	hypothetical protein	PA4691	51	94.17
1688093	0.709	0.5380255	null	hypothetical protein	PA4679	553	97.05
1688094	1.067	0.911544	null	conserved hypothetical protein	PA4692	33	92.03
1688097	0.889	0.677986	null	hypothetical protein	PA4536	308	93.09
1688100	1.004	0.89791334	pssA	phosphatidylserine synthase	PA4693	135	93.03
1688103	0.835	0.72115827	ilvI	acetolactate synthase large subunit	PA4696	10	92.76
1688106	1.247	1.079171	ilvC	ketol-acid reductoisomerase	PA4694	255	92.12
1688109	0.833	0.7190889	null	hypothetical protein	PA4698	134	94.2
1688111	0.985	0.8432867	ilvH	acetolactate synthase isozyme III small subunit	PA4695	370	92.49
1688114	0.923	0.7163325	null	hypothetical protein	PA4681	185	92.35
1688117	0.980	0.3295012	null	hypothetical protein	PA4703	39	93.93
1688120	0.848	0.67366725	radA	DNA repair protein RadA	PA4609	300	93.02
1688123	0.995	0.6195388	mrcB	penicillin-binding protein 1B	PA4700	264	97.76
1688126	1.121	0.97642994	null	conserved hypothetical protein	PA4701	60	92.5
1688129	0.245	-4.3315105	null	hypothetical protein	PA4705	180	92.16
1688132	1.061	0.80937517	null	hypothetical protein	PA4697	288	93.29
1688137	1.045	0.8007413	null	hypothetical protein	PA4702	233	92.57
1688138	1.035	1.1307907	null	hypothetical protein	PA4708	240	92.07
1688141	1.222	0.89560544	hitB	iron (III)-transport system permease HitB	PA4688	133	95.41
1688144	1.088	1.0009855	null	hypothetical protein	PA4711	10	92.39
1688146	1.081	0.88519156	null	probable hemin degrading factor	PA4709	3	93.85

1688151	0.895	0.62007487	null	probable ATP-binding component of ABC	PA4706	711	93.82
1688152	1.262	1.0273758	null	probable TonB-dependent receptor	PA4675	45	94.47
1688155	0.981	0.71461	null	hypothetical protein	PA4713	3	93.43
1688158	0.993	0.5190977	null	conserved hypothetical protein	PA4714	94	95.23
1688161	0.719	0.3906335	null	hypothetical protein	PA4716	324	97
1688164	1.086	0.88411397	null	conserved hypothetical protein	PA4717	7	94.9
1688167	1.167	0.9442412	null	hypothetical protein	PA4686	50	93.09
1688170	1.103	0.75750744	trmA	tRNA (uracil-5-)-methyltransferase	PA4720	35	94.12
1688173	0.981	0.7514045	null	probable aminotransferase	PA4715	61	92.72
1688176	0.900	0.6925057	null	hypothetical protein	PA4718	373	92.37
1688179	1.123	1.0789273	dksA	suppressor protein DksA	PA4723	146	92.16
1688182	0.757	0.5269348	null	conserved hypothetical protein	PA4721	123	92.42
1688185	0.835	0.55765593	null	hypothetical protein	PA4704	240	97.66
1688188	0.922	0.48150435	null	probable transporter	PA4719	55	92.19
1688191	0.602	0.3769607	null	probable aminotransferase	PA4722	348	97.84
1688194	1.062	0.91607875	panC	pantoate--beta-alanine ligase	PA4730	136	92.66
1688197	1.128	0.86585486	folK	2-amino-4-hydroxy-6-	PA4728	333	95.39
1688200	1.208	0.98448694	panB	3-methyl-2-oxobutanoate	PA4729	166	94.01
1688203	0.447	-6.0991516	null	probable aminoacyl-transfer RNA synthetase	PA4724	288	94.48
1688206	0.982	0.70116943	panD	aspartate 1-decarboxylase precursor	PA4731	31	93.26
1688209	0.742	0.5416201	pcnB	poly(A) polymerase	PA4727	44	94.09
1688212	0.732	0.6096841	null	hypothetical protein	PA4737	59	94.91
1688214	0.747	0.53370816	null	conserved hypothetical protein	PA4738	32	92.67
1688216	1.065	0.79633856	null	hypothetical protein	PA4736	171	93.2
1688218	0.819	0.4013769	acsB	acetyl-coenzyme A synthetase	PA4733	126	94.12
1688221	1.016	0.6226168	pgi	glucose-6-phosphate isomerase	PA4732	3	92.59
1688224	0.701	0.55888355	null	conserved hypothetical protein	PA4739	18	92.4
1688226	0.665	0.72115284	rpsO	30S ribosomal protein S15	PA4741	155	92.7
1688228	1.009	0.06492901	phuR	Haem/Haemoglobin uptake outer membrane receptor	PA4710	141	95.91
1688231	0.828	0.65069187	null	hypothetical protein	PA4734	250	95.79
1688234	0.778	-0.34831113	null	probable permease of ABC transporter	PA4707	415	94.42
1688237	0.986	0.7208374	truB	tRNA pseudouridine 55 synthase	PA4742	43	94.37
1688240	1.111	0.9909064	infB	translation initiation factor IF-2	PA4744	64	94.16
1688243	0.734	0.7470153	tpiA	triosephosphate isomerase	PA4748	50	92.61
1688246	0.845	0.7392747	cbrB	two-component response regulator CbrB	PA4726	105	93.36
1688249	0.908	0.8113496	nusA	N utilization substance protein A	PA4745	18	92.71
1688252	0.970	0.97745144	secG	secretion protein SecG	PA4747	44	93.12
1688254	1.046	0.75816596	rbfA	ribosome-binding factor A	PA4743	15	92.76
1688257	0.915	0.88961047	null	conserved hypothetical protein	PA4746	68	92.04
1688260	0.920	0.79839325	folP	dihydropteroate synthase	PA4750	30	93.65
1688263	0.941	0.71571165	glmM	phosphoglucosamine mutase	PA4749	284	93.97
1688266	0.887	0.790202	cbrA	two-component sensor CbrA	PA4725	32	92.46
1688269	0.892	0.822703	null	conserved hypothetical protein	PA4757	2	92.21
1688272	1.182	1.0236704	pnp	polyribonucleotide nucleotidyltransferase	PA4740	77	95.66
1688275	0.732	0.5077136	null	hypothetical protein	PA4735	355	96.74
1688278	0.913	0.6949877	null	hypothetical protein	PA4699	226	96.45
1688281	0.794	0.78516644	null	hypothetical protein	PA4754	50	96.23
1688284	0.615	0.45026866	ftsJ	cell division protein FtsJ	PA4752	216	93.28
1688287	1.062	0.86896145	carA	carbamoyl-phosphate synthase small chain	PA4758	275	94.29
1688290	1.281	1.0235546	carB	carbamoylphosphate synthetase large subunit	PA4756	40	94.21
1688294	1.001	0.8802055	null	conserved hypothetical protein	PA4753	212	92.28
1688295	0.908	0.7707401	dapB	dihydrodipicolinate reductase	PA4759	377	92.65
1688298	0.695	0.5744336	null	conserved hypothetical protein	PA4766	120	93.35
1688300	0.994	0.8600589	greA	transcription elongation factor GreA	PA4755	197	93.92
1688303	0.980	0.5102117	null	hypothetical protein	PA4712	90	92.38
1688306	0.753	0.6782385	fur	ferric uptake regulation protein	PA4764	72	94.35
1688309	0.965	0.83561885	omlA	Outer membrane lipoprotein OmlA precursor	PA4765	115	92.3
1688312	0.965	0.7744	dnaJ	DnaJ protein	PA4760	78	93.98
1688316	0.762	0.63594484	smpB	SmpB protein	PA4768	382	92.69
1688320	0.386	0.07877849	null	probable transcriptional regulator	PA4769	588	95.53
1688321	1.868	1.3586748	null	hypothetical protein	PA4773	32	92.6
1688324	1.019	-0.10672271	null	hypothetical protein	PA4775	139	95.76
1688328	1.382	0.9063638	pmrA	PmrA: two-component regulator system response	PA4776	294	93.66
1688329	0.726	0.62988	null	probable transcriptional regulator	PA4778	108	92.28
1688331	1.529	1.127402	null	hypothetical protein	PA4774	94	92
1688334	1.063	0.70503604	recN	DNA repair protein RecN	PA4763	32	92
1688337	0.926	0.70030487	null	conserved hypothetical protein	PA4767	63	92.12
1688340	1.072	0.92129356	null	hypothetical protein	PA4782	48	92.04
1688343	0.610	1.292803	null	hypothetical protein	PA4779	269	92.54
1688346	0.786	0.48125696	null	conserved hypothetical protein	PA4780	67	93.03
1688349	1.086	0.9666284	dnaK	DnaK protein	PA4761	166	94.11
1688352	1.742	1.25648	lldP	L-lactate permease	PA4770	250	92.12
1688355	1.413	0.62895423	null	probable transcriptional regulator	PA4784	178	92.5
1688358	0.998	0.7180842	null	probable acyl-CoA thiolase	PA4785	23	94.06
1688361	0.904	0.784895	ftsH	cell division protein FtsH	PA4751	68	92.41
1688364	1.152	0.8967326	null	probable ferredoxin	PA4772	89	92.86
1688367	1.009	-0.9746887	pmrB	PmrB: two-component regulator system signal	PA4777	612	96.46
1688370	1.101	0.87429476	null	probable two-component response regulator	PA4781	518	94.9
1688374	0.878	0.3784212	null	conserved hypothetical protein	PA4789	114	92.15
1688375	0.517	0.19160832	null	conserved hypothetical protein	PA4790	186	97.96
1688378	0.499	0.21035643	null	probable short-chain dehydrogenase	PA4786	850	93.2
1688382	0.888	0.34993586	null	hypothetical protein	PA4791	405	96.56
1688384	0.630	0.46577153	null	probable transcriptional regulator	PA4787	459	92.71
1688388	0.905	0.6689273	null	hypothetical protein	PA4794	77	92.41
1688390	0.950	0.6752473	null	hypothetical protein	PA4793	121	94.68
1688393	1.244	0.82431924	null	hypothetical protein	PA4796	36	94.37
1688397	1.002	0.7955159	null	hypothetical protein	PA4795	326	95.64
1688399	0.932	0.7963049	null	probable transposase	PA4797	45	92.38
1688402	0.434	-0.34177968	null	hypothetical protein	PA4798	273	94.37
1688405	0.982	0.8125976	null	conserved hypothetical protein	PA4792	480	94.1
1688408	1.236	0.1254421	null	hypothetical protein	PA4799	94	92.24
1688411	0.593	0.4437136	null	hypothetical protein	PA4801	208	94.72
1688414	1.009	1.4763815	null	hypothetical protein	PA4800	77	93.97
1688417	1.013	0.80013025	null	hypothetical protein	PA4803	4	92.57
1688420	1.276	1.0552237	null	hypothetical protein	PA4802	647	97.67
1688423	0.934	0.06675826	null	probable transcriptional regulator	PA4806	617	92.75
1688426	0.765	0.3092195	null	probable amino acid permease	PA4804	53	93.24
1688429	1.037	0.92656434	fdnH	nitrate-inducible formate dehydrogenase, beta	PA4811	42	93.79
1688432	0.843	0.72547096	fdnI	nitrate-inducible formate dehydrogenase, gamma	PA4810	378	92.37
1688435	1.009	-3.5368686	null	probable class III aminotransferase	PA4805	56	93.41
1688439	1.108	0.95761645	null	hypothetical protein	PA4788	184	96.96
1688441	0.623	0.29090118	fdhE	FdhE protein	PA4809	642	93.12
1688444	1.223	0.93273914	lipC	lipase LipC	PA4813	43	97.18
1688447	1.042	0.8457304	null	hypothetical protein	PA4815	139	96.83
1688450	1.615	-1.493789	null	hypothetical protein	PA4820	304	93
1688453	0.043	0.3206763	null	probable glycosyl transferase	PA4819	580	92.5
1688456	1.076	0.28111115	fadH2	2,4-dienoyl-CoA reductase FadH2	PA4814	109	94.4
1688459	1.009	1.2542796	null	hypothetical protein	PA4817	273	93.17
1688462	1.090	1.863847	null	hypothetical protein	PA4823	121	92.62

1688463	1.587	1.2551088	null	conserved hypothetical protein	PA4783	42	92.04
1688466	0.819	0.43888465	selA	L-seryl-tRNA(ser) selenium transferase	PA4808	648	94.2
1688470	1.008	0.73348594	null	hypothetical protein	PA4816	951	95.25
1688472	3.098	1.0482517	null	hypothetical protein	PA4826	157	95.22
1688474	1.343	0.98029804	null	hypothetical protein	PA4824	400	95.47
1688477	1.082	1.1785715	mgtA	Mg(2+) transport ATPase, P-type 2	PA4825	93	95.89
1688480	0.963	0.80279076	fdnG	formate dehydrogenase-O, major subunit	PA4812	431	95.76
1688483	1.939	2.5079255	null	conserved hypothetical protein	PA4818	289	93.52
1688486	0.795	0.6566889	selB	selenocysteine-specific elongation factor	PA4807	100	93.14
1688489	3.212	1.9716667	null	probable transcriptional regulator	PA4831	2	92.29
1688492	0.843	0.48046693	null	conserved hypothetical protein	PA4833	7	92.33
1688495	0.383	0.057806514	null	arylamine N-acetyltransferase	PA4827	15	93.32
1688498	1.009	-1.8085712	null	hypothetical protein	PA4822	287	93.07
1688501	1.000	-6.5443964	null	hypothetical protein	PA4830	458	94.19
1688504	1.679	0.38428575	null	hypothetical protein	PA4834	264	96.12
1688507	1.178	0.56923306	null	hypothetical protein	PA4836	36	92.34
1688510	1.737	1.6685209	null	conserved hypothetical protein	PA4828	222	92.49
1688513	0.863	0.8400067	grpE	heat shock protein GrpE	PA4762	432	92.19
1688516	1.222	1.8395904	null	hypothetical protein	PA4835	408	97.63
1688520	0.716	0.52509314	null	conserved hypothetical protein	PA4840	297	92.23
1688522	1.153	0.50095224	null	probable transporter	PA4821	755	92.68
1688525	1.013	0.8107053	null	hypothetical protein	PA4838	2	92.77
1688528	0.838	0.6949604	null	conserved hypothetical protein	PA4841	362	96.51
1688531	1.009	1.6111367	null	probable short-chain dehydrogenase	PA4832	144	94.55
1688534	0.810	0.6573454	aroQ1	3-dehydroquinate dehydratase	PA4846	1	92.56
1688537	1.051	0.9124091	accB	biotin carboxyl carrier protein (BCCP)	PA4847	35	92.04
1688540	0.986	0.86843663	null	hypothetical protein	PA4842	132	94.75
1688543	0.859	0.25569656	prmA	ribosomal protein L11 methyltransferase	PA4850	239	93.68
1688546	0.811	0.5335488	null	conserved hypothetical protein	PA4852	12	92.51
1688549	0.912	0.69089437	null	hypothetical protein	PA4849	74	95.65
1688552	1.067	0.9218235	fis	DNA-binding protein Fis	PA4853	32	92.54
1688555	1.533	0.9740513	null	probable chemotaxis transducer	PA4844	85	93.97
1688558	0.978	0.7714644	null	hypothetical protein	PA4851	96	93.28
1688561	1.009	1.1950446	null	probable outer membrane protein precursor	PA4837	180	94.27
1688564	1.268	0.9750289	lldD	L-lactate dehydrogenase	PA4771	355	92.49
1688567	1.130	0.62389094	null	hypothetical protein	PA4857	44	92.54
1688570	1.460	1.0112932	speA	biosynthetic arginine decarboxylase	PA4839	7	93.89
1688573	0.886	0.775926	accC	biotin carboxylase	PA4848	46	94.25
1688576	0.940	0.8118393	retS	RetS (Regulator of Exopolysaccharide and Type	PA4856	33	95.54
1688579	0.941	0.4832651	dipZ	thiol:disulfide interchange protein DipZ	PA4845	195	92.01
1688582	1.188	0.98371714	purH	phosphoribosylaminoimidazolecarboxamide	PA4854	343	95.28
1688585	1.097	0.8338485	null	probable ATP-binding component of ABC	PA4861	9	97.8
1688588	1.404	-0.47985077	null	conserved hypothetical protein	PA4858	89	94.39
1688591	1.043	0.53673637	ureB	urease beta subunit	PA4867	98	92.35
1688593	1.009	-2.8260398	null	hypothetical protein	PA4863	101	95.24
1688596	1.009	-0.9665158	null	probable permease of ABC transporter	PA4859	142	92.34
1688599	1.030	4.984603	null	conserved hypothetical protein	PA4870	5	96.17
1688602	0.342	0.07889674	ureD	urease accessory protein	PA4864	146	97.12
1688605	1.087	0.7696834	purD	phosphoribosylamine--glycine ligase	PA4855	16	96.89
1688609	1.016	0.73647803	ureA	urease gamma subunit	PA4865	182	96.71
1688611	0.941	0.80903137	null	conserved hypothetical protein	PA4866	454	93.2
1688614	0.944	-0.81731606	null	hypothetical protein	PA4871	95	94.41
1688617	1.009	1.1227274	null	hypothetical protein	PA4869	382	92.42
1688620	1.084	0.932399	null	hypothetical protein	PA4875	97	95.04
1688623	1.693	1.3158085	null	probable heat-shock protein	PA4873	26	94.04
1688626	0.899	0.72174966	null	probable transcriptional regulator	PA4878	32	92.04
1688629	2.490	1.5423281	null	hypothetical protein	PA4877	47	93.28
1688631	1.276	0.91492796	osmE	osmotically inducible lipoprotein OsmE	PA4876	53	92.32
1688634	1.234	0.5030553	lpd3	dihydrolipoamide dehydrogenase 3	PA4829	242	94.55
1688637	1.287	1.129265	null	hypothetical protein	PA4872	31	94.42
1688642	0.884	0.70204175	null	conserved hypothetical protein	PA4874	313	93.88
1688643	0.986	0.962865	null	hypothetical protein	PA4882	113	95.71
1688646	1.493	1.1950333	null	probable bacterioferritin	PA4880	10	93.66
1688651	14.722	14.399069	null	hypothetical protein	PA4881	153	94.17
1688652	1.009	1.5046962	null	hypothetical protein	PA4883	25	93.13
1688655	1.009	0.7266666	null	probable ATP-binding component of ABC	PA4862	26	92.27
1688658	0.668	0.5466334	null	conserved hypothetical protein	PA4888	359	92.59
1688661	1.017	0.61755365	ureC	urease alpha subunit	PA4868	64	93.25
1688664	0.872	0.71383727	null	probable oxidoreductase	PA4889	143	96.48
1688667	1.119	0.7740495	irlR	two-component response regulator	PA4885	165	93.27
1688670	0.995	0.55609834	ureF	urease accessory protein UreF	PA4892	515	93.88
1688673	0.665	0.5762165	null	probable major facilitator superfamily (MFS)	PA4887	1	94.71
1688676	1.636	1.1091396	null	hypothetical protein	PA4894	229	95.73
1688678	0.433	-0.14453506	ureE	urease accessory protein UreE	PA4891	322	92.16
1688681	0.974	0.67114925	ureG	urease accessory protein UreG	PA4893	339	92.8
1688684	1.237	1.0490656	null	probable sigma-70 factor, ECF subfamily	PA4896	202	94.48
1688687	1.173	0.83856213	null	hypothetical protein	PA4884	359	94.86
1688690	0.996	0.77359986	null	conserved hypothetical protein	PA4890	485	94.06
1688693	1.708	-4.676385	null	probable transmembrane sensor	PA4895	624	95.28
1688696	1.111	0.41547623	null	probable porin	PA4898	272	94.46
1688699	1.005	0.68743336	null	conserved hypothetical protein	PA4879	90	92.54
1688702	0.331	-0.016009402	null	probable transcriptional regulator	PA4906	522	92.93
1688705	1.048	0.8619107	null	probable short-chain dehydrogenase	PA4907	193	95.99
1688708	0.556	0.22316928	null	probable two-component sensor	PA4886	605	96.3
1688711	1.009	-0.28287554	vanB	vanillate O-demethylase oxidoreductase	PA4905	31	93.13
1688714	0.821	0.7119115	null	probable two-component response regulator	PA4843	28	92.82
1688717	1.009	0.6448288	mdlC	benzoylformate decarboxylase	PA4901	3	92.07
1688720	0.934	-0.5753885	null	hypothetical protein	PA4897	1	93.72
1688723	0.833	-0.38269228	null	hypothetical protein	PA4908	287	97.84
1688726	1.228	0.8550245	null	probable binding protein component of ABC	PA4913	25	92.65
1688729	1.009	-0.07308712	null	probable ATP-binding component of ABC	PA4910	5	92.26
1688732	1.009	-0.66402125	null	probable chemotaxis transducer	PA4915	134	92.52
1688735	0.888	0.6753218	null	hypothetical protein	PA4916	5	92.21
1688738	1.138	0.9989802	null	probable ATP-binding component of ABC	PA4909	8	93.46
1688741	1.122	0.8022046	null	probable major facilitator superfamily (MFS)	PA4900	22	92.23
1688744	1.241	0.9787399	null	hypothetical protein	PA4918	1	92.72
1688747	1.009	1.6010258	vanA	vanillate O-demethylase oxygenase subunit	PA4904	540	93.68
1688750	1.454	0.7097553	null	probable aldehyde dehydrogenase	PA4899	1078	96.04
1688753	1.382	1.458691	azu	azurin precursor	PA4922	3	92.02
1688756	0.926	0.78429806	null	hypothetical protein	PA4917	263	92.49
1688759	1.224	1.0612166	nadE	NH3-dependent NAD synthetase	PA4920	88	94.24
1688762	0.836	0.43286648	null	probable transcriptional regulator	PA4914	212	92.86
1688765	0.811	0.7086566	null	hypothetical protein	PA4921	286	95.07
1688768	1.199	1.1322178	pncB1	nicotinate phosphoribosyltransferase	PA4919	1107	95.9
1688771	1.009	-0.109419376	null	conserved hypothetical protein	PA4925	145	93.07
1688774	0.700	-0.14790733	null	conserved hypothetical protein	PA4926	50	93.68
1688777	1.356	-3.0583704	dnaB	replicative DNA helicase	PA4931	13	92.7
1688780	0.771	0.6671913	alr	biosynthetic alanine racemase	PA4930	1	95.49
1688783	1.205	1.2120109	rpsR	30S ribosomal protein S18	PA4934	83	92.33

1688785	0.926	0.9268546	null	hypothetical protein	PA4933	53	93.18
1688788	1.168	1.0209738	null	conserved hypothetical protein	PA4924	356	92.71
1688791	1.211	1.1762373	rpsF	30S ribosomal protein S6	PA4935	28	93.5
1688794	1.009	1.4680785	null	probable permease of ABC branched-chain amino	PA4911	909	93.11
1688797	0.947	0.8353483	null	probable rRNA methylase	PA4936	26	94.69
1688801	0.666	0.5989562	rplI	50S ribosomal protein L9	PA4932	387	92.85
1688803	0.845	0.5762139	null	conserved hypothetical protein	PA4927	1	92.33
1688806	1.173	0.9625195	purA	adenylosuccinate synthetase	PA4938	77	94.7
1688809	0.723	0.5646814	null	conserved hypothetical protein	PA4940	105	97.8
1688810	0.706	0.51842046	null	conserved hypothetical protein	PA4939	63	96.1
1688813	0.827	0.8034374	null	conserved hypothetical protein	PA4944	152	92.28
1688814	1.002	0.9216794	hflC	protease subunit HflC	PA4941	44	92.31
1688817	0.731	0.6793364	hflK	protease subunit HflK	PA4942	81	97.15
1688820	1.010	0.7493927	null	conserved hypothetical protein	PA4923	241	97.39
1688823	0.742	0.7553711	null	probable GTP-binding protein	PA4943	16	93.51
1688826	0.772	0.67649806	miaA	delta 2-isopentenylpyrophosphate transferase	PA4945	1	94.87
1688829	0.806	0.49205703	null	conserved hypothetical protein	PA4948	2	92
1688832	1.088	0.79392385	null	probable permease of ABC transporter	PA4860	126	95.52
1688835	1.058	0.80276424	null	conserved hypothetical protein	PA4950	1	96.86
1688838	0.727	0.60039157	mutL	DNA mismatch repair protein MutL	PA4946	305	95.46
1688841	0.890	0.8714102	amiB	N-acetylmuramoyl-L-alanine amidase	PA4947	1	93.2
1688844	1.153	0.9353781	orn	oligoribonuclease	PA4951	51	92.7
1688847	0.942	0.8254692	rnr	exoribonuclease RNase R	PA4937	323	94.58
1688850	0.985	0.85833645	motA	chemotaxis protein MotA	PA4954	127	95.31
1688853	1.017	0.6862653	null	conserved hypothetical protein	PA4952	26	94.34
1688856	0.818	0.67870367	rhdA	thiosulfate sulfurtransferase	PA4956	2	92.57
1688859	0.434	0.7819786	null	conserved hypothetical protein	PA4928	133	95.56
1688862	1.072	0.78578484	psd	phosphatidylserine decarboxylase	PA4957	486	95.64
1688865	1.337	1.0069606	null	probable permease of ABC branched chain amino	PA4912	3	92.63
1688868	1.385	1.1731774	null	hypothetical protein	PA4958	102	94.93
1688871	0.950	0.6604461	null	hypothetical protein	PA4963	151	92.44
1688874	0.792	2.0245168	null	hypothetical protein	PA4929	946	95.98
1688877	1.073	0.38657343	null	probable phosphoserine phosphatase	PA4960	1	95.36
1688880	1.006	0.70999753	null	hypothetical protein	PA4961	1	93.62
1688884	0.255	0.63000554	null	conserved hypothetical protein	PA4962	417	94.28
1688886	0.878	0.7319498	null	conserved hypothetical protein	PA4949	3	92.54
1688889	0.696	0.41470408	null	conserved hypothetical protein	PA4959	222	97.75
1688892	0.618	0.4137661	null	hypothetical protein	PA4955	240	95.85
1688895	0.862	0.6545619	null	conserved hypothetical protein	PA4969	85	95.3
1688898	0.854	0.6419059	aspP	adenosine diphosphate sugar pyrophosphatase	PA4971	24	94.68
1688901	0.702	0.61558586	null	conserved hypothetical protein	PA4970	160	95.27
1688904	0.848	0.68111616	null	conserved hypothetical protein	PA4968	312	95.62
1688907	1.323	1.0290754	null	hypothetical protein	PA4972	126	92.56
1688910	0.766	0.59209	null	hypothetical protein	PA4965	81	92.68
1688913	0.811	0.6870284	null	hypothetical protein	PA4966	291	97.14
1688916	0.963	0.73665345	null	probable outer membrane protein precursor	PA4974	60	97.65
1688919	0.857	0.68495315	parE	topoisomerase IV subunit B	PA4967	55	97.28
1688922	1.278	0.89777935	null	probable acyl-CoA dehydrogenase	PA4979	231	93.96
1688925	0.987	0.670269	thiC	thiamin biosynthesis protein ThiC	PA4973	46	93.53
1688928	1.180	1.0689682	null	probable enoyl-CoA hydratase/isomerase	PA4980	1	93.25
1688931	0.899	5.5400643	null	hypothetical protein	PA4978	458	93.23
1688934	1.914	-0.2222228	null	probable amino acid permease	PA4981	265	92.34
1688937	1.196	0.84710324	aspC	aspartate transaminase	PA4976	568	93.79
1688940	0.636	0.4598348	null	probable transcriptional regulator	PA4984	1	93.04
1688943	1.032	0.7082721	null	probable transcriptional regulator	PA4987	334	95.78
1688946	0.941	-1.8029226	null	NAD(P)H quinone oxidoreductase	PA4975	6	92.46
1688949	1.009	0.3810526	null	probable oxidoreductase	PA4986	59	92.57
1688952	0.592	0.29664943	waaA	3-deoxy-D-manno-octulosonic-acid (KDO)	PA4988	211	96.3
1688955	0.762	0.30039912	null	SMR multidrug efflux transporter	PA4990	171	92.84
1688957	1.187	1.066788	null	hypothetical protein	PA4993	268	93.21
1688960	0.862	0.52390474	null	hypothetical protein	PA4992	91	95.24
1688963	0.802	0.70318747	motB	chemotaxis protein MotB	PA4953	145	92.14
1688966	0.948	1.0465533	null	hypothetical protein	PA4985	178	94.84
1688969	0.765	0.6620113	null	probable acyl-CoA dehydrogenase	PA4995	25	97.01
1688972	0.828	0.706587	rfaE	LPS biosynthesis protein RfaE	PA4996	1	93.23
1688975	5.464	-1.0133566	null	probable acetolactate synthase large subunit	PA4977	101	94.81
1688978	1.024	0.7346599	null	probable transcriptional regulator	PA4989	348	97.35
1688981	0.758	0.3803978	null	probable glycosyl transferase	PA5000	81	94.04
1688984	1.009	0.84115493	null	probable acyl-CoA dehydrogenase	PA4994	75	92.27
1688987	1.002	0.87199175	null	conserved hypothetical protein	PA4998	51	92.52
1688990	0.747	0.62316877	waaL	O-antigen ligase, WaaL	PA4999	2	92.42
1688993	1.013	0.38750866	null	probable transcriptional regulator	PA4902	189	96.42
1688996	2.031	0.5110196	null	probable two-component sensor	PA4982	45	92.12
1688999	0.793	0.6897299	null	hypothetical protein	PA5003	119	93.94
1689002	0.699	0.52255946	null	probable two-component response regulator	PA4983	463	95.36
1689005	1.209	0.39181677	null	probable major facilitator superfamily (MFS)	PA4903	55	92.78
1689008	0.881	0.7105761	msbA	transport protein MsbA	PA4997	94	92.12
1689011	0.815	0.6953606	null	hypothetical protein	PA5002	84	95.74
1689014	0.908	0.75055236	null	probable carbamoyl transferase	PA5005	1	92.8
1689017	0.962	0.8105609	null	hypothetical protein	PA5001	119	94.51
1689020	0.735	0.5938825	null	hypothetical protein	PA5006	418	94.64
1689023	0.853	0.7021505	waaP	lipopolysaccharide kinase WaaP	PA5009	118	94.02
1689026	0.796	0.540249	null	hypothetical protein	PA5008	546	92.46
1689029	1.076	1.0572399	ilvE	branched-chain amino acid transferase	PA5013	16	92.47
1689032	1.036	0.85856616	msrA	peptide methionine sulfoxide reductase	PA5018	14	92.08
1689035	0.991	0.9037374	null	probable glycosyl transferase	PA5004	4	92.99
1689038	1.176	1.053406	aceF	dihydrolipoamide acetyltransferase	PA5016	142	92.86
1689041	1.162	0.6829542	null	hypothetical protein	PA4991	1	94.48
1689044	0.794	0.36946505	waaG	UDP-glucose:(heptosyl) LPS alpha	PA5010	89	94.07
1689047	0.906	0.7939346	null	conserved hypothetical protein	PA5017	97	94.35
1689050	1.109	1.0779492	aceE	pyruvate dehydrogenase	PA5015	1	92.62
1689053	0.787	0.5629712	null	conserved hypothetical protein	PA5024	44	93.23
1689056	1.015	1.0207965	metY	homocysteine synthase	PA5025	10	96.95
1689059	0.838	0.62147725	glnE	glutamate-ammonia-ligase adenylyltransferase	PA5014	325	94.56
1689062	0.981	0.7583768	null	conserved hypothetical protein	PA5028	2	93.72
1689065	0.973	0.9216509	null	conserved hypothetical protein	PA5022	3	92.38
1689068	0.824	0.54045165	null	hypothetical protein	PA5026	4	92.45
1689071	0.901	0.72445554	waaF	heptosyltransferase II	PA5012	414	96.95
1689074	0.778	0.4109621	null	conserved hypothetical protein	PA5019	85	95.88
1689077	1.250	0.8016931	null	probable acyl-CoA dehydrogenase	PA5020	25	92.57
1689080	1.469	1.0467632	null	probable short chain dehydrogenase	PA5031	98	97.91
1689083	1.379	1.1909491	null	hypothetical protein	PA5027	279	93.06
1689086	0.813	0.57472324	null	probable transcriptional regulator	PA5029	293	92.4
1689089	0.890	0.6720055	null	conserved hypothetical protein	PA5023	211	94.55
1689092	0.509	0.3853969	gltB	glutamate synthase large chain precursor	PA5036	69	92.6
1689095	1.398	1.0255082	null	probable major facilitator superfamily (MFS)	PA5030	169	92.96
1689098	0.906	0.3650384	null	probable transcriptional regulator	PA5032	7	96.46
1689101	1.438	1.0800326	gltD	glutamate synthase small chain	PA5035	19	92.37
1689104	1.057	0.9316157	pilN	type 4 fimbrial biogenesis protein PilN	PA5043	78	96.02

1689107	1.164	0.8477701	aroB	3-dehydroquinate synthase	PA5038	156	95.13
1689110	1.071	0.9331562	pilQ	Type 4 fimbrial biogenesis outer membrane	PA5040	1	96.13
1689113	1.062	0.93709385	pilP	type 4 fimbrial biogenesis protein PilP	PA5041	126	92.59
1689116	0.760	0.7021755	aroK	shikimate kinase	PA5039	1	92.65
1689119	1.181	0.9447347	pilO	type 4 fimbrial biogenesis protein PilO	PA5042	181	92.64
1689122	1.009	1.9316498	null	hypothetical protein	PA5033	75	97.84
1689125	0.858	0.75670946	rpmE	50S ribosomal protein L31	PA5049	66	92.82
1689126	0.832	0.63473845	pilM	type 4 fimbrial biogenesis protein PilM	PA5044	53	93.31
1689129	1.035	0.8915594	null	malic enzyme	PA5046	43	93.61
1689132	0.852	0.74929535	null	hypothetical protein	PA5037	432	95.56
1689135	0.830	0.63903564	null	hypothetical protein	PA5047	117	92.12
1689138	0.898	0.62398493	null	probable sodium/hydrogen antiporter	PA5021	818	93.12
1689141	0.811	0.66565746	null	probable nuclease	PA5048	426	92.83
1689144	1.220	0.8823801	hemE	uroporphyrinogen decarboxylase	PA5034	122	93.45
1689147	1.102	0.8297806	null	hypothetical protein	PA5052	119	92.63
1689150	1.336	0.9635234	ponA	penicillin-binding protein 1A	PA5045	32	93.14
1689153	0.789	0.5978036	hslV	heat shock protein HslV	PA5053	64	93.14
1689156	0.667	0.57369345	null	hypothetical protein	PA5055	2	92.74
1689159	0.907	0.78302467	null	conserved hypothetical protein	PA5061	33	92.76
1689162	1.022	0.79739314	parC	topoisomerase IV subunit A	PA4964	171	93.23
1689165	0.790	0.60346055	null	conserved hypothetical protein	PA5062	69	95.01
1689167	1.057	0.92296505	phaF	polyhydroxyalkanoate synthesis protein PhaF	PA5060	171	95.97
1689169	0.945	0.43637615	phaD	poly(3-hydroxyalkanoic acid) depolymerase	PA5057	108	93.82
1689172	0.855	0.6007025	ubiE	ubiquinone biosynthesis methyltransferase UbiE	PA5063	22	94.73
1689175	1.093	0.7963482	null	probable transcriptional regulator	PA5059	217	93.94
1689178	1.128	0.8424591	phaC1	poly(3-hydroxyalkanoic acid) synthase 1	PA5056	36	92.93
1689181	0.951	0.82945365	hisI	phosphoribosyl-AMP cyclohydrolase	PA5066	121	92.64
1689184	1.061	0.87275285	tatA	translocation protein TatA	PA5068	57	94.53
1689187	0.786	0.6843634	tatB	translocation protein TatB	PA5069	179	92.99
1689189	0.980	0.85112923	ubiB	ubiquinone biosynthetic protein UbiB	PA5065	59	95.25
1689192	0.699	0.5163501	tatC	transport protein TatC	PA5070	2	92.82
1689195	0.671	0.47468537	null	conserved hypothetical protein	PA5071	315	92.63
1689198	0.813	0.71068037	hisE	phosphoribosyl-ATP pyrophosphohydrolase	PA5067	50	94.45
1689201	1.097	0.8020445	null	hypothetical protein	PA5073	34	92.96
1689204	1.122	0.43465042	null	probable permease of ABC transporter	PA5075	86	92.09
1689207	0.934	0.50308895	null	probable ATP-binding component of ABC	PA5074	59	96.16
1689210	0.882	0.67236584	null	probable binding protein component of ABC	PA5076	119	92.53
1689213	0.837	0.61025774	null	prolyl aminopeptidase	PA5080	14	92.33
1689216	0.865	0.5757821	null	conserved hypothetical protein	PA5079	123	92.1
1689219	1.085	0.3922997	null	probable chemotaxis transducer	PA5072	3	92.69
1689222	1.076	-1.1607143	null	hypothetical protein	PA5081	125	92.61
1689225	1.062	0.81027794	null	conserved hypothetical protein	PA5078	291	92.48
1689228	0.734	0.6012447	waaC	heptosyltransferase I	PA5011	280	95.02
1689231	1.046	0.8493121	null	hypothetical protein	PA5086	569	93.58
1689232	0.757	0.54781455	mdoH	periplasmic glucans biosynthesis protein MdoH	PA5077	233	95.29
1689235	0.771	1.2291539	phaC2	poly(3-hydroxyalkanoic acid) synthase 2	PA5058	80	96.97
1689238	1.214	1.068759	null	probable binding protein component of ABC	PA5082	115	92.45
1689241	0.792	-1.2654762	null	conserved hypothetical protein	PA5083	216	93.78
1689244	1.009	3.7874322	null	probable oxidoreductase	PA5084	135	94.86
1689247	0.858	0.67622924	null	hypothetical protein	PA5064	86	96.15
1689250	1.251	0.8920013	hutG	N-formylglutamate amidohydrolase	PA5091	109	95.65
1689253	2.696	2.3267376	null	hypothetical protein	PA5088	121	92.09
1689256	1.156	0.925722	argS	arginyl-tRNA synthetase	PA5051	191	96.39
1689259	1.450	1.1192253	null	hypothetical protein	PA5087	1	93.68
1689262	0.668	0.5740271	hslU	heat shock protein HslU	PA5054	14	93.58
1689265	1.073	0.38331527	null	probable permease of ABC transporter	PA5095	256	93.79
1689268	1.009	1.0988096	null	probable histidine/phenylalanine ammonia-lyase	PA5093	708	94.86
1689271	0.663	0.270873	null	probable transcriptional regulator	PA5085	35	92.58
1689274	0.862	0.4906841	hutI	imidazolone-5-propionate hydrolase HutI	PA5092	491	97.54
1689277	1.009	-2.5657542	hutU	urocanase	PA5100	1	92.27
1689280	1.135	0.8535086	null	hypothetical protein	PA5101	222	96.06
1689283	1.009	0.28267974	null	probable binding protein component of ABC	PA5096	224	93.37
1689286	0.457	0.1410148	null	hypothetical protein	PA5102	96	95.7
1689289	1.545	0.53781986	null	hypothetical protein	PA5089	64	93.76
1689292	1.017	0.6558632	hutC	histidine utilization repressor HutC	PA5105	66	92.18
1689295	1.002	0.6377933	null	conserved hypothetical protein	PA5104	288	92.47
1689298	0.720	0.53687143	blc	outer membrane lipoprotein Blc	PA5107	178	92.55
1689301	0.257	0.1225061	null	hypothetical protein	PA5103	223	95.72
1689304	0.762	0.63494194	null	hypothetical protein	PA5109	426	95.19
1689307	0.797	0.69387704	fbp	fructose-1,6-bisphosphatase	PA5110	1	93.02
1689310	1.009	1.0901463	null	conserved hypothetical protein	PA5090	188	96.5
1689313	0.814	2.3221445	gloA3	lactoylglutathione lyase	PA5111	89	93.17
1689316	0.776	0.24483517	null	probable transcriptional regulator	PA5116	50	92.24
1689317	1.009	-0.6047619	null	probable transporter	PA5099	1	95.21
1689320	0.769	0.5308111	null	hypothetical protein	PA5113	129	92.23
1689323	0.763	0.21257885	null	conserved hypothetical protein	PA5106	35	93.01
1689326	1.009	-0.2920168	null	probable amino acid permease	PA5097	13	92.8
1689329	1.039	0.8561033	priA	primosomal protein N'	PA5050	1	93.2
1689332	0.938	0.80674964	null	hypothetical protein	PA5120	1	93.51
1689334	0.856	0.66237175	null	hypothetical protein	PA5108	153	95.32
1689336	1.257	1.3082902	glnA	glutamine synthetase	PA5119	67	93.92
1689339	1.009	1.7843488	null	conserved hypothetical protein	PA5115	36	92.04
1689342	0.714	0.088764675	null	probable ATP-binding component of ABC	PA5094	157	92.45
1689345	0.788	0.59180486	null	hypothetical protein	PA5114	305	97.41
1689348	1.286	0.73129684	ntrB	two-component sensor NtrB	PA5124	2	93
1689351	1.014	0.83812493	null	hypothetical protein	PA5123	377	95.15
1689354	0.826	0.67955756	null	hypothetical protein	PA5126	342	94.75
1689356	1.095	1.2286484	secB	secretion protein SecB	PA5128	28	93.81
1689359	0.987	0.85432446	estA	esterase EstA	PA5112	61	95.03
1689362	0.997	0.6263931	null	hypothetical protein	PA5122	156	93.95
1689365	1.037	0.72385854	ntrC	two-component response regulator NtrC	PA5125	227	93.82
1689368	0.928	0.754032	null	conserved hypothetical protein	PA5130	160	95.7
1689370	0.951	0.92939043	grx	glutaredoxin	PA5129	22	95.18
1689372	1.153	0.85265225	null	hypothetical protein	PA5132	74	95.2
1689375	1.045	0.92150986	typA	regulatory protein TypA	PA5117	157	92.14
1689378	1.096	0.6045314	null	conserved hypothetical protein	PA5135	343	95.72
1689381	0.768	0.5277778	thiI	thiazole biosynthesis protein ThiI	PA5118	110	95.07
1689384	0.964	0.7955921	hisF1	imidazoleglycerol-phosphate synthase, cyclase	PA5140	2	93.84
1689387	0.785	0.6769787	null	conserved hypothetical protein	PA5133	153	94.34
1689390	1.051	0.8088907	null	hypothetical protein	PA5138	57	94.43
1689393	0.905	0.71073806	hisH1	glutamine amidotransferase	PA5142	270	94.36
1689396	1.093	0.9005512	hisA	phosphoribosylformimino-5-aminoimidazole	PA5141	53	94.84
1689399	0.966	0.800906	pgm	phosphoglycerate mutase	PA5131	1	93.85
1689402	0.833	0.60277987	null	hypothetical protein	PA5136	53	93.61
1689405	0.468	1.3034925	null	hypothetical protein	PA5121	117	95.68
1689408	0.798	0.5195098	null	hypothetical protein	PA5144	1	95.85
1689409	1.053	0.9122387	null	conserved hypothetical protein	PA5148	1	93.97
1689412	2.117	-0.114511296	hisB	imidazoleglycerol-phosphate dehydratase	PA5143	43	94.08
1689415	0.172	0.6342246	null	probable short-chain dehydrogenase	PA5150	33	93.12

1689418	0.991	0.7623022	null	hypothetical protein	PA5139	582	92.74
1689421	1.009	1.0505335	null	probable rRNA methylase	PA5127	86	93.34
1689423	0.934	0.60153496	null	hypothetical protein	PA5145	90	96.59
1689426	0.885	0.64741737	null	hypothetical protein	PA5137	57	95.42
1689429	0.600	0.433635	null	hypothetical protein	PA5156	7	95.51
1689432	0.932	0.8615829	null	probable periplasmic binding protein	PA5153	472	92.41
1689435	0.802	0.66760933	null	conserved hypothetical protein	PA5149	357	96.35
1689438	0.724	0.69869286	null	probable permease of ABC transporter	PA5154	151	96.6
1689441	1.106	0.73911494	null	hypothetical protein	PA5151	179	94.53
1689444	0.814	0.44413814	null	probable ATP-binding component of ABC	PA5152	343	94.64
1689447	1.895	1.5297396	null	multidrug resistance protein	PA5159	102	95.12
1689450	1.217	0.5890151	null	probable transcriptional regulator	PA5157	3	92.5
1689453	1.169	0.38002592	null	probable outer membrane protein precursor	PA5158	67	96
1689456	1.093	0.9440409	rmlC	dTDP-4-dehydrorhamnose 3,5-epimerase	PA5164	8	94.61
1689459	1.053	0.8883225	rmlA	glucose-1-phosphate thymidylyltransferase	PA5163	45	96.71
1689462	0.816	0.65170145	rmlB	dTDP-D-glucose 4,6-dehydratase	PA5161	61	94.82
1689465	0.884	0.59938526	mutY	A / G specific adenine glycosylase	PA5147	87	93.56
1689468	0.922	0.71214354	null	probable two-component response regulator	PA5166	1	93.63
1689471	0.869	0.75531757	null	probable carboxyl-terminal protease	PA5134	111	97.43
1689474	1.116	0.8103523	null	probable c4-dicarboxylate-binding protein	PA5167	49	92.43
1689477	0.898	0.74230015	rmlD	dTDP-4-dehydrorhamnose reductase	PA5162	280	93.64
1689480	0.245	4.886421	null	probable dicarboxylate transporter	PA5168	500	93.74
1689483	1.020	0.9418311	arcC	carbamate kinase	PA5173	45	95.14
1689486	1.243	1.2811682	arcA	arginine deiminase	PA5171	22	92.45
1689489	0.865	0.7321785	cysQ	CysQ protein	PA5175	279	92.05
1689492	0.661	0.50117177	null	conserved hypothetical protein	PA5176	36	92.24
1689495	1.203	0.8636049	null	probable hydrolase	PA5177	60	92.62
1689498	1.038	0.8574894	null	drug efflux transporter	PA5160	253	94.79
1689501	1.160	1.0159934	null	conserved hypothetical protein	PA5178	23	92.71
1689504	0.714	0.46844134	null	probable transcriptional regulator	PA5179	341	92.77
1689507	0.301	-0.49572968	null	conserved hypothetical protein	PA5180	1	96.26
1689510	1.006	0.8298252	null	hypothetical protein	PA5183	320	95
1689512	1.009	1.5009091	null	probable C4-dicarboxylate transporter	PA5169	871	93.15
1689515	2.309	1.2528747	hutH	histidine ammonia-lyase	PA5098	180	95.61
1689518	1.009	2.83428	null	hypothetical protein	PA5184	104	92.68
1689521	0.668	0.49830413	null	probable beta-ketoacyl synthase	PA5174	25	92.58
1689524	0.783	0.38334346	null	conserved hypothetical protein	PA5185	334	96.56
1689527	1.009	5.9060926	null	probable oxidoreductase	PA5181	122	95.28
1689530	1.790	0.4171276	null	probable nitroreductase	PA5190	7	92.03
1689533	0.999	0.6194296	null	hypothetical protein	PA5191	116	94.03
1689535	0.494	0.3362669	null	probable permease of ABC transporter	PA5155	5	92.14
1689538	0.885	0.65317714	null	hypothetical protein	PA5194	74	96.74
1689541	0.856	0.60681784	yrfI	heat shock protein HSP33	PA5193	86	94.24
1689544	1.476	0.97125167	null	probable transcriptional regulator	PA5189	300	92.95
1689547	0.828	0.6926129	pckA	phosphoenolpyruvate carboxykinase	PA5192	1	93.07
1689550	0.487	-0.5619889	rimK	ribosomal protein S6 modification protein	PA5197	72	93.1
1689553	1.064	0.8579319	null	LD-carboxypeptidase	PA5198	3	94.67
1689556	1.105	0.80658454	null	hypothetical protein	PA5196	39	94.48
1689559	0.811	-0.019047618	null	probable iron-containing alcohol dehydrogenase	PA5186	809	94.83
1689562	0.843	0.6033972	ompR	two-component response regulator OmpR	PA5200	1	92.59
1689565	0.960	0.78892696	null	hypothetical protein	PA5146	488	95.84
1689568	1.046	0.14722821	null	probable two-component sensor	PA5165	418	93.05
1689571	0.762	0.6420838	null	hypothetical protein	PA5202	180	95.76
1689572	0.875	0.6839642	null	probable heat shock protein	PA5195	133	92.42
1689575	0.337	3.3097363	envZ	two-component sensor EnvZ	PA5199	254	92.15
1689578	0.669	0.4837162	argA	N-acetylglutamate synthase	PA5204	17	92.19
1689581	1.405	1.403854	arcD	arginine/ornithine antiporter	PA5170	27	92.22
1689584	1.127	0.9411583	null	probable phosphate transporter	PA5207	30	92.44
1689587	1.296	0.3425642	null	conserved hypothetical protein	PA5205	110	93.84
1689590	1.092	0.97087157	null	hypothetical protein	PA5212	66	92.18
1689592	0.737	0.6068904	null	probable secretion pathway ATPase	PA5210	393	92.92
1689595	0.973	0.67045105	null	hypothetical protein	PA5007	81	95.8
1689598	0.955	1.0459068	arcB	ornithine carbamoyltransferase, catabolic	PA5172	215	93.34
1689601	1.286	0.9553583	null	conserved hypothetical protein	PA5201	2	92.36
1689604	0.802	0.5947732	argE	acetylornithine deacetylase	PA5206	529	93.56
1689607	0.580	-0.54553926	gshA	glutamate--cysteine ligase	PA5203	73	93.87
1689610	1.081	1.0022173	gcvH1	glycine cleavage system protein H1	PA5214	94	93.15
1689613	0.822	0.6459304	gcvT1	glycine-cleavage system protein T1	PA5215	74	93.53
1689616	0.458	0.3056268	null	hypothetical protein	PA5222	126	93.1
1689619	1.009	0.91726184	null	hypothetical protein	PA5220	81	96.74
1689622	1.002	0.6821519	null	hypothetical protein	PA5209	62	94.63
1689625	0.921	0.6701435	null	hypothetical protein	PA5219	22	95.27
1689628	0.945	0.791265	ubiH	ubiH protein	PA5223	126	95.64
1689632	0.836	0.650299	null	probable FAD-dependent monooxygenase	PA5221	309	93.99
1689634	0.800	0.65874016	null	conserved hypothetical protein	PA5211	10	93.47
1689636	1.203	0.679136	null	probable transcriptional regulator	PA5218	85	95.87
1689639	0.741	0.6429083	null	conserved hypothetical protein	PA5228	99	94.16
1689642	1.045	0.89086735	null	conserved hypothetical protein	PA5227	151	96.31
1689644	0.955	-0.581884	gcvP1	glycine cleavage system protein P1	PA5213	96	93.8
1689647	0.711	0.4665927	null	hypothetical protein	PA5226	77	92.75
1689648	1.009	3.0184417	null	probable acyl-CoA dehydrogenase	PA5187	1548	92.76
1689651	1.107	0.96622765	null	hypothetical protein	PA5225	8	92.22
1689654	0.752	0.6041968	pepP	aminopeptidase P	PA5224	390	94.78
1689657	1.481	1.1217483	null	conserved hypothetical protein	PA5208	122	94.42
1689660	0.966	0.73456776	null	probable permease of ABC transporter	PA5230	60	93.45
1689663	0.719	0.598945	null	probable aromatic hydrocarbon reductase	PA5236	166	92.29
1689666	1.056	0.8720335	null	conserved hypothetical protein	PA5229	204	97.41
1689669	1.081	0.82609576	null	hypothetical protein	PA5233	209	93.61
1689672	0.942	0.92151415	trxA	thioredoxin	PA5240	210	92.52
1689673	0.624	0.41611356	null	conserved hypothetical protein	PA5237	81	96.87
1689676	1.174	0.83641547	null	probable ATP-binding/permease fusion ABC	PA5231	308	92.75
1689679	1.141	0.96066475	glpT	glycerol-3-phosphate transporter	PA5235	126	92.34
1689682	0.478	0.79920805	null	probable oxidoreductase	PA5234	109	92.11
1689685	1.308	1.1295624	null	conserved hypothetical protein	PA5245	9	92.33
1689688	1.072	0.8857911	null	conserved hypothetical protein	PA5232	905	96.2
1689691	0.603	0.5985378	rho	transcription termination factor Rho	PA5239	572	93.28
1689694	1.090	0.7387571	null	conserved hypothetical protein	PA5246	46	92.4
1689697	1.023	0.98610514	hemB	delta-aminolevulinic acid dehydratase	PA5243	1	93.55
1689700	1.536	1.2425406	null	probable O-antigen acetylase	PA5238	56	92.91
1689703	0.899	0.821315	ppk	polyphosphate kinase	PA5242	124	92.06
1689706	0.829	0.5371334	null	conserved hypothetical protein	PA5247	144	92.64
1689709	0.951	0.8247266	null	conserved hypothetical protein	PA5244	274	93.53
1689712	1.061	0.923903	null	hypothetical protein	PA5249	1	93.47
1689715	1.166	0.96434486	null	hypothetical protein	PA5248	67	94.79
1689718	1.152	0.99995023	null	probable peptidyl-prolyl cis-trans isomerase,	PA5254	27	94.98
1689721	0.502	0.48894176	algQ	Alginate regulatory protein AlgQ	PA5255	280	92.11
1689724	1.130	1.0867507	algP	alginate regulatory protein AlgP	PA5253	82	92.7
1689727	0.797	0.85608643	null	hypothetical protein	PA5258	25	92.43
1689730	0.773	0.64532465	null	hypothetical protein	PA5251	312	92.5

1689733	0.837	0.7395834	dsbH	disulfide bond formation protein	PA5256	186	95.01
1689736	0.992	0.82032835	null	conserved hypothetical protein	PA5250	6	92.37
1689739	0.717	0.49172854	null	probable binding protein component of ABC iron	PA5217	137	92.74
1689742	1.011	0.91058886	hemC	porphobilinogen deaminase	PA5260	230	93.81
1689745	0.165	0.052380968	algZ	alginate biosynthesis protein AlgZ/FimS	PA5262	33	94.29
1689748	1.427	1.1111103	null	hypothetical protein	PA5264	524	92.43
1689751	0.891	0.7270392	hemD	uroporphyrinogen-III synthetase	PA5259	101	93.6
1689754	0.767	0.6624365	null	hypothetical protein	PA5257	101	92.28
1689759	0.927	0.695074	hcpB	secreted protein Hcp	PA5267	402	92.55
1689760	0.854	1.21788	corA	magnesium/cobalt transport protein	PA5268	126	94.11
1689763	0.987	0.76854163	ppx	exopolyphosphatase	PA5241	24	95.52
1689766	1.071	0.8914629	null	hypothetical protein	PA5269	225	97.87
1689769	1.231	1.069671	null	hypothetical protein	PA5271	1	92.37
1689771	1.056	0.8930656	algR	alginate biosynthesis regulatory protein AlgR	PA5261	42	95.7
1689774	0.933	0.6919296	null	hypothetical protein	PA5273	251	93.68
1689777	1.014	0.80269384	cyaA	adenylate cyclase	PA5272	1	92.08
1689780	0.661	0.5280405	null	conserved hypothetical protein	PA5275	191	94.51
1689782	0.921	0.73637027	null	hypothetical protein	PA5270	33	93.54
1689785	1.009	-0.90159416	null	hypothetical protein	PA5265	40	95.91
1689788	1.057	0.9150616	lppL	Lipopeptide LppL precursor	PA5276	1	95.54
1689789	0.951	0.7679092	argH	argininosuccinate lyase	PA5263	58	94.46
1689792	0.788	0.63579637	dapF	diaminopimelate epimerase	PA5278	83	95.71
1689795	0.767	0.641693	null	conserved hypothetical protein	PA5279	45	93.94
1689798	0.656	0.55811393	null	hypothetical protein	PA5285	143	93.07
1689800	1.368	-0.15118468	null	hypothetical protein	PA5284	141	92.42
1689803	0.819	-0.06802888	null	probable transcriptional regulator	PA5283	95	94.14
1689806	0.543	0.4096362	null	conserved hypothetical protein	PA5286	310	94.71
1689808	0.713	0.31059527	null	probable ATP-binding component of ABC	PA5252	79	95.61
1689811	0.989	0.75425875	sss	site-specific recombinase Sss	PA5280	208	97.12
1689814	1.042	0.72070813	null	probable major facilitator superfamily (MFS)	PA5282	432	94.45
1689817	0.806	0.72173375	amtB	ammonium transporter AmtB	PA5287	103	92.23
1689820	0.971	0.6499383	null	hypothetical protein	PA5289	6	97.07
1689823	1.171	1.141533	glnK	nitrogen regulatory protein P-II 2	PA5288	42	93.25
1689826	1.052	2.1041064	pchP	phosphorylcholine phosphatase	PA5292	106	92.25
1689829	0.774	0.61765367	null	conserved hypothetical protein	PA5290	155	96.53
1689832	1.438	-2.09375	null	probable transcriptional regulator	PA5293	99	95.46
1689835	1.483	0.5967966	rep	ATP-dependent DNA helicase Rep	PA5296	2	92.93
1689838	0.712	0.39927286	null	xanthine phosphoribosyltransferase	PA5298	92	97.12
1689843	0.664	0.61645854	rnk	nucleoside diphosphate kinase regulator	PA5274	189	92
1689844	0.364	1.6700001	null	hypothetical protein	PA5294	63	93.6
1689847	1.246	1.0936713	lysA	diaminopimelate decarboxylase	PA5277	20	96.24
1689850	0.726	0.321295	null	probable hydrolase	PA5281	30	95.2
1689853	0.935	0.75988984	null	probable transcriptional regulator	PA5301	113	92.79
1689856	1.105	1.0038667	null	conserved hypothetical protein	PA5306	140	92.46
1689857	0.842	0.7353009	cycB	cytochrome c5	PA5300	32	92.9
1689860	0.728	0.59705675	dadX	catabolic alanine racemase	PA5302	12	92.31
1689863	0.851	0.6938638	lrp	leucine-responsive regulatory protein	PA5308	27	92.28
1689866	1.476	0.8633183	null	hypothetical protein	PA5295	48	96.92
1689870	0.773	0.705077	null	conserved hypothetical protein	PA5303	301	92.76
1689872	0.295	0.13714619	null	hypothetical protein	PA5307	378	92.1
1689875	0.878	-0.77095234	poxB	pyruvate dehydrogenase (cytochrome)	PA5297	106	95.08
1689878	0.948	0.76342183	null	probable oxidoreductase	PA5309	61	97.62
1689881	0.652	0.088888906	null	hypothetical protein	PA5314	81	95.3
1689884	1.000	0.08899156	null	conserved hypothetical protein	PA5310	179	95.82
1689887	0.869	0.9233495	rpmB	50S ribosomal protein L28	PA5316	79	94.12
1689891	1.138	0.80801904	null	conserved hypothetical protein	PA5305	37	96.3
1689892	0.662	0.6194564	null	probable permease of ABC iron transporter	PA5216	5	95.97
1689895	0.838	0.6838787	null	probable choline transporter	PA5291	254	92.52
1689898	1.083	0.9499714	null	probable aldehyde dehydrogenase	PA5312	655	95.75
1689901	1.218	1.6107447	null	hypothetical protein	PA5299	78	97.13
1689904	0.959	0.70437455	null	probable binding protein component of ABC	PA5317	132	93.5
1689907	0.803	0.6111797	algC	phosphomannomutase AlgC	PA5322	94	96.76
1689910	0.953	0.6776509	null	probable pyridoxal-dependent aminotransferase	PA5313	228	96.69
1689913	0.040	-0.26767	null	hypothetical protein	PA5318	327	94.68
1689916	1.181	0.26598063	null	probable transcriptional regulator	PA5324	18	93.43
1689919	1.271	0.9300763	null	hypothetical protein	PA5325	101	92.38
1689922	0.867	0.67944026	dut	deoxyuridine 5'-triphosphate	PA5321	370	92.24
1689924	1.009	1.6856661	null	probable cytochrome c(mono-heme type)	PA5328	306	93.29
1689927	1.009	1.4773059	null	hypothetical protein	PA5326	151	95.54
1689930	1.021	0.8609826	argB	acetylglutamate kinase	PA5323	12	96.85
1689933	1.077	0.7175308	null	conserved hypothetical protein	PA5329	13	92.08
1689936	0.816	0.69916475	null	hypothetical protein	PA5330	469	92.02
1689939	1.012	0.69897336	radC	DNA repair protein RadC	PA5319	377	94.13
1689942	0.896	0.7732955	crc	catabolite repression control protein	PA5332	69	92.23
1689945	1.009	1.2666667	null	probable oxidoreductase	PA5327	330	92.4
1689948	0.819	0.59350383	pyrE	orotate phosphoribosyltransferase	PA5331	46	95.01
1689951	0.939	0.9491782	rpoZ	RNA polymerase omega subunit	PA5337	3	92.64
1689954	0.965	0.7301019	rph	ribonuclease PH	PA5334	68	92.41
1689957	0.659	0.54404205	coaC	Phosphopantothenoylcysteine	PA5320	606	92.62
1689960	0.687	0.6008654	null	conserved hypothetical protein	PA5333	48	94.43
1689963	1.286	1.1665285	null	conserved hypothetical protein	PA5339	152	92.6
1689966	1.032	0.7593453	null	hypothetical protein	PA5341	8	93.9
1689969	0.833	0.72304255	null	hypothetical protein	PA5340	135	92.65
1689972	0.868	0.69769967	null	probable transcriptional regulator	PA5342	415	96.54
1689975	0.963	0.58444566	null	conserved hypothetical protein	PA5335	42	96.63
1689978	0.787	0.62075436	null	probable transcriptional regulator	PA5344	528	92.8
1689981	0.917	0.47626907	gmk	guanylate kinase	PA5336	216	94.17
1689984	0.649	0.46525916	null	hypothetical protein	PA5343	468	97.5
1689987	0.621	0.5814892	null	probable DNA-binding protein	PA5348	38	92.85
1689989	0.781	0.49628195	recG	ATP-dependent DNA helicase RecG	PA5345	4	96.41
1689992	0.966	0.8384773	rubA2	Rubredoxin 2	PA5350	10	93.95
1689993	0.574	0.50379074	rubA1	Rubredoxin 1	PA5351	79	94.97
1689994	1.009	1.2178442	null	conserved hypothetical protein	PA5352	150	97.18
1689997	1.065	0.8361111	null	probable 3-hydroxyacyl-CoA dehydrogenase	PA5188	84	95.52
1690000	1.499	0.96650016	null	hypothetical protein	PA5347	49	94.32
1690003	0.913	0.4952969	null	probable rubredoxin reductase	PA5349	246	93.92
1690006	1.199	1.048774	null	hypothetical protein	PA5346	254	95.51
1690009	1.259	0.831141	glcE	glycolate oxidase subunit GlcE	PA5354	180	95.5
1690012	1.453	-1.3840002	glcF	glycolate oxidase subunit GlcF	PA5353	140	93.49
1690015	0.862	0.7464764	spoT	guanosine-3',5'-bis(diphosphate)	PA5338	141	94.68
1690018	0.871	0.69903654	glcC	transcriptional regulator GlcC	PA5356	164	96.64
1690021	1.174	0.92915356	phoR	two-component sensor PhoR	PA5361	1	92.87
1690024	1.231	0.81230277	null	hypothetical protein	PA5359	378	93.39
1690027	0.843	0.6767227	null	probable two-component response regulator	PA5364	60	92.57
1690030	0.755	0.57407534	null	hypothetical protein	PA5363	156	92.05
1690033	0.982	0.69945866	ubiA	4-hydroxybenzoate-octaprenyl transferase	PA5358	202	93.52
1690036	1.185	1.0462068	null	conserved hypothetical protein	PA5362	47	92.63
1690039	1.200	0.67004496	phoB	two-component response regulator PhoB	PA5360	79	95.06
1690042	1.190	0.46542683	phoU	phosphate uptake regulatory protein PhoU	PA5365	479	92

1690045	1.074	0.90987414	null	conserved hypothetical protein	PA5371	86	92.68
1690048	0.756	0.034391165	glcD	glycolate oxidase subunit GlcD	PA5355	332	94.18
1690051	1.296	0.88539684	null	probable major facilitator superfamily (MFS)	PA5370	1	97.44
1690054	1.014	0.7530901	betA	choline dehydrogenase	PA5372	38	93.1
1690057	1.042	0.8546646	betI	transcriptional regulator BetI	PA5374	48	95.86
1690060	1.561	1.5716203	pstB	ATP-binding component of ABC phosphate	PA5366	2	93.2
1690063	1.035	-0.018141747	null	probable permease of ABC transporter	PA5377	98	92.04
1690066	1.238	1.0058677	null	hypothetical protein	PA5378	3	92.54
1690069	1.081	0.5124132	null	probable ATP-binding component of ABC	PA5376	3	92.21
1690072	0.766	0.6378121	null	probable transcriptional regulator	PA5380	1	93.36
1690075	0.955	9.58394	null	hypothetical protein	PA5381	64	94.07
1690078	0.948	0.9545706	null	hypothetical protein	PA5369	85	93.33
1690081	1.405	0.6371304	null	probable transcriptional regulator	PA5382	4	93.08
1690084	0.797	-0.29804498	null	conserved hypothetical protein	PA5383	1	95.5
1690087	0.943	0.7937395	pstC	membrane protein component of ABC phosphate	PA5368	68	93.65
1690090	1.062	0.91758126	betB	betaine aldehyde dehydrogenase	PA5373	187	96.88
1690093	1.142	1.1533217	null	probable 3-hydroxyacyl-CoA dehydrogenase	PA5386	672	94.66
1690096	1.009	1.0177263	null	conserved hypothetical protein	PA5387	65	95.98
1690099	1.845	0.8346709	null	hypothetical protein	PA5385	46	92.7
1690102	1.326	0.93579066	pstA	membrane protein component of ABC phosphate	PA5367	317	93.94
1690105	1.009	3.3083336	sdaB	L-serine dehydratase	PA5379	392	92.3
1690108	1.407	0.9746208	null	probable lipolytic enzyme	PA5384	541	94.91
1690111	0.785	0.58770865	null	hypothetical protein	PA5357	208	96.6
1690114	1.031	1.2433631	null	probable peptidic bond hydrolase	PA5390	30	96.78
1690117	1.133	2.7142856	null	hypothetical protein	PA5391	210	94.82
1690119	1.009	2.9067101	null	hypothetical protein	PA5388	168	92.53
1690122	1.009	-1.3678882	null	conserved hypothetical protein	PA5395	100	95.61
1690125	1.297	1.011722	null	probable transcriptional regulator	PA5389	27	95.23
1690128	0.755	2.4337485	null	conserved hypothetical protein	PA5393	3	97.44
1690131	3.345	1.1504525	null	hypothetical protein	PA5397	145	93.08
1690134	1.450	0.22624478	null	conserved hypothetical protein	PA5392	38	92.82
1690137	0.848	-3.6196506	null	probable transcriptional regulator	PA5403	18	92.4
1690139	0.977	0.98068845	null	hypothetical protein	PA5405	1	93.7
1690141	1.009	4.1518717	null	hypothetical protein	PA5404	208	93.1
1690144	1.009	0.7476263	null	probable FMN oxidoreductase	PA5398	111	92.13
1690147	1.272	4.447196	null	hypothetical protein	PA5401	87	96.55
1690150	1.343	0.50712615	null	probable electron transfer flavoprotein alpha	PA5400	2	96.14
1690153	1.348	0.9315149	null	probable major facilitator superfamily (MFS)	PA5311	13	92.6
1690156	1.321	0.77671385	null	hypothetical protein	PA5408	35	95.74
1690158	1.453	0.76882744	null	hypothetical protein	PA5406	30	93.2
1690161	1.118	0.88016	ltaA	low specificity l-threonine aldolase	PA5413	10	92.16
1690164	0.869	0.6919712	null	hypothetical protein	PA5407	109	92.27
1690166	1.371	0.2943746	null	hypothetical protein	PA5402	3	92.56
1690169	1.125	0.6397848	null	probable transport protein	PA4334	141	92.87
1690172	0.713	0.5946144	null	hypothetical protein	PA5412	534	97.16
1690175	1.035	0.75999343	null	hypothetical protein	PA5409	8	93.34
1690178	1.096	1.4166667	null	probable ring hydroxylating dioxygenase,	PA5410	309	95.4
1690181	1.020	0.8222159	null	hypothetical protein	PA5414	105	95.51
1690184	0.593	1.7312925	cls	cardiolipin synthase	PA5394	8	93.25
1690187	1.109	1.0285277	dadA	D-amino acid dehydrogenase, small subunit	PA5304	299	93.32
1690190	1.009	1.7446274	soxB	sarcosine oxidase beta subunit	PA5416	136	93.08
1690193	1.076	0.18282828	null	probable ferredoxin	PA5411	39	97.24
1690196	1.215	-5.0992455	null	hypothetical protein	PA5396	283	92.11
1690199	1.082	-1.4515873	fdhA	glutathione-independent formaldehyde	PA5421	1	92.47
1690202	0.981	-0.619943	soxG	sarcosine oxidase gamma subunit	PA5419	250	93.12
1690205	0.869	0.59638256	null	hypothetical protein	PA5422	3	92.43
1690208	0.749	0.4743897	null	conserved hypothetical protein	PA5424	62	92.51
1690210	1.009	0.91053116	soxA	sarcosine oxidase alpha subunit	PA5418	125	94.61
1690213	1.304	1.1338621	adhA	alcohol dehydrogenase	PA5427	1	93.91
1690216	1.052	-0.3494318	purU2	formyltetrahydrofolate deformylase	PA5420	90	95.25
1690219	1.009	-0.5	null	probable acetyltransferase	PA5432	336	95.92
1690222	1.028	0.8002564	purK	phosphoribosylaminoimidazole carboxylase	PA5425	178	92.49
1690226	1.455	0.8485484	null	conserved hypothetical protein	PA5433	128	96.18
1690228	1.089	0.8794777	null	probable transcriptional regulator	PA5431	932	94.43
1690231	1.009	1.2848485	null	probable ferredoxin	PA5399	147	92.07
1690234	1.177	1.3573749	null	probable transcriptional regulator	PA5428	395	96.09
1690237	1.057	0.88043433	mtr	tryptophan permease	PA5434	1	96.89
1690240	0.633	0.46989116	null	probable transcriptional regulator	PA5437	16	92.67
1690243	0.770	0.61841977	null	probable transcriptional regulator	PA5438	8	95.11
1690246	1.009	2.6232638	soxD	sarcosine oxidase delta subunit	PA5417	21	93.64
1690249	1.111	-0.27549398	null	probable peptidase	PA5440	168	95.11
1690252	0.880	0.64166343	null	hypothetical protein	PA5430	305	93.53
1690255	1.105	0.9163723	purE	phosphoribosylaminoimidazole carboxylase,	PA5426	23	92.5
1690258	0.897	0.69286793	null	conserved hypothetical protein	PA5444	230	93.71
1690261	0.743	0.52191347	null	hypothetical protein	PA5441	204	94.96
1690264	1.154	0.93629676	aspA	aspartate ammonia-lyase	PA5429	45	94.02
1690267	1.062	0.9988551	wbpY	glycosyltransferase WbpY	PA5448	81	92.45
1690270	0.832	0.6275956	wbpZ	glycosyltransferase WbpZ	PA5447	141	97.34
1690273	1.189	1.3448467	null	hypothetical protein	PA5446	7	92.23
1690276	0.883	0.5083933	null	probable glucose-6-phosphate dehydrogenase	PA5439	3	92.61
1690279	1.025	0.5465105	glyA1	serine hydroxymethyltransferase	PA5415	100	95.19
1690282	1.039	0.82280064	null	probable coenzyme A transferase	PA5445	17	95.83
1690285	0.722	0.5769665	wzm	membrane subunit of A-band LPS efflux	PA5451	14	92.29
1690288	0.802	0.46897277	wzt	ABC subunit of A-band LPS efflux transporter	PA5450	62	95.76
1690291	1.092	0.95020837	null	probable biotin carboxylase subunit of a	PA5436	1129	92.77
1690294	0.973	0.73991823	rmd	oxidoreductase Rmd	PA5454	39	92.22
1690297	0.649	2.6428802	gmd	GDP-mannose 4,6-dehydratase	PA5453	242	97.97
1690300	0.851	0.61251414	null	hypothetical protein	PA5456	12	92.04
1690303	1.209	1.1381838	null	hypothetical protein	PA5460	33	97.52
1690304	0.575	0.44178724	null	hypothetical protein	PA5457	351	93.28
1690307	0.824	0.6770845	null	hypothetical protein	PA5462	72	92.67
1690308	1.076	0.79999304	null	hypothetical protein	PA5463	71	93.58
1690310	0.933	0.872598	null	hypothetical protein	PA5461	97	93.24
1690312	1.000	0.32029107	null	conserved hypothetical protein	PA5442	37	93.91
1690315	0.661	0.48315686	null	hypothetical protein	PA5455	257	95.43
1690318	1.087	0.82802886	null	hypothetical protein	PA5465	59	95.78
1690321	1.193	0.91204387	wbpX	glycosyltransferase WbpX	PA5449	105	94.7
1690324	0.591	0.44287133	null	hypothetical protein	PA5467	246	97.23
1690326	1.207	0.9116179	null	hypothetical protein	PA5466	123	92.63
1690329	0.725	0.3949836	null	hypothetical protein	PA5458	49	92.22
1690332	1.004	0.8348281	betT1	choline transporter BetT	PA5375	1	93.63
1690335	0.913	0.697476	null	hypothetical protein	PA5459	464	93.55
1690338	0.821	0.57923937	null	probable peptide chain release factor	PA5470	206	96.89
1690341	0.811	0.57948244	null	hypothetical protein	PA5472	328	92.63
1690344	0.891	0.5273462	null	hypothetical protein	PA5471	26	95.5
1690347	2.052	5.190589	null	conserved hypothetical protein	PA5469	106	97.94
1690350	1.451	1.2591925	null	hypothetical protein	PA5475	227	92.73
1690353	1.317	1.330034	null	hypothetical protein	PA5477	177	92.76
1690356	1.014	0.89650613	null	probable transcarboxylase subunit	PA5435	85	94.84

1690359	1.075	0.12917438	null	hypothetical protein	PA5480	119	92.75
1690360	0.756	0.43976155	null	hypothetical protein	PA5482	25	92.57
1690361	0.201	-0.12715039	null	hypothetical protein	PA5481	128	95.25
1690363	1.586	0.77017546	null	probable citrate transporter	PA5468	27	92.03
1690366	1.601	1.3894886	gltP	proton-glutamate symporter	PA5479	328	92.52
1690369	1.624	0.35382885	null	conserved hypothetical protein	PA5485	60	95.53
1690372	0.847	0.53220963	null	conserved hypothetical protein	PA5266	149	94.03
1690375	1.025	0.94799834	null	conserved hypothetical protein	PA5478	151	95.08
1690378	0.771	0.76119024	null	hypothetical protein	PA5488	34	95.51
1690381	0.901	0.69439477	null	conserved hypothetical protein	PA5486	206	95.45
1690384	0.779	0.53086007	null	hypothetical protein	PA5464	109	94.24
1690387	0.791	0.67767406	null	probable cytochrome	PA5491	59	95.02
1690389	0.959	0.9286148	dsbA	thiol:disulfide interchange protein DsbA	PA5489	126	92.67
1690392	0.652	0.44622236	null	hypothetical protein	PA5487	266	94.34
1690395	1.318	1.1975851	null	hypothetical protein	PA5494	170	93.32
1690398	0.890	0.9046074	cc4	cytochrome c4 precursor	PA5490	544	92.12
1690399	0.801	0.26617938	null	conserved hypothetical protein	PA5492	162	95.03
1690402	0.786	0.36028293	null	conserved hypothetical protein	PA5473	339	92.01
1690405	1.093	0.78534776	null	hypothetical protein	PA5496	177	92.64
1690408	1.367	0.8661173	algB	two-component response regulator AlgB	PA5483	580	95.58
1690411	0.954	0.79245555	np20	transcriptional regulator np20	PA5499	287	95.5
1690413	1.072	0.92722577	citA	citrate transporter	PA5476	125	94.04
1690416	0.799	0.588568	null	hypothetical protein	PA5497	16	94.14
1690419	0.907	0.75038564	znuC	zinc transport protein ZnuC	PA5500	26	95.04
1690422	0.819	0.6093914	znuB	permease of ABC zinc transporter ZnuB	PA5501	61	95.34
1690425	0.688	0.30074707	null	probable adhesin	PA5498	608	92.12
1690428	1.254	1.0871073	null	probable TonB-dependent receptor	PA5505	89	97.2
1690431	0.989	0.8605469	null	hypothetical protein	PA5507	107	95.27
1690434	1.018	0.74758583	null	hypothetical protein	PA5423	112	94.76
1690437	0.721	0.62828207	null	hypothetical protein	PA5502	600	93.79
1690440	0.998	0.80662966	null	hypothetical protein	PA5509	208	96.22
1690443	1.019	0.8319937	null	probable permease of ABC transporter	PA5504	125	94.59
1690446	0.799	0.57887375	polA	DNA polymerase I	PA5493	440	94.68
1690449	0.822	-1.97206	poxA	hypothetical protein	PA5513	142	94.01
1690452	0.855	0.6256148	null	probable ATP-binding component of ABC	PA5503	848	94.39
1690455	0.794	0.4998412	null	probable transporter	PA5510	206	92.83
1690458	1.033	0.7819003	null	hypothetical protein	PA5515	88	92.5
1690460	1.000	1.0997871	null	probable beta-lactamase	PA5514	1	93.56
1690463	0.958	0.76621497	null	hypothetical protein	PA5506	669	94.43
1690466	0.953	0.77857155	null	probable two-component sensor	PA5484	9	93.19
1690469	12.620	7.4189577	pdxY	pyridoxamine kinase	PA5516	279	92.11
1690472	0.776	0.58139086	null	probable glutamine synthetase	PA5508	364	94.04
1690475	1.078	1.814872	null	hypothetical protein	PA5520	210	94.45
1690478	0.936	0.7041946	null	probable short-chain dehydrogenase	PA5521	192	92.68
1690481	1.065	0.49288458	null	probable aminotransferase	PA5523	5	93.11
1690484	1.107	0.89130294	null	hypothetical protein	PA5526	60	92.99
1690487	1.270	1.0447123	null	conserved hypothetical protein	PA5519	396	92.99
1690488	1.071	0.78458506	null	conserved hypothetical protein	PA5517	64	92.97
1690491	1.414	0.79276216	null	probable potassium efflux transporter	PA5518	4	92.6
1690494	6.207	2.842523	null	probable short-chain dehydrogenase	PA5524	6	92.58
1690497	0.882	0.7231386	null	hypothetical protein	PA5527	20	93.03
1690500	0.699	0.20116988	null	probable transcriptional regulator	PA5525	73	92.58
1690503	0.991	0.8623711	tonB	TonB protein	PA5531	80	95.95
1690506	0.825	0.5695239	null	probable metalloprotease	PA5474	110	96.75
1690509	1.141	0.9261389	null	hypothetical protein	PA5532	187	97.55
1690512	0.923	1.8505557	null	hypothetical protein	PA5533	48	92.33
1690515	0.792	0.38081875	wbpW	phosphomannose isomerase/GDP-mannose WbpW	PA5452	176	94.37
1690518	1.009	1.5638204	null	conserved hypothetical protein	PA5535	57	92.76
1690521	1.009	0.5766789	null	conserved hypothetical protein	PA5536	19	95.44
1690524	0.558	-0.8128788	null	probable glutamine synthetase	PA5522	353	94.48
1690527	1.160	0.841698	null	hypothetical protein	PA5537	99	95.35
1690530	0.747	0.64925134	null	hypothetical protein	PA5528	273	92.34
1690533	0.941	0.6627501	null	probable MFS dicarboxylate transporter	PA5530	16	96.29
1690536	1.392	0.7544152	amiA	N-acetylmuramoyl-L-alanine amidase	PA5538	37	96.94
1690539	1.007	0.6203475	null	hypothetical protein	PA5543	64	93.44
1690541	0.298	0.48349243	null	probable two-component sensor	PA5512	351	93.67
1690544	0.887	0.9939755	null	hypothetical protein	PA5540	72	92.41
1690547	0.859	0.72588015	null	probable sodium/proton antiporter	PA5529	30	92.36
1690550	1.045	0.821177	null	conserved hypothetical protein	PA5547	59	92.4
1690553	1.029	0.8890998	thrB	homoserine kinase	PA5495	664	92.61
1690556	1.009	1.7017567	null	hypothetical protein	PA5542	170	92.4
1690559	0.979	0.36858553	null	conserved hypothetical protein	PA5545	29	92.65
1690562	1.382	1.0572798	null	conserved hypothetical protein	PA5546	56	95.32
1690565	0.788	0.5311201	glmR	GlmR transcriptional regulator	PA5550	73	93.89
1690568	0.951	0.6711377	null	hypothetical protein	PA5534	229	92.76
1690571	0.723	0.6738842	atpG	ATP synthase gamma chain	PA5555	163	92.62
1690574	0.922	0.61932313	null	probable major facilitator superfamily (MFS)	PA5548	82	96.04
1690577	0.548	0.3761659	null	hypothetical protein	PA5551	272	92.86
1690580	0.832	0.7314946	atpD	ATP synthase beta chain	PA5554	17	92.79
1690583	0.800	0.80094653	atpI	ATP synthase protein I	PA5561	2	93.27
1690586	0.853	0.92198545	atpF	ATP synthase B chain	PA5558	51	93
1690589	0.812	0.8629945	atpE	atp synthase C chain	PA5559	15	92.8
1690591	0.715	0.7507241	atpH	ATP synthase delta chain	PA5557	1	93.71
1690594	0.770	0.7356517	atpA	ATP synthase alpha chain	PA5556	57	92.31
1690597	1.096	-0.7529412	glmS	glucosamine--fructose-6-phosphate	PA5549	97	96.39
1690600	0.516	0.5008441	atpC	ATP synthase epsilon chain	PA5553	194	92.26
1690603	6.068	-0.3688272	null	hypothetical protein	PA5566	111	96.32
1690606	0.736	0.4971796	spoOJ	chromosome partitioning protein Spo0J	PA5562	51	95.21
1690609	1.014	0.8818706	gidB	glucose inhibited division protein B	PA5564	197	92.47
1690612	0.712	0.62429565	atpB	ATP synthase A chain	PA5560	106	92.31
1690615	0.934	0.8130349	soj	chromosome partitioning protein Soj	PA5563	142	92.37
1690618	1.009	2.8921044	null	conserved hypothetical protein	PA5567	107	95.33
1690621	0.850	0.83401835	rpmH	50S ribosomal protein L34	PA5570	36	96.1
1690622	0.686	0.7155313	rnpA	ribonuclease P protein component	PA5569	1	92.61
1690625	1.252	0.7142165	null	conserved hypothetical protein	PA5544	5	92.37
1690628	0.743	0.6413321	null	conserved hypothetical protein	PA5568	267	96.06
1690631	0.848	0.6479276	gidA	glucose-inhibited division protein A	PA5565	70	93.81
1690634	1.132	0.9431922	glmU	glucosamine-1-phosphate	PA5552	38	95.71
1690637	0.754	3.6336207	null	hypothetical protein	PA5539	1	92.78
1690640	0.965	0.04000001	pyrQ	dihydroorotase	PA5541	193	93.62
1690643	0.610	0.4683308	null	probable two-component response regulator	PA5511	275	92.49
1690646	0.734	0.5593416	uvrD	DNA helicase II	PA5443	152	93.93
